# Modeling Dynamics of Human Gut Microbiota Derived from Gluten Metabolism: Obtention, Maintenance and Characterization of Complex Microbial Communities

**DOI:** 10.3390/ijms25074013

**Published:** 2024-04-04

**Authors:** Yaiza Carnicero-Mayo, Luis E. Sáenz de Miera, Miguel Ángel Ferrero, Nicolás Navasa, Javier Casqueiro

**Affiliations:** 1 Área de Microbiología, Facultad de Ciencias Biológicas y Ambientales, Universidad de León, 24007 León, Spain; ycarm@unileon.es; 2 Área de Genética, Facultad de Ciencias Biológicas y Ambientales, Universidad de León, 24007 León, Spain; luis.saenzdemiera@unileon.es; 3 Área de Bioquímica y Biología Molecular, Facultad de Veterinaria, Universidad de León, 24007 León, Spain; ma.ferrero@unileon.es (M.Á.F.); nnavm@unileon.es (N.N.)

**Keywords:** gluten, gluten metabolism, gut microbiota, in vitro digestion, microbial communities

## Abstract

Western diets are rich in gluten-containing products, which are frequently poorly digested. The human large intestine harbors microorganisms able to metabolize undigested gluten fragments that have escaped digestion by human enzymatic activities. The aim of this work was obtaining and culturing complex human gut microbial communities derived from gluten metabolism to model the dynamics of healthy human large intestine microbiota associated with different gluten forms. For this purpose, stool samples from six healthy volunteers were inoculated in media containing predigested gluten or predigested gluten plus non-digested gluten. Passages were carried out every 24 h for 15 days in the same medium and community composition along time was studied via V3–V4 16S rDNA sequencing. Diverse microbial communities were successfully obtained. Moreover, communities were shown to be maintained in culture with stable composition for 14 days. Under non-digested gluten presence, communities were enriched in members of *Bacillota*, such as *Lachnospiraceae*, *Clostridiaceae*, *Streptococcaceae*, *Peptoniphilaceae*, *Selenomonadaceae* or *Erysipelotrichaceae*, and members of *Actinomycetota*, such as *Bifidobacteriaceae* and *Eggerthellaceae*. Contrarily, communities exposed to digested gluten were enriched in *Pseudomonadota*. Hence, this study shows a method for culture and stable maintenance of gut communities derived from gluten metabolism. This method enables the analysis of microbial metabolism of gluten in the gut from a community perspective.

## 1. Introduction

The human gastrointestinal tract comprises the most numerous and complex ecosystem in the human body. Humans have co-evolved with their microbiota for thousands of years, establishing a symbiotic relationship [1]. Traditionally, microorganisms from human microbiota have been isolated for their study. Despite progress made in recent years, not all members of the microbiota can be isolated in pure culture using standard laboratory media and conditions [2,3]. Pure culture is unquestionably a fundamental technique in microbiology research. However, in the environment, microorganisms do not usually live isolated from each other. Instead, they are commonly part of complex multispecies communities in which different kinds of intra- and interspecies relationships are established [4,5,6]. Bacteria are adapted to living in these complex communities, and, as a consequence, their metabolic needs and functions are influenced by other members of the community [7,8,9,10]. Indeed, interactions occurring within communities at this micro-scale may give rise to macroscopic-appreciable effects. An illustrative example is how human gut microbial communities influence a host’s health [6,11,12]. Gut microbiota plays a relevant role in the digestion, absorption and energy extraction from food components, as well as in the synthesis of relevant molecules, such as vitamins, essential amino acids or short-chain fatty acids. Additionally, gut microbiota is crucial for immune system development and regulation, as well as for protection against pathogens. Gut microbiota influences the enteric nervous system and also the central nervous system via the gut–brain axis [11]. The diversity and importance of functions performed by gut microbiota are translated into a wide range of health implications. Imbalances in the gut microbiota have been associated not only with gastrointestinal disorders but also with several other conditions, such as diabetes, cardiovascular, metabolic and respiratory diseases, neurological conditions and cancer [11,12].

In communities, microorganisms may interact in various ways. Several studies highlight the importance of cooperative relationships in host-associated communities [13,14,15]. Indeed, the Black Queen Hypothesis proposed by Morris et al. describes how selective advantages can emerge when dependence relationships are established among community members [16]. This model supports the complex metabolic interdependence relationships existing in communities [9,10,13,17,18]. Cooperative interactions are even suggested to hold microbial diversity in the human gut [19]. The presence of specific bacteria that facilitate the proliferation of other microorganisms frequently yields benefits for microbial communities. For instance, the existence of complementary metabolic pathways results in a wider range of potentially available nutrient sources from the environment [9,13,20].

However, the existence of metabolic interdependency relationships complicates the study of communities and their members. The majority of microorganisms that are part of these communities are unlikely to thrive when attempted to grow in pure culture without their partners’ presence, unless proper nutritional requirements and signaling molecules, which are little known, are supplied [21,22,23]. Moreover, interactions occurring in communities may lead to the emergence of new functions that are not performed by the community members individually [24,25]. A further reason why the study of communities is challenging is that these community emergent properties are difficult to predict based on the community composition. Thus, not only the study of the individual microorganisms but also of the community-intrinsic emergent properties is relevant for understanding the role of microbiota in human health [26].

The intestinal microbiota is a dynamic ecosystem shaped by genetic but also a multitude of environmental factors (age, antibiotic usage, lifestyle, disease, etc.). One of the most important factors by which microbiota can be modulated is the diet [27,28,29,30,31]. In the last years, low-gluten or gluten-free diets have gained popularity as healthy diets. However, there is no solid evidence that gluten is harmful to healthy individuals, nor that a gluten-free diet would improve health [32,33]. Several works have shown that a high diversity of microorganisms able to metabolize gluten is present in human intestinal microbiota [34,35,36,37]. Nevertheless, these microorganisms have been isolated and studied using pure cultures and, given the significance of microbial interactions in the human gut, the current study addresses the obtention and study of gut microbial communities derived from gluten metabolism.

The objective of this study was to obtain and maintain microbial communities from the human distal gut derived from gluten metabolism, as well as to evaluate their diversity and composition. Through this approach, we aimed to model the dynamics of healthy human large intestine microbiota associated with digested gluten and digested plus undigested gluten.

## 2. Results

**Figure 1:**
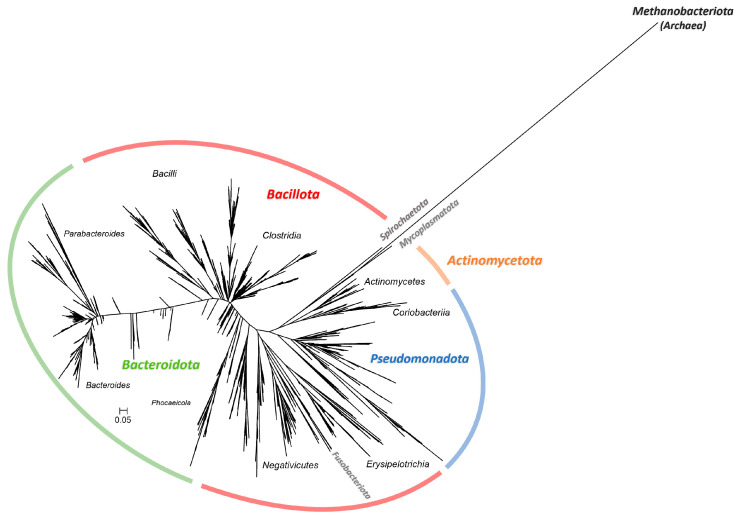
Approximately-maximum-likelihood phylogenetic tree of the 2336 studied OTUs performed using the GTR evolutionary model.

Communities were obtained and maintained through serial passaging in two media: one containing non-digested gluten (NDG) and digested gluten (MCG-3 medium) and the other containing only digested gluten (MCG-3B medium). Community composition and dynamics along passages were studied via partial 16S rDNA sequencing. A total of 8,685,351 clean reads, corresponding to 2336 different Operational Taxonomic Units (OTUs) were detected. Selected OTUs are represented in the phylogenetic tree of Figure 1. Based on the number of branches, bifurcations and distances observed in this metric phylogram, a notable phylogenetical and, thus, taxonomical diversity could be initially perceived. Indeed, 2 domains, 8 phyla, 15 classes, 30 orders, 55 families and 125 genera were detected in the global of the samples.

### 2.1. Alpha Diversity Was Affected by Non-Digested Gluten Presence in the Culture Media

**Figure 2:**
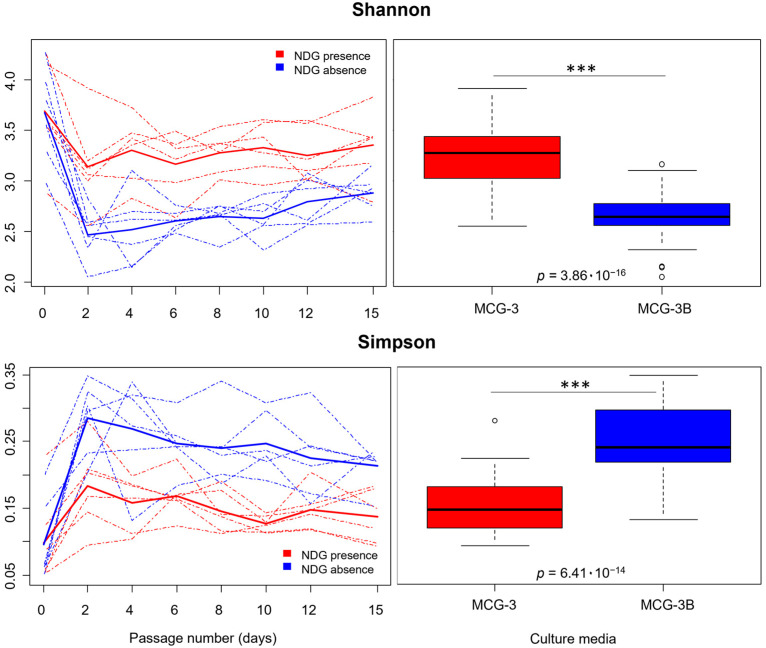
Graphical representation of values obtained for Shannon diversity and Simpson’s dominance. On the left, alpha diversity indices are represented for each time point and culture with dashed lines, while thicker continuous lines represent the mean values for all samples in MCG-3 medium cultures (red lines) or MCG-3B cultures (blue lines). On the right, boxplots represent the distribution of Shannon and Simpson’s values from passage 2 to passage 15 for each culture media. The grade of significance between differences observed for each culture media, as well as the *p*-value obtained from the analysis of variance (ANOVA) performed after the generation of linear models, are indicated inside the graph. Significance: *** (*p* < 0.001).

Regarding α-diversity, Shannon index values for communities cultured in MCG-3 and MCG-3B clearly dropped from passage 0 (initial inoculum grown for 24 h) compared to passages 2 to 15 (Figure 2). The descent in Shannon was significative either for MCG-3 (*p* = 4.14 × 10^−3^) and for MCG-3B media (*p* = 1.07 × 10^−10^), as shown by analysis of variance (ANOVAs) performed after generation of linear models (Table S1). For all volunteer samples, the decrease in diversity was more accentuated after two days of culture in the MCG-3B medium, compared to MCG-3, suggesting that NDG presence favored a higher diversity of communities.

This contribution of NDG to the diversity of communities was also observed when comparing α-diversity indicators for both kinds of communities. Average Shannon values from passages 2 to 15 were significantly higher under NDG presence compared to its absence. These results are in concordance with Simpson’s dominance index, which exhibited significantly higher values for communities cultured without NDG presence (Table 1; Figure 2). Both kinds of media harbored a notable phylogenetic diversity, as shown by the negative values of NRI (Net Relatedness Index). Higher phylogenetic overdispersion was observed for MCG-3B, tough (Table 1). All differences stated for α-diversity indices were strongly significative (*p* < 0.001), as shown by ANOVAs performed after the generation of linear models, including NDG presence as a factor (Table 1).

### 2.2. Non-Digested-Gluten Presence Affected Community Composition at All Taxonomic Levels

When communities were evaluated from passages 2 to 15, the *Bacteria* domain accounted for more than 99.99% of reads. The remaining reads corresponded to the single OTU_83, identified as *Methanobrevibacter smithii*, belonging to the *Methanobacteriota* phylum (syn. *Euryarchaeota*) from the *Archaea* domain. Overall, the most abundant phyla were *Pseudomonadota* (syn. *Proteobacteria*), relating on average to 43.78% of reads, and *Bacillota* (syn. *Firmicutes*; 34.45%), distantly followed by *Bacteroidota* (syn. *Bacteroidetes*; 19.62%) and *Actinomycetota* (syn. *Actinobacteria*; 1.68%).

Stability along subculturing was analyzed for each of the taxonomic groups by performing linear models from passages 2 to 15. These models were generated independently for taxa in MCG-3 and in MCG-3B, including the passage number as a variable. Data from passage 0 were excluded from the analysis since genetic material from all microorganisms present in the fecal inoculum could be detected, regardless of its ability to grow on the culture media. ANOVAs with subsequent False Discovery Rate (FDR) correction performed after multiple model generations showed no taxonomic groups for which abundances changed over time for communities in MCG-3B (*p*_FDR_ > 0.05). For communities cultured in MCG-3, only six taxa statistically varied along time (*p*_FDR_ < 0.05), which represents 2.58% of total number of taxa detected. This time dependence was not highly significant, however, as *p*_FDR_ ≥ 0.02 for all six taxa (Table S2). Therefore, taxa showed reasonably stable values along time from passages 2 to 15. Then, further models were performed to address the effect of NDG presence on the relative abundance of the different taxa present within communities.

**Figure 3:**
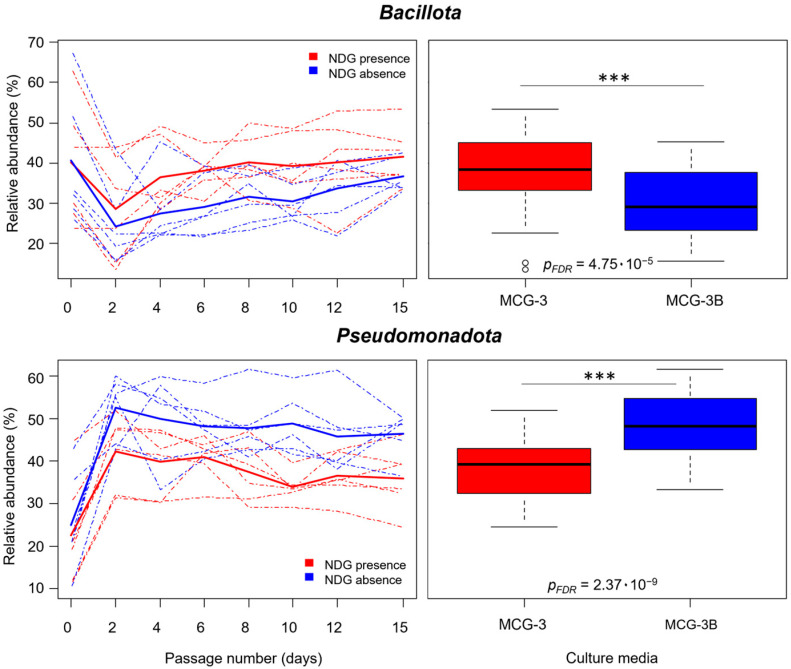
On the left, relative abundance along time for *Bacillota* and *Pseudomonadota* in communities grown in NDG presence (red lines) or absence (blue lines) is shown. Solid lines represent the average values for the different communities cultured. Boxplots on the right represent the distribution of relative abundance values for these phyla from passages 2 to 15. Significance for differences observed between both conditions is also shown, based on *p*_FDR_ values obtained from ANOVA tests performed after the generation of mixed linear models. Significance: *** (*p* < 0.001).

The effect of NDG presence on the different taxonomic groups was evaluated through the performance of multiple linear mixed models, including NDG presence as a factor and the individual volunteer as a random effect. ANOVAs followed by FDR correction of *p* values showed that 75 taxa (32.19% of the taxa detected) were significantly affected by NDG presence (*p*_FDR_ < 0.05). Particularly, these models showed that *Bacillota* relative abundance from passages 2 to 15 was significantly higher for communities cultured with NDG (38.18% average reads) compared to communities cultured with only digested gluten (30.72% average reads; *p*_FDR_ = 4.75 × 10^−5^) (Figure 3). *Actinomycetota* were also enriched in communities cultured in the medium containing NDG (2.35%) compared to NDG absence (1.01%; *p*_FDR_ = 2.56 × 10^−2^). Contrarily, microorganisms belonging to *Pseudomonadota* had lower relative abundance in samples from communities cultured under NDG presence (38.50%) compared to NDG absence (49.06%; *p*_FDR_ = 2.37 × 10^−9^) (Figure 3; Table S2).

Communities obtained in NDG presence were also significantly enriched in *Lachnospiraceae* and *Clostridiaceae 1* families, as well as in *Streptococcaceae*, *Peptoniphilaceae*, *Selenomonadaceae* and *Erysipelotrichaceae* families, all belonging to *Bacillota*. Whereas *Bacteroidota* relative abundance was not affected by NDG, communities cultured with NDG were significantly enriched in *Porphyromonadaceae*, *Odoribacteraceae* and *Prevotellaceae* families. Within *Actinomycetota*, enrichment in *Bifidobacteriaceae* and *Eggerthellaceae* populations was detected when NDG was present. Interestingly, *Sutterellaceae* was the only family from *Pseudomonadota* being statistically enriched in NDG presence (Table 2). Due to the interindividual variability, which is intrinsic to human microbiota, not all the 125 detected genera were present in all the individuals participating in the study. The most abundant genera, as well as other less abundant genera showing remarkable changes under NDG presence, are indicated in Table 2.

### 2.3. Community Composition at the OTU Level Was Affected by NDG Presence and Maintained Stability through Subculturing

Once observed that NDG presence caused communities to be enriched in several taxa, communities were studied at the OTU level from passages 2 to 15. Mixed linear models revealed that NDG was a significant factor for 1070 OTUs, which represents 45.80% of total OTUs (*p*_FDR_ < 0.05). Additionally, NDG presence was a very significant factor yet for 535 OTUs (22.90%, *p*_FDR_ < 0.01) and a highly significant factor for 226 OTUs (9.67%, *p*_FDR_ < 0.001) (Table S3). These results, together with variations observed for the different taxa, support the influence of NDG on communities composition.

Moreover, the study of OTU stability over time was also addressed. Linear models were performed for each OTU and for each culture medium independently, from passages 2 to 15, including the passage number as a variable. All OTUs were stable in MCG-3 medium (*p*_FDR_ > 0.05). Passage number resulted to be a significant factor just for OTU_1370 and OTU_1510 in MCG-3B medium. This time dependence was not highly significant for none of them, though (*p*_FDR_ = 4.38 × 10^−2^). Thus, 99% of OTUs did not statistically vary in their relative abundance through subculturing in NDG presence nor in NDG absence (*p*_FDR_ > 0.05). These results show how community composition remained stable for both conditions and along all the 14 passages analyzed.

### 2.4. Principal Coordinate Analysis Confirmed the Achievement of the Obtention and the Stable Culture of Communities Influenced by NDG Presence

**Figure 4:**
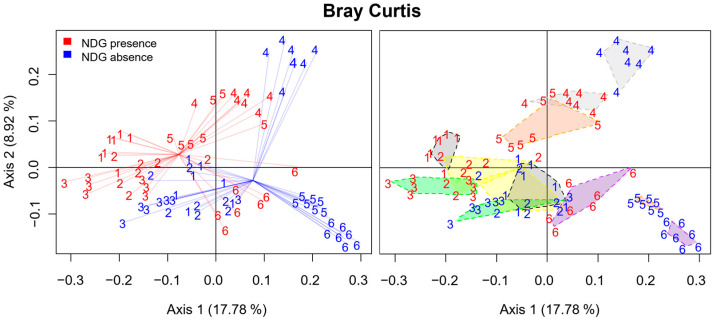
Principal coordinate analysis (PCoA) plots representing dissimilarities among samples from passages 2 to 15 were calculated using the Bray–Curtis classical ecological index. Samples are represented using the number of volunteer. Samples for communities cultured in NDG presence are represented in red, while those cultured in NDG absence are in blue. On the left, samples are linked to the centroid of each group. On the right, samples from the same volunteer and culture media are grouped together, using a different color for each volunteer. For each of the volunteers individually, this representation allows appreciation of how samples from communities cultured with NDG are clearly separated from those of communities cultured without NDG. The percentage of variance explained for each dimension on the PCoA is indicated inside brackets next to the axis number.

Principal Coordinate Analysis (PCoA) based on the Bray–Curtis dissimilarity index was performed for all samples between passages 2 and 15. The first three PCoA components were able to explain only 34.53% of the total variance, and 33 components were necessary to explain at least 80% of the variance. This situation could not be explained by controlled factors of the experiment, neither the culture medium nor the passage number. Then, it was considered that the cause of this variability was differences among the gut microbiota of the individual volunteers. This is an expected limitation when working with human gut microbiota, as each human individual has a unique gut microbiota profile [38]. To investigate this, the effect of the individual volunteers on sample separation was studied. Lineal models including the individual volunteer as a factor were performed for values of samples for the first three components of PCoA. As previously considered, values for the three first components of PCoA were highly dependent on the individual volunteer (*p* < 2.2 × 10^−16^), based on *p* values obtained from ANOVAs performed on the linear models. This result showed an undeniable implication of the volunteer factor in the high variability observed. The aleatory effect associated with the individual volunteer was responsible for samples not grouping perfectly according to the medium in which communities had been cultured. Despite this, when considering the volunteers individually, samples were perfectly grouped according to the culture media (Figure 4). Indeed, values for all axis 1 (*p* = 2.67 × 10^−6^), axis 2 (*p* = 1.87 × 10^−2^) and axis 3 (*p* = 6.19 × 10^−3^) of PCoA were found to be statistically affected by NDG presence or absence in the culture media, as shown by ANOVAs performed after linear models generation (Table S1). Permutational multivariate analysis of variance using distance matrices based on the Bray–Curtis dissimilarity index confirmed NDG presence as a highly significant factor involved in the dissimilarity of samples from passages 2 to 15 (*p* = 1 × 10^−4^), which clearly corroborates the influence of NDG on communities composition. In other words, a high variability due to the personal unique nature of gut microbiota was found to be responsible for major differences among communities. Notwithstanding, a substantial effect of NDG on communities was detected.

Additionally, ANOVAs performed after lineal models generation confirmed passage number was not a significant factor for any of the first three PCoA dimensions (*p* ≥ 0.25), thus supporting the previous results indicating that communities remained with stable composition through subculturing from passages 2 to 15 (Table S1).

## 3. Discussion

Microorganisms in the human body are not isolated, but several kinds of interactions take place in the complex communities conforming to human intestinal microbiota. These multiple nature interactions can positively or negatively influence the survival, growth and/or activities of their neighbors [5,6,9]. Starting from the idea of dietary nutrients acting in the human intestine as an enrichment culture for the intestinal microbiota, two different enrichment culture media containing gluten as the main nitrogen source were used in this study. Additionally, the human gastrointestinal tract is not continuously fed, as foods are introduced at determined intervals during mealtimes. Whereas in healthy individuals, transit through the upper digestive tract is faster, the remaining undigested foods stay in the colon for a median of 21 h [39]. Therefore, in this study, it has been considered that the large intestine could function as a bioreactor holding sequential batch fermentations. Thus, several serial batch cultures were performed to obtain and study complex communities of microorganisms from human microbiota derived from gluten metabolism.

### 3.1. The Initial Diversity Loss Observed for Communities Is Common in Top-Down Approaches

For complex communities, such as those of the human gut, in vitro models better allow the study of dynamics and identification of important microorganisms involved in microbiota functionality, which can be further validated using in vivo models [40,41]. Several works have been based on bottom-up approaches, consisting of the use of previously isolated microorganisms for the construction of new communities [42]. Often, microorganisms from collections previously isolated from several hosts are used, which does not ensure strain compatibility [43]. This fact, together with the incomplete knowledge of the gut ecosystem, may complicate the task of creating solid communities from previously isolated strains [42]. On the other hand, top-down approaches are based on the reduction of the community in the initial sample. They offer more confidence relating to strain compatibility and thus enhance the probability of obtention of robust communities [40,44]. Gut communities of reduced diversity compared to the initial community but still retaining functional characteristics have been obtained using this approach [40]. In this reductionist method transition from in vivo to in vitro environment, this last based on a culture medium with limited substrate diversity and a set of constantly defined environmental conditions, implies an unavoidable loss of diversity [40,41,45,46]. Here, even when the initial community was not analyzed, the initial loss of diversity was still evident between samples of day 0 culture (24 h post-inoculation) and samples of day 2 (72 h post-inoculation).

### 3.2. Communities Early Reached Compositional Stability

Modeling of gut microbiota is usually addressed using simulators, often consisting of continuous or semi-continuous fermentation models. Gut simulators seeded with cecal or fecal samples can give rise to highly diverse and reproducible communities that reach a steady state [40,41,45,47,48]. No consensus over literature exists about how community stability should be measured, nor is it clear how much time is required to achieve stability [40,42,45,47,48]. Here, the compositional stability of communities was addressed through individual study of each of the OTUs and taxa detected. In this respect, communities showed a stable composition from passages 2 to 15. Secondly, β-diversity changes among samples over time were studied via PCoA. No differences were found for community composition from passage 2 on, meaning stable composition was reached after three consecutive batch cultures, i.e., 72 h of culture. This time to reach a compositional steady state is low compared to those found in the literature. McDonald et al. [45] reported that 30 to 36 days were needed for their distal gut chemostat model to reach a steady state. Shorter times of stabilization were reported by Possemiers et al. [48] and Van den Abbeele et al. [41], with 12 days required for human distal gut communities to reach compositional stability in multicompartmental gastrointestinal models. Poeker et al. [40] reported times from 4 to 15 days required to reach a metabolic steady state.

Despite most of the studies have used mainly continuous models or single batch cultures, there is little research performed using serial batch cultures, similar to the herein followed approach. Wang et al. [49] constructed communities of bacteria isolated from poplar rhizosphere through a bottom-up approach based on serial subculturing. Relative stabilization was achieved after five consecutive batch cultures. Few strains ended up dominating the communities, tough [49], which was not observed here. In another study in which fiber-fermenting consortia were obtained from fecal samples through several batch sequential cultures, the dominance of a few OTUs was also observed after some passages [50].

Rapid alterations in the microbiota of even less than one day in response to dietary shifts have been reported for human subjects [51] and humanized mice [52]. This could indicate that distal gut microbiota changes rapidly according to environmental variations, which could have led to an early stabilization of communities. On the other side, the presence of cross-feeding interactions likely occurring within communities may have helped community stabilization. Eminently cooperative communities usually maintain their stability and diversity and are more resistant to abiotic stresses [13,18,53,54]. This kind of interaction is commonly established between *Bacillota*, which is by far the phylum with higher implication in gluten metabolism in the human gut, and both *Actinomycetota* and *Pseudomonadota* [13,34,35].

### 3.3. NDG Promoted Higher Diversity in Communities Derived from Gluten Metabolism

The study of α-diversity brought to light that communities obtained following the herein described method were diverse, as shown by richness and α-diversity indices as Shannon. Similar or even lower Shannon values have been found in some studies not addressing fecal cultures but directly human fecal communities [55,56,57]. Likewise, equivalent numbers [45] or decreased values compared to those reported in the present research [40] have been found for studies in which both more complex models and culture media, better mimicking the large intestinal environment, have been used. These results show that despite being designed as an enrichment medium, MCG-3 succeeded in harboring a notable microbial diversity. Additionally, this means that the obtention and culture of diverse communities derived from gluten metabolism was achieved.

Culture medium containing NDG harbored communities with higher α-diversity than the medium containing only gluten peptone. This could probably be due to NDG directly promoting the proliferation of proteolytic microorganisms [58,59] and/or indirectly facilitating the thriving of other microorganisms, for instance, via syntrophic interactions. Protein intake has previously been linked to higher gut microbial diversity [60].

### 3.4. Gluten in its Non-Digested Form Affected the Communities at All Taxonomic Levels

Substantial changes were found at all taxonomic levels between the communities obtained in media containing or lacking NDG. Both media contained gluten peptone, but only MCG-3 contained NDG. Then, communities cultured in MCG-3 must be enriched in microorganisms able to degrade intact gluten protein. These communities could also be enriched in microorganisms benefitting from the former’s metabolism.

Communities cultured with NDG were significantly enriched in *Bacillota*, which has been largely identified as the main taxon responsible for gluten metabolism [34,35,61]. Caminero et al. identified lactobacilli, *Streptococcus*, *Staphylococcus* and *Clostridium* as important groups related to gluten metabolism [34]. Within this study, *Clostridium sensu stricto* and *Streptococcus*, together with the *Clostridium* XIVa cluster, were significantly enriched in those communities cultured with NDG. Also, *Erysipelotrichaceae* and *Lachnospiraceae* were enriched in communities under NDG presence. Both families have been identified along with *Clostridiaceae* as contributors to protein catabolism in the gut [62]. Furthermore, all genera from the *Peptoniphilaceae* family were enriched under NDG presence. This group of microorganisms is not known to be involved in gluten proteolysis in the human gut but in amino acid fermentation [63,64,65]. Then, it is possible that these microorganisms established cross-feeding interactions with other NDG degraders within the community.

*Actinomycetota*, and especially *Bifidobacterium*, have also been reported as relevant microorganisms regarding gluten and derived peptides metabolism in the human gut [34,35,36,66]. Here, *Actinomycetota*, as well as *Bifidobacterium*, were significantly enriched in communities under NDG presence.

*Bacteroidota* showed notable relative abundance in all communities. Increases in abundance from passage 0 to 2 were detected for this phylum, suggesting its ability to metabolize gluten. Interestingly, *Bacteroidota* were not enriched in NDG presence or absence. However, 70% of OTUs assigned to this phylum did show changes in its relative abundance associated with NDG presence. Then, some members of *Bacteroidota* preferred predigested gluten, while others were enriched under NDG presence. A high abundance of *Bacteroides* in MCG-3 fecal cultures has been reported [34,36]. Also, several *Bacteroidota* strains have shown a gliadin-hydrolyzing ability [67].

NDG presence affected a wide range of diverse taxonomic groups. Thus, it could be declared that NDG presence noticeably drove communities composition. Observed effects become clearer when considering gluten in its not digested form is not directly available for microorganisms but it must be made available. This shows how some microorganisms within communities exhibited a marked preference for this form of gluten as a substrate. Specifically, it should be highlighted the enrichment in *Bacillota* and *Actinomycetota* under NDG presence, which comprises taxa showing essential functions for gut function and homeostasis maintenance [68,69]. Moreover, *Pseudomonadota*, a phylum that has been widely linked to disease susceptibility, was notably decreased under NDG presence [70].

### 3.5. Gluten in Its Non-Digested Form Affected Communities at the OTU Level

Once changes associated with NDG presence had been observed at all taxonomic levels, a global analysis at the OTU level was carried out. Almost half of the detected OTUs underwent significant changes associated with NDG presence. This is a high proportion of microorganisms, considering that the only difference between the two culture media is the addition of gluten in its undigested form (NDG). Moreover, NDG influence in community composition was confirmed via PCoA. This corroborates what was seen in the taxonomic analysis. Despite most microorganisms could solely grow with previously digested gluten as the main nitrogen source, a high number of microorganisms had a direct or indirect preference for NDG.

### 3.6. Communities Were Highly Variable among Volunteers, though Common Patterns Were Detected

The herein presented study is not exempt from limitations, though, with one of them being the high interindividual variability. This variability was due to human individuals naturally presenting unique microbiota profiles. It was the cause of the low variance explained by the first components of PCoA and sample clustering not being perfect. This is not something strange when studying gut microbiota or, what is more, it may be an expected inconvenience. Other authors studying fecal communities have undergone similar volunteer variability effects giving place to low variance explained in this kind of analysis [44,46]. Despite this, samples from each of the volunteers did clearly group according to the culture media, which reinforces the influence of NDG presence on the composition of communities derived from gluten metabolism.

On the other hand, despite this high impact variability and the heterogeneity of the volunteers involved in the study, this method still enabled the identification of clear changes related to NDG metabolism in relevant gut taxa. These changes were significant and consistent among volunteers.

### 3.7. Potential Translational Applications of In Vitro Study of Gut Microbial Communities

In this study, serial passage culturing in enrichment media enabled the obtention, culture and study of gut microbial communities. Exposing communities to different stimuli or disturbances in vitro and evaluating their responses might help to understand the potential effects of such alterations in gut microbiota and, consequently, in host health.

Moreover, microbiota modulation through diet has gained increased interest in the last few years. However, responsiveness to dietary interventions highly varies among individuals [71]. Therefore, herein presented methodology could be applied to assess, in an individualized manner, how gluten or other food components may affect intestinal communities. Consequently, this could serve as a tool to contribute to the design of personalized dietary interventions.

Finally, by performing adequate optimizations of the culture media, communities with specific composition or enriched/depleted in certain microbial groups might be obtained from the fecal microbiota of an individual. Then, selected populations could have an application as an “autologous fecal microbiota transplant”. This may contribute to the effectiveness of microbiota transplants by reducing the emergence of incompatibilities observed in heterologous microbiota transplants, which are caused by interactions between the donor and the recipient microbiota. Compared to heterologous microbiota transplants, autologous transplants also avoid some safety concerns regarding the potential transference of pathogens from the donor to the recipient [72].

## 4. Materials and Methods

### 4.1. Subjects of Study and Fecal Sampling

Fecal samples from six healthy adults, three men and three women (aged 25–54, average 34.8 years), on a normal diet, were used to inoculate different cultures with the aim of studying fecal communities derived from gluten metabolism. None of the volunteers had received antibiotic treatment for at least one month prior to the sampling date. Fresh stools were kept under anoxic conditions using the Anaerogen system (Oxoid, Basingstoke, UK) and processed within a maximum of one hour. This study was performed in accordance with the Declaration of Helsinki. Previous informed written consent was obtained from all volunteers. All procedures were approved by the Ethics Committee from the University of León (ETICA-ULE-036-2021).

### 4.2. Culture Media

Two different culture media, MCG-3 and MCG-3B, were used for the obtention and maintenance of fecal communities derived from gluten metabolism [34]. Both culture media contained gluten as the main nitrogen source, but gluten was included in different forms in each one. MCG-3 medium contained non-digested gluten (NDG) and digested gluten (gluten peptone) as the main nitrogen source, while MCG-3B medium contained only gluten peptone. Both culture media also contained glucose (1 g/L), sodium pyruvate (1 g/L), sodium succinate (0.5 g/L), NaHCO_3_ (0.4 g/L), NaCl (5 g/L), CaCl_2_ (0.05 g/L), Ca(OH)_2_ (0.15 g/L), ZnSO_4_ (0.07 g/L), L-cysteine (0.5 g/L), L-arginine (1 g/L), meat extract (1 g/L), meat peptone (1 g/L), sodium pyrophosphate (0.25 g/L), hemin (0.01 g/L), K1 vitamin (0.0001% (*v/v*)), biotin (0.001 g/L), riboflavin (0.001 g/L) and thiamine (0.001 g/L). Gluten peptone was used at 5 g/L and, for simulation of a high gluten diet, NDG was used at 20 g/L. Both media were adjusted to pH 7 and buffered with 1% KH_2_PO_4_/K_2_HPO_4_.

### 4.3. Obtention and Maintenance of Microbial Communities Derived from Gluten Metabolism

For obtention of communities derived from gluten metabolism, fecal samples were homogenized 1:5 (p/v) in sterile NaCl (0.9%) and L-cysteine (0.5 g/L) solution. Then, 1 mL of the homogenate was used to inoculate 50 mL cultures of either MCG-3 or MCG-3B media. Several passages were performed for each culture to obtain complex microbial communities derived from gluten metabolism and study their dynamics. Briefly, after 24 h incubation, each culture was used for inoculation (2% *v/v*) of 50 mL of the same fresh medium in which the culture was originally performed. A total of 15 passages were carried out. All cultures were incubated at 37 °C in anoxic conditions (Anaerogen, Oxoid, Basingstoke, UK). Daily measurement of culture pH was carried out to ensure the maintenance of physiological conditions according to those in the healthy human large colon [73].

### 4.4. Microbial Community Composition Analysis

To study microbial community composition, cultures from passages 0, 2, 4, 6, 8, 10, 12 and 15 were analyzed. For this purpose, microbial genomic DNA was extracted from 4 mL culture aliquots collected after 24 h incubation using DNeasy PowerSoil kit (Qiagen, Hilden, Germany). NanoDrop™ 2000 spectrophotometer (Thermo Fisher Scientific, Wilmington, DE, USA), Qubit^®^ 2.0 fluorometer (Thermo Fisher Scientific, Wilmington, DE, USA) and agarose gel electrophoresis were used to determine DNA concentration, quality and integrity. PCR amplification of the V3−V4 region from 16S rDNA was carried out using 341F/806R primers. Barcoded libraries for the 96 samples were pair-end sequenced (250 pb × 2) by Illumina technology. Raw reads were processed using vsearch-2.8.4 [74]. First, primer sequences were trimmed, and paired-end reads were merged. Then, quality filtering was carried out. Sequences with low quality or not corresponding to the target region were discarded. Reads were clustered in Operational Taxonomic Units (OTUs) at 97% identity with vsearch. OTUs with a frequency higher than 10 in the total of samples were selected. Taxonomic assignment of OTUs was obtained using the CLASSIFIER tool [75] from the RDP database [76]. Sequences of the determinate OTUs were aligned using Infernal [77]. An approximately maximum likelihood phylogenetic tree using the GTR evolutionary model was constructed with FastTree [78] and edited with MEGA7 [79]. The Vegan 2.5 R package [80] was used for α- and β-diversity analysis based on the phylogenetical tree and the OTU frequency chart. The phylogenetic Net Relatedness Index (NRI) was estimated using the Picante 1.8.2 R package [81]. The dissimilarity matrix was obtained through the classic ecological Bray–Curtis index calculation for each pair of samples. Then, Principal Coordinate Analysis (PCoA) was carried out using the ape v5.4 R package [82]. Permutation tests of multivariate analysis of variance were performed with the Vegan package, using dissimilarity matrices to identify statistical differences between communities due to culture conditions.

To perform further statistical analysis reads were normalized to 100,000 per sample. Multiple linear and linear mixed models were generated using the lmerTest R package [83]. Extraordinarily low abundant OTUs showing no reads from passages 0 to 15 in MCG-3 or in MCG-3B were not considered in the study, as fitting the linear mixed models was not possible. Linear models including passage number as a variable were performed for communities in MCG-3 and MCG-3B media individually for samples corresponding to passages 2 to 15. These models revealed that passage number was not significant for OTUs nor for taxa in MCG-3 and MCG-3B. Thus, linear mixed models comprising NDG presence as a fixed factor and the volunteer as a random factor were used for further evaluation of communities between passages 2 and 15. Due to the intrinsic interindividual variability of the human microbiota, relative abundances for some taxa/OTUs showed varying ranges of values in communities corresponding to the different volunteers. Evaluation of the effect of NDG was carried out through mixed effects models, considering different intercepts for each volunteer in the model equation. Thus, the inclusion of the volunteer as a random effect in the models enabled the detection of changes that were consistent throughout the communities studied. All models performed and data extracted from them are included in the Supplementary Material (Tables S1–S3). A False Discovery Rate (FDR) correction for pairwise comparison using the Benjamini and Hochberg method [84] was applied to assess the significance of passage number and NDG presence for taxa and OTUs. All statistical analyses were performed using the R programming language [85].

## 5. Conclusions

Altogether, our results suggested an influence of NDG presence on the fecal community composition, not only at the different taxonomic levels but also at the OTU level, showing potential changes in gut microbiota associated with undigested gluten presence. Furthermore, not only diverse communities derived from gluten metabolism were obtained, but also its maintenance over time with a stable composition for a long period was achieved.

## Figures and Tables

**Table 1:** Statistics and data extracted from models performed for α-diversity indices calculated for communities cultured in MCG-3 or MCG-3B from days 2 to 15. The Table shows the mean ± standard deviation for the indices in each kind of community. Lineal models were generated for evaluation of the differences between both culture media. For such models, the mean sum of squares between groups (MSB), F statistic (F) and significance levels based on *p*-values obtained from ANOVA tests are indicated.

			Linear Model (y~NDG ^1^ Presence) Degrees of Freedom: 1, 82		
	Digested Gluten + NDG ^1^ (MCG-3)	Digested Gluten (MCG-3B)	MSB	F	Significance (*p*) ^2^
Shannon diversity (H’)	3.26 ± 0.30	2.65 ± 0.25	7.89	102.93	3.86 × 10^−16^ ***
Simpson dominance (D)	0.15 ± 0.04	0.25 ± 0.05	0.19	81.46	6.41 × 10^−14^ ***
NRI ^3^	−5.60 ± 1.87	−7.40 ± 1.81	67.75	20.07	2.40 × 10^−5^ ***

^1^ NDG: Non-Digested Gluten ^2^ Significance: *** (*p* < 0.001). ^3^ NRI: Net Relatedness Index.

**Table 2:** Relative abundance from passage 2 to 15 for some families and genera detected in communities obtained in NDG presence (MCG-3 medium) or absence (MCG-3B medium). The percentage of reads for each group within the corresponding taxonomic level is shown in each condition. Log Fold Change (LFC) was calculated as the base-2 logarithm of the ratio between relative abundance in NDG presence and relative abundance in NDG absence for each taxon. The significance of the differences found between both conditions was assessed through FDR-corrected *p*-values obtained from ANOVA tests performed after multiple linear mixed model generation (Table S2).

Taxon	Relative Abundance (% Reads) in MCG-3	Relative Abundance (% Reads) in MCG-3B	LFC	Significance (*p*_FDR_) ^1^
*Enterobacteriaceae*	38.17	48.98	−0.36	8.19 × 10^−10^ ***
*Lachnospiraceae*	13.47	10.90	0.31	6.19 × 10^−4^ ***
*Clostridiaceae 1*	8.42	3.28	1.36	6.70 × 10^−7^ ***
*Streptococcaceae*	1.26	0.09	3.88	4.87 × 10^−5^ ***
*Bifidobacteriaceae*	1.06	0.27	2.00	1.43 × 10^−3^ **
*Peptoniphilaceae*	0.75	0.24	1.64	2.02 × 10^−3^ **
*Prevotellaceae*	0.56	0.08	2.84	3.98 × 10^−3^ **
*Porphyromonadaceae*	0.50	0.10	2.37	3.58 × 10^−17^ ***
*Sutterellaceae*	0.39	0.08	2.27	4.17 × 10^−4^ ***
*Erysipelotrichaceae*	0.30	0.10	1.65	3.41 × 10^−2^ *
*Eggerthellaceae*	0.12	0.05	1.34	8.77 × 10^−3^ **
*Odoribacteraceae*	3.81 × 10^−3^	4.78 × 10^−4^	3.00	1.74 × 10^−5^ ***
*Selenomonadaceae*	1.52 × 10^−2^	4.74 × 10^−3^	1.68	6.63 × 10^−3^ **
*Escherichia/Shigella*	40.69	51.12	−0.33	8.19 × 10^−10^ ***
*Bacteroides*	18.84	19.30	−0.03	5.18 × 10^−1^
*Clostridium sensu stricto*	8.97	3.38	1.41	7.74 × 10^−7^ ***
*Enterococcus*	4.94	5.54	−0.17	2.82 × 10^−1^
*Veillonella*	4.69	5.55	−0.24	2.54 × 10^−1^
*Roseburia*	2.81	1.26	1.16	6.56 × 10^−3^ **
*Enterocloster*	2.31	3.34	−0.53	8.31 × 10^−8^ ***
*Phocaeicola*	2.27	1.03	1.14	7.88 × 10^−4^ ***
*Paraclostridium*	1.57	2.05	−0.38	9.24 × 10^−2^
*Streptococcus*	1.41	0.09	3.92	4.87 × 10^−5^ ***
*Collinsella*	1.21	0.63	0.96	2.88 × 10^−1^
*Bifidobacterium*	1.02	0.25	2.04	1.43 × 10^−3^ **
*Peptoniphilus*	0.77	0.25	1.62	6.22 × 10^−3^ **
*Parabacteroides*	0.56	0.10	2.52	5.67 × 10^−18^ ***
*Clostridium* XIVa	0.29	0.18	0.70	9.68 × 10^−3^ **
*Mediterraneibacter*	2.99 × 10^−2^	7.14 × 10^−3^	2.06	8.77 × 10^−3^ **
*Intestinimonas*	2.38 × 10^−2^	6.44 × 10^−3^	1.88	6.19 × 10^−4^ ***
*Intestinibacillus*	8.20 × 10^−3^	2.23 × 10^−3^	1.88	6.12 × 10^−5^ ***
*Butyricimonas*	1.35 × 10^−3^	1.56 × 10^−4^	3.11	9.17 × 10^−3^ **

^1^ Significance: * (*p* < 0.05); ** (*p* < 0.01); *** (*p* < 0.001).

## Data Availability

Data will be made available by the authors on request.

## Supplementary Materials

The following supporting information can be downloaded at https://www.mdpi.com/article/10.3390/ijms25074013/s1.
