# Supplementary material for: Modeling Dynamics of Human Gut Microbiota Derived from Gluten Metabolism: Obtention, Maintenance and Characterization of Complex Microbial Communities
Source: Int J Mol Sci. 2024 Apr 4;25(7):4013. doi: 10.3390/ijms25074013 (PMC11012253; doi:10.3390/ijms25074013)
Supplement: Supplementary file 1 [file ijms-25-04013-s001.zip › Supplementary.pdf]

Table S1. Data extracted from linear models performed for diversity analysis.

	Shannon: lm(passage 0 Vs passages 2-15)		
	Coefficient passage 0	Coefficient passages 2-15	*p* -ANOVA
MCG-3	3.69	3.26	4.14E-03
MCG-3B	3.67	2.64	1.07E-10

PCoA	lm (y~NDG presence)			lm (y~passage number)	lm (y~volunteer)
Bray-Curtis	*p* -ANOVA	Coefficient NDG presence	Coefficient NDG absence	*p* -ANOVA	*p* -ANOVA
Axis 1	2.68E-06	-0.08	0.08	2.52E-01	1.70E-16
Axis 2	1.87E-02	0.03	-0.03	6.93E-01	6.31E-22
Axis 3	6.19E-03	-0.03	0.03	6.15E-01	5.44E-17

Table S2. Data extracted from linear models performed for assessing the effect of passage number and NDG presence for taxa. Table shows *p* FDR values obtained from multiple linear and linear mixed models performed for taxa from passage 2 to passage 15. "Model 1" was used for assessing significance of passage number and "Model 2" was used for studying significance for NDG presence, by performing ANOVAs over the models generated. A false discovery rate (FDR) correction for multiple comparisons was applied to *p* values obtained. Coefficients for NDG presence (MCG-3) and NDG absence (MCG-3B) obtained from "Model 2" allowed to know whether each taxa levels were higher or lower in NDG presence compared to NDG absence. Percentage of average reads for each taxa in NDG presence and in NDG absence, from passages 2 to 15, is also indicated. Log Fold CHange (LFC) was calculated as the base 2 logarithm of the ratio between the average relative abundance in NDG presence and absence.

	Model 1 - lm(y~passage number)		Model 2 - lmer(y~NDG presence+(1|volunteer))					
Taxa	*p* FDR passage number for MCG-3	*p* FDR passage number for MCG-3B	*p* FDR	Coeficient Model 2 - NDG presence	Coeficient Model 2 - NDG absence	Average percentage of reads in MCG-3 for passages 2-15	Average percentage of reads in MCG-3D for passages 2-15	LFC
P_Firmicutes	2.03E-01	3.94E-01	4.75E-05	37764.07	30381.67	3.82E+01	3.07E+01	3.14E-01
C_Clostridia	3.32E-02	5.22E-01	8.51E-08	25041.41	17222.31	2.55E+01	1.75E+01	5.41E-01
O_Clostridiales	3.32E-02	5.22E-01	8.51E-08	25041.41	17222.31	2.55E+01	1.76E+01	5.39E-01
F_Lachnospiraceae	7.37E-01	8.19E-01	6.19E-04	13113.80	10643.19	1.35E+01	1.09E+01	3.06E-01
G_Enterocloster	5.03E-01	6.26E-01	8.31E-08	2010.52	3010.57	2.31E+00	3.34E+00	-5.31E-01
G_Roseburia	8.47E-01	8.19E-01	6.56E-03	2446.70	1136.25	2.81E+00	1.26E+00	1.16E+00
G_Hungatella	8.43E-01	5.22E-01	8.93E-01	773.14	782.08	8.89E-01	8.68E-01	3.44E-02
G_Dorea	5.71E-01	6.89E-01	3.24E-02	386.80	239.29	4.45E-01	2.65E-01	7.44E-01
G_Clostridium_XlVa	5.70E-01	5.22E-01	9.68E-03	251.59	160.77	2.89E-01	1.78E-01	6.97E-01
G_Blautia	7.35E-01	8.89E-01	2.76E-02	149.77	110.51	1.72E-01	1.23E-01	4.90E-01
G_Eisenbergiella	8.83E-01	5.99E-01	2.00E-04	82.70	130.25	9.50E-02	1.45E-01	-6.04E-01
G_Anaerobutyricum	7.81E-01	8.33E-01	6.03E-01	28.03	36.73	3.22E-02	4.07E-02	-3.39E-01
G_Anaerotignum	9.17E-01	5.48E-01	3.88E-07	84.92	8.28	9.76E-02	9.18E-03	3.41E+00
G_Anaerostipes	9.83E-01	8.19E-01	1.20E-01	16.68	10.25	1.92E-02	1.14E-02	7.53E-01
G_Bariatricus	9.73E-01	6.33E-01	8.72E-01	33.84	33.01	3.89E-02	3.66E-02	8.68E-02
G_Sellimonas	9.17E-01	6.33E-01	7.21E-02	17.25	32.65	1.98E-02	3.62E-02	-8.70E-01
G_Fusicatenibacter	7.37E-01	8.19E-01	6.32E-02	13.10	8.45	1.51E-02	9.38E-03	6.84E-01
G_Mediterraneibacter	4.38E-01	8.19E-01	8.77E-03	25.98	6.44	2.99E-02	7.14E-03	2.06E+00
G_Agathobacter	5.47E-01	8.52E-01	6.32E-02	12.63	5.68	1.45E-02	6.30E-03	1.20E+00
G_Coprococcus	8.41E-01	6.33E-01	5.50E-01	3.22	2.56	3.70E-03	2.84E-03	3.81E-01
G_Lachnospira	8.83E-01	8.19E-01	3.63E-01	2.32	1.59	2.67E-03	1.76E-03	5.98E-01
G_Schaedlerella	9.17E-01	5.22E-01	6.02E-02	7.83	0.77	9.00E-03	8.51E-04	3.40E+00
G_Kineothrix	7.82E-01	5.22E-01	4.96E-02	3.76	0.51	4.32E-03	5.63E-04	2.94E+00
G_Lactonifactor	4.56E-01	9.68E-01	5.53E-03	2.53	0.23	2.90E-03	2.54E-04	3.52E+00
G_Ruminococcus2	9.00E-01	6.00E-01	8.68E-02	0.86	0.28	9.93E-04	3.06E-04	1.70E+00
F_Clostridiaceae_1	3.58E-02	5.22E-01	6.70E-07	8193.19	3201.62	8.42E+00	3.28E+00	1.36E+00
G_Clostridium_sensu_stricto	3.48E-02	5.22E-01	7.74E-07	7801.64	3044.11	8.97E+00	3.38E+00	1.41E+00
F_Peptostreptococcaceae	9.00E-01	5.22E-01	1.34E-01	1448.66	1884.69	1.49E+00	1.93E+00	-3.75E-01
G_Paraclostridium	9.00E-01	5.22E-01	9.24E-02	1365.96	1846.85	1.57E+00	2.05E+00	-3.84E-01
G_Paeniclostridium	9.17E-01	6.33E-01	6.72E-01	18.62	16.50	2.14E-02	1.83E-02	2.25E-01
G_Romboutsia	9.57E-01	5.48E-01	5.37E-02	59.34	14.35	6.82E-02	1.59E-02	2.10E+00
F_Ruminococcaceae	9.56E-01	7.36E-01	6.99E-01	1062.26	1003.40	1.09E+00	1.03E+00	8.71E-02
G_Faecalibacterium	8.93E-01	7.25E-01	6.32E-02	246.31	123.48	2.83E-01	1.37E-01	1.05E+00
G_Flavonifractor	7.59E-01	5.48E-01	3.94E-01	211.85	229.96	2.43E-01	2.55E-01	-6.73E-02
G_Flintibacter	4.38E-01	8.19E-01	4.11E-01	72.09	99.11	8.29E-02	1.10E-01	-4.08E-01
G_Gemmiger	9.00E-01	8.19E-01	6.83E-01	29.55	26.02	3.40E-02	2.89E-02	2.34E-01
G_Lawsonibacter	4.76E-01	5.58E-01	8.42E-01	9.81	10.57	1.13E-02	1.17E-02	-5.62E-02
G_Dysosmobacter	4.38E-01	5.22E-01	1.18E-01	13.77	24.30	1.58E-02	2.70E-02	-7.68E-01
G_Intestinimonas	9.96E-01	5.22E-01	6.19E-04	20.70	5.81	2.38E-02	6.44E-03	1.88E+00
G_Ruthenibacterium	9.00E-01	5.22E-01	5.37E-01	4.99	4.26	5.74E-03	4.72E-03	2.81E-01
G_Oscillibacter	8.13E-01	6.33E-01	3.51E-03	6.04	1.08	6.94E-03	1.20E-03	2.54E+00
G_Ruminococcus	7.82E-01	8.52E-01	3.41E-02	4.27	0.97	4.91E-03	1.07E-03	2.19E+00
G_Pseudoflavonifractor	6.33E-01	5.22E-01	9.50E-01	1.70	1.67	1.96E-03	1.85E-03	8.16E-02
F_Peptoniphilaceae	2.30E-01	5.22E-01	2.02E-03	731.54	235.61	7.51E-01	2.41E-01	1.64E+00
G_Peptoniphilus	2.29E-01	5.22E-01	6.22E-03	673.50	227.68	7.74E-01	2.53E-01	1.62E+00
G_Anaerococcus	7.82E-01	5.22E-01	4.65E-02	43.80	5.05	5.03E-02	5.61E-03	3.17E+00
G_Finegoldia	9.00E-01	6.33E-01	3.61E-02	13.25	2.43	1.52E-02	2.70E-03	2.50E+00
F_Eubacteriaceae	3.65E-02	6.54E-01	1.72E-02	44.64	92.66	4.58E-02	9.49E-02	-1.05E+00
G_Intestinibacillus	9.00E-01	8.33E-01	6.12E-05	7.13	2.01	8.20E-03	2.23E-03	1.88E+00
G_Pseudoramibacter	2.77E-01	5.22E-01	1.75E-01	1.47	2.90	1.69E-03	3.22E-03	-9.34E-01
G_Eubacterium	9.00E-01	5.22E-01	1.62E-01	0.35	1.70	4.02E-04	1.88E-03	-2.23E+00
C_Bacilli	9.83E-01	5.48E-01	3.94E-01	6359.85	5768.79	6.46E+00	5.86E+00	1.42E-01
O_Lactobacillales	9.83E-01	5.58E-01	3.59E-01	6300.22	5656.03	6.42E+00	5.77E+00	1.55E-01
F_Enterococcaceae	9.02E-01	5.48E-01	2.41E-01	4492.69	5259.10	4.61E+00	5.38E+00	-2.22E-01
G_Enterococcus	9.00E-01	5.55E-01	2.82E-01	4298.12	4994.33	4.94E+00	5.54E+00	-1.66E-01
F_Streptococcaceae	7.82E-01	7.66E-01	4.87E-05	1228.51	83.89	1.26E+00	8.59E-02	3.88E+00
G_Streptococcus	7.82E-01	7.66E-01	4.87E-05	1228.51	83.89	1.41E+00	9.31E-02	3.92E+00
F_Lactobacillaceae	8.43E-01	5.22E-01	1.89E-01	325.60	143.67	3.34E-01	1.47E-01	1.19E+00
G_Limosilactobacillus	5.47E-01	5.58E-01	5.35E-01	160.98	89.85	1.85E-01	9.97E-02	8.92E-01
G_Ligilactobacillus	9.56E-01	9.56E-01	3.46E-01	41.95	25.27	4.82E-02	2.80E-02	7.83E-01
G_Lactobacillus	6.97E-01	5.22E-01	7.54E-02	118.88	27.78	1.37E-01	3.08E-02	2.15E+00
F_Carnobacteriaceae	4.38E-01	8.19E-01	3.84E-01	0.90	3.86	9.29E-04	3.95E-03	-2.09E+00
G_Granulicatella	4.38E-01	8.19E-01	3.84E-01	0.90	3.86	1.04E-03	4.28E-03	-2.04E+00
C_Negativicutes	9.00E-01	5.22E-01	1.87E-01	5166.48	6227.11	5.25E+00	6.32E+00	-2.68E-01
O_Veillonellales	9.00E-01	5.48E-01	2.41E-01	4336.45	5271.20	4.42E+00	5.37E+00	-2.82E-01
F_Veillonellaceae	9.00E-01	5.48E-01	2.41E-01	4336.45	5271.20	4.45E+00	5.40E+00	-2.77E-01
G_Veillonella	9.17E-01	5.22E-01	2.54E-01	4078.76	4999.77	4.69E+00	5.55E+00	-2.43E-01
G_Dialister	4.38E-01	5.22E-01	6.03E-01	158.78	231.05	1.82E-01	2.56E-01	-4.90E-01
G_Megasphaera	9.08E-01	8.19E-01	4.98E-02	25.86	8.89	2.97E-02	9.87E-03	1.59E+00
G_Allisonella	9.00E-01	7.27E-01	8.61E-02	29.31	13.34	3.37E-02	1.48E-02	1.19E+00
G_Negativicoccus	2.29E-01	6.33E-01	1.92E-01	34.25	12.27	3.94E-02	1.36E-02	1.53E+00
O_Selenomonadales	5.00E-01	5.22E-01	6.63E-03	14.82	4.63	1.51E-02	4.72E-03	1.68E+00
F_Selenomonadaceae	5.00E-01	5.22E-01	6.63E-03	14.82	4.63	1.52E-02	4.74E-03	1.68E+00
G_Megamonas	8.47E-01	5.22E-01	2.27E-02	9.69	3.25	1.11E-02	3.60E-03	1.63E+00
G_Mitsuokella	5.84E-01	8.19E-01	1.97E-01	5.14	1.38	5.91E-03	1.53E-03	1.95E+00
O_Acidaminococcales	9.00E-01	8.19E-01	6.01E-01	725.82	816.58	7.40E-01	8.33E-01	-1.71E-01
F_Acidaminococcaceae	9.00E-01	8.19E-01	6.01E-01	725.82	816.58	7.46E-01	8.36E-01	-1.65E-01
G_Phascolarctobacterium	4.38E-01	5.48E-01	2.08E-03	289.18	585.66	3.32E-01	6.50E-01	-9.67E-01
G_Acidaminococcus	7.96E-01	5.22E-01	1.97E-01	434.35	227.67	4.99E-01	2.53E-01	9.83E-01
C_Erysipelotrichia	9.00E-01	5.22E-01	9.50E-01	767.35	754.98	7.80E-01	7.67E-01	2.45E-02
O_Erysipelotrichales	9.00E-01	5.22E-01	9.50E-01	767.35	754.98	7.82E-01	7.70E-01	2.27E-02
F_Erysipelatoclostridiaceae	8.83E-01	5.22E-01	3.94E-01	476.00	661.90	4.89E-01	6.78E-01	-4.71E-01
G_Erysipelatoclostridium	8.83E-01	5.22E-01	3.94E-01	476.00	661.90	5.47E-01	7.34E-01	-4.25E-01
F_Erysipelotrichaceae	9.46E-01	5.22E-01	3.41E-02	291.33	93.07	2.99E-01	9.53E-02	1.65E+00
G_Catenibacterium	8.83E-01	8.95E-01	8.50E-01	31.62	34.86	3.63E-02	3.87E-02	-8.98E-02
G_Turicibacter	9.55E-01	5.22E-01	7.21E-02	134.15	26.78	1.54E-01	2.97E-02	2.38E+00
G_Holdemanella	9.00E-01	5.22E-01	5.75E-03	6.79	1.64	7.80E-03	1.82E-03	2.10E+00

G_Faecalibaculum	9.00E-01	5.22E-01	1.57E-01	26.47	7.36	3.04E-02	8.16E-03	1.90E+00
G_Solobacterium	4.56E-01	8.87E-01	4.17E-02	9.12	1.15	1.05E-02	1.28E-03	3.04E+00
G_Allobaculum	9.56E-01	5.22E-01	4.27E-02	18.21	1.41	2.09E-02	1.57E-03	3.74E+00
G_Faecalibacillus	7.82E-01	9.68E-01	1.55E-01	3.78	0.26	4.35E-03	2.93E-04	3.89E+00
G_Holdemania	4.56E-01	8.45E-01	8.72E-01	2.58	2.72	2.96E-03	3.02E-03	-2.55E-02
G_Dielma	8.13E-01	5.22E-01	4.84E-03	1.93	0.14	2.22E-03	1.51E-04	3.88E+00
P_Proteobacteria	2.29E-01	5.22E-01	2.37E-09	38083.11	48529.55	3.85E+01	4.91E+01	-3.50E-01
C_Gammaproteobacteria	2.29E-01	5.22E-01	1.20E-09	37544.54	48163.04	3.82E+01	4.89E+01	-3.58E-01
O_Enterobacterales	2.29E-01	5.22E-01	8.19E-10	37188.24	47906.09	3.79E+01	4.88E+01	-3.66E-01
F_Enterobacteriaceae	2.29E-01	5.22E-01	8.19E-10	37162.10	47841.09	3.82E+01	4.90E+01	-3.60E-01
G_Escherichia/Shigella	2.29E-01	5.22E-01	8.19E-10	35398.98	46074.65	4.07E+01	5.11E+01	-3.29E-01
G_Pseudescherichia	9.90E-01	9.14E-01	8.72E-01	725.24	718.93	8.34E-01	7.98E-01	6.36E-02
G_Enterobacter	4.38E-01	5.74E-01	5.01E-01	48.83	25.38	5.61E-02	2.82E-02	9.95E-01
F_Morganellaceae	9.00E-01	5.22E-01	5.97E-02	4.22	19.85	4.34E-03	2.03E-02	-2.23E+00
G_Morganella	9.00E-01	5.22E-01	5.97E-02	4.22	19.85	4.85E-03	2.20E-02	-2.18E+00
F_Yersiniaceae	5.71E-01	8.87E-01	6.60E-01	0.45	0.66	4.65E-04	6.75E-04	-5.39E-01
G_Serratia	5.71E-01	8.87E-01	6.60E-01	0.45	0.66	5.20E-04	7.32E-04	-4.93E-01
O_Aeromonadales	8.83E-01	6.54E-01	9.30E-02	224.74	75.96	2.29E-01	7.75E-02	1.56E+00
F_Succinivibrionaceae	8.83E-01	6.54E-01	9.30E-02	224.74	75.96	2.31E-01	7.78E-02	1.57E+00
G_Succinivibrio	8.83E-01	6.54E-01	9.30E-02	224.74	75.96	2.58E-01	8.43E-02	1.62E+00
O_Pasteurellales	6.37E-01	7.25E-01	3.94E-01	12.69	29.79	1.29E-02	3.04E-02	-1.23E+00
F_Pasteurellaceae	6.37E-01	7.25E-01	3.94E-01	12.69	29.79	1.30E-02	3.05E-02	-1.23E+00
G_Haemophilus	5.82E-01	7.27E-01	3.94E-01	9.30	27.17	1.07E-02	3.01E-02	-1.50E+00
G_Conservatibacter	9.00E-01	8.17E-01	3.94E-01	0.18	0.32	2.03E-04	3.57E-04	-8.16E-01
O_Nevskiales	6.97E-01	6.33E-01	2.14E-01	0.03	9.16	2.67E-05	9.34E-03	-8.45E+00
F_Nevskiaceae	6.97E-01	6.33E-01	2.14E-01	0.03	9.16	2.69E-05	9.38E-03	-8.45E+00
G_Nevskia	6.97E-01	6.33E-01	2.14E-01	0.03	9.16	3.01E-05	1.02E-02	-8.40E+00
O_Pseudomonadales	NA	5.22E-01	1.75E-01	0.00	0.91	0.00E+00	9.32E-04	#¡NUM!
F_Pseudomonadaceae	NA	5.22E-01	1.75E-01	0.00	0.91	0.00E+00	9.36E-04	#¡NUM!
G_Pseudomonas	NA	5.22E-01	1.75E-01	0.00	0.91	0.00E+00	1.01E-03	#¡NUM!
O_Xanthomonadales	NA	5.62E-01	1.20E-01	0.00	1.75	0.00E+00	1.78E-03	#¡NUM!
F_Rhodanobacteraceae	NA	5.62E-01	1.20E-01	0.00	1.75	0.00E+00	1.79E-03	#¡NUM!
G_Aquimonas	NA	5.62E-01	1.20E-01	0.00	1.75	0.00E+00	1.94E-03	#¡NUM!
C_Betaproteobacteria	8.97E-01	9.81E-01	7.54E-02	401.89	212.74	4.08E-01	2.16E-01	9.19E-01
O_Burkholderiales	8.97E-01	8.19E-01	1.66E-02	401.79	169.11	4.09E-01	1.72E-01	1.25E+00
F_Sutterellaceae	9.00E-01	5.22E-01	4.17E-04	376.29	78.40	3.87E-01	8.03E-02	2.27E+00
G_Sutterella	9.00E-01	5.22E-01	6.19E-04	366.50	75.60	4.21E-01	8.39E-02	2.33E+00
G_Parasutterella	9.10E-01	5.70E-01	9.93E-03	9.04	2.17	1.04E-02	2.40E-03	2.11E+00
G_Duodenibacillus	5.84E-01	8.19E-01	7.23E-01	0.76	0.62	8.70E-04	6.92E-04	3.30E-01
F_Burkholderiaceae	9.17E-01	6.58E-01	3.27E-01	25.41	55.37	2.61E-02	5.67E-02	-1.12E+00
G_Ralstonia	9.17E-01	6.58E-01	3.27E-01	25.41	55.37	2.92E-02	6.14E-02	-1.07E+00
F_Comamonadaceae	4.38E-01	6.54E-01	4.95E-02	0.09	28.10	9.29E-05	2.88E-02	-8.27E+00
G_Hydrogenophaga	NA	7.89E-01	2.12E-01	0.00	2.46	0.00E+00	2.73E-03	#¡NUM!
G_Piscinibacter	NA	7.81E-01	1.12E-02	0.00	2.12	0.00E+00	2.35E-03	#¡NUM!
G_Simplicispira	NA	6.54E-01	2.41E-01	0.00	1.86	0.00E+00	2.06E-03	#¡NUM!
O_Nitrosomonadales	9.00E-01	6.54E-01	4.17E-02	0.10	32.27	9.71E-05	3.29E-02	-8.41E+00
F_Methylophilaceae	6.97E-01	7.89E-01	3.17E-02	0.02	16.36	2.20E-05	1.68E-02	-9.57E+00
G_Methylophilus	NA	8.32E-01	4.47E-02	0.00	8.21	0.00E+00	9.11E-03	#¡NUM!
G_Methylotenera	NA	5.97E-01	2.38E-01	0.00	3.18	0.00E+00	3.53E-03	#¡NUM!
F_Sterolibacteriaceae	6.97E-01	5.99E-01	7.95E-02	0.07	14.79	7.58E-05	1.51E-02	-7.64E+00
G_Methyloversatilis	6.97E-01	5.99E-01	7.95E-02	0.07	14.79	8.48E-05	1.64E-02	-7.60E+00
O_Neisseriales	NA	8.19E-01	3.94E-01	0.00	4.61	0.00E+00	4.70E-03	#¡NUM!
F_Neisseriaceae	NA	8.19E-01	3.94E-01	0.00	4.61	0.00E+00	4.72E-03	#¡NUM!
G_Neisseria	NA	8.19E-01	3.94E-01	0.00	4.61	0.00E+00	5.11E-03	#¡NUM!
O_Rhodocyclales	NA	8.19E-01	2.41E-01	0.00	2.79	0.00E+00	2.85E-03	#¡NUM!
F_Rhodocyclaceae	NA	8.19E-01	2.41E-01	0.00	2.79	0.00E+00	2.86E-03	#¡NUM!
G_Azospira	NA	8.19E-01	2.41E-01	0.00	2.79	0.00E+00	3.10E-03	#¡NUM!
C_Deltaproteobacteria	4.38E-01	8.83E-01	1.10E-01	36.03	72.27	3.66E-02	7.34E-02	-1.00E+00
O_Desulfovibrionales	4.38E-01	8.19E-01	2.20E-01	36.03	63.51	3.67E-02	6.48E-02	-8.18E-01
F_Desulfovibrionaceae	4.38E-01	8.19E-01	2.20E-01	36.03	63.51	3.70E-02	6.50E-02	-8.13E-01
G_Desulfovibrio	2.77E-01	8.19E-01	1.50E-01	25.32	56.56	2.91E-02	6.28E-02	-1.11E+00
G_Bilophila	4.38E-01	7.27E-01	4.18E-03	2.59	5.70	2.98E-03	6.33E-03	-1.09E+00
O_Myxococcales	NA	6.54E-01	9.24E-02	0.00	6.40	0.00E+00	6.53E-03	#¡NUM!
F_Polyangiaceae	NA	8.30E-01	7.95E-02	0.00	3.97	0.00E+00	4.07E-03	#¡NUM!
F_Kofleriaceae	NA	5.22E-01	1.89E-01	0.00	2.43	0.00E+00	2.48E-03	#¡NUM!
G_Kofleria	NA	5.22E-01	1.89E-01	0.00	2.43	0.00E+00	2.69E-03	#¡NUM!
C_Alphaproteobacteria	4.38E-01	6.33E-01	1.74E-01	0.18	40.12	1.86E-04	4.07E-02	-7.77E+00
O_Sphingomonadales	4.38E-01	6.33E-01	2.62E-01	0.18	14.12	1.87E-04	1.44E-02	-6.27E+00
F_Sphingomonadaceae	7.37E-01	6.33E-01	3.11E-01	0.02	12.57	2.45E-05	1.29E-02	-9.04E+00
G_Sphingomonas	NA	6.33E-01	3.94E-01	0.00	9.73	0.00E+00	1.08E-02	#¡NUM!
G_Sphingobium	7.37E-01	9.41E-01	3.02E-01	0.02	1.76	2.74E-05	1.95E-03	-6.16E+00
G_Aquisediminimonas	NA	9.42E-01	1.57E-01	0.00	1.08	0.00E+00	1.20E-03	#¡NUM!
F_Erythrobacteraceae	4.38E-01	9.06E-01	3.08E-01	0.16	1.55	1.64E-04	1.58E-03	-3.27E+00
O_Rhizobiales	NA	5.99E-01	2.36E-01	0.00	16.98	0.00E+00	1.73E-02	#¡NUM!
F_Methylobacteriaceae	NA	6.33E-01	3.94E-01	0.00	9.77	0.00E+00	1.00E-02	#¡NUM!
G_Methylobacterium	NA	6.33E-01	3.94E-01	0.00	9.77	0.00E+00	1.08E-02	#¡NUM!
F_Pleomorphomonadaceae	NA	5.99E-01	9.24E-02	0.00	5.79	0.00E+00	5.93E-03	#¡NUM!
G_Oharaeibacter	NA	5.99E-01	9.24E-02	0.00	5.79	0.00E+00	6.42E-03	#¡NUM!
O_Rhodospirillales	NA	9.57E-01	4.47E-02	0.00	9.02	0.00E+00	9.20E-03	#¡NUM!
F_Azospirillaceae	NA	8.15E-01	4.27E-02	0.00	7.06	0.00E+00	7.23E-03	#¡NUM!
G_Azospirillum	NA	8.15E-01	4.27E-02	0.00	7.06	0.00E+00	7.83E-03	#¡NUM!
F_Rhodospirillaceae	NA	6.54E-01	3.27E-01	0.00	1.96	0.00E+00	2.01E-03	#¡NUM!
G_Elstera	NA	6.54E-01	3.27E-01	0.00	1.96	0.00E+00	2.17E-03	#¡NUM!
P_Bacteroidetes	5.84E-01	5.96E-01	4.01E-01	20050.86	18758.06	2.03E+01	1.90E+01	9.61E-02
C_Bacteroidia	5.84E-01	5.96E-01	4.01E-01	20050.86	18758.06	2.04E+01	1.91E+01	9.72E-02
O_Bacteroidales	5.84E-01	5.96E-01	4.01E-01	20050.86	18758.06	2.04E+01	1.91E+01	9.54E-02
F_Bacteroidaceae	7.93E-01	5.70E-01	8.20E-01	18939.72	18578.30	1.95E+01	1.90E+01	3.26E-02
G_Bacteroides	8.43E-01	5.48E-01	5.18E-01	16392.03	17394.56	1.88E+01	1.93E+01	-3.46E-02
G_Phocaeicola	8.83E-01	5.22E-01	7.88E-04	1975.44	928.73	2.27E+00	1.03E+00	1.14E+00
F_Prevotellaceae	2.77E-01	5.22E-01	3.98E-03	542.56	76.20	5.57E-01	7.80E-02	2.84E+00
G_Prevotella	2.77E-01	5.22E-01	3.88E-03	534.83	70.70	6.15E-01	7.84E-02	2.97E+00
G_Paraprevotella	9.00E-01	5.45E-01	6.02E-02	7.74	0.24	8.89E-03	2.62E-04	5.09E+00
F_Porphyromonadaceae	8.83E-01	5.22E-01	3.58E-17	486.21	94.16	4.99E-01	9.64E-02	2.37E+00
G_Parabacteroides	8.83E-01	5.22E-01	5.67E-18	486.21	87.59	5.59E-01	9.72E-02	2.52E+00
G_Porphyromonas	NA	8.19E-01	3.94E-01	0.00	6.57	0.00E+00	7.29E-03	#¡NUM!
F_Muribaculaceae	8.13E-01	5.22E-01	6.70E-02	40.17	2.18	4.13E-02	2.23E-03	4.21E+00
G_Muribaculum	9.83E-01	NA	4.26E-02	2.61	0.00	3.00E-03	0.00E+00	#¡NUM!

F_Rikenellaceae	8.41E-01	7.89E-01	1.20E-01	1.80	0.83	1.85E-03	8.51E-04	1.12E+00
G_Alistipes	8.41E-01	7.89E-01	1.20E-01	1.80	0.83	2.07E-03	9.22E-04	1.17E+00
F_Odoribacteraceae	9.17E-01	5.22E-01	1.74E-05	3.71	0.47	3.81E-03	4.78E-04	3.00E+00
G_Odoribacter	9.41E-01	5.22E-01	2.08E-03	2.53	0.33	2.91E-03	3.62E-04	3.01E+00
G_Butyricimonas	9.60E-01	6.54E-01	9.17E-03	1.17	0.14	1.35E-03	1.56E-04	3.11E+00
F_Barnesiellaceae	9.17E-01	5.99E-01	3.95E-02	2.24	0.71	2.30E-03	7.26E-04	1.66E+00
G_Barnesiella	9.17E-01	5.99E-01	3.95E-02	2.24	0.71	2.57E-03	7.87E-04	1.71E+00
P_Actinobacteria	1.99E-02	5.22E-01	2.56E-02	2323.80	997.96	2.35E+00	1.01E+00	1.22E+00
C_Coriobacteriia	8.73E-02	5.22E-01	2.66E-01	1289.05	735.09	1.31E+00	7.47E-01	8.11E-01
O_Coriobacteriales	8.62E-02	5.22E-01	3.27E-01	1171.93	688.72	1.19E+00	7.02E-01	7.66E-01
F_Coriobacteriaceae	8.62E-02	5.22E-01	3.27E-01	1161.05	679.47	1.19E+00	6.96E-01	7.78E-01
G_Collinsella	1.27E-01	5.22E-01	2.88E-01	1054.57	563.55	1.21E+00	6.25E-01	9.55E-01
G_Senegalimassilia	4.76E-01	5.48E-01	8.50E-01	100.78	112.79	1.16E-01	1.25E-01	-1.11E-01
F_Atopobiaceae	3.85E-01	5.22E-01	7.79E-01	9.18	8.03	9.43E-03	8.22E-03	1.98E-01
G_Lancefieldella	8.41E-01	5.22E-01	3.63E-01	3.13	6.29	3.59E-03	6.98E-03	-9.57E-01
G_Olsenella	4.38E-01	7.25E-01	7.95E-02	6.05	1.74	6.95E-03	1.93E-03	1.85E+00
O_Eggerthellales	9.00E-01	7.27E-01	8.77E-03	116.44	46.13	1.19E-01	4.70E-02	1.33E+00
F_Eggerthellaceae	9.00E-01	7.27E-01	8.77E-03	116.44	46.13	1.20E-01	4.72E-02	1.34E+00
G_Eggerthella	8.83E-01	9.42E-01	2.56E-02	87.10	31.07	1.00E-01	3.45E-02	1.54E+00
G_Slackia	2.30E-01	5.48E-01	3.27E-01	26.15	13.73	3.01E-02	1.52E-02	9.80E-01
G_Adlercreutzia	4.56E-01	5.22E-01	3.17E-02	2.75	0.88	3.16E-03	9.80E-04	1.69E+00
C_Actinobacteria	8.62E-02	5.22E-01	1.49E-03	1034.75	262.86	1.05E+00	2.67E-01	1.98E+00
O_Bifidobacteriales	8.62E-02	5.22E-01	1.43E-03	1034.70	259.08	1.05E+00	2.64E-01	2.00E+00
F_Bifidobacteriaceae	8.62E-02	5.22E-01	1.43E-03	1034.70	259.08	1.06E+00	2.65E-01	2.00E+00
G_Bifidobacterium	8.62E-02	5.22E-01	1.43E-03	883.97	222.77	1.02E+00	2.47E-01	2.04E+00
O_Micrococcales	4.56E-01	8.19E-01	3.94E-01	0.05	3.79	4.85E-05	3.87E-03	-6.32E+00
F_Micrococcaceae	4.56E-01	8.19E-01	3.94E-01	0.05	3.79	4.89E-05	3.88E-03	-6.31E+00
G_Rothia	4.56E-01	8.19E-01	3.94E-01	0.05	3.79	5.47E-05	4.21E-03	-6.26E+00
P_Fusobacteria	4.38E-01	5.22E-01	3.59E-01	691.81	242.50	6.99E-01	2.45E-01	1.51E+00
C_Fusobacteriia	4.38E-01	5.22E-01	3.59E-01	691.81	242.50	7.03E-01	2.46E-01	1.51E+00
O_Fusobacteriales	4.38E-01	5.22E-01	3.59E-01	691.81	242.50	7.05E-01	2.47E-01	1.51E+00
F_Fusobacteriaceae	4.38E-01	5.22E-01	3.60E-01	680.05	238.87	6.99E-01	2.45E-01	1.51E+00
G_Fusobacterium	4.76E-01	5.53E-01	3.94E-01	0.68	1.15	7.77E-04	1.28E-03	-7.15E-01
P_Tenericutes	9.00E-01	5.55E-01	4.18E-01	3.58	1.85	3.62E-03	1.88E-03	9.49E-01
C_Mollicutes	9.00E-01	5.55E-01	4.18E-01	3.58	1.85	3.64E-03	1.88E-03	9.50E-01
O_Anaeroplasmatales	9.00E-01	5.55E-01	4.18E-01	3.58	1.85	3.65E-03	1.89E-03	9.48E-01
F_Anaeroplasmataceae	9.00E-01	5.55E-01	4.18E-01	3.58	1.85	3.68E-03	1.90E-03	9.54E-01
G_Anaeroplasma	9.00E-01	5.22E-01	4.74E-01	2.76	1.19	3.17E-03	1.32E-03	1.26E+00
G_Asteroleplasma	8.43E-01	5.99E-01	7.19E-01	0.82	0.66	9.41E-04	7.34E-04	3.58E-01
P_Verrucomicrobia	4.38E-01	9.68E-01	4.01E-01	1.19	0.75	1.20E-03	7.53E-04	6.70E-01
C_Verrucomicrobiae	4.38E-01	9.68E-01	4.01E-01	1.19	0.75	1.21E-03	7.57E-04	6.71E-01
O_Verrucomicrobiales	4.38E-01	9.68E-01	4.01E-01	1.19	0.75	1.21E-03	7.60E-04	6.69E-01
F_Akkermansiaceae	4.38E-01	9.68E-01	4.01E-01	1.19	0.75	1.22E-03	7.63E-04	6.75E-01
G_Akkermansia	4.38E-01	9.68E-01	4.01E-01	1.19	0.75	1.36E-03	8.27E-04	7.21E-01
P_Euryarchaeota	4.38E-01	8.19E-01	5.50E-01	0.60	0.41	6.07E-04	4.16E-04	5.43E-01
C_Methanobacteria	4.38E-01	8.19E-01	5.50E-01	0.60	0.41	6.10E-04	4.18E-04	5.44E-01
O_Methanobacteriales	4.38E-01	8.19E-01	5.50E-01	0.60	0.41	6.11E-04	4.20E-04	5.42E-01
F_Methanobacteriaceae	4.38E-01	8.19E-01	5.50E-01	0.60	0.41	6.16E-04	4.22E-04	5.47E-01
G_Methanobrevibacter	4.38E-01	8.19E-01	5.50E-01	0.60	0.41	6.90E-04	4.57E-04	5.94E-01

	**Significance for passage number (Model 1)**	
	**pFDR<0.05 in MCG-3**	**pFDR<0.05 in MCG-3B**
**Number of Taxa**	6	0
**Percentage of Taxa**	2.58%	0.00%
**Total number of Taxa**	233	

Number and percentage of taxa significantly affected by passage number

	**Significance for NDG presence (Model 2)**		
	**pFDR<0.05**	**pFDR<0.01**	**pFDR<0.001**
**Number of Taxa**	75	48	24
**Percentage of Taxa**	32.19%	20.60%	10.30%
**Total number of Taxa**	233		

Number and percentage of taxa significantly affected by NDG presence

Table S3. Data extracted from linear models performed for assessing the effect of passage number and NDG presence for OTUs. Table shows *p* FDR values obtained from multiple linear and linear mixed models performed for OTUs from passage 2 to passage 15. "Model 1" was used for assessing significance of passage number and "Model 2" was used for studying significance for NDG presence, by performing ANOVAs over the models generated. A false discovery rate (FDR) correction for multiple comparisons was applied to *p* values obtained. Coefficients for NDG presence (MCG-3) and NDG absence (MCG-3B) obtained from "Model 2" allowed to know whether OTUs levels were higher or lower in NDG presence compared to NDG absence.

	**Model 1 - lm(y~passage number)**		**Model 2 - lmer(y~NDG presence+(1|volunteer))**				
OTUs	*p* FDR passage number for MCG-3	*p* FDR passage number for MCG-3B	*p* FDR	Coeficient Model 2 - NDG presence	Coeficient Model 2 - NDG absence	BLAST best hit	RDP classiffication
OTU_1	7.07E-01	6.04E-01	4.81E-09	35057.59	45785.42	Pseudescherichia_vulneris_100	Bacteria_Proteobacteria_Gammaproteobacteria_Enterobacterales_Enterobacteriaceae_Escherichia/Shigella_100
OTU_9	8.74E-01	6.04E-01	7.05E-08	3858.01	7645.33	Bacteroides_faecis_100	Bacteria_Bacteroidetes_Bacteroidia_Bacteroidales_Bacteroidaceae_Bacteroides_100
OTU_6	9.38E-01	7.20E-01	3.51E-01	3918.32	4451.56	Enterococcus_hirae_100	Bacteria_Firmicutes_Bacilli_Lactobacillales_Enterococcaceae_Enterococcus_100
OTU_3	7.07E-01	6.04E-01	1.11E-04	6898.71	2859.15	Clostridium_perfringens_100	Bacteria_Firmicutes_Clostridia_Clostridiales_Clostridiaceae 1_Clostridium sensu stricto_99
OTU_20	8.18E-01	6.14E-01	1.72E-02	2564.51	4100.24	Veillonella_dispar_100	Bacteria_Firmicutes_Negativicutes_Veillonellales_Veillonellaceae_Veillonella_100
OTU_5	9.99E-01	9.12E-01	5.26E-03	567.02	4408.57	Bacteroides_fragilis_100	Bacteria_Bacteroidetes_Bacteroidia_Bacteroidales_Bacteroidaceae_Bacteroides_100
OTU_35	7.07E-01	7.38E-01	4.27E-06	1611.93	2472.28	Enterocloster_clostridioformis_100	Bacteria_Firmicutes_Clostridia_Clostridiales_Lachnospiraceae_Enterocloster_100
OTU_13	9.83E-01	6.04E-01	2.34E-02	2186.88	1588.57	Kineothrix_alysoides_98	Bacteria_Firmicutes_Clostridia_Clostridiales_Lachnospiraceae_Clostridium XlVa_63
OTU_10	7.07E-01	6.04E-01	5.70E-09	3874.71	614.94	Bacteroides_uniformis_100	Bacteria_Bacteroidetes_Bacteroidia_Bacteroidales_Bacteroidaceae_Bacteroides_100
OTU_15	8.74E-01	6.04E-01	4.07E-05	1712.43	468.47	Phocaeicola_vulgatus_100	Bacteria_Bacteroidetes_Bacteroidia_Bacteroidales_Bacteroidaceae_Phocaeicola_100
OTU_8	8.67E-01	8.79E-01	5.66E-03	2316.87	1055.54	Roseburia_inulinivorans_100	Bacteria_Firmicutes_Clostridia_Clostridiales_Lachnospiraceae_Roseburia_100
OTU_14	9.32E-01	6.05E-01	9.78E-02	1230.77	1631.39	Paraclostridium_benzoelyticum_100	Bacteria_Firmicutes_Clostridia_Clostridiales_Peptostreptococcaceae_Paraclostridium_100
OTU_21	9.46E-01	9.46E-01	1.45E-01	1428.49	1710.52	Bacteroides_kribbi_100	Bacteria_Bacteroidetes_Bacteroidia_Bacteroidales_Bacteroidaceae_Bacteroides_100
OTU_12	7.07E-01	6.04E-01	2.94E-01	924.40	506.66	Collinsella_aerofaciens_100	Bacteria_Actinobacteria_Coriobacteriia_Coriobacteriales_Coriobacteriaceae_Collinsella_100
OTU_17	9.16E-01	7.70E-01	2.75E-02	1391.60	919.73	Coprococcus_comes_100	Bacteria_Firmicutes_Clostridia_Clostridiales_Lachnospiraceae_Coprococcus_69
OTU_2	7.07E-01	7.51E-01	1.21E-02	2161.92	128.66	Bacteroides_stercoris_100	Bacteria_Bacteroidetes_Bacteroidia_Bacteroidales_Bacteroidaceae_Bacteroides_100
OTU_46	9.67E-01	6.73E-01	1.39E-03	1038.81	747.47	Clostridium_symbiosum_98	Bacteria_Firmicutes_Clostridia_Clostridiales_Lachnospiraceae_Clostridium XlVa_65
OTU_7	7.07E-01	9.41E-01	2.08E-03	533.63	89.85	Bifidobacterium_catenulatum_100	Bacteria_Actinobacteria_Actinobacteria_Bifidobacteriales_Bifidobacteriaceae_Bifidobacterium_100
OTU_40	8.97E-01	6.04E-01	8.43E-01	695.42	709.77	Hungatella_hathewayi_100	Bacteria_Firmicutes_Clostridia_Clostridiales_Lachnospiraceae_Hungatella_96
OTU_31	7.07E-01	6.63E-01	2.94E-01	419.69	522.68	Enterocloster_clostridioformis_96	Bacteria_Firmicutes_Clostridia_Clostridiales_Lachnospiraceae_Butyrivibrio_33
OTU_4	7.07E-01	6.04E-01	3.44E-01	635.40	224.60	Fusobacterium_perfoetens_95	Bacteria_Fusobacteria_Fusobacteriia_Fusobacteriales_Fusobacteriaceae_99_Fusobacterium_38
OTU_56	7.16E-01	6.10E-01	7.33E-03	676.65	225.23	Veillonella_atypica_100	Bacteria_Firmicutes_Negativicutes_Veillonellales_Veillonellaceae_Veillonella_100
OTU_131	7.76E-01	8.82E-01	1.36E-03	770.03	317.10	Bacteroides_xylanisolvens_99	Bacteria_Bacteroidetes_Bacteroidia_Bacteroidales_Bacteroidaceae_Bacteroides_100
OTU_27	8.48E-01	6.04E-01	4.18E-01	401.20	541.18	Erysipelatoclostridium_ramosum_100	Bacteria_Firmicutes_Erysipelotrichia_Erysipelotrichales_Erysipelatoclostridiaceae_Erysipelatoclostridium_100
OTU_32	7.07E-01	6.04E-01	1.08E-02	579.96	191.29	Peptoniphilus_phoceensis_100	Bacteria_Firmicutes_Clostridia_Clostridiales_Peptoniphilaceae_Peptoniphilus_100
OTU_42	7.12E-01	6.04E-01	5.43E-04	209.83	499.96	Phascolarctobacterium_faecium_100	Bacteria_Firmicutes_Negativicutes_Acidaminococcales_Acidaminococcaceae_Phascolarctobacterium_100
OTU_23	7.59E-01	7.73E-01	2.07E-02	328.92	188.31	Dorea_longicatena_100	Bacteria_Firmicutes_Clostridia_Clostridiales_Lachnospiraceae_Dorea_100
OTU_120	9.99E-01	9.70E-01	1.07E-04	280.84	363.20	Pseudescherichia_vulneris_98	Bacteria_Proteobacteria_95_Gammaproteobacteria_95_Enterobacterales_95_Enterobacteriaceae_94_Pseudescherichia_72
OTU_29	8.25E-01	6.04E-01	1.43E-01	410.59	202.14	Acidaminococcus_fermentans_97	Bacteria_Firmicutes_Negativicutes_Acidaminococcales_Acidaminococcaceae_Acidaminococcus_100
OTU_33	9.10E-01	8.14E-01	4.92E-02	218.34	102.19	Faecalibacterium_prausnitzii_99	Bacteria_Firmicutes_Clostridia_Clostridiales_Ruminococcaceae_Faecalibacterium_100
OTU_80	7.12E-01	6.83E-01	6.03E-06	578.49	140.17	Bacteroides_finegoldii_98	Bacteria_Bacteroidetes_Bacteroidia_Bacteroidales_Bacteroidaceae_Bacteroides_100
OTU_16	7.57E-01	6.04E-01	3.05E-02	724.43	20.89	Clostridium_cadaveris_100	Bacteria_Firmicutes_Clostridia_Clostridiales_Clostridiaceae 1_Clostridium sensu stricto_99
OTU_30	1.00E+00	9.53E-01	2.53E-03	56.39	157.65	Phocaeicola_coprophilus_100	Bacteria_Bacteroidetes_Bacteroidia_Bacteroidales_Bacteroidaceae_Phocaeicola_100
OTU_34	7.07E-01	8.68E-01	6.27E-04	678.60	37.92	Streptococcus_parasanguinis_100	Bacteria_Firmicutes_Bacilli_Lactobacillales_Streptococcaceae_Streptococcus_100
OTU_423	8.16E-01	8.36E-01	6.69E-03	282.44	416.32	Bacteroides_thetaiotaomicron_98	Bacteria_Bacteroidetes_Bacteroidia_Bacteroidales_Bacteroidaceae_Bacteroides_100
OTU_162	8.25E-01	7.38E-01	3.79E-01	247.65	234.68	Pseudescherichia_vulneris_97	Bacteria_Proteobacteria_Gammaproteobacteria_Enterobacterales_Enterobacteriaceae_Escherichia/Shigella_88
OTU_19	7.07E-01	6.04E-01	5.91E-02	295.58	61.97	Prevotella_histicola_94	Bacteria_Bacteroidetes_Bacteroidia_Bacteroidales_Prevotellaceae_Prevotella_99
OTU_36	8.83E-01	6.52E-01	7.73E-01	163.36	168.76	Flavonifractor_plautii_100	Bacteria_Firmicutes_Clostridia_Clostridiales_Ruminococcaceae_Flavonifractor_100
OTU_7572	8.25E-01	9.57E-01	1.33E-01	277.78	216.72	Bacteroides_xylanisolvens_100	Bacteria_Bacteroidetes_Bacteroidia_Bacteroidales_Bacteroidaceae_Bacteroides_100
OTU_52	7.07E-01	6.62E-01	3.02E-02	113.34	286.00	Faecalibacterium_prausnitzii_95	Bacteria_Firmicutes_Clostridia_Clostridiales_Ruminococcaceae_Faecalibacterium_47
OTU_24	9.08E-01	7.73E-01	7.60E-02	210.74	69.68	Succinivibrio_dextrinosolvens_97	Bacteria_Proteobacteria_Gammaproteobacteria_Aeromonadales_Succinivibrionaceae_Succinivibrio_100
OTU_25	8.20E-01	6.04E-01	6.45E-02	263.43	14.79	Streptococcus_salivarius_100	Bacteria_Firmicutes_Bacilli_Lactobacillales_Streptococcaceae_Streptococcus_100
OTU_85	8.53E-01	8.64E-01	1.33E-02	147.99	210.33	Enterocloster_aldenensis_100	Bacteria_Firmicutes_Clostridia_Clostridiales_Lachnospiraceae_Enterocloster_94
OTU_4781	7.07E-01	9.25E-01	2.94E-02	272.17	158.11	Bacteroides_xylanisolvens_98	Bacteria_Bacteroidetes_Bacteroidia_Bacteroidales_Bacteroidaceae_Bacteroides_100
OTU_60	8.85E-01	6.04E-01	4.19E-09	271.75	44.27	Parabacteroides_distasonis_100	Bacteria_Bacteroidetes_Bacteroidia_Bacteroidales_Porphyromonadaceae_Parabacteroides_100
OTU_37	7.07E-01	6.04E-01	5.03E-01	119.99	216.20	Dialister_succinatiphilus_97	Bacteria_Firmicutes_Negativicutes_Veillonellales_Veillonellaceae_Dialister_100
OTU_59	9.93E-01	8.73E-01	9.99E-01	206.99	206.89	Bacteroides_cellulosilyticus_100	Bacteria_Bacteroidetes_Bacteroidia_Bacteroidales_Bacteroidaceae_Bacteroides_100
OTU_26	7.10E-01	7.08E-01	7.02E-01	129.46	83.50	Limosilactobacillus_mucosae_100	Bacteria_Firmicutes_Bacilli_Lactobacillales_Lactobacillaceae_Limosilactobacillus_100
OTU_1839	7.70E-01	6.50E-01	3.96E-01	161.99	129.61	Veillonella_atypica_98	Bacteria_Firmicutes_Negativicutes_Veillonellales_Veillonellaceae_Veillonella_100
OTU_38	9.78E-01	6.37E-01	2.07E-02	43.38	109.65	Phocaeicola_plebeius_100	Bacteria_Bacteroidetes_Bacteroidia_Bacteroidales_Bacteroidaceae_Phocaeicola_100
OTU_10582	7.88E-01	6.04E-01	1.40E-02	104.99	152.57	Enterococcus_faecalis_98	Bacteria_Firmicutes_Bacilli_Lactobacillales_99_Enterococcaceae_99_Enterococcus_88
OTU_66	7.57E-01	6.04E-01	9.35E-03	203.67	117.63	Clostridium_scindens_100	Bacteria_Firmicutes_Clostridia_Clostridiales_Lachnospiraceae_Clostridium XlVa_100
OTU_657	8.57E-01	9.34E-01	7.71E-04	126.82	187.81	Bacteroides_thetaiotaomicron_99	Bacteria_Bacteroidetes_Bacteroidia_Bacteroidales_Bacteroidaceae_Bacteroides_100
OTU_119	7.81E-01	6.04E-01	1.69E-05	234.36	61.14	Clostridium_perfringens_96	Bacteria_Firmicutes_Clostridia_Clostridiales_Lachnospiraceae_45_Mobilitalea_5
OTU_143	9.74E-01	6.04E-01	1.54E-06	75.65	129.51	Pseudescherichia_vulneris_89	Bacteria_Firmicutes_51_Bacilli_51_Lactobacillales_50_Carnobacteriaceae_37_Isobaculum_32
OTU_31227	7.94E-01	8.17E-01	3.47E-03	84.92	195.53	Bacteroides_thetaiotaomicron_100	Bacteria_Bacteroidetes_Bacteroidia_Bacteroidales_Bacteroidaceae_Bacteroides_100
OTU_79	8.29E-01	6.62E-01	1.09E-05	137.63	19.71	Phocaeicola_paurosaccharolyticus_96	Bacteria_Bacteroidetes_Bacteroidia_Bacteroidales_Bacteroidaceae_Bacteroides_68
OTU_94	9.95E-01	7.16E-01	7.05E-08	62.33	164.33	Bacteroides_fragilis_99	Bacteria_Bacteroidetes_74_Bacteroidia_74_Bacteroidales_74_Bacteroidaceae_74_Bacteroides_43
OTU_9822	7.07E-01	9.25E-01	8.31E-04	95.62	23.51	Bifidobacterium_stercoris_98	Bacteria_Actinobacteria_Actinobacteria_Bifidobacteriales_Bifidobacteriaceae_Bifidobacterium_100

OTU_153	9.39E-01	6.04E-01	3.54E-06	62.54	109.47	Enterococcus_faecalis_97	Bacteria_Firmicutes_Bacilli_Lactobacillales_99_Enterococcaceae_87_Enterococcus_65
OTU_109	9.84E-01	6.82E-01	1.82E-03	51.26	121.50	Veillonella_dispar_91	Bacteria_Firmicutes_Negativicutes_89_Veillonellales_60_Veillonellaceae_60_Veillonella_60
OTU_117	8.57E-01	6.04E-01	1.45E-04	137.14	61.44	Pseudescherichia_vulneris_97	Bacteria_Proteobacteria_99_Gammaproteobacteria_99_Enterobacterales_99_Enterobacteriaceae_98_Pseudescherichia_75
OTU_134	8.57E-01	6.50E-01	5.98E-06	120.44	22.16	Bacteroides_caecimuris_96	Bacteria_Bacteroidetes_Bacteroidia_Bacteroidales_Bacteroidaceae_Bacteroides_100
OTU_44	8.19E-01	7.55E-01	1.54E-02	184.22	29.34	Sutterella_massiliensis_100	Bacteria_Proteobacteria_Betaproteobacteria_Burkholderiales_Sutterellaceae_Sutterella_100
OTU_53	7.57E-01	6.83E-01	3.87E-01	8.95	15.48	Haemophilus_parainfluenzae_100	Bacteria_Proteobacteria_Gammaproteobacteria_Pasteurellales_Pasteurellaceae_Haemophilus_100
OTU_355	7.07E-01	9.52E-01	7.26E-03	72.03	19.16	Bifidobacterium_stercoris_98	Bacteria_Actinobacteria_Actinobacteria_Bifidobacteriales_Bifidobacteriaceae_Bifidobacterium_100
OTU_168	8.19E-01	9.34E-01	2.99E-03	60.15	115.93	Veillonella_dispar_87	Bacteria_Proteobacteria_81_Gammaproteobacteria_81_Enterobacterales_79_Enterobacteriaceae_79_Pseudescherichia_79
OTU_50	7.39E-01	6.04E-01	2.82E-02	182.11	3.60	Prevotella_copri_99	Bacteria_Bacteroidetes_Bacteroidia_Bacteroidales_Prevotellaceae_Prevotella_100
OTU_100973	9.71E-01	6.05E-01	3.89E-01	53.23	74.21	Enterococcus_faecalis_98	Bacteria_Firmicutes_Bacilli_Lactobacillales_Enterococcaceae_Enterococcus_97
OTU_244	9.27E-01	6.04E-01	4.09E-02	72.50	86.99	Hungatella_hathewayi_91	Bacteria_Firmicutes_Bacilli_77_Lactobacillales_74_Carnobacteriaceae_56_Isobaculum_49
OTU_148	9.87E-01	6.14E-01	4.57E-06	56.70	91.90	Enterococcus_faecalis_89	Bacteria_Proteobacteria_92_Gammaproteobacteria_92_Enterobacterales_92_Enterobacteriaceae_91_Pseudescherichia_83
OTU_28	9.84E-01	9.12E-01	5.62E-02	19.60	6.60	Megasphaera_elsdenii_99	Bacteria_Firmicutes_Negativicutes_Veillonellales_Veillonellaceae_Megasphaera_100
OTU_61	9.04E-01	6.04E-01	1.19E-02	108.19	27.42	Sutterella_wadsworthensis_100	Bacteria_Proteobacteria_Betaproteobacteria_Burkholderiales_Sutterellaceae_Sutterella_100
OTU_116	9.37E-01	6.90E-01	3.25E-04	77.92	123.67	Eisenbergiella_tayi_100	Bacteria_Firmicutes_Clostridia_Clostridiales_Lachnospiraceae_Eisenbergiella_98
OTU_281	9.78E-01	8.18E-01	9.38E-06	50.35	94.59	Enterocloster_citroniae_98	Bacteria_Firmicutes_89_Clostridia_89_Clostridiales_89_Lachnospiraceae_89_Enterocloster_81
OTU_409	9.92E-01	7.31E-01	8.07E-04	65.37	80.74	Pseudescherichia_vulneris_93	Bacteria_Proteobacteria_Gammaproteobacteria_Enterobacterales_Enterobacteriaceae_99_Pseudescherichia_98
OTU_35755	8.16E-01	5.62E-01	9.27E-03	40.20	82.05	Enterococcus_faecalis_98	Bacteria_Firmicutes_Bacilli_Lactobacillales_Enterococcaceae_99_Enterococcus_94
OTU_41	7.10E-01	6.50E-01	8.51E-01	92.69	105.45	Senegalimassilia_anaerobia_100	Bacteria_Actinobacteria_Coriobacteriia_Coriobacteriales_Coriobacteriaceae_Senegalimassilia_100
OTU_55	7.07E-01	6.63E-01	5.12E-01	48.89	25.51	Enterobacter_cloacae_100	Bacteria_Proteobacteria_Gammaproteobacteria_Enterobacterales_Enterobacteriaceae_Enterobacter_97
OTU_128	9.87E-01	9.46E-01	5.32E-04	39.00	100.82	Gibbsiella_dentisursi_90	Bacteria_Proteobacteria_99_Gammaproteobacteria_99_Enterobacterales_98_Enterobacteriaceae_98_Pseudescherichia_95
OTU_18	9.64E-01	9.21E-01	5.30E-01	15.02	22.17	Ligilactobacillus_ruminis_100	Bacteria_Firmicutes_Bacilli_Lactobacillales_Lactobacillaceae_Ligilactobacillus_100
OTU_130	7.73E-01	1.22E-01	3.93E-04	93.11	46.29	Clostridium_carnis_92	Bacteria_Firmicutes_Bacilli_99_Lactobacillales_95_Carnobacteriaceae_47_Isobaculum_47
OTU_2194	7.12E-01	9.25E-01	2.00E-01	79.59	57.10	Veillonella_parvula_98	Bacteria_Firmicutes_Negativicutes_Veillonellales_Veillonellaceae_Veillonella_100
OTU_375	8.49E-01	7.07E-01	1.43E-01	76.56	57.95	Veillonella_parvula_98	Bacteria_Firmicutes_Negativicutes_Veillonellales_Veillonellaceae_Veillonella_100
OTU_49	7.75E-01	6.04E-01	2.45E-02	107.78	13.57	Lactobacillus_johnsonii_100	Bacteria_Firmicutes_Bacilli_Lactobacillales_Lactobacillaceae_Lactobacillus_100
OTU_74	9.92E-01	8.14E-01	8.47E-02	39.17	90.99	Flintibacter_butyricus_97	Bacteria_Firmicutes_Clostridia_Clostridiales_Ruminococcaceae_Flintibacter_92
OTU_63	8.16E-01	9.27E-01	6.28E-01	28.12	36.82	Anaerobutyricum_hallii_99	Bacteria_Firmicutes_Clostridia_Clostridiales_Lachnospiraceae_Anaerobutyricum_100
OTU_152	9.87E-01	6.32E-01	3.39E-03	35.28	83.55	Enterococcus_dispar_91	Bacteria_Firmicutes_Bacilli_97_Lactobacillales_78_Carnobacteriaceae_66_Isobaculum_48
OTU_51	9.80E-01	6.04E-01	5.83E-02	129.77	26.04	Turicibacter_sanguinis_96	Bacteria_Firmicutes_Erysipelotrichia_Erysipelotrichales_Erysipelotrichaceae_Turicibacter_100
OTU_48	7.57E-01	7.66E-01	2.47E-02	19.46	92.06	Clostridium_tepidum_100	Bacteria_Firmicutes_Clostridia_Clostridiales_Clostridiaceae 1_Clostridium sensu stricto_100
OTU_462	7.11E-01	9.61E-01	1.09E-05	103.04	13.07	Bacteroides_uniformis_97	Bacteria_Bacteroidetes_Bacteroidia_Bacteroidales_Bacteroidaceae_Bacteroides_44
OTU_175	8.25E-01	6.04E-01	6.59E-05	98.70	39.31	Escherichia_albertii_89	Bacteria_Firmicutes_56_Clostridia_56_Clostridiales_56_Clostridiaceae 1_56_Desnuesiella_22
OTU_58	8.25E-01	6.04E-01	1.74E-02	126.67	37.83	Faecalicatena_orotica_98	Bacteria_Firmicutes_Clostridia_Clostridiales_Lachnospiraceae_Mediterraneibacter_54
OTU_86	8.16E-01	9.42E-01	1.95E-01	59.33	49.90	Blautia_obeum_100	Bacteria_Firmicutes_Clostridia_Clostridiales_Lachnospiraceae_Blautia_100
OTU_266	9.67E-01	6.27E-01	1.08E-01	56.12	69.92	Clostridium_symbiosum_96	Bacteria_Firmicutes_72_Clostridia_72_Clostridiales_72_Lachnospiraceae_71_Clostridium XlVa_15
OTU_64	7.07E-01	8.25E-01	5.66E-03	32.13	79.51	Eubacterium_callanderi_98	Bacteria_Firmicutes_Clostridia_Clostridiales_Eubacteriaceae_Eubacterium_40
OTU_176	8.67E-01	9.82E-01	1.97E-02	41.13	86.89	Pseudescherichia_vulneris_99	Bacteria_Proteobacteria_97_Gammaproteobacteria_97_Enterobacterales_97_Enterobacteriaceae_97_Pseudescherichia_63
OTU_198	9.59E-01	8.50E-01	5.13E-01	72.44	64.12	Escherichia_albertii_89	Bacteria_Firmicutes_78_Clostridia_78_Clostridiales_78_Lachnospiraceae_78_Roseburia_48
OTU_302	7.07E-01	7.25E-01	2.75E-05	87.63	10.35	Phocaeicola_dorei_97	Bacteria_Bacteroidetes_Bacteroidia_Bacteroidales_Bacteroidaceae_Bacteroides_78
OTU_67	7.07E-01	9.75E-01	6.41E-02	36.05	87.28	Bacteroides_nordii_100	Bacteria_Bacteroidetes_Bacteroidia_Bacteroidales_Bacteroidaceae_Bacteroides_100
OTU_47	8.06E-01	6.04E-01	4.56E-01	83.85	43.59	Clostridium_saudiense_100	Bacteria_Firmicutes_Clostridia_Clostridiales_Clostridiaceae 1_Clostridium sensu stricto_100
OTU_20340	8.54E-01	9.46E-01	6.20E-01	55.44	64.11	Veillonella_parvula_100	Bacteria_Firmicutes_Negativicutes_Veillonellales_Veillonellaceae_Veillonella_100
OTU_140	1.00E+00	6.83E-01	3.61E-04	12.07	82.96	Bacteroides_faecichinchillae_96	Bacteria_Bacteroidetes_Bacteroidia_Bacteroidales_Bacteroidaceae_Bacteroides_93
OTU_158	8.08E-01	4.60E-01	2.61E-04	81.42	34.43	Clostridium_perfringens_97	Bacteria_Firmicutes_Clostridia_Clostridiales_Clostridiaceae 1_99_Sarcina_24
OTU_95	7.07E-01	9.25E-01	8.81E-01	32.37	33.48	Dorea_formicigenerans_99	Bacteria_Firmicutes_Clostridia_Clostridiales_Lachnospiraceae_Dorea_95
OTU_474	7.80E-01	6.04E-01	5.21E-01	49.70	53.50	Enterococcus_faecalis_97	Bacteria_Firmicutes_Bacilli_Lactobacillales_Enterococcaceae_93_Enterococcus_89
OTU_39	7.46E-01	8.28E-01	1.41E-01	52.37	27.34	Pseudoflavonifractor_phocaeensis_97	Bacteria_Firmicutes_Clostridia_Clostridiales_Ruminococcaceae_Intestinimonas_55
OTU_554	7.07E-01	6.57E-01	1.17E-02	68.48	29.25	Veillonella_atypica_97	Bacteria_Firmicutes_Negativicutes_Veillonellales_Veillonellaceae_Veillonella_100
OTU_303	9.87E-01	7.73E-01	1.39E-01	39.27	51.15	Veillonella_parvula_98	Bacteria_Firmicutes_Negativicutes_63_Veillonellales_62_Veillonellaceae_62_Veillonella_61
OTU_164	7.07E-01	6.04E-01	8.39E-07	94.87	30.41	Bacteroides_kribbi_97	Bacteria_Bacteroidetes_Bacteroidia_Bacteroidales_Bacteroidaceae_Bacteroides_100
OTU_223	9.54E-01	7.31E-01	1.57E-03	16.63	60.36	Phocaeicola_coprophilus_96	Bacteria_Bacteroidetes_Bacteroidia_Bacteroidales_Bacteroidaceae_Phocaeicola_89
OTU_123	7.07E-01	6.04E-01	7.42E-02	33.89	15.30	Collinsella_aerofaciens_94	Bacteria_Actinobacteria_95_Coriobacteriia_95_Coriobacteriales_95_Coriobacteriaceae_95_Collinsella_94
OTU_314	8.25E-01	9.69E-01	4.39E-03	38.43	52.11	Enterococcus_hirae_92	Bacteria_Proteobacteria_58_Gammaproteobacteria_58_Enterobacterales_58_Enterobacteriaceae_57_Pseudescherichia_55
OTU_323	8.57E-01	2.47E-01	1.45E-02	35.80	52.00	Enterococcus_faecalis_98	Bacteria_Firmicutes_Bacilli_Lactobacillales_Enterococcaceae_96_Enterococcus_88
OTU_69	7.93E-01	6.04E-01	9.42E-02	82.46	35.01	Ruminococcus_gnavus_100	Bacteria_Firmicutes_Clostridia_Clostridiales_Lachnospiraceae_Lachnospiracea_incertae_sedis_82
OTU_150	7.46E-01	4.38E-02	3.75E-04	59.07	22.46	Enterococcus_faecalis_92	Bacteria_Firmicutes_Clostridia_67_Clostridiales_67_Clostridiaceae 1_67_Sarcina_25
OTU_111226	9.01E-01	8.28E-01	2.42E-02	115.63	0.69	Bacteroides_xylanisolvens_98	Bacteria_Bacteroidetes_Bacteroidia_Bacteroidales_Bacteroidaceae_Bacteroides_100
OTU_70	8.94E-01	9.80E-01	1.87E-02	86.34	30.30	Eggerthella_lenta_100	Bacteria_Actinobacteria_Coriobacteriia_Eggerthellales_Eggerthellaceae_Eggerthella_100
OTU_159	9.85E-01	6.63E-01	2.79E-03	83.53	18.61	Kineothrix_alysoides_93	Bacteria_Firmicutes_Clostridia_Clostridiales_Lachnospiraceae_98_Clostridium XlVa_38
OTU_763	8.94E-01	7.36E-01	4.97E-02	61.13	42.04	Falcatimonas_natans_97	Bacteria_Firmicutes_Clostridia_Clostridiales_Lachnospiraceae_Enterocloster_36
OTU_124	9.04E-01	6.05E-01	1.29E-02	78.52	18.17	Pseudescherichia_vulneris_94	Bacteria_Proteobacteria_Gammaproteobacteria_83_Enterobacterales_83_Enterobacteriaceae_83_Pseudescherichia_80
OTU_126	7.94E-01	8.14E-01	2.19E-02	57.38	40.31	Pseudescherichia_vulneris_99	Bacteria_Proteobacteria_98_Gammaproteobacteria_98_Enterobacterales_98_Enterobacteriaceae_98_Pseudescherichia_91
OTU_490	9.91E-01	8.73E-01	1.18E-03	26.78	63.43	Veillonella_dispar_96	Bacteria_Firmicutes_99_Negativicutes_98_Veillonellales_98_Veillonellaceae_98_Veillonella_98
OTU_76	7.07E-01	9.23E-01	1.28E-01	22.64	52.96	Desulfovibrio_piger_99	Bacteria_Proteobacteria_Deltaproteobacteria_Desulfovibrionales_Desulfovibrionaceae_Desulfovibrio_99
OTU_264	7.07E-01	6.04E-01	9.27E-03	60.69	14.95	Bifidobacterium_longum_91	Bacteria_Actinobacteria_83_Actinobacteria_83_Bifidobacteriales_83_Bifidobacteriaceae_83_Pseudoscardovia_42
OTU_3338	9.33E-01	9.75E-01	9.25E-10	28.82	73.10	Bacteroides_faecichinchillae_98	Bacteria_Bacteroidetes_Bacteroidia_Bacteroidales_Bacteroidaceae_Bacteroides_100

OTU_246	8.59E-01	6.04E-01	3.09E-02	27.44	40.66	Enterococcus_faecalis_97	Bacteria_Firmicutes_Bacilli_Lactobacillales_Enterococcaceae_90_Enterococcus_78
OTU_318	7.43E-01	8.70E-01	5.32E-04	92.69	8.15	Bacteroides_xylanisolvens_98	Bacteria_Bacteroidetes_Bacteroidia_Bacteroidales_Bacteroidaceae_Bacteroides_100
OTU_393	8.33E-01	6.72E-01	2.82E-08	56.07	12.08	Parabacteroides_distasonis_98	Bacteria_Bacteroidetes_Bacteroidia_Bacteroidales_Porphyromonadaceae_Parabacteroides_100
OTU_341	7.07E-01	8.88E-01	1.60E-02	84.51	11.05	Bacteroides_stercoris_97	Bacteria_Bacteroidetes_Bacteroidia_Bacteroidales_Bacteroidaceae_Bacteroides_100
OTU_227	8.16E-01	4.60E-01	8.56E-05	57.65	22.71	Clostridium_perfringens_91	Bacteria_Proteobacteria_80_Gammaproteobacteria_80_Enterobacterales_79_Enterobacteriaceae_78_Pseudescherichia_70
OTU_65	9.46E-01	9.01E-01	6.95E-01	26.49	22.88	Gemmiger_formicilis_100	Bacteria_Firmicutes_Clostridia_Clostridiales_Ruminococcaceae_Gemmiger_95
OTU_1025	7.07E-01	6.04E-01	8.39E-07	76.98	9.00	Bacteroides_faecichinchillae_96	Bacteria_Bacteroidetes_Bacteroidia_Bacteroidales_Bacteroidaceae_Bacteroides_99
OTU_166	7.07E-01	9.71E-01	2.10E-03	59.28	12.11	Pseudescherichia_vulneris_90	Bacteria_Proteobacteria_Gammaproteobacteria_Enterobacterales_Enterobacteriaceae_Pseudescherichia_46
OTU_78	9.67E-01	7.56E-01	3.03E-01	24.59	52.13	Ralstonia_pickettii_100	Bacteria_Proteobacteria_Betaproteobacteria_Burkholderiales_Burkholderiaceae_Ralstonia_100
OTU_858	9.99E-01	8.72E-01	1.54E-03	24.73	12.17	Bacteroides_caccae_99	Bacteria_Bacteroidetes_Bacteroidia_Bacteroidales_Bacteroidaceae_Bacteroides_100
OTU_15897	7.10E-01	7.24E-01	7.10E-04	63.46	10.14	Streptococcus_mitis_98	Bacteria_Firmicutes_Bacilli_Lactobacillales_Streptococcaceae_Streptococcus_100
OTU_365	9.61E-01	6.90E-01	6.48E-07	22.23	46.33	Enterocloster_citroniae_97	Bacteria_Firmicutes_Clostridia_Clostridiales_Lachnospiraceae_Enterocloster_100
OTU_75	9.13E-01	8.14E-01	6.82E-02	29.35	13.36	Allisonella_histaminiformans_100	Bacteria_Firmicutes_Negativicutes_Veillonellales_Veillonellaceae_Allisonella_100
OTU_567	7.07E-01	5.09E-01	1.79E-06	76.41	10.26	Bacteroides_caecimuris_98	Bacteria_Bacteroidetes_Bacteroidia_Bacteroidales_Bacteroidaceae_Bacteroides_100
OTU_72	7.80E-01	6.05E-01	2.66E-02	7.72	2.51	Megamonas_funiformis_98	Bacteria_Firmicutes_Negativicutes_Selenomonadales_Selenomonadaceae_Megamonas_100
OTU_600	7.91E-01	8.88E-01	4.56E-02	47.37	34.17	Hungatella_effluvii_97	Bacteria_Firmicutes_Clostridia_Clostridiales_Lachnospiraceae_Hungatella_53
OTU_73	8.06E-01	6.63E-01	3.25E-01	4.59	7.80	Phocaeicola_coprocola_100	Bacteria_Bacteroidetes_Bacteroidia_Bacteroidales_Bacteroidaceae_Phocaeicola_100
OTU_921	8.20E-01	8.68E-01	2.77E-01	40.43	34.34	Coprococcus_comes_97	Bacteria_Firmicutes_Clostridia_Clostridiales_Lachnospiraceae_Coprococcus_37
OTU_118	9.00E-01	6.04E-01	1.72E-02	53.53	13.79	Pseudescherichia_vulneris_94	Bacteria_Proteobacteria_Gammaproteobacteria_88_Enterobacterales_88_Enterobacteriaceae_88_Pseudescherichia_85
OTU_96	8.53E-01	6.04E-01	1.28E-01	45.97	13.69	Faecalibaculum_rodentium_89	Bacteria_Firmicutes_Erysipelotrichia_Erysipelotrichales_Erysipelotrichaceae_Faecalibaculum_44
OTU_216	9.31E-01	4.60E-01	7.99E-02	38.48	26.61	Clostridium_tarantellae_97	Bacteria_Firmicutes_Clostridia_Clostridiales_Clostridiaceae 1_83_Clostridium sensu stricto_58
OTU_212	7.07E-01	9.62E-01	1.60E-02	22.26	46.73	Paraclostridium_benzoelyticum_99	Bacteria_Firmicutes_84_Clostridia_84_Clostridiales_84_Peptostreptococcaceae_84_Paraclostridium_84
OTU_57	9.49E-01	6.04E-01	5.28E-03	6.79	1.64	Holdemanella_biformis_98	Bacteria_Firmicutes_Erysipelotrichia_Erysipelotrichales_Erysipelotrichaceae_Holdemanella_100
OTU_394	9.65E-01	6.04E-01	9.01E-03	22.31	39.22	Enterococcus_faecalis_98	Bacteria_Firmicutes_Bacilli_Lactobacillales_Enterococcaceae_96_Enterococcus_83
OTU_62	7.07E-01	6.47E-01	3.08E-01	20.09	11.09	Slackia_isoflavoniconvertens_100	Bacteria_Actinobacteria_Coriobacteriia_Eggerthellales_Eggerthellaceae_Slackia_100
OTU_82	7.84E-01	9.25E-01	1.25E-02	14.85	46.75	Phocaeicola_coprocola_97	Bacteria_Bacteroidetes_Bacteroidia_Bacteroidales_Bacteroidaceae_Phocaeicola_100
OTU_338	9.37E-01	8.18E-01	6.69E-03	28.60	37.24	Pseudescherichia_vulneris_95	Bacteria_Proteobacteria_Gammaproteobacteria_Enterobacterales_Enterobacteriaceae_Pseudescherichia_62
OTU_3399	7.07E-01	6.72E-01	2.35E-02	20.65	53.36	Faecalibacterium_prausnitzii_94	Bacteria_Firmicutes_Clostridia_Clostridiales_Ruminococcaceae_Faecalibacterium_71
OTU_404	9.92E-01	7.16E-01	8.89E-01	33.89	33.07	Coprococcus_comes_98	Bacteria_Firmicutes_92_Clostridia_92_Clostridiales_92_Lachnospiraceae_92_Bariatricus_84
OTU_188	9.92E-01	6.14E-01	2.39E-04	5.74	51.34	Bacteroides_faecis_97	Bacteria_Bacteroidetes_Bacteroidia_Bacteroidales_Bacteroidaceae_Bacteroides_97
OTU_535	7.12E-01	5.32E-01	2.61E-04	43.59	17.36	Enterocloster_citroniae_94	Bacteria_Firmicutes_Clostridia_Clostridiales_Lachnospiraceae_98_Enterocloster_85
OTU_184	9.86E-01	9.46E-01	2.61E-04	17.39	46.77	Veillonella_dispar_96	Bacteria_Firmicutes_Negativicutes_Veillonellales_99_Veillonellaceae_99_Veillonella_96
OTU_285	9.59E-01	8.10E-01	1.12E-05	13.85	47.91	Bacteroides_fragilis_100	Bacteria_Bacteroidetes_92_Bacteroidia_92_Bacteroidales_92_Bacteroidaceae_92_Bacteroides_89
OTU_256	9.78E-01	8.08E-01	1.64E-01	27.61	33.18	Clostridium_symbiosum_95	Bacteria_Firmicutes_Clostridia_Clostridiales_Lachnospiraceae_Clostridium XlVa_48
OTU_192	9.67E-01	9.70E-01	2.66E-01	41.05	32.69	Roseburia_inulinivorans_97	Bacteria_Firmicutes_Clostridia_Clostridiales_Lachnospiraceae_Roseburia_100
OTU_228	7.07E-01	4.60E-01	3.15E-03	44.21	18.91	Veillonella_dispar_98	Bacteria_Firmicutes_Negativicutes_87_Veillonellales_81_Veillonellaceae_81_Veillonella_81
OTU_211	7.07E-01	6.39E-01	1.76E-03	48.21	5.00	Bacteroides_eggerthii_96	Bacteria_Bacteroidetes_Bacteroidia_Bacteroidales_Bacteroidaceae_Bacteroides_87
OTU_317	9.43E-01	7.65E-01	7.97E-03	20.89	45.92	Paraclostridium_benzoelyticum_95	Bacteria_Firmicutes_Clostridia_Clostridiales_Peptostreptococcaceae_82_Paraclostridium_80
OTU_122	7.50E-01	7.73E-01	3.27E-01	11.17	8.48	Anaerostipes_hadrus_100	Bacteria_Firmicutes_Clostridia_Clostridiales_Lachnospiraceae_Anaerostipes_100
OTU_224	9.64E-01	9.12E-01	1.32E-02	12.41	48.07	Phocaeicola_plebeius_96	Bacteria_Bacteroidetes_Bacteroidia_Bacteroidales_Bacteroidaceae_Phocaeicola_74
OTU_54	9.78E-01	6.04E-01	3.33E-07	41.68	1.21	Anaerotignum_faecicola_95	Bacteria_Firmicutes_Clostridia_Clostridiales_Lachnospiraceae_94_Anaerotignum_91
OTU_204	7.12E-01	6.04E-01	1.46E-01	31.57	20.47	Pseudescherichia_vulneris_99	Bacteria_Proteobacteria_Gammaproteobacteria_Enterobacterales_Enterobacteriaceae_Pseudescherichia_85
OTU_467	7.07E-01	4.60E-01	1.39E-04	41.70	19.34	Clostridium_perfringens_97	Bacteria_Firmicutes_Clostridia_97_Clostridiales_97_Clostridiaceae 1_97_Clostridium sensu stricto_67
OTU_415	8.29E-01	6.04E-01	6.35E-04	38.01	19.23	Pseudescherichia_vulneris_92	Bacteria_Proteobacteria_93_Gammaproteobacteria_93_Enterobacterales_93_Enterobacteriaceae_93_Pseudescherichia_91
OTU_336	9.49E-01	9.94E-01	3.52E-03	35.05	19.70	Blautia_coccoides_98	Bacteria_Firmicutes_Clostridia_Clostridiales_Lachnospiraceae_Blautia_89
OTU_191	7.07E-01	4.60E-01	9.82E-02	34.06	16.49	Collinsella_aerofaciens_99	Bacteria_Actinobacteria_69_Coriobacteriia_69_Coriobacteriales_69_Coriobacteriaceae_68_Collinsella_68
OTU_173	9.08E-01	8.14E-01	5.10E-03	49.14	2.11	Phascolarctobacterium_succinatutens_99	Bacteria_Firmicutes_Negativicutes_Acidaminococcales_Acidaminococcaceae_Phascolarctobacterium_100
OTU_205	8.82E-01	7.40E-01	8.06E-03	53.29	10.90	Sutterella_massiliensis_93	Bacteria_Proteobacteria_Gammaproteobacteria_54_Enterobacterales_54_Enterobacteriaceae_54_Pseudescherichia_51
OTU_1014	8.45E-01	6.04E-01	3.83E-04	38.65	12.35	Bacteroides_finegoldii_97	Bacteria_Bacteroidetes_Bacteroidia_Bacteroidales_Bacteroidaceae_Bacteroides_100
OTU_296	8.20E-01	6.04E-01	1.61E-02	40.53	13.33	Sutterella_wadsworthensis_92	Bacteria_Proteobacteria_Gammaproteobacteria_70_Enterobacterales_70_Enterobacteriaceae_70_Pseudescherichia_63
OTU_113566	7.07E-01	6.66E-01	4.02E-01	24.31	14.01	Veillonella_parvula_98	Bacteria_Firmicutes_Negativicutes_Veillonellales_Veillonellaceae_Veillonella_100
OTU_232	7.94E-01	3.49E-01	2.24E-01	31.49	24.22	Paraclostridium_benzoelyticum_97	Bacteria_Firmicutes_Clostridia_Clostridiales_Peptostreptococcaceae_Paraclostridium_97
OTU_1103	7.80E-01	9.83E-01	3.87E-01	30.45	26.05	Veillonella_parvula_98	Bacteria_Firmicutes_Negativicutes_Veillonellales_Veillonellaceae_Veillonella_100
OTU_1202	9.93E-01	7.40E-01	2.44E-01	21.70	26.75	Veillonella_tobetsuensis_89	Bacteria_Proteobacteria_41_Gammaproteobacteria_41_Enterobacterales_41_Enterobacteriaceae_41_Pseudescherichia_41
OTU_546	9.69E-01	9.62E-01	3.37E-05	16.77	30.75	Pseudescherichia_vulneris_94	Bacteria_Proteobacteria_98_Gammaproteobacteria_98_Enterobacterales_98_Enterobacteriaceae_96_Pseudescherichia_83
OTU_71	9.14E-01	6.62E-01	1.71E-02	74.99	2.23	Streptococcus_anginosus_100	Bacteria_Firmicutes_Bacilli_Lactobacillales_Streptococcaceae_Streptococcus_100
OTU_1504	9.93E-01	9.90E-01	2.61E-03	17.15	29.51	Enterocloster_citroniae_98	Bacteria_Firmicutes_Clostridia_Clostridiales_Lachnospiraceae_Enterocloster_97
OTU_356	9.38E-01	6.04E-01	5.79E-03	41.78	16.27	Clostridium_symbiosum_93	Bacteria_Firmicutes_Clostridia_Clostridiales_Lachnospiraceae_88_Clostridium XlVa_56
OTU_512	8.60E-01	9.61E-01	9.45E-01	30.80	30.45	Roseburia_inulinivorans_92	Bacteria_Firmicutes_62_Clostridia_62_Clostridiales_62_Lachnospiraceae_62_Roseburia_34
OTU_100	7.07E-01	6.10E-01	3.34E-02	24.49	6.58	Phocaeicola_massiliensis_100	Bacteria_Bacteroidetes_Bacteroidia_Bacteroidales_Bacteroidaceae_Phocaeicola_87
OTU_103	7.97E-01	8.83E-01	4.97E-02	13.12	8.47	Fusicatenibacter_saccharivorans_100	Bacteria_Firmicutes_Clostridia_Clostridiales_Lachnospiraceae_Fusicatenibacter_97
OTU_19986	8.61E-01	8.83E-01	7.14E-03	40.56	21.09	Bacteroides_xylanisolvens_98	Bacteria_Bacteroidetes_Bacteroidia_Bacteroidales_Bacteroidaceae_Bacteroides_100
OTU_43	8.61E-01	6.76E-01	7.37E-01	0.82	0.66	Asteroleplasma_anaerobium_79	Bacteria_Tenericutes_91_Mollicutes_91_Anaeroplasmatales_91_Anaeroplasmataceae_91_Asteroleplasma_91
OTU_215	7.70E-01	6.04E-01	4.10E-02	24.07	9.13	Bacteroides_fragilis_96	Bacteria_Bacteroidetes_Bacteroidia_Bacteroidales_Bacteroidaceae_Bacteroides_100
OTU_1220	9.78E-01	8.14E-01	2.43E-03	20.97	27.92	Hungatella_effluvii_97	Bacteria_Firmicutes_77_Clostridia_77_Clostridiales_77_Lachnospiraceae_77_Hungatella_73
OTU_196	9.66E-01	7.51E-01	6.69E-02	15.77	28.23	Sellimonas_intestinalis_100	Bacteria_Firmicutes_Clostridia_Clostridiales_Lachnospiraceae_Sellimonas_100
OTU_286	9.11E-01	7.73E-01	1.25E-01	20.08	24.51	Pseudescherichia_vulneris_97	Bacteria_Proteobacteria_Gammaproteobacteria_Enterobacterales_Enterobacteriaceae_Escherichia/Shigella_84

OTU_45	7.10E-01	6.53E-01	8.52E-01	9.83	10.58	Pseudoflavonifractor_phocaeensis_98	Bacteria_Firmicutes_Clostridia_Clostridiales_Ruminococcaceae_Lawsonibacter_96
OTU_68	9.61E-01	6.63E-01	9.27E-03	9.04	2.17	Parasutterella_excrementihominis_100	Bacteria_Proteobacteria_Betaproteobacteria_Burkholderiales_Sutterellaceae_Parasutterella_100
OTU_2483	7.91E-01	9.90E-01	2.48E-01	27.49	21.81	Blautia_hominis_99	Bacteria_Firmicutes_Clostridia_Clostridiales_Lachnospiraceae_Blautia_100
OTU_190	9.92E-01	9.53E-01	5.62E-03	8.34	35.83	Pseudescherichia_vulneris_99	Bacteria_Proteobacteria_99_Gammaproteobacteria_99_Enterobacterales_99_Enterobacteriaceae_99_Pseudescherichia_93
OTU_274	7.84E-01	8.73E-01	6.60E-03	4.97	43.32	Bacteroides_faecichinchillae_96	Bacteria_Bacteroidetes_Bacteroidia_Bacteroidales_Bacteroidaceae_Bacteroides_89
OTU_315	7.07E-01	6.04E-01	1.34E-04	43.73	9.16	Roseburia_inulinivorans_94	Bacteria_Firmicutes_Clostridia_Clostridiales_Lachnospiraceae_90_Roseburia_78
OTU_114	7.07E-01	9.62E-01	1.42E-01	8.05	2.83	Collinsella_aerofaciens_92	Bacteria_Actinobacteria_Coriobacteriia_97_Coriobacteriales_97_Coriobacteriaceae_97_Collinsella_96
OTU_1012	9.69E-01	6.63E-01	4.51E-01	25.18	19.26	Veillonella_parvula_91	Bacteria_Firmicutes_Negativicutes_72_Veillonellales_64_Veillonellaceae_64_Veillonella_64
OTU_110	8.97E-01	8.23E-01	4.37E-03	44.65	7.54	Clostridium_viride_96	Bacteria_Firmicutes_Clostridia_Clostridiales_Ruminococcaceae_Intestinimonas_57
OTU_1334	8.05E-01	7.75E-01	8.71E-02	22.60	15.39	Veillonella_atypica_97	Bacteria_Firmicutes_Negativicutes_Veillonellales_Veillonellaceae_Veillonella_100
OTU_21213	7.10E-01	6.04E-01	5.74E-08	40.01	8.35	Bacteroides_faecis_97	Bacteria_Bacteroidetes_Bacteroidia_Bacteroidales_Bacteroidaceae_Bacteroides_100
OTU_1362	7.57E-01	6.04E-01	1.68E-03	29.35	14.52	Clostridium_perfringens_97	Bacteria_Firmicutes_Clostridia_99_Clostridiales_99_Clostridiaceae 1_98_Clostridium sensu stricto_83
OTU_1533	9.98E-01	8.83E-01	3.33E-02	16.26	22.84	Shigella_dysenteriae_95	Bacteria_Proteobacteria_Gammaproteobacteria_Enterobacterales_Enterobacteriaceae_Pseudescherichia_60
OTU_342	9.31E-01	9.37E-01	5.52E-03	13.70	19.52	Pseudescherichia_vulneris_98	Bacteria_Proteobacteria_86_Gammaproteobacteria_86_Enterobacterales_86_Enterobacteriaceae_86_Pseudescherichia_63
OTU_157	9.78E-01	9.63E-01	1.80E-02	15.86	24.97	Lachnoclostridium_pacaense_97	Bacteria_Firmicutes_Clostridia_Clostridiales_Lachnospiraceae_Clostridium XlVa_44
OTU_304	9.54E-01	6.04E-01	2.48E-08	38.18	3.64	Parabacteroides_distasonis_94	Bacteria_Bacteroidetes_Bacteroidia_Bacteroidales_Porphyromonadaceae_93_Parabacteroides_93
OTU_575	7.57E-01	7.01E-01	8.25E-06	9.46	21.55	Enterococcus_faecalis_90	Bacteria_Firmicutes_Clostridia_92_Clostridiales_92_Lachnospiraceae_92_Enterocloster_87
OTU_107	8.06E-01	6.04E-01	7.58E-02	31.20	5.58	Limosilactobacillus_reuteri_100	Bacteria_Firmicutes_Bacilli_Lactobacillales_Lactobacillaceae_Limosilactobacillus_100
OTU_27286	9.92E-01	8.35E-01	1.83E-03	17.89	32.88	Bacteroides_thetaiotaomicron_97	Bacteria_Bacteroidetes_Bacteroidia_Bacteroidales_Bacteroidaceae_Bacteroides_100
OTU_154	8.31E-01	8.73E-01	6.32E-03	19.27	4.60	Parabacteroides_merdae_100	Bacteria_Bacteroidetes_Bacteroidia_Bacteroidales_Porphyromonadaceae_Parabacteroides_100
OTU_251	9.37E-01	7.31E-01	1.84E-03	6.81	19.01	Phocaeicola_coprophilus_97	Bacteria_Bacteroidetes_Bacteroidia_Bacteroidales_Bacteroidaceae_Phocaeicola_97
OTU_275	8.82E-01	9.97E-01	1.49E-02	14.50	27.59	Paraclostridium_benzoelyticum_97	Bacteria_Firmicutes_Clostridia_Clostridiales_Peptostreptococcaceae_Paraclostridium_96
OTU_384	9.06E-01	7.42E-01	9.08E-01	22.14	21.55	Coprococcus_comes_97	Bacteria_Firmicutes_Clostridia_Clostridiales_Lachnospiraceae_Bariatricus_76
OTU_113	9.56E-01	9.37E-01	1.11E-04	43.01	4.44	Oscillibacter_ruminantium_95	Bacteria_Firmicutes_Clostridia_Clostridiales_Ruminococcaceae_Dysosmobacter_51
OTU_1000	8.06E-01	6.04E-01	1.35E-03	13.65	21.18	Enterococcus_faecalis_98	Bacteria_Firmicutes_Bacilli_Lactobacillales_95_Enterococcaceae_67_Enterococcus_66
OTU_831	9.38E-01	7.20E-01	8.64E-01	16.75	17.10	Hungatella_hathewayi_94	Bacteria_Firmicutes_Clostridia_93_Clostridiales_93_Lachnospiraceae_93_Hungatella_33
OTU_92	7.07E-01	7.23E-01	1.59E-01	34.27	12.28	Negativicoccus_succinicivorans_100	Bacteria_Firmicutes_Negativicutes_Veillonellales_Veillonellaceae_Negativicoccus_100
OTU_5293	9.87E-01	6.05E-01	5.39E-02	37.08	6.77	Romboutsia_timonensis_99	Bacteria_Firmicutes_Clostridia_Clostridiales_Peptostreptococcaceae_Romboutsia_100
OTU_10419	8.57E-01	9.46E-01	4.45E-01	19.19	21.50	Pseudescherichia_vulneris_96	Bacteria_Proteobacteria_89_Gammaproteobacteria_89_Enterobacterales_87_Enterobacteriaceae_87_Pseudescherichia_64
OTU_104	8.85E-01	6.83E-01	9.88E-01	5.53	5.56	Eubacterium_ramulus_98	Bacteria_Firmicutes_Clostridia_Clostridiales_Lachnospiraceae_Lachnospiracea_incertae_sedis_92
OTU_852	9.38E-01	9.77E-01	1.09E-05	34.14	4.34	Anaerotignum_aminivorans_96	Bacteria_Firmicutes_Clostridia_Clostridiales_Lachnospiraceae_99_Anaerotignum_98
OTU_668	9.78E-01	7.73E-01	1.65E-04	18.73	8.87	Faecalimonas_umbilicata_97	Bacteria_Firmicutes_Clostridia_Clostridiales_Lachnospiraceae_Clostridium XlVa_71
OTU_155	7.07E-01	6.04E-01	1.20E-01	7.19	2.56	Bifidobacterium_callitrichidarum_94	Bacteria_Actinobacteria_Actinobacteria_99_Bifidobacteriales_99_Bifidobacteriaceae_99_Bifidobacterium_50
OTU_142	7.07E-01	9.75E-01	2.04E-02	21.79	5.42	Ruminococcus_faecis_100	Bacteria_Firmicutes_Clostridia_Clostridiales_Lachnospiraceae_Mediterraneibacter_100
OTU_407	9.14E-01	8.60E-01	7.60E-02	24.63	15.49	Roseburia_inulinivorans_93	Bacteria_Firmicutes_Clostridia_92_Clostridiales_92_Lachnospiraceae_92_Roseburia_81
OTU_177	7.43E-01	9.35E-01	4.97E-02	12.64	5.69	Eubacterium_rectale_100	Bacteria_Firmicutes_Clostridia_Clostridiales_Lachnospiraceae_Agathobacter_95
OTU_21921	8.42E-01	6.83E-01	1.03E-02	16.49	27.52	Bacteroides_thetaiotaomicron_98	Bacteria_Bacteroidetes_Bacteroidia_Bacteroidales_Bacteroidaceae_Bacteroides_100
OTU_992	9.90E-01	6.82E-01	7.60E-01	7.63	8.43	Paeniclostridium_sordellii_98	Bacteria_Firmicutes_Clostridia_Clostridiales_Peptostreptococcaceae_Paeniclostridium_100
OTU_339	7.07E-01	6.36E-01	1.03E-02	11.32	3.67	Bifidobacterium_stercoris_99	Bacteria_Actinobacteria_Actinobacteria_Bifidobacteriales_Bifidobacteriaceae_Bifidobacterium_100
OTU_678	9.64E-01	8.36E-01	6.57E-02	14.95	25.01	Bacteroides_faecis_97	Bacteria_Bacteroidetes_Bacteroidia_Bacteroidales_Bacteroidaceae_Bacteroides_100
OTU_260	9.37E-01	6.04E-01	1.91E-01	11.33	19.91	Erysipelatoclostridium_ramosum_92	Bacteria_Firmicutes_Erysipelotrichia_Erysipelotrichales_Erysipelatoclostridiaceae_Erysipelatoclostridium_100
OTU_664	7.45E-01	2.14E-01	9.27E-03	23.49	12.10	Bacteroides_thetaiotaomicron_100	Bacteria_Bacteroidetes_97_Bacteroidia_97_Bacteroidales_97_Bacteroidaceae_96_Bacteroides_73
OTU_102	7.07E-01	6.04E-01	6.55E-02	28.97	6.04	Flintibacter_butyricus_97	Bacteria_Firmicutes_Clostridia_Clostridiales_Ruminococcaceae_Flintibacter_97
OTU_587	7.07E-01	6.62E-01	2.29E-05	28.70	6.16	Coprococcus_comes_94	Bacteria_Firmicutes_Clostridia_Clostridiales_Lachnospiraceae_99_Coprococcus_53
OTU_516	1.00E+00	9.13E-01	2.84E-04	7.44	21.74	Enterocloster_citroniae_98	Bacteria_Firmicutes_Clostridia_95_Clostridiales_95_Lachnospiraceae_95_Enterocloster_78
OTU_2157	7.07E-01	6.04E-01	3.04E-01	19.35	2.83	Veillonella_rogosae_98	Bacteria_Firmicutes_Negativicutes_Veillonellales_Veillonellaceae_Veillonella_100
OTU_199	9.60E-01	6.04E-01	1.77E-01	10.83	19.27	Melissococcus_plutonius_92	Bacteria_Firmicutes_Erysipelotrichia_72_Erysipelotrichales_72_Erysipelatoclostridiaceae_72_Erysipelatoclostridium_72
OTU_138	7.07E-01	9.46E-01	8.07E-04	30.58	2.18	Dialister_pneumosintes_100	Bacteria_Firmicutes_Negativicutes_Veillonellales_Veillonellaceae_Dialister_100
OTU_91	7.10E-01	6.04E-01	1.97E-01	33.79	11.18	Bifidobacterium_breve_100	Bacteria_Actinobacteria_Actinobacteria_Bifidobacteriales_Bifidobacteriaceae_Bifidobacterium_100
OTU_77	8.97E-01	6.04E-01	6.29E-02	37.99	4.23	Anaerococcus_vaginalis_100	Bacteria_Firmicutes_Clostridia_Clostridiales_Peptoniphilaceae_Anaerococcus_100
OTU_84	9.93E-01	6.04E-01	3.74E-02	34.63	3.66	Ruminococcus_bromii_97	Bacteria_Firmicutes_Clostridia_Clostridiales_Ruminococcaceae_Ruminococcus_52
OTU_543	9.65E-01	7.96E-01	4.49E-04	8.84	22.86	Veillonella_dispar_96	Bacteria_Firmicutes_Negativicutes_Veillonellales_Veillonellaceae_Veillonella_100
OTU_932	7.07E-01	9.90E-01	6.08E-04	21.82	5.85	Bacteroides_kribbi_96	Bacteria_Bacteroidetes_Bacteroidia_Bacteroidales_Bacteroidaceae_Bacteroides_97
OTU_139	7.07E-01	6.04E-01	8.52E-02	12.74	23.07	Oscillibacter_ruminantium_96	Bacteria_Firmicutes_Clostridia_Clostridiales_Ruminococcaceae_Dysosmobacter_100
OTU_1117	7.57E-01	4.60E-01	9.16E-02	17.00	13.02	Enterococcus_faecalis_97	Bacteria_Firmicutes_Bacilli_Lactobacillales_Enterococcaceae_82_Enterococcus_58
OTU_1285	9.38E-01	8.68E-01	8.29E-01	12.22	12.74	Enterococcus_faecalis_99	Bacteria_Firmicutes_Bacilli_Lactobacillales_Enterococcaceae_99_Enterococcus_92
OTU_2902	7.07E-01	6.63E-01	4.57E-01	16.66	15.43	Enterocloster_lavalensis_99	Bacteria_Firmicutes_Clostridia_Clostridiales_Lachnospiraceae_Enterocloster_100
OTU_360	7.46E-01	7.80E-01	3.50E-04	25.83	2.86	Enterococcus_pallens_96	Bacteria_Firmicutes_Bacilli_Lactobacillales_Enterococcaceae_53_Melissococcus_23
OTU_402	9.87E-01	6.04E-01	4.85E-04	24.34	7.80	Pseudescherichia_vulneris_97	Bacteria_Proteobacteria_Gammaproteobacteria_Enterobacterales_Enterobacteriaceae_Escherichia/Shigella_83
OTU_3801	9.04E-01	7.51E-01	1.60E-02	12.10	15.64	Pseudescherichia_vulneris_95	Bacteria_Proteobacteria_Gammaproteobacteria_Enterobacterales_Enterobacteriaceae_Pseudescherichia_57
OTU_5254	9.67E-01	9.34E-01	2.98E-05	22.32	3.94	Bacteroides_caccae_98	Bacteria_Bacteroidetes_Bacteroidia_Bacteroidales_Bacteroidaceae_Bacteroides_100
OTU_1026	8.24E-01	6.04E-01	1.16E-03	24.65	9.28	Clostridium_perfringens_97	Bacteria_Firmicutes_Clostridia_Clostridiales_Clostridiaceae 1_75_Clostridium sensu stricto_49
OTU_182	7.07E-01	8.97E-01	6.52E-02	6.81	18.76	Pantoea_stewartii_97	Bacteria_Proteobacteria_Gammaproteobacteria_Enterobacterales_Enterobacteriaceae_67_Escherichia/Shigella_37
OTU_1113	7.85E-01	7.25E-01	8.23E-01	15.80	16.16	Pseudescherichia_vulneris_97	Bacteria_Proteobacteria_Gammaproteobacteria_Enterobacterales_Enterobacteriaceae_Escherichia/Shigella_97
OTU_413	9.05E-01	5.62E-01	8.45E-02	9.97	16.35	Pseudescherichia_vulneris_96	Bacteria_Proteobacteria_Gammaproteobacteria_Enterobacterales_Enterobacteriaceae_Escherichia/Shigella_81
OTU_1311	7.45E-01	8.89E-01	2.05E-01	35.61	5.00	Streptococcus_alactolyticus_100	Bacteria_Firmicutes_Bacilli_Lactobacillales_Streptococcaceae_Streptococcus_100
OTU_527	9.78E-01	6.04E-01	4.47E-01	5.53	11.02	Bifidobacterium_bifidum_100	Bacteria_Actinobacteria_Actinobacteria_Bifidobacteriales_Bifidobacteriaceae_Bifidobacterium_100

OTU_249	7.07E-01	8.14E-01	3.61E-03	13.72	4.79	Phocaeicola_vulgatus_99	Bacteria_Bacteroidetes_89_Bacteroidia_89_Bacteroidales_89_Bacteroidaceae_89_Phocaeicola_78
OTU_3348	8.70E-01	6.47E-01	5.74E-01	14.02	14.97	Escherichia_coli_95	Bacteria_Proteobacteria_Gammaproteobacteria_Enterobacterales_Enterobacteriaceae_Pseudescherichia_73
OTU_8213	1.00E+00	8.14E-01	2.27E-02	13.37	21.50	Bacteroides_xylanisolvens_98	Bacteria_Bacteroidetes_Bacteroidia_Bacteroidales_Bacteroidaceae_Bacteroides_100
OTU_121	9.45E-01	6.06E-01	4.53E-02	4.23	19.87	Morganella_morganii_99	Bacteria_Proteobacteria_Gammaproteobacteria_Enterobacterales_Morganellaceae_Morganella_100
OTU_758	9.37E-01	8.79E-01	7.70E-02	10.18	12.92	Flavonifractor_plautii_93	Bacteria_Firmicutes_Clostridia_Clostridiales_Ruminococcaceae_89_Flavonifractor_87
OTU_117242	7.07E-01	7.01E-01	1.64E-01	3.04	8.99	Veillonella_parvula_98	Bacteria_Firmicutes_Negativicutes_Veillonellales_Veillonellaceae_Veillonella_100
OTU_631	9.71E-01	6.99E-01	1.69E-01	12.98	18.03	Kineothrix_alysoides_96	Bacteria_Firmicutes_87_Clostridia_87_Clostridiales_87_Lachnospiraceae_87_Merdimonas_36
OTU_129	9.43E-01	6.04E-01	2.79E-01	12.93	8.30	Clostridium_hylemonae_98	Bacteria_Firmicutes_Clostridia_Clostridiales_Lachnospiraceae_Clostridium XlVa_82
OTU_108	9.52E-01	6.04E-01	1.27E-01	26.48	7.36	Faecalibaculum_rodentium_100	Bacteria_Firmicutes_Erysipelotrichia_Erysipelotrichales_Erysipelotrichaceae_Faecalibaculum_100
OTU_28838	9.39E-01	8.83E-01	6.15E-01	9.82	12.08	Lacrimispora_amygdalina_97	Bacteria_Firmicutes_Clostridia_Clostridiales_Lachnospiraceae_Enterocloster_46
OTU_956	8.97E-01	9.90E-01	2.92E-05	25.57	2.56	Bacteroides_finegoldii_96	Bacteria_Bacteroidetes_Bacteroidia_Bacteroidales_Bacteroidaceae_Bacteroides_94
OTU_88	7.07E-01	6.61E-01	2.50E-01	7.45	5.48	Oscillibacter_ruminantium_95	Bacteria_Firmicutes_Clostridia_Clostridiales_Ruminococcaceae_Oscillibacter_55
OTU_391	7.45E-01	6.04E-01	3.82E-03	21.42	4.82	Clostridium_perfringens_95	Bacteria_Firmicutes_Clostridia_Clostridiales_Clostridiaceae 1_93_Sarcina_49
OTU_13500	9.75E-01	7.77E-01	3.23E-01	13.49	14.80	Pseudescherichia_vulneris_96	Bacteria_Proteobacteria_Gammaproteobacteria_Enterobacterales_Enterobacteriaceae_Pseudescherichia_54
OTU_105	7.91E-01	9.62E-01	5.76E-01	5.10	4.51	Gemmiger_formicilis_97	Bacteria_Firmicutes_Clostridia_Clostridiales_Ruminococcaceae_Gemmiger_83
OTU_3011	7.74E-01	6.04E-01	5.74E-08	21.49	4.41	Phocaeicola_dorei_96	Bacteria_Bacteroidetes_Bacteroidia_Bacteroidales_Bacteroidaceae_Bacteroides_73
OTU_350	9.43E-01	6.77E-01	3.37E-04	5.91	20.70	Bacteroides_fragilis_100	Bacteria_Bacteroidetes_84_Bacteroidia_84_Bacteroidales_84_Bacteroidaceae_81_Mediterranea_55
OTU_935	9.61E-01	6.04E-01	1.14E-02	20.97	9.49	Kineothrix_alysoides_96	Bacteria_Firmicutes_Clostridia_Clostridiales_Lachnospiraceae_Kineothrix_56
OTU_14935	7.43E-01	9.61E-01	5.29E-02	23.31	5.09	Veillonella_parvula_98	Bacteria_Firmicutes_Negativicutes_Veillonellales_Veillonellaceae_Veillonella_100
OTU_180	7.07E-01	7.97E-01	6.10E-03	8.24	2.37	Bifidobacterium_callitrichidarum_92	Bacteria_Actinobacteria_90_Actinobacteria_90_Bifidobacteriales_90_Bifidobacteriaceae_90_Pseudoscardovia_21
OTU_297	9.38E-01	6.04E-01	4.67E-05	20.92	2.67	Bacteroides_fragilis_96	Bacteria_Bacteroidetes_Bacteroidia_Bacteroidales_Bacteroidaceae_Bacteroides_100
OTU_3533	8.33E-01	8.71E-01	5.85E-01	14.07	13.29	Falcatimonas_natans_90	Bacteria_Firmicutes_54_Clostridia_54_Clostridiales_54_Lachnospiraceae_54_Falcatimonas_22
OTU_90	1.00E+00	6.83E-01	7.18E-02	26.97	3.14	Ligilactobacillus_apodemi_100	Bacteria_Firmicutes_Bacilli_Lactobacillales_Lactobacillaceae_Ligilactobacillus_100
OTU_34575	8.83E-01	9.46E-01	3.04E-02	11.71	14.75	Pseudescherichia_vulneris_97	Bacteria_Proteobacteria_Gammaproteobacteria_Enterobacterales_Enterobacteriaceae_Escherichia/Shigella_96
OTU_106	9.08E-01	9.42E-01	9.37E-02	12.13	4.38	Peptoniphilus_duerdenii_100	Bacteria_Firmicutes_Clostridia_Clostridiales_Peptoniphilaceae_Peptoniphilus_100
OTU_456	9.78E-01	6.04E-01	1.79E-01	9.24	14.20	Enterococcus_faecalis_90	Bacteria_Firmicutes_Clostridia_76_Clostridiales_76_Lachnospiraceae_76_Clostridium XlVa_18
OTU_471	8.57E-01	6.04E-01	2.08E-02	9.49	16.69	Romboutsia_timonensis_92	Bacteria_Firmicutes_Bacilli_81_Lactobacillales_46_Carnobacteriaceae_30_Isobaculum_29
OTU_147	7.07E-01	9.98E-01	2.45E-02	26.97	3.33	Bacteroides_stercoris_99	Bacteria_Bacteroidetes_74_Bacteroidia_74_Bacteroidales_74_Bacteroidaceae_73_Mediterranea_43
OTU_4588	9.69E-01	6.04E-01	7.27E-03	9.49	13.44	Enterococcus_faecalis_92	Bacteria_Firmicutes_62_Bacilli_62_Lactobacillales_60_Carnobacteriaceae_51_Catellicoccus_34
OTU_12075	9.04E-01	6.05E-01	2.57E-01	14.87	18.38	Bacteroides_thetaiotaomicron_98	Bacteria_Bacteroidetes_Bacteroidia_Bacteroidales_Bacteroidaceae_Bacteroides_100
OTU_451	7.07E-01	6.04E-01	1.09E-05	22.38	5.86	Bacteroides_uniformis_99	Bacteria_Bacteroidetes_74_Bacteroidia_74_Bacteroidales_74_Bacteroidaceae_73_Mediterranea_42
OTU_1018	8.38E-01	9.27E-01	2.45E-01	12.37	15.54	Paraclostridium_benzoelyticum_92	Bacteria_Firmicutes_73_Clostridia_73_Clostridiales_73_Peptostreptococcaceae_73_Paraclostridium_51
OTU_219	9.38E-01	6.76E-01	2.43E-03	3.41	11.11	Pseudescherichia_vulneris_99	Bacteria_Proteobacteria_93_Gammaproteobacteria_93_Enterobacterales_93_Enterobacteriaceae_93_Pseudescherichia_83
OTU_2465	7.22E-01	9.10E-01	1.33E-01	14.12	10.14	Clostridium_symbiosum_96	Bacteria_Firmicutes_Clostridia_Clostridiales_Lachnospiraceae_Clostridium XlVa_36
OTU_3104	7.46E-01	6.04E-01	6.35E-05	25.28	7.40	Bacteroides_uniformis_97	Bacteria_Bacteroidetes_Bacteroidia_Bacteroidales_Bacteroidaceae_Bacteroides_100
OTU_571	8.61E-01	9.69E-01	9.61E-01	15.44	15.29	Shigella_dysenteriae_93	Bacteria_Proteobacteria_95_Gammaproteobacteria_95_Enterobacterales_95_Enterobacteriaceae_95_Pseudescherichia_93
OTU_733	7.57E-01	6.04E-01	1.36E-01	9.45	17.04	Erysipelatoclostridium_ramosum_99	Bacteria_Firmicutes_93_Erysipelotrichia_93_Erysipelotrichales_93_Erysipelatoclostridiaceae_93_Erysipelatoclostridium_93
OTU_2168	9.43E-01	9.06E-01	4.12E-01	16.25	13.68	Roseburia_inulinivorans_97	Bacteria_Firmicutes_Clostridia_Clostridiales_Lachnospiraceae_Coprococcus_20
OTU_335	9.32E-01	6.04E-01	7.33E-03	21.55	6.01	Clostridium_perfringens_98	Bacteria_Firmicutes_Clostridia_91_Clostridiales_91_Clostridiaceae 1_91_Anaerobacter_28
OTU_325	7.50E-01	6.04E-01	3.01E-04	20.34	2.03	Streptococcus_gordonii_95	Bacteria_Firmicutes_Bacilli_Lactobacillales_Streptococcaceae_89_Streptococcus_89
OTU_112	7.16E-01	9.41E-01	1.01E-01	18.88	4.01	Clostridium_moniliforme_100	Bacteria_Firmicutes_Clostridia_Clostridiales_Clostridiaceae 1_Clostridium sensu stricto_100
OTU_240	7.46E-01	6.72E-01	3.22E-02	11.20	3.97	Kineothrix_alysoides_91	Bacteria_Firmicutes_Clostridia_Clostridiales_Lachnospiraceae_Merdimonas_15
OTU_772	1.00E+00	9.69E-01	1.08E-01	17.50	11.15	Roseburia_inulinivorans_97	Bacteria_Firmicutes_Clostridia_Clostridiales_Lachnospiraceae_Ruminococcus2_42
OTU_406	8.25E-01	9.75E-01	6.79E-02	9.58	15.56	Enterocloster_citroniae_95	Bacteria_Firmicutes_Clostridia_Clostridiales_Lachnospiraceae_96_Enterocloster_96
OTU_915	7.57E-01	8.65E-01	6.66E-04	17.62	2.96	Serratia_marcescens_88	Bacteria_Proteobacteria_94_Gammaproteobacteria_94_Enterobacterales_93_Enterobacteriaceae_93_Pseudescherichia_93
OTU_276	7.07E-01	9.58E-01	6.66E-04	9.24	1.90	Phocaeicola_vulgatus_95	Bacteria_Bacteroidetes_Bacteroidia_Bacteroidales_Bacteroidaceae_Phocaeicola_97
OTU_327	7.59E-01	6.04E-01	2.85E-04	7.18	21.44	Bacteroides_faecis_95	Bacteria_Bacteroidetes_Bacteroidia_Bacteroidales_Bacteroidaceae_Bacteroides_96
OTU_536	8.85E-01	8.83E-01	8.24E-03	7.76	11.05	Flavonifractor_plautii_98	Bacteria_Firmicutes_93_Clostridia_93_Clostridiales_93_Ruminococcaceae_93_Flavonifractor_92
OTU_871	9.92E-01	7.74E-01	1.19E-02	10.68	17.57	Bacteroides_thetaiotaomicron_97	Bacteria_Bacteroidetes_68_Bacteroidia_68_Bacteroidales_68_Bacteroidaceae_68_Bacteroides_54
OTU_146	8.29E-01	8.65E-01	2.64E-03	16.12	3.82	Peptoniphilus_koenoeneniae_100	Bacteria_Firmicutes_Clostridia_Clostridiales_Peptoniphilaceae_Peptoniphilus_100
OTU_669	7.57E-01	6.04E-01	9.84E-02	7.62	4.74	Blautia_wexlerae_98	Bacteria_Firmicutes_Clostridia_Clostridiales_Lachnospiraceae_Blautia_95
OTU_489	9.08E-01	7.40E-01	1.84E-01	12.21	9.72	Pseudescherichia_vulneris_97	Bacteria_Proteobacteria_Gammaproteobacteria_Enterobacterales_Enterobacteriaceae_Escherichia/Shigella_95
OTU_1251	9.87E-01	7.77E-01	2.26E-02	21.11	6.92	Romboutsia_timonensis_98	Bacteria_Firmicutes_Clostridia_Clostridiales_Peptostreptococcaceae_Romboutsia_97
OTU_267	9.87E-01	6.04E-01	2.51E-01	9.23	16.53	Clostridium_symbiosum_94	Bacteria_Firmicutes_Clostridia_Clostridiales_Lachnospiraceae_92_Clostridium XlVa_74
OTU_660	7.07E-01	6.04E-01	9.95E-01	9.10	9.06	Kluyvera_intermedia_98	Bacteria_Proteobacteria_Gammaproteobacteria_Enterobacterales_Enterobacteriaceae_Kluyvera_31
OTU_4399	7.07E-01	6.04E-01	4.15E-04	18.26	6.87	Clostridium_perfringens_97	Bacteria_Firmicutes_Clostridia_99_Clostridiales_99_Clostridiaceae 1_84_Sarcina_33
OTU_221	8.25E-01	9.62E-01	7.01E-02	2.86	1.28	Phocaeicola_coprophilus_96	Bacteria_Bacteroidetes_Bacteroidia_Bacteroidales_Bacteroidaceae_Phocaeicola_100
OTU_8880	9.38E-01	6.04E-01	1.49E-02	9.30	13.02	Enterococcus_faecalis_97	Bacteria_Firmicutes_Bacilli_Lactobacillales_Enterococcaceae_89_Enterococcus_76
OTU_1389	9.38E-01	9.69E-01	5.93E-01	10.93	11.73	Shigella_dysenteriae_97	Bacteria_Proteobacteria_Gammaproteobacteria_Enterobacterales_Enterobacteriaceae_Escherichia/Shigella_76
OTU_8403	8.57E-01	6.63E-01	6.79E-02	9.58	12.65	Enterococcus_faecalis_98	Bacteria_Firmicutes_Bacilli_Lactobacillales_Enterococcaceae_94_Enterococcus_91
OTU_461	9.17E-01	8.79E-01	3.75E-04	4.63	21.30	Bacteroides_thetaiotaomicron_100	Bacteria_Bacteroidetes_99_Bacteroidia_99_Bacteroidales_99_Bacteroidaceae_96_Mediterranea_50
OTU_2163	9.14E-01	9.34E-01	3.93E-02	17.81	9.12	Roseburia_inulinivorans_97	Bacteria_Firmicutes_Clostridia_Clostridiales_Lachnospiraceae_Roseburia_90
OTU_101	7.07E-01	7.25E-01	1.05E-01	0.09	21.68	Rhizobacter_profundi_98	Bacteria_Proteobacteria_Betaproteobacteria_Burkholderiales_Comamonadaceae_95_Aquincola_54
OTU_4810	8.33E-01	7.45E-01	5.40E-06	13.34	2.14	Phocaeicola_vulgatus_97	Bacteria_Bacteroidetes_Bacteroidia_Bacteroidales_Bacteroidaceae_Phocaeicola_86
OTU_89	7.59E-01	6.04E-01	1.80E-01	22.93	1.40	Muribaculum_intestinale_91	Bacteria_Bacteroidetes_Bacteroidia_Bacteroidales_Muribaculaceae_Duncaniella_49
OTU_545	7.07E-01	6.04E-01	2.50E-01	8.76	12.85	Erysipelatoclostridium_ramosum_98	Bacteria_Firmicutes_Erysipelotrichia_93_Erysipelotrichales_93_Erysipelatoclostridiaceae_91_Erysipelatoclostridium_91
OTU_473	7.57E-01	6.07E-01	9.40E-03	15.82	7.24	Peptoniphilus_tyrrelliae_95	Bacteria_Firmicutes_Clostridia_Clostridiales_Peptoniphilaceae_94_Peptoniphilus_94

OTU_289	9.45E-01	6.04E-01	9.32E-01	12.90	13.25	Murimonas_intestini_98	Bacteria_Firmicutes_Clostridia_Clostridiales_Lachnospiraceae_Murimonas_49
OTU_389	7.07E-01	5.62E-01	2.88E-01	11.63	6.77	Collinsella_aerofaciens_96	Bacteria_Actinobacteria_Coriobacteriia_Coriobacteriales_99_Coriobacteriaceae_99_Collinsella_99
OTU_2183	7.11E-01	9.27E-01	3.55E-04	14.26	6.57	Pseudescherichia_vulneris_96	Bacteria_Proteobacteria_Gammaproteobacteria_Enterobacterales_Enterobacteriaceae_Pseudescherichia_48
OTU_430	8.25E-01	6.62E-01	6.33E-04	9.10	2.07	Parabacteroides_distasonis_94	Bacteria_Bacteroidetes_Bacteroidia_Bacteroidales_Porphyromonadaceae_64_Parabacteroides_64
OTU_416	9.04E-01	6.04E-01	2.57E-01	11.16	8.68	Pseudescherichia_vulneris_98	Bacteria_Proteobacteria_98_Gammaproteobacteria_98_Enterobacterales_98_Enterobacteriaceae_97_Pseudescherichia_75
OTU_97	7.46E-01	6.04E-01	3.79E-01	17.86	5.51	Kineothrix_alysoides_98	Bacteria_Firmicutes_Clostridia_Clostridiales_Lachnospiraceae_Kineothrix_63
OTU_3549	9.31E-01	9.59E-01	1.75E-02	8.22	10.95	Pseudescherichia_vulneris_97	Bacteria_Proteobacteria_Gammaproteobacteria_Enterobacterales_Enterobacteriaceae_Escherichia/Shigella_75
OTU_517	9.61E-01	8.22E-01	4.84E-01	8.76	10.72	Veillonella_tobetsuensis_90	Bacteria_Firmicutes_Clostridia_94_Clostridiales_94_Lachnospiraceae_93_Faecalimonas_18
OTU_563	7.07E-01	6.47E-01	4.03E-03	16.45	3.94	Peptoniphilus_tyrrelliae_95	Bacteria_Firmicutes_Clostridia_Clostridiales_Peptoniphilaceae_Peptoniphilus_100
OTU_2061	9.78E-01	6.10E-01	9.58E-04	14.17	5.49	Enterococcus_faecalis_98	Bacteria_Firmicutes_Bacilli_Lactobacillales_Enterococcaceae_97_Enterococcus_92
OTU_854	9.46E-01	9.79E-01	2.46E-01	10.48	11.85	Escherichia_coli_89	Bacteria_Proteobacteria_78_Gammaproteobacteria_78_Enterobacterales_78_Enterobacteriaceae_78_Pseudescherichia_78
OTU_2185	8.73E-01	6.62E-01	3.26E-03	8.96	12.93	Hungatella_effluvii_97	Bacteria_Firmicutes_Clostridia_Clostridiales_Lachnospiraceae_Hungatella_99
OTU_4694	8.16E-01	9.62E-01	1.10E-01	14.20	10.47	Enterocloster_clostridioformis_97	Bacteria_Firmicutes_Clostridia_Clostridiales_Lachnospiraceae_Enterocloster_39
OTU_773	9.99E-01	7.55E-01	3.80E-01	9.69	11.25	Hungatella_effluvii_97	Bacteria_Firmicutes_Clostridia_Clostridiales_Lachnospiraceae_Hungatella_98
OTU_278	9.67E-01	6.04E-01	9.88E-02	4.56	18.02	Erysipelatoclostridium_ramosum_91	Bacteria_Firmicutes_Erysipelotrichia_Erysipelotrichales_Erysipelatoclostridiaceae_Erysipelatoclostridium_100
OTU_434	7.46E-01	6.04E-01	1.83E-03	10.84	3.42	Pseudescherichia_vulneris_99	Bacteria_Proteobacteria_95_Gammaproteobacteria_95_Enterobacterales_95_Enterobacteriaceae_95_Pseudescherichia_87
OTU_588	8.06E-01	9.46E-01	5.32E-03	7.31	12.38	Pseudescherichia_vulneris_96	Bacteria_Proteobacteria_Gammaproteobacteria_Enterobacterales_Enterobacteriaceae_Escherichia/Shigella_64
OTU_1338	8.85E-01	7.11E-01	4.94E-01	3.59	4.96	Faecalibacterium_prausnitzii_98	Bacteria_Firmicutes_Clostridia_Clostridiales_Ruminococcaceae_Faecalibacterium_100
OTU_247	7.07E-01	6.04E-01	7.71E-04	9.47	3.81	Flavonifractor_plautii_93	Bacteria_Firmicutes_Clostridia_Clostridiales_Ruminococcaceae_99_Flavonifractor_98
OTU_437	7.31E-01	6.50E-01	9.47E-01	7.73	7.65	Enterococcus_dispar_98	Bacteria_Firmicutes_Bacilli_67_Lactobacillales_65_Carnobacteriaceae_35_Isobaculum_35
OTU_518	8.27E-01	9.34E-01	9.72E-01	12.50	12.36	Veillonella_tobetsuensis_91	Bacteria_Firmicutes_Clostridia_87_Clostridiales_87_Lachnospiraceae_87_Roseburia_55
OTU_52067	8.97E-01	6.04E-01	5.35E-02	5.45	9.43	Enterococcus_faecalis_98	Bacteria_Firmicutes_Bacilli_Lactobacillales_Enterococcaceae_98_Enterococcus_93
OTU_93	8.48E-01	9.45E-01	2.77E-01	2.86	6.23	Fournierella_massiliensis_96	Bacteria_Firmicutes_Clostridia_Clostridiales_Ruminococcaceae_Fournierella_74
OTU_957	7.07E-01	6.04E-01	5.66E-05	18.68	5.30	Roseburia_inulinivorans_96	Bacteria_Firmicutes_Clostridia_Clostridiales_Lachnospiraceae_Roseburia_91
OTU_1900	9.92E-01	6.81E-01	9.41E-01	9.34	9.47	Clostridium_symbiosum_96	Bacteria_Firmicutes_Clostridia_Clostridiales_Lachnospiraceae_Clostridium XlVa_38
OTU_780	7.07E-01	7.73E-01	3.93E-06	17.87	1.18	Bacteroides_uniformis_95	Bacteria_Bacteroidetes_Bacteroidia_Bacteroidales_Bacteroidaceae_99_Bacteroides_96
OTU_316	8.49E-01	4.60E-01	2.05E-03	9.27	4.09	Clostridium_perfringens_93	Bacteria_Firmicutes_Clostridia_Clostridiales_Clostridiaceae 1_61_Sarcina_30
OTU_501	7.12E-01	6.16E-01	4.03E-06	14.22	2.68	Phocaeicola_dorei_97	Bacteria_Bacteroidetes_Bacteroidia_Bacteroidales_Bacteroidaceae_Phocaeicola_94
OTU_1351	8.54E-01	6.04E-01	8.66E-10	15.76	2.12	Parabacteroides_distasonis_97	Bacteria_Bacteroidetes_Bacteroidia_Bacteroidales_Porphyromonadaceae_Parabacteroides_100
OTU_186	7.94E-01	6.04E-01	3.39E-02	18.66	0.92	Eisenbergiella_massiliensis_93	Bacteria_Firmicutes_Clostridia_Clostridiales_Lachnospiraceae_Stomatobaculum_48
OTU_769	7.07E-01	4.60E-01	9.01E-02	11.72	6.67	Pseudescherichia_vulneris_96	Bacteria_Proteobacteria_Gammaproteobacteria_Enterobacterales_Enterobacteriaceae_Pseudescherichia_51
OTU_562	7.07E-01	6.04E-01	1.42E-01	9.15	5.06	Collinsella_aerofaciens_97	Bacteria_Actinobacteria_Coriobacteriia_Coriobacteriales_Coriobacteriaceae_Collinsella_100
OTU_916	7.59E-01	7.67E-01	6.77E-04	12.16	2.21	Anaerobacillus_alkalilacustris_94	Bacteria_Firmicutes_Bacilli_99_Lactobacillales_94_Carnobacteriaceae_75_Isobaculum_55
OTU_3920	8.48E-01	6.73E-01	3.38E-01	12.66	9.93	Clostridium_symbiosum_95	Bacteria_Firmicutes_Clostridia_Clostridiales_Lachnospiraceae_Coprococcus_46
OTU_167	8.60E-01	6.04E-01	6.48E-02	16.51	3.39	Ruminiclostridium_cellobioparum_88	Bacteria_Firmicutes_91_Clostridia_86_Clostridiales_85_Lachnospiraceae_27_Abyssivirga_12
OTU_1174	7.07E-01	4.60E-01	2.33E-04	14.09	6.38	Clostridium_perfringens_97	Bacteria_Firmicutes_Clostridia_98_Clostridiales_98_Clostridiaceae 1_95_Clostridium sensu stricto_54
OTU_259	8.06E-01	6.72E-01	3.93E-02	3.12	14.14	Escherichia_albertii_98	Bacteria_Proteobacteria_Gammaproteobacteria_Enterobacterales_Morganellaceae_66_Morganella_60
OTU_1776	8.97E-01	9.70E-01	2.66E-02	6.24	10.78	Hungatella_hathewayi_96	Bacteria_Firmicutes_Clostridia_Clostridiales_Lachnospiraceae_Hungatella_66
OTU_3076	8.31E-01	6.04E-01	7.31E-04	13.32	3.62	Parabacteroides_distasonis_98	Bacteria_Bacteroidetes_Bacteroidia_Bacteroidales_Porphyromonadaceae_Parabacteroides_100
OTU_48702	7.07E-01	6.72E-01	1.75E-01	10.78	1.08	Veillonella_atypica_97	Bacteria_Firmicutes_Negativicutes_Veillonellales_Veillonellaceae_Veillonella_100
OTU_1044	9.78E-01	7.73E-01	2.11E-02	16.04	7.12	Eubacterium_oxidoreducens_97	Bacteria_Firmicutes_Clostridia_Clostridiales_Lachnospiraceae_Roseburia_54
OTU_197	7.91E-01	8.83E-01	6.35E-04	2.38	6.91	Phocaeicola_coprophilus_99	Bacteria_Bacteroidetes_86_Bacteroidia_86_Bacteroidales_86_Bacteroidaceae_86_Phocaeicola_78
OTU_290	7.07E-01	9.93E-01	8.41E-02	4.07	0.76	Collinsella_aerofaciens_97	Bacteria_Actinobacteria_Coriobacteriia_Coriobacteriales_99_Coriobacteriaceae_99_Collinsella_99
OTU_1035	9.08E-01	8.79E-01	1.84E-03	2.88	13.59	Bacteroides_thetaiotaomicron_95	Bacteria_Bacteroidetes_Bacteroidia_Bacteroidales_Bacteroidaceae_Bacteroides_89
OTU_510	7.59E-01	7.77E-01	1.61E-01	7.35	13.43	Enterocloster_lavalensis_93	Bacteria_Firmicutes_Clostridia_Clostridiales_Lachnospiraceae_88_Clostridium XlVa_26
OTU_570	8.25E-01	8.78E-01	3.09E-01	7.33	9.19	Enterocloster_clostridioformis_90	Bacteria_Firmicutes_77_Clostridia_77_Clostridiales_77_Lachnospiraceae_77_Murimonas_8
OTU_1472	7.46E-01	6.72E-01	3.88E-04	14.12	5.56	Hungatella_effluvii_96	Bacteria_Firmicutes_Clostridia_Clostridiales_Lachnospiraceae_Hungatella_88
OTU_633	1.00E+00	7.73E-01	7.71E-04	15.79	1.00	Veillonella_atypica_91	Bacteria_Firmicutes_Negativicutes_87_Veillonellales_66_Veillonellaceae_66_Veillonella_64
OTU_1235	7.07E-01	8.42E-01	8.73E-01	8.80	9.31	Veillonella_parvula_98	Bacteria_Firmicutes_Negativicutes_Veillonellales_Veillonellaceae_Veillonella_100
OTU_200	9.46E-01	7.31E-01	1.11E-02	0.67	7.22	Phocaeicola_coprophilus_95	Bacteria_Bacteroidetes_Bacteroidia_Bacteroidales_Bacteroidaceae_Phocaeicola_100
OTU_379	7.81E-01	6.04E-01	8.50E-02	6.55	12.72	Pseudescherichia_vulneris_99	Bacteria_Proteobacteria_51_Gammaproteobacteria_51_Enterobacterales_51_Enterobacteriaceae_50_Pseudescherichia_45
OTU_185	8.06E-01	9.69E-01	9.21E-02	2.35	1.21	Phocaeicola_vulgatus_96	Bacteria_Bacteroidetes_Bacteroidia_Bacteroidales_Bacteroidaceae_Phocaeicola_100
OTU_171	7.07E-01	6.50E-01	2.28E-01	13.18	5.88	Pseudescherichia_vulneris_99	Bacteria_Proteobacteria_93_Gammaproteobacteria_93_Enterobacterales_93_Enterobacteriaceae_93_Pseudescherichia_81
OTU_632	7.59E-01	6.36E-01	3.12E-01	8.01	10.78	Kineothrix_alysoides_94	Bacteria_Firmicutes_Clostridia_Clostridiales_Lachnospiraceae_Kineothrix_31
OTU_359	9.08E-01	9.76E-01	4.24E-03	16.29	3.73	Collinsella_aerofaciens_93	Bacteria_Actinobacteria_94_Coriobacteriia_94_Coriobacteriales_94_Coriobacteriaceae_94_Collinsella_93
OTU_388	9.02E-01	7.58E-01	9.96E-02	3.48	2.32	Faecalibacterium_prausnitzii_93	Bacteria_Firmicutes_Clostridia_Clostridiales_Ruminococcaceae_97_Faecalibacterium_90
OTU_1647	7.85E-01	9.46E-01	3.79E-02	9.55	12.57	Bacteroides_thetaiotaomicron_98	Bacteria_Bacteroidetes_Bacteroidia_Bacteroidales_Bacteroidaceae_Bacteroides_100
OTU_329	9.92E-01	6.04E-01	2.28E-01	6.60	13.57	Veillonella_parvula_91	Bacteria_Firmicutes_Erysipelotrichia_57_Erysipelotrichales_57_Erysipelatoclostridiaceae_57_Erysipelatoclostridium_57
OTU_734	8.16E-01	7.73E-01	5.77E-01	7.75	8.75	Escherichia_coli_92	Bacteria_Proteobacteria_Gammaproteobacteria_Enterobacterales_Enterobacteriaceae_Pseudescherichia_93
OTU_2150	9.93E-01	9.54E-01	2.69E-02	10.33	7.52	Coprococcus_comes_97	Bacteria_Firmicutes_Clostridia_Clostridiales_Lachnospiraceae_Coprococcus_65
OTU_201	7.07E-01	6.04E-01	5.06E-01	6.01	10.70	Pseudescherichia_vulneris_91	Bacteria_Proteobacteria_Gammaproteobacteria_Enterobacterales_Enterobacteriaceae_Pseudescherichia_51
OTU_475	9.93E-01	6.93E-01	3.15E-01	12.56	9.37	Enterococcus_faecalis_91	Bacteria_Firmicutes_Clostridia_65_Clostridiales_65_Lachnospiraceae_65_Roseburia_35
OTU_998	9.38E-01	6.04E-01	3.13E-04	16.68	3.61	Bacteroides_stercoris_97	Bacteria_Bacteroidetes_Bacteroidia_Bacteroidales_Bacteroidaceae_Bacteroides_100
OTU_828	9.66E-01	9.36E-01	8.59E-04	4.25	14.20	Phascolarctobacterium_faecium_97	Bacteria_Firmicutes_Negativicutes_Acidaminococcales_Acidaminococcaceae_Phascolarctobacterium_100
OTU_2391	7.07E-01	6.50E-01	6.15E-01	8.45	9.39	Enterococcus_hirae_97	Bacteria_Firmicutes_Bacilli_Lactobacillales_99_Enterococcaceae_91_Vagococcus_29
OTU_593	7.45E-01	9.69E-01	7.10E-04	17.92	3.16	Clostridium_perfringens_100	Bacteria_Firmicutes_66_Clostridia_66_Clostridiales_66_Clostridiaceae 1_66_Anaerobacter_20
OTU_9590	9.10E-01	9.43E-01	1.47E-02	10.91	7.51	Hungatella_effluvii_97	Bacteria_Firmicutes_Clostridia_Clostridiales_Lachnospiraceae_Hungatella_88

OTU_4591	7.73E-01	9.37E-01	1.49E-01	11.68	9.01	Roseburia_inulinivorans_97	Bacteria_Firmicutes_Clostridia_Clostridiales_Lachnospiraceae_Roseburia_70
OTU_1723	8.89E-01	9.94E-01	1.42E-01	7.93	9.49	Pseudescherichia_vulneris_100	Bacteria_Proteobacteria_99_Gammaproteobacteria_99_Enterobacterales_98_Enterobacteriaceae_97_Pseudescherichia_51
OTU_2235	7.33E-01	6.05E-01	8.26E-03	5.07	8.44	Enterococcus_faecalis_98	Bacteria_Firmicutes_Bacilli_Lactobacillales_Enterococcaceae_96_Enterococcus_85
OTU_83	7.07E-01	9.12E-01	5.78E-01	0.60	0.42	Methanobrevibacter_smithii_100	Archaea_Euryarchaeota_Methanobacteria_Methanobacteriales_Methanobacteriaceae_Methanobrevibacter_100
OTU_4534	8.75E-01	9.61E-01	1.52E-01	8.17	10.75	Veillonella_dispar_99	Bacteria_Firmicutes_Negativicutes_87_Veillonellales_85_Veillonellaceae_85_Veillonella_84
OTU_4416	7.07E-01	9.42E-01	3.33E-07	14.66	3.78	Clostridium_tarantellae_93	Bacteria_Firmicutes_Clostridia_Clostridiales_Lachnospiraceae_52_Ruminococcus2_17
OTU_292	8.18E-01	8.42E-01	6.36E-02	5.82	2.96	Kineothrix_alysoides_97	Bacteria_Firmicutes_Clostridia_Clostridiales_Lachnospiraceae_Kineothrix_50
OTU_721	8.97E-01	6.04E-01	1.04E-01	9.90	7.04	Peptoniphilus_grossensis_98	Bacteria_Firmicutes_74_Clostridia_74_Clostridiales_74_Peptoniphilaceae_74_Peptoniphilus_74
OTU_183	7.07E-01	6.04E-01	7.10E-01	6.91	8.65	Escherichia_coli_91	Bacteria_Proteobacteria_Gammaproteobacteria_85_Enterobacterales_85_Enterobacteriaceae_85_Pseudescherichia_84
OTU_4233	7.59E-01	9.05E-01	8.49E-03	10.21	6.02	Enterocloster_citroniae_97	Bacteria_Firmicutes_Clostridia_Clostridiales_Lachnospiraceae_Enterocloster_81
OTU_385	7.07E-01	9.38E-01	4.03E-03	1.86	12.47	Enterococcus_canis_93	Bacteria_Firmicutes_Bacilli_66_Lactobacillales_53_Carnobacteriaceae_41_Isobaculum_27
OTU_28886	9.87E-01	9.61E-01	1.32E-02	6.98	2.12	Paeniclostridium_sordellii_99	Bacteria_Firmicutes_Clostridia_Clostridiales_Peptostreptococcaceae_Paeniclostridium_100
OTU_1114	7.07E-01	7.80E-01	9.70E-02	8.68	10.71	Pseudescherichia_vulneris_99	Bacteria_Proteobacteria_96_Gammaproteobacteria_96_Enterobacterales_96_Enterobacteriaceae_96_Pseudescherichia_77
OTU_1134	7.11E-01	9.12E-01	5.27E-01	8.10	8.81	Clostridium_symbiosum_91	Bacteria_Firmicutes_87_Clostridia_87_Clostridiales_87_Lachnospiraceae_87_Stomatobaculum_21
OTU_1956	9.67E-01	6.04E-01	2.63E-01	7.98	9.25	Pseudescherichia_vulneris_97	Bacteria_Proteobacteria_Gammaproteobacteria_Enterobacterales_Enterobacteriaceae_Escherichia/Shigella_81
OTU_382	7.57E-01	6.04E-01	6.15E-01	8.12	9.27	Acetanaerobacterium_elongatum_90	Bacteria_Firmicutes_Clostridia_Clostridiales_Ruminococcaceae_Hydrogenoanaerobacterium_36
OTU_2158	7.14E-01	6.04E-01	3.57E-01	9.13	4.57	Veillonella_rogosae_98	Bacteria_Firmicutes_Negativicutes_Veillonellales_Veillonellaceae_Veillonella_100
OTU_179	8.01E-01	9.12E-01	2.40E-01	11.21	5.76	Pseudescherichia_vulneris_93	Bacteria_Proteobacteria_Gammaproteobacteria_Enterobacterales_95_Enterobacteriaceae_92_Escherichia/Shigella_45
OTU_220	7.07E-01	6.04E-01	1.82E-01	11.78	3.63	Fusobacterium_mortiferum_95	Bacteria_Fusobacteria_80_Fusobacteriia_80_Fusobacteriales_80_Fusobacteriaceae_72_Cetobacterium_43
OTU_229	7.88E-01	6.83E-01	6.35E-02	0.07	14.83	Methyloversatilis_universalis_100	Bacteria_Proteobacteria_Betaproteobacteria_Nitrosomonadales_Sterolibacteriaceae_Methyloversatilis_100
OTU_601	9.93E-01	6.72E-01	2.68E-01	10.14	8.44	Bacteroides_acidifaciens_89	Bacteria_Proteobacteria_76_Gammaproteobacteria_76_Enterobacterales_76_Enterobacteriaceae_76_Pseudescherichia_68
OTU_940	9.37E-01	6.47E-01	5.24E-04	10.85	4.67	Pseudescherichia_vulneris_96	Bacteria_Proteobacteria_Gammaproteobacteria_Enterobacterales_Enterobacteriaceae_Escherichia/Shigella_83
OTU_144	9.38E-01	9.70E-01	2.12E-01	3.52	2.14	Ruminococcus_bromii_98	Bacteria_Firmicutes_Clostridia_Clostridiales_Ruminococcaceae_Ruminococcus_67
OTU_194	9.78E-01	7.80E-01	8.94E-01	4.15	4.57	Parabacteroides_johnsonii_96	Bacteria_Bacteroidetes_Bacteroidia_Bacteroidales_Porphyromonadaceae_Parabacteroides_100
OTU_32704	7.33E-01	6.63E-01	2.43E-03	9.57	1.91	Bifidobacterium_stercoris_98	Bacteria_Actinobacteria_Actinobacteria_Bifidobacteriales_Bifidobacteriaceae_Bifidobacterium_100
OTU_615	8.16E-01	8.65E-01	3.98E-02	5.95	9.24	Escherichia_albertii_88	Bacteria_Proteobacteria_63_Gammaproteobacteria_63_Enterobacterales_63_Enterobacteriaceae_63_Pseudescherichia_63
OTU_3970	9.78E-01	7.80E-01	3.65E-01	9.01	7.92	Kineothrix_alysoides_97	Bacteria_Firmicutes_Clostridia_Clostridiales_Lachnospiraceae_Clostridium XlVa_42
OTU_4479	9.75E-01	6.39E-01	6.33E-04	5.47	12.47	Pseudescherichia_vulneris_97	Bacteria_Proteobacteria_91_Gammaproteobacteria_91_Enterobacterales_91_Enterobacteriaceae_90_Pseudescherichia_88
OTU_300	8.19E-01	6.36E-01	6.02E-03	5.23	10.36	Murimonas_intestini_96	Bacteria_Firmicutes_Clostridia_Clostridiales_Lachnospiraceae_Eisenbergiella_14
OTU_812	7.10E-01	8.50E-01	2.99E-03	10.12	2.01	Bifidobacterium_stercoris_97	Bacteria_Actinobacteria_Actinobacteria_Bifidobacteriales_Bifidobacteriaceae_Bifidobacterium_99
OTU_777	7.88E-01	9.67E-01	1.71E-02	12.51	4.04	Ruminococcus_gnavus_97	Bacteria_Firmicutes_Clostridia_Clostridiales_Lachnospiraceae_Dorea_38
OTU_310	1.00E+00	6.04E-01	5.52E-03	4.44	10.58	Enterococcus_faecalis_91	Bacteria_Firmicutes_Clostridia_94_Clostridiales_94_Peptostreptococcaceae_94_Paraclostridium_93
OTU_163	7.07E-01	7.40E-01	3.63E-01	5.11	1.33	Collinsella_aerofaciens_96	Bacteria_Actinobacteria_Coriobacteriia_Coriobacteriales_96_Coriobacteriaceae_96_Collinsella_96
OTU_698	1.00E+00	6.73E-01	6.83E-03	7.87	2.01	Phocaeicola_dorei_96	Bacteria_Bacteroidetes_Bacteroidia_Bacteroidales_Bacteroidaceae_Bacteroides_89
OTU_898	7.07E-01	7.44E-01	1.16E-02	4.95	12.80	Faecalibacterium_prausnitzii_93	Bacteria_Firmicutes_Clostridia_Clostridiales_Ruminococcaceae_Faecalibacterium_59
OTU_2313	9.32E-01	9.25E-01	4.92E-02	6.72	9.04	Enterocloster_citroniae_97	Bacteria_Firmicutes_Clostridia_Clostridiales_Lachnospiraceae_Sellimonas_51
OTU_390	1.00E+00	7.94E-01	2.46E-03	13.28	3.27	Clostridium_tarantellae_93	Bacteria_Firmicutes_97_Clostridia_97_Clostridiales_97_Clostridiaceae 1_92_Anaerobacter_18
OTU_598	9.60E-01	6.04E-01	6.35E-01	8.00	9.03	Paraclostridium_benzoelyticum_97	Bacteria_Firmicutes_Clostridia_Clostridiales_Peptostreptococcaceae_Paraclostridium_93
OTU_556	9.14E-01	8.83E-01	5.36E-03	2.27	12.59	Bacteroides_fragilis_94	Bacteria_Bacteroidetes_99_Bacteroidia_99_Bacteroidales_99_Bacteroidaceae_99_Bacteroides_97
OTU_174	7.07E-01	8.42E-01	1.46E-01	3.78	1.20	Caecibacter_massiliensis_98	Bacteria_Firmicutes_Negativicutes_Veillonellales_Veillonellaceae_Megasphaera_100
OTU_2104	7.46E-01	9.77E-01	6.31E-01	7.34	7.89	Hungatella_effluvii_97	Bacteria_Firmicutes_Clostridia_Clostridiales_Lachnospiraceae_Hungatella_100
OTU_135	7.82E-01	6.04E-01	4.92E-02	10.33	1.55	Vallitalea_pronyensis_86	Bacteria_Firmicutes_97_Clostridia_92_Clostridiales_92_Lachnospiraceae_30_Lactonifactor_11
OTU_3874	7.57E-01	8.36E-01	8.09E-01	7.70	7.47	Hespellia_porcina_90	Bacteria_Proteobacteria_70_Gammaproteobacteria_70_Enterobacterales_70_Enterobacteriaceae_70_Pseudescherichia_67
OTU_1370	7.46E-01	4.38E-02	9.81E-05	10.96	4.32	Clostridium_perfringens_95	Bacteria_Firmicutes_Clostridia_55_Clostridiales_55_Clostridiaceae 1_50_Anaerobacter_19
OTU_10302	7.91E-01	9.53E-01	7.03E-03	13.70	1.63	Bacteroides_xylanisolvens_97	Bacteria_Bacteroidetes_Bacteroidia_Bacteroidales_Bacteroidaceae_Bacteroides_100
OTU_3371	7.82E-01	6.04E-01	3.15E-03	5.19	9.50	Enterococcus_faecalis_97	Bacteria_Firmicutes_Bacilli_Lactobacillales_Enterococcaceae_94_Enterococcus_85
OTU_628	9.59E-01	9.54E-01	2.69E-03	4.22	7.49	Flintibacter_butyricus_89	Bacteria_Firmicutes_80_Clostridia_80_Clostridiales_80_Ruminococcaceae_80_Flavonifractor_80
OTU_1528	9.14E-01	9.34E-01	1.77E-02	5.18	9.64	Veillonella_dispar_87	Bacteria_Proteobacteria_70_Gammaproteobacteria_70_Enterobacterales_69_Enterobacteriaceae_69_Pseudescherichia_69
OTU_1001	9.98E-01	6.04E-01	1.59E-01	5.48	8.25	Enterococcus_faecalis_97	Bacteria_Firmicutes_Bacilli_99_Lactobacillales_98_Enterococcaceae_92_Enterococcus_79
OTU_125	7.07E-01	8.14E-01	2.43E-02	7.56	0.79	Solobacterium_moorei_99	Bacteria_Firmicutes_Erysipelotrichia_Erysipelotrichales_Erysipelotrichaceae_Solobacterium_100
OTU_243	7.57E-01	6.04E-01	9.79E-02	11.82	4.61	Escherichia_albertii_91	Bacteria_Proteobacteria_Gammaproteobacteria_Aeromonadales_94_Succinivibrionaceae_94_Succinivibrio_94
OTU_9774	9.31E-01	6.04E-01	5.41E-04	9.84	4.24	Escherichia_albertii_92	Bacteria_Proteobacteria_76_Gammaproteobacteria_76_Enterobacterales_76_Enterobacteriaceae_76_Pseudescherichia_76
OTU_151	9.31E-01	7.30E-01	2.59E-02	13.26	2.43	Finegoldia_magna_100	Bacteria_Firmicutes_Clostridia_Clostridiales_Peptoniphilaceae_Finegoldia_100
OTU_38387	7.10E-01	9.89E-01	2.97E-02	15.07	2.14	Bacteroides_faecis_97	Bacteria_Bacteroidetes_Bacteroidia_Bacteroidales_Bacteroidaceae_Bacteroides_100
OTU_484	7.57E-01	8.97E-01	4.91E-03	15.07	1.89	Bifidobacterium_longum_92	Bacteria_Actinobacteria_99_Actinobacteria_99_Bifidobacteriales_99_Bifidobacteriaceae_99_Pseudoscardovia_47
OTU_4176	7.74E-01	6.04E-01	7.94E-03	5.24	10.04	Escherichia_coli_90	Bacteria_Proteobacteria_Gammaproteobacteria_Enterobacterales_Enterobacteriaceae_Pseudescherichia_79
OTU_1691	9.87E-01	7.02E-01	1.87E-02	4.98	7.20	Enterocloster_citroniae_96	Bacteria_Firmicutes_Clostridia_Clostridiales_Lachnospiraceae_Enterocloster_98
OTU_262	9.98E-01	6.04E-01	3.95E-02	16.05	2.87	Bifidobacterium_pseudolongum_100	Bacteria_Actinobacteria_Actinobacteria_Bifidobacteriales_Bifidobacteriaceae_Bifidobacterium_100
OTU_12789	9.14E-01	6.05E-01	6.29E-01	7.06	7.54	Shigella_dysenteriae_95	Bacteria_Proteobacteria_Gammaproteobacteria_Enterobacterales_Enterobacteriaceae_Pseudescherichia_73
OTU_2236	9.65E-01	9.12E-01	2.30E-02	5.02	7.85	Enterococcus_sulfureus_88	Bacteria_Firmicutes_98_Negativicutes_63_Selenomonadales_58_Sporomusaceae_54_Anaerosinus_54
OTU_2009	7.07E-01	8.73E-01	8.13E-05	13.53	1.19	Bacteroides_finegoldii_96	Bacteria_Bacteroidetes_Bacteroidia_Bacteroidales_Bacteroidaceae_Bacteroides_89
OTU_487	8.06E-01	9.74E-01	7.22E-01	1.18	0.96	Phocaeicola_coprocola_96	Bacteria_Bacteroidetes_Bacteroidia_Bacteroidales_Bacteroidaceae_Phocaeicola_100
OTU_280	7.07E-01	6.99E-01	4.03E-03	10.69	3.64	Intestinimonas_butyriciproducens_100	Bacteria_Firmicutes_Clostridia_Clostridiales_Ruminococcaceae_Intestinimonas_100
OTU_3228	9.78E-01	9.70E-01	4.61E-02	9.89	7.81	Bacteroides_thetaiotaomicron_98	Bacteria_Bacteroidetes_Bacteroidia_Bacteroidales_Bacteroidaceae_Bacteroides_100
OTU_729	7.85E-01	7.96E-01	1.52E-03	6.04	2.18	Phocaeicola_vulgatus_100	Bacteria_Bacteroidetes_94_Bacteroidia_94_Bacteroidales_94_Bacteroidaceae_94_Phocaeicola_86
OTU_12878	9.69E-01	9.46E-01	2.29E-04	4.31	10.29	Gibbsiella_dentisursi_87	Bacteria_Proteobacteria_90_Gammaproteobacteria_90_Enterobacterales_90_Enterobacteriaceae_90_Pseudescherichia_89
OTU_620	7.07E-01	6.04E-01	2.20E-02	10.05	5.20	Erysipelatoclostridium_ramosum_98	Bacteria_Firmicutes_Erysipelotrichia_Erysipelotrichales_Erysipelatoclostridiaceae_97_Erysipelatoclostridium_97

OTU_1376	7.07E-01	9.94E-01	6.97E-04	11.86	1.88	Clostridium_tarantellae_97	Bacteria_Firmicutes_Clostridia_82_Clostridiales_82_Clostridiaceae 1_81_Anaerobacter_29
OTU_111	7.88E-01	7.07E-01	1.79E-01	0.03	9.23	Nevskia_lacus_100	Bacteria_Proteobacteria_Gammaproteobacteria_Nevskiales_Nevskiaceae_Nevskia_100
OTU_326	8.06E-01	7.02E-01	1.33E-02	0.37	5.25	Bacteroides_mediterraneensis_96	Bacteria_Bacteroidetes_Bacteroidia_Bacteroidales_Bacteroidaceae_Phocaeicola_100
OTU_443	9.08E-01	6.04E-01	7.86E-02	4.76	10.39	Erysipelatoclostridium_ramosum_96	Bacteria_Firmicutes_Erysipelotrichia_Erysipelotrichales_Erysipelatoclostridiaceae_Erysipelatoclostridium_100
OTU_337	9.06E-01	7.65E-01	1.89E-03	1.53	11.28	Phascolarctobacterium_faecium_93	Bacteria_Firmicutes_Negativicutes_Acidaminococcales_Acidaminococcaceae_Phascolarctobacterium_100
OTU_98	8.31E-01	7.67E-01	7.65E-01	3.14	3.69	Dialister_invisus_100	Bacteria_Firmicutes_Negativicutes_Veillonellales_Veillonellaceae_Dialister_100
OTU_1092	7.46E-01	6.04E-01	2.55E-02	19.09	0.12	Prevotella_copri_96	Bacteria_Bacteroidetes_Bacteroidia_Bacteroidales_Prevotellaceae_Prevotella_99
OTU_10803	9.45E-01	9.53E-01	7.71E-04	4.60	7.29	Pseudescherichia_vulneris_95	Bacteria_Proteobacteria_Gammaproteobacteria_Enterobacterales_Enterobacteriaceae_Pseudescherichia_62
OTU_9992	7.07E-01	8.79E-01	1.40E-02	5.38	7.76	Pseudescherichia_vulneris_90	Bacteria_Proteobacteria_54_Gammaproteobacteria_54_Enterobacterales_54_Enterobacteriaceae_50_Pseudescherichia_50
OTU_4069	9.43E-01	9.41E-01	5.40E-01	6.67	5.58	Bacteroides_fragilis_97	Bacteria_Bacteroidetes_Bacteroidia_Bacteroidales_Bacteroidaceae_Bacteroides_100
OTU_1348	8.33E-01	8.72E-01	1.43E-01	5.42	6.83	Enterococcus_faecalis_92	Bacteria_Firmicutes_Clostridia_95_Clostridiales_95_Lachnospiraceae_95_Hungatella_94
OTU_3006	7.07E-01	9.41E-01	2.61E-04	10.78	3.45	Coprococcus_comes_97	Bacteria_Firmicutes_Clostridia_Clostridiales_Lachnospiraceae_Coprococcus_75
OTU_651	7.07E-01	9.74E-01	2.68E-01	3.47	5.02	Enterocloster_clostridioformis_94	Bacteria_Firmicutes_Clostridia_Clostridiales_Lachnospiraceae_Butyrivibrio_30
OTU_939	9.61E-01	6.04E-01	5.42E-03	12.93	2.50	Pseudescherichia_vulneris_96	Bacteria_Proteobacteria_Gammaproteobacteria_98_Enterobacterales_98_Enterobacteriaceae_97_Pseudescherichia_69
OTU_378	9.59E-01	7.31E-01	1.20E-01	1.74	10.89	Burkholderia_pseudomultivorans_93	Bacteria_Proteobacteria_Gammaproteobacteria_89_Enterobacterales_85_Enterobacteriaceae_85_Pseudescherichia_73
OTU_6325	7.33E-01	9.61E-01	9.20E-02	5.56	8.34	Veillonella_dispar_96	Bacteria_Firmicutes_Negativicutes_95_Veillonellales_89_Veillonellaceae_89_Veillonella_89
OTU_860	9.61E-01	6.04E-01	1.69E-01	4.82	6.54	Enterocloster_clostridioformis_93	Bacteria_Firmicutes_Clostridia_Clostridiales_Lachnospiraceae_Butyrivibrio_25
OTU_3943	7.57E-01	9.61E-01	2.53E-01	6.14	7.53	Pseudescherichia_vulneris_97	Bacteria_Proteobacteria_Gammaproteobacteria_Enterobacterales_Enterobacteriaceae_Escherichia/Shigella_64
OTU_539	7.10E-01	9.54E-01	2.47E-01	5.57	8.65	Kineothrix_alysoides_95	Bacteria_Firmicutes_Clostridia_Clostridiales_Lachnospiraceae_Kineothrix_40
OTU_708	7.07E-01	6.04E-01	1.12E-04	11.72	1.70	Streptococcus_mitis_97	Bacteria_Firmicutes_Bacilli_Lactobacillales_Streptococcaceae_99_Streptococcus_98
OTU_569	7.93E-01	8.73E-01	9.10E-04	9.62	1.84	Pseudescherichia_vulneris_100	Bacteria_Proteobacteria_91_Gammaproteobacteria_91_Enterobacterales_90_Enterobacteriaceae_90_Pseudescherichia_87
OTU_83047	7.59E-01	9.94E-01	5.10E-04	2.88	11.09	Veillonella_tobetsuensis_87	Bacteria_Proteobacteria_67_Gammaproteobacteria_67_Enterobacterales_67_Enterobacteriaceae_66_Pseudescherichia_66
OTU_30097	9.78E-01	6.04E-01	3.69E-03	10.15	4.47	Bacteroides_faecis_97	Bacteria_Bacteroidetes_Bacteroidia_Bacteroidales_Bacteroidaceae_Bacteroides_100
OTU_1701	9.92E-01	6.04E-01	2.98E-01	7.35	6.06	Hungatella_effluvii_96	Bacteria_Firmicutes_Clostridia_Clostridiales_Lachnospiraceae_Hungatella_60
OTU_1324	7.07E-01	6.14E-01	5.88E-01	6.21	5.37	Blautia_faecis_100	Bacteria_Firmicutes_Clostridia_Clostridiales_Lachnospiraceae_Blautia_100
OTU_617	8.30E-01	9.46E-01	7.65E-05	12.99	1.06	Anaerotignum_aminivorans_93	Bacteria_Firmicutes_Clostridia_Clostridiales_Lachnospiraceae_84_Eisenbergiella_9
OTU_785	8.16E-01	7.73E-01	6.84E-03	3.39	9.09	Vibrio_quintilis_88	Bacteria_Proteobacteria_Gammaproteobacteria_Enterobacterales_Enterobacteriaceae_99_Pseudescherichia_84
OTU_458	7.07E-01	6.04E-01	7.06E-01	2.34	2.02	Collinsella_aerofaciens_97	Bacteria_Actinobacteria_Coriobacteriia_Coriobacteriales_Coriobacteriaceae_Collinsella_100
OTU_566	8.06E-01	7.44E-01	1.27E-01	5.53	9.41	Kineothrix_alysoides_96	Bacteria_Firmicutes_Clostridia_Clostridiales_Lachnospiraceae_Kineothrix_50
OTU_6534	7.11E-01	8.54E-01	1.87E-05	11.74	1.87	Bacteroides_uniformis_96	Bacteria_Bacteroidetes_Bacteroidia_Bacteroidales_Bacteroidaceae_Bacteroides_80
OTU_36016	8.39E-01	6.04E-01	1.29E-02	3.35	6.24	Enterococcus_faecalis_99	Bacteria_Firmicutes_Bacilli_Lactobacillales_99_Enterococcaceae_97_Enterococcus_93
OTU_766	9.72E-01	7.38E-01	9.22E-02	5.06	7.13	Clostridium_symbiosum_95	Bacteria_Firmicutes_Clostridia_Clostridiales_Lachnospiraceae_Clostridium XlVa_40
OTU_1222	7.75E-01	7.59E-01	7.70E-02	5.20	7.32	Escherichia_albertii_91	Bacteria_Proteobacteria_94_Gammaproteobacteria_94_Enterobacterales_94_Enterobacteriaceae_94_Pseudescherichia_93
OTU_1428	9.87E-01	6.04E-01	2.99E-03	10.39	2.02	Bacteroides_clarus_98	Bacteria_Bacteroidetes_Bacteroidia_Bacteroidales_Bacteroidaceae_Bacteroides_100
OTU_739	9.06E-01	8.78E-01	7.88E-01	3.22	3.54	Paeniclostridium_sordellii_99	Bacteria_Firmicutes_Clostridia_Clostridiales_Peptostreptococcaceae_Paeniclostridium_100
OTU_3837	8.19E-01	7.02E-01	7.42E-02	7.27	4.16	Blautia_coccoides_98	Bacteria_Firmicutes_Clostridia_Clostridiales_Lachnospiraceae_Blautia_100
OTU_762	9.31E-01	8.33E-01	4.35E-02	4.08	9.65	Bacteroides_fragilis_98	Bacteria_Bacteroidetes_69_Bacteroidia_69_Bacteroidales_69_Bacteroidaceae_67_Mediterranea_57
OTU_145	7.77E-01	8.28E-01	9.43E-01	1.06	1.02	Bacteroides_gallinaceum_100	Bacteria_Bacteroidetes_Bacteroidia_Bacteroidales_Bacteroidaceae_98_Bacteroides_80
OTU_3766	7.68E-01	8.71E-01	9.35E-01	6.95	6.78	Coprococcus_comes_97	Bacteria_Firmicutes_Clostridia_Clostridiales_Lachnospiraceae_Coprococcus_71
OTU_4633	9.75E-01	8.79E-01	5.05E-02	9.64	5.41	Roseburia_inulinivorans_97	Bacteria_Firmicutes_Clostridia_Clostridiales_Lachnospiraceae_Roseburia_61
OTU_1452	9.05E-01	9.57E-01	1.96E-03	3.15	6.02	Enterocloster_citroniae_93	Bacteria_Firmicutes_Clostridia_Clostridiales_Lachnospiraceae_73_Enterocloster_66
OTU_2759	9.23E-01	8.65E-01	7.58E-01	6.93	6.56	Enterococcus_hirae_97	Bacteria_Firmicutes_Bacilli_Lactobacillales_98_Enterococcaceae_83_Enterococcus_62
OTU_165	9.73E-01	6.04E-01	6.57E-02	14.01	1.77	Kineothrix_alysoides_98	Bacteria_Firmicutes_Clostridia_Clostridiales_Lachnospiraceae_Kineothrix_68
OTU_3119	9.87E-01	6.27E-01	5.00E-01	6.16	7.11	Paraclostridium_benzoelyticum_96	Bacteria_Firmicutes_Clostridia_Clostridiales_Peptostreptococcaceae_Paraclostridium_91
OTU_1938	1.00E+00	6.93E-01	6.63E-01	6.20	6.65	Pseudescherichia_vulneris_96	Bacteria_Proteobacteria_Gammaproteobacteria_Enterobacterales_Enterobacteriaceae_Escherichia/Shigella_88
OTU_7396	7.07E-01	6.04E-01	2.82E-08	13.68	1.62	Bacteroides_uniformis_96	Bacteria_Bacteroidetes_Bacteroidia_Bacteroidales_Bacteroidaceae_Bacteroides_100
OTU_372	9.14E-01	7.31E-01	6.68E-01	0.96	1.15	Phocaeicola_vulgatus_96	Bacteria_Bacteroidetes_Bacteroidia_Bacteroidales_Bacteroidaceae_Phocaeicola_100
OTU_656	9.78E-01	9.60E-01	1.01E-02	1.91	11.09	Clostridium_symbiosum_96	Bacteria_Firmicutes_76_Clostridia_76_Clostridiales_76_Lachnospiraceae_76_Faecalimonas_17
OTU_3362	8.53E-01	6.04E-01	4.42E-02	4.61	6.49	Enterococcus_faecalis_95	Bacteria_Firmicutes_Bacilli_95_Lactobacillales_94_Enterococcaceae_73_Melissococcus_40
OTU_11360	7.57E-01	6.04E-01	7.10E-04	9.52	3.57	Clostridium_perfringens_97	Bacteria_Firmicutes_Clostridia_Clostridiales_Clostridiaceae 1_90_Clostridium sensu stricto_56
OTU_4150	7.10E-01	9.41E-01	2.56E-05	10.62	1.12	Bacteroides_clarus_98	Bacteria_Bacteroidetes_Bacteroidia_Bacteroidales_Bacteroidaceae_Bacteroides_96
OTU_2126	9.08E-01	7.75E-01	3.09E-01	7.97	6.72	Roseburia_inulinivorans_88	Bacteria_Proteobacteria_73_Gammaproteobacteria_73_Enterobacterales_73_Enterobacteriaceae_72_Pseudescherichia_67
OTU_8777	9.14E-01	8.68E-01	4.67E-02	5.35	8.37	Pseudescherichia_vulneris_96	Bacteria_Proteobacteria_Gammaproteobacteria_Enterobacterales_Enterobacteriaceae_Escherichia/Shigella_78
OTU_1094	8.33E-01	8.69E-01	1.08E-03	10.95	3.43	Clostridium_symbiosum_95	Bacteria_Firmicutes_Clostridia_Clostridiales_Lachnospiraceae_99_Clostridium XlVa_67
OTU_3684	7.07E-01	9.85E-01	2.42E-02	7.39	4.75	Blautia_hominis_97	Bacteria_Firmicutes_Clostridia_Clostridiales_Lachnospiraceae_Enterocloster_48
OTU_945	9.67E-01	6.63E-01	3.36E-03	2.88	8.44	Veillonella_denticariosi_91	Bacteria_Firmicutes_Bacilli_54_Lactobacillales_29_Carnobacteriaceae_29_Isobaculum_23
OTU_27019	9.87E-01	6.04E-01	7.34E-01	5.68	5.96	Pseudescherichia_vulneris_96	Bacteria_Proteobacteria_Gammaproteobacteria_Enterobacterales_Enterobacteriaceae_Escherichia/Shigella_74
OTU_913	8.82E-01	6.04E-01	2.85E-04	9.12	3.59	Escherichia_albertii_92	Bacteria_Proteobacteria_94_Gammaproteobacteria_94_Enterobacterales_93_Enterobacteriaceae_93_Pseudescherichia_88
OTU_9851	7.12E-01	8.50E-01	1.64E-04	8.46	3.68	Pseudescherichia_vulneris_95	Bacteria_Proteobacteria_95_Gammaproteobacteria_95_Enterobacterales_94_Enterobacteriaceae_94_Pseudescherichia_86
OTU_1058	9.04E-01	9.70E-01	4.11E-01	6.78	8.05	Escherichia_albertii_92	Bacteria_Proteobacteria_90_Gammaproteobacteria_90_Enterobacterales_90_Enterobacteriaceae_90_Pseudescherichia_89
OTU_181	7.46E-01	8.51E-01	9.28E-01	2.02	2.11	Coprococcus_catus_100	Bacteria_Firmicutes_Clostridia_Clostridiales_Lachnospiraceae_Coprococcus_98
OTU_193	8.54E-01	6.04E-01	3.30E-01	3.13	6.32	Lancefieldella_parvulum_100	Bacteria_Actinobacteria_Coriobacteriia_Coriobacteriales_Atopobiaceae_Lancefieldella_100
OTU_847	7.86E-01	7.73E-01	2.54E-01	5.55	7.29	Veillonella_dispar_98	Bacteria_Firmicutes_Negativicutes_75_Veillonellales_56_Veillonellaceae_56_Veillonella_54
OTU_222	7.07E-01	6.07E-01	2.94E-01	3.94	1.04	Slackia_isoflavoniconvertens_96	Bacteria_Actinobacteria_Coriobacteriia_Eggerthellales_99_Eggerthellaceae_99_Slackia_99
OTU_1121	1.00E+00	8.78E-01	2.64E-02	5.02	7.78	Enterococcus_dispar_99	Bacteria_Firmicutes_83_Bacilli_83_Lactobacillales_80_Carnobacteriaceae_63_Isobaculum_48
OTU_28022	8.44E-01	7.55E-01	1.02E-03	12.54	2.06	Bacteroides_xylanisolvens_98	Bacteria_Bacteroidetes_Bacteroidia_Bacteroidales_Bacteroidaceae_Bacteroides_100
OTU_178	9.78E-01	6.06E-01	2.09E-03	2.54	0.33	Odoribacter_splanchnicus_99	Bacteria_Bacteroidetes_Bacteroidia_Bacteroidales_Odoribacteraceae_Odoribacter_100

OTU_1468	8.54E-01	6.72E-01	2.69E-04	1.21	11.15	Bacteroides_thetaiotaomicron_95	Bacteria_Bacteroidetes_Bacteroidia_Bacteroidales_Bacteroidaceae_Bacteroides_87
OTU_4647	7.07E-01	6.14E-01	8.45E-02	12.78	0.47	Streptococcus_peroris_98	Bacteria_Firmicutes_Bacilli_Lactobacillales_Streptococcaceae_Streptococcus_100
OTU_3912	9.86E-01	9.90E-01	7.24E-03	2.42	8.67	Bacteroides_fragilis_97	Bacteria_Bacteroidetes_Bacteroidia_Bacteroidales_Bacteroidaceae_Bacteroides_100
OTU_10126	7.57E-01	8.14E-01	6.77E-04	8.41	3.74	Hungatella_effluvii_97	Bacteria_Firmicutes_Clostridia_Clostridiales_Lachnospiraceae_Hungatella_74
OTU_759	9.82E-01	8.22E-01	3.57E-03	2.63	8.60	Phascolarctobacterium_faecium_96	Bacteria_Firmicutes_84_Negativicutes_84_Acidaminococcales_84_Acidaminococcaceae_84_Phascolarctobacterium_84
OTU_846	7.07E-01	9.66E-01	9.64E-01	5.66	5.60	Shigella_dysenteriae_97	Bacteria_Proteobacteria_Gammaproteobacteria_Enterobacterales_Enterobacteriaceae_Escherichia/Shigella_68
OTU_663	8.06E-01	6.53E-01	3.00E-02	9.47	2.60	Sutterella_massiliensis_99	Bacteria_Proteobacteria_91_Betaproteobacteria_91_Burkholderiales_91_Sutterellaceae_89_Sutterella_89
OTU_870	9.64E-01	8.73E-01	1.46E-02	3.63	6.35	Veillonella_parvula_98	Bacteria_Firmicutes_Negativicutes_73_Veillonellales_71_Veillonellaceae_71_Veillonella_70
OTU_8409	8.06E-01	8.16E-01	8.43E-01	5.78	5.61	Enterocloster_citroniae_97	Bacteria_Firmicutes_Clostridia_Clostridiales_Lachnospiraceae_Enterocloster_86
OTU_715	7.85E-01	6.04E-01	5.06E-01	5.02	4.01	Enterocloster_citroniae_93	Bacteria_Firmicutes_99_Clostridia_99_Clostridiales_99_Lachnospiraceae_99_Enterocloster_92
OTU_857	8.97E-01	9.06E-01	5.27E-01	5.89	6.50	Escherichia_coli_92	Bacteria_Proteobacteria_99_Gammaproteobacteria_99_Enterobacterales_98_Enterobacteriaceae_98_Pseudescherichia_97
OTU_514	7.74E-01	9.41E-01	7.65E-01	3.87	3.58	Veillonella_parvula_100	Bacteria_Firmicutes_95_Negativicutes_90_Veillonellales_81_Veillonellaceae_81_Veillonella_80
OTU_271	7.11E-01	8.83E-01	2.09E-01	5.00	3.69	Hespellia_porcina_98	Bacteria_Firmicutes_Clostridia_Clostridiales_Lachnospiraceae_Faecalicatena_41
OTU_1217	9.06E-01	9.37E-01	1.21E-04	2.45	7.46	Filobacillus_milosensis_88	Bacteria_Firmicutes_98_Bacilli_96_Lactobacillales_74_Carnobacteriaceae_74_Isobaculum_65
OTU_1136	7.46E-01	6.04E-01	4.49E-04	9.86	2.72	Clostridium_perfringens_96	Bacteria_Firmicutes_99_Clostridia_99_Clostridiales_99_Clostridiaceae 1_98_Sarcina_42
OTU_946	7.45E-01	6.04E-01	1.15E-01	4.75	6.99	Romboutsia_timonensis_95	Bacteria_Firmicutes_Clostridia_97_Clostridiales_97_Peptostreptococcaceae_97_Romboutsia_29
OTU_1010	8.29E-01	9.90E-01	5.11E-06	11.50	1.85	Clostridium_gasigenes_88	Bacteria_Bacteroidetes_78_Bacteroidia_78_Bacteroidales_78_Bacteroidaceae_78_Mediterranea_65
OTU_1321	7.07E-01	7.16E-01	2.58E-01	6.74	5.22	Dorea_longicatena_97	Bacteria_Firmicutes_92_Clostridia_92_Clostridiales_92_Lachnospiraceae_92_Dorea_86
OTU_955	7.85E-01	6.04E-01	2.73E-03	8.89	3.38	Enterococcus_dispar_98	Bacteria_Firmicutes_62_Bacilli_62_Lactobacillales_60_Carnobacteriaceae_52_Isobaculum_32
OTU_208	8.25E-01	6.63E-01	8.83E-02	15.96	0.20	Flintibacter_butyricus_91	Bacteria_Firmicutes_Clostridia_Clostridiales_Ruminococcaceae_94_Flintibacter_53
OTU_599	7.10E-01	9.46E-01	9.37E-01	2.63	2.71	Collinsella_aerofaciens_100	Bacteria_Actinobacteria_93_Coriobacteriia_93_Coriobacteriales_92_Coriobacteriaceae_91_Collinsella_86
OTU_623	7.59E-01	6.04E-01	2.15E-01	7.49	3.03	Fusobacterium_mortiferum_90	Bacteria_Fusobacteria_85_Fusobacteriia_85_Fusobacteriales_85_Fusobacteriaceae_85_Cetobacterium_55
OTU_829	9.08E-01	9.27E-01	1.32E-02	2.64	8.51	Bacteroides_fragilis_95	Bacteria_Bacteroidetes_Bacteroidia_Bacteroidales_Bacteroidaceae_Bacteroides_100
OTU_1963	9.31E-01	8.28E-01	2.89E-03	3.55	6.27	Hungatella_hathewayi_89	Bacteria_Firmicutes_99_Bacilli_57_Lactobacillales_53_Carnobacteriaceae_52_Isobaculum_44
OTU_248	7.58E-01	9.20E-01	2.12E-01	9.87	0.92	Bacteroides_stercoris_97	Bacteria_Bacteroidetes_Bacteroidia_Bacteroidales_Bacteroidaceae_Bacteroides_82
OTU_636	7.07E-01	9.97E-01	1.14E-04	9.39	1.52	Streptococcus_gordonii_100	Bacteria_Proteobacteria_91_Gammaproteobacteria_91_Enterobacterales_89_Enterobacteriaceae_83_Pseudescherichia_83
OTU_195	7.07E-01	6.04E-01	2.10E-01	6.79	0.63	Bacteroides_eggerthii_100	Bacteria_Bacteroidetes_Bacteroidia_Bacteroidales_Bacteroidaceae_Bacteroides_100
OTU_1234	9.58E-01	8.65E-01	2.63E-02	3.73	6.12	Escherichia_albertii_95	Bacteria_Proteobacteria_Gammaproteobacteria_Enterobacterales_Enterobacteriaceae_Pseudescherichia_58
OTU_358	9.45E-01	8.63E-01	5.62E-02	2.61	7.02	Clostridium_sartagoforme_89	Bacteria_Proteobacteria_72_Gammaproteobacteria_72_Enterobacterales_71_Enterobacteriaceae_71_Pseudescherichia_66
OTU_11352	9.32E-01	6.14E-01	6.56E-01	6.40	5.88	Roseburia_inulinivorans_90	Bacteria_Firmicutes_Clostridia_69_Clostridiales_69_Lachnospiraceae_69_Bariatricus_35
OTU_133	NA	9.69E-01	6.30E-02	0.00	5.27	Methylophilus_luteus_100	Bacteria_Proteobacteria_Betaproteobacteria_Nitrosomonadales_Methylophilaceae_Methylophilus_100
OTU_2266	9.08E-01	7.11E-01	4.59E-02	2.78	6.65	Enterocloster_asparagiformis_97	Bacteria_Firmicutes_Clostridia_Clostridiales_Lachnospiraceae_Enterocloster_49
OTU_3389	9.08E-01	7.36E-01	5.05E-02	7.36	4.58	Kineothrix_alysoides_95	Bacteria_Firmicutes_Clostridia_Clostridiales_Lachnospiraceae_Clostridium XlVa_66
OTU_1366	7.07E-01	7.31E-01	4.26E-08	9.33	2.08	Bacteroides_uniformis_98	Bacteria_Bacteroidetes_70_Bacteroidia_70_Bacteroidales_70_Bacteroidaceae_70_Bacteroides_47
OTU_624	7.07E-01	6.44E-01	1.06E-01	3.14	5.10	Veillonella_magna_91	Bacteria_Firmicutes_Negativicutes_Veillonellales_70_Veillonellaceae_70_Veillonella_70
OTU_1371	9.46E-01	6.04E-01	5.97E-01	5.38	5.98	Clostridium_symbiosum_95	Bacteria_Firmicutes_Clostridia_Clostridiales_Lachnospiraceae_Clostridium XlVa_56
OTU_950	7.07E-01	6.72E-01	1.67E-01	8.21	0.65	Veillonella_atypica_90	Bacteria_Firmicutes_Negativicutes_83_Selenomonadales_39_Sporomusaceae_36_Anaerosinus_33
OTU_156	7.57E-01	6.14E-01	7.91E-02	0.85	0.19	Alistipes_onderdonkii_100	Bacteria_Bacteroidetes_Bacteroidia_Bacteroidales_Rikenellaceae_Alistipes_100
OTU_435	8.94E-01	7.31E-01	1.68E-02	9.63	2.03	Sutterella_massiliensis_97	Bacteria_Proteobacteria_Betaproteobacteria_Burkholderiales_Sutterellaceae_Sutterella_100
OTU_694	7.07E-01	6.04E-01	6.58E-04	8.93	2.97	Clostridium_perfringens_98	Bacteria_Firmicutes_Erysipelotrichia_68_Erysipelotrichales_68_Erysipelatoclostridiaceae_68_Erysipelatoclostridium_68
OTU_917	9.59E-01	9.65E-01	1.36E-01	4.47	6.60	Enterococcus_hirae_96	Bacteria_Firmicutes_Bacilli_Lactobacillales_Enterococcaceae_83_Vagococcus_42
OTU_15680	1.00E+00	6.83E-01	2.76E-02	6.53	4.09	Enterococcus_faecalis_98	Bacteria_Firmicutes_Bacilli_Lactobacillales_Enterococcaceae_Enterococcus_96
OTU_1487	9.65E-01	6.72E-01	9.89E-01	6.39	6.38	Roseburia_inulinivorans_97	Bacteria_Firmicutes_Clostridia_Clostridiales_Lachnospiraceae_Roseburia_97
OTU_1497	7.82E-01	8.36E-01	1.61E-02	3.48	6.22	Escherichia_albertii_92	Bacteria_Proteobacteria_95_Gammaproteobacteria_95_Enterobacterales_95_Enterobacteriaceae_95_Pseudescherichia_87
OTU_635	9.67E-01	6.04E-01	1.72E-03	10.41	0.95	Streptococcus_gordonii_91	Bacteria_Firmicutes_Bacilli_95_Lactobacillales_88_Streptococcaceae_49_Streptococcus_43
OTU_455	9.06E-01	9.77E-01	2.69E-03	1.32	9.05	Pseudescherichia_vulneris_99	Bacteria_Proteobacteria_64_Gammaproteobacteria_64_Enterobacterales_64_Enterobacteriaceae_64_Pseudescherichia_63
OTU_745	8.61E-01	6.81E-01	2.33E-04	12.29	1.01	Streptococcus_cristatus_96	Bacteria_Firmicutes_Bacilli_Lactobacillales_Streptococcaceae_46_Streptococcus_46
OTU_590	7.07E-01	6.04E-01	5.91E-01	4.91	6.93	Dialister_succinatiphilus_89	Bacteria_Proteobacteria_70_Gammaproteobacteria_70_Enterobacterales_70_Enterobacteriaceae_68_Pseudescherichia_68
OTU_607	8.12E-01	6.14E-01	5.10E-01	6.44	4.97	Enterococcus_hirae_91	Bacteria_Firmicutes_Clostridia_77_Clostridiales_77_Lachnospiraceae_76_Merdimonas_17
OTU_787	9.53E-01	9.46E-01	5.01E-01	5.71	6.84	Roseburia_inulinivorans_93	Bacteria_Firmicutes_Clostridia_93_Clostridiales_93_Lachnospiraceae_92_Ruminococcus2_7
OTU_20240	8.25E-01	6.04E-01	2.77E-06	9.44	3.37	Bacteroides_uniformis_97	Bacteria_Bacteroidetes_Bacteroidia_Bacteroidales_Bacteroidaceae_Bacteroides_100
OTU_3901	7.12E-01	8.28E-01	1.64E-04	3.40	6.30	Enterocloster_citroniae_93	Bacteria_Firmicutes_97_Clostridia_97_Clostridiales_97_Lachnospiraceae_97_Enterocloster_80
OTU_1488	1.00E+00	8.53E-01	1.32E-02	4.29	7.13	Enterocloster_clostridioformis_98	Bacteria_Firmicutes_84_Clostridia_84_Clostridiales_84_Lachnospiraceae_84_Enterocloster_44
OTU_8474	9.38E-01	9.66E-01	2.35E-02	7.97	4.71	Bacteroides_xylanisolvens_97	Bacteria_Bacteroidetes_Bacteroidia_Bacteroidales_Bacteroidaceae_Bacteroides_100
OTU_1381	7.07E-01	5.62E-01	2.00E-02	6.95	2.89	Veillonella_atypica_96	Bacteria_Firmicutes_Negativicutes_Veillonellales_Veillonellaceae_Veillonella_100
OTU_4905	7.07E-01	6.50E-01	6.51E-01	5.59	3.96	Kosakonia_quasisacchari_97	Bacteria_Proteobacteria_Gammaproteobacteria_Enterobacterales_Enterobacteriaceae_94_Leclercia_21
OTU_425	7.07E-01	7.77E-01	9.35E-01	4.83	5.03	Serratia_marcescens_91	Bacteria_Proteobacteria_Gammaproteobacteria_95_Enterobacterales_95_Enterobacteriaceae_95_Pseudescherichia_83
OTU_277	7.07E-01	6.47E-01	7.73E-03	0.20	0.91	Actinobacillus_porcinus_96	Bacteria_Proteobacteria_Gammaproteobacteria_Pasteurellales_94_Pasteurellaceae_94_Conservatibacter_24
OTU_7373	9.75E-01	9.86E-01	4.58E-03	3.62	5.94	Enterocloster_citroniae_97	Bacteria_Firmicutes_Clostridia_Clostridiales_Lachnospiraceae_Enterocloster_99
OTU_1742	8.06E-01	8.72E-01	1.68E-02	2.78	7.45	Faecalibacterium_prausnitzii_94	Bacteria_Firmicutes_Clostridia_Clostridiales_Ruminococcaceae_98_Faecalibacterium_54
OTU_1420	7.68E-01	9.70E-01	6.55E-04	4.23	0.91	Bifidobacterium_callitrichidarum_92	Bacteria_Actinobacteria_98_Actinobacteria_98_Bifidobacteriales_98_Bifidobacteriaceae_98_Pseudoscardovia_41
OTU_239	9.99E-01	7.32E-01	3.66E-05	6.63	2.15	Eubacterium_ventriosum_99	Bacteria_Firmicutes_Clostridia_Clostridiales_Lachnospiraceae_Lachnospiracea_incertae_sedis_100
OTU_11560	8.33E-01	7.97E-01	1.71E-02	7.22	3.38	Bacteroides_xylanisolvens_98	Bacteria_Bacteroidetes_Bacteroidia_Bacteroidales_Bacteroidaceae_Bacteroides_100
OTU_1375	8.06E-01	6.04E-01	8.12E-01	4.84	5.22	Clostridium_symbiosum_96	Bacteria_Firmicutes_Clostridia_Clostridiales_Lachnospiraceae_Clostridium XlVa_69
OTU_709	7.07E-01	7.73E-01	3.77E-02	3.30	4.96	Escherichia_albertii_90	Bacteria_Proteobacteria_58_Gammaproteobacteria_58_Enterobacterales_58_Enterobacteriaceae_58_Pseudescherichia_56
OTU_1945	7.57E-01	8.77E-01	6.47E-02	4.99	7.55	Pseudescherichia_vulneris_97	Bacteria_Proteobacteria_Gammaproteobacteria_Enterobacterales_Enterobacteriaceae_Escherichia/Shigella_76
OTU_532	9.31E-01	9.25E-01	1.14E-04	7.15	2.02	Intestinibacillus_massiliensis_100	Bacteria_Firmicutes_Clostridia_Clostridiales_Eubacteriaceae_90_Intestinibacillus_90

OTU_958	7.07E-01	6.04E-01	1.91E-05	5.66	0.69	Parabacteroides_distasonis_96	Bacteria_Bacteroidetes_Bacteroidia_Bacteroidales_Porphyromonadaceae_96_Parabacteroides_96
OTU_127	9.69E-01	6.81E-01	2.87E-02	2.24	0.71	Barnesiella_intestinihominis_100	Bacteria_Bacteroidetes_Bacteroidia_Bacteroidales_Barnesiellaceae_Barnesiella_100
OTU_24618	9.69E-01	9.12E-01	8.72E-04	4.05	8.32	Bacteroides_faecis_97	Bacteria_Bacteroidetes_Bacteroidia_Bacteroidales_Bacteroidaceae_Bacteroides_100
OTU_351	7.07E-01	6.04E-01	7.58E-02	2.91	6.53	Pseudescherichia_vulneris_97	Bacteria_Proteobacteria_82_Gammaproteobacteria_82_Enterobacterales_81_Enterobacteriaceae_81_Pseudescherichia_62
OTU_922	9.93E-01	7.44E-01	8.90E-02	2.82	6.22	Pseudescherichia_vulneris_98	Bacteria_Proteobacteria_99_Gammaproteobacteria_99_Enterobacterales_98_Enterobacteriaceae_98_Pseudescherichia_75
OTU_2833	9.73E-01	8.51E-01	1.84E-01	5.23	3.62	Lachnoclostridium_pacaense_98	Bacteria_Firmicutes_Clostridia_Clostridiales_Lachnospiraceae_Clostridium XlVa_61
OTU_5420	7.07E-01	7.24E-01	2.35E-04	9.38	1.66	Bacteroides_thetaiotaomicron_97	Bacteria_Bacteroidetes_Bacteroidia_Bacteroidales_Bacteroidaceae_Bacteroides_99
OTU_343	7.07E-01	9.77E-01	5.34E-02	3.78	1.13	Hungatella_effluvii_89	Bacteria_Firmicutes_Clostridia_Clostridiales_Lachnospiraceae_Merdimonas_18
OTU_225	1.00E+00	6.04E-01	1.60E-02	8.32	0.56	Eubacterium_coprostanoligenes_96	Bacteria_Firmicutes_Clostridia_99_Clostridiales_99_Ruminococcaceae_87_Paludicola_20
OTU_738	8.25E-01	8.75E-01	1.20E-02	2.10	5.86	Acidaminococcus_intestini_92	Bacteria_Firmicutes_Negativicutes_Acidaminococcales_Acidaminococcaceae_Phascolarctobacterium_100
OTU_3751	9.64E-01	6.10E-01	1.32E-02	7.25	2.10	Pseudescherichia_vulneris_91	Bacteria_Proteobacteria_Betaproteobacteria_90_Burkholderiales_90_Sutterellaceae_90_Sutterella_90
OTU_559	7.07E-01	9.61E-01	6.66E-04	1.43	8.66	Phascolarctobacterium_faecium_98	Bacteria_Firmicutes_Negativicutes_97_Acidaminococcales_97_Acidaminococcaceae_97_Phascolarctobacterium_97
OTU_1466	9.95E-01	6.72E-01	2.40E-01	6.07	4.61	Coprococcus_comes_96	Bacteria_Firmicutes_Clostridia_Clostridiales_Lachnospiraceae_Coprococcus_69
OTU_531	7.07E-01	6.47E-01	5.05E-02	3.14	0.65	Phocaeicola_vulgatus_96	Bacteria_Bacteroidetes_Bacteroidia_Bacteroidales_Bacteroidaceae_99_Phocaeicola_99
OTU_626	7.07E-01	9.70E-01	1.45E-02	10.42	1.72	Bacteroides_stercoris_100	Bacteria_Bacteroidetes_99_Bacteroidia_98_Bacteroidales_98_Bacteroidaceae_94_Mediterranea_54
OTU_107068	7.07E-01	8.17E-01	2.45E-01	8.60	1.12	Veillonella_tobetsuensis_98	Bacteria_Firmicutes_Negativicutes_Veillonellales_Veillonellaceae_Veillonella_100
OTU_4528	8.61E-01	6.04E-01	2.80E-01	6.30	4.96	Veillonella_tobetsuensis_96	Bacteria_Firmicutes_Negativicutes_97_Veillonellales_87_Veillonellaceae_87_Veillonella_83
OTU_819	7.07E-01	8.14E-01	6.11E-07	9.56	0.72	Bacteroides_uniformis_97	Bacteria_Bacteroidetes_Bacteroidia_Bacteroidales_Bacteroidaceae_Bacteroides_99
OTU_695	9.08E-01	6.07E-01	2.39E-02	6.12	1.78	Sutterella_wadsworthensis_99	Bacteria_Proteobacteria_91_Betaproteobacteria_91_Burkholderiales_91_Sutterellaceae_90_Sutterella_90
OTU_60649	9.64E-01	6.04E-01	1.49E-01	4.58	6.16	Shigella_dysenteriae_96	Bacteria_Proteobacteria_Gammaproteobacteria_Enterobacterales_Enterobacteriaceae_Pseudescherichia_68
OTU_3581	9.63E-01	7.08E-01	7.02E-01	5.27	4.92	Faecalicatena_orotica_97	Bacteria_Firmicutes_Clostridia_Clostridiales_Lachnospiraceae_Clostridium XlVa_94
OTU_21202	7.46E-01	8.51E-01	1.42E-01	4.49	2.50	Faecalibacterium_prausnitzii_99	Bacteria_Firmicutes_Clostridia_Clostridiales_Ruminococcaceae_Faecalibacterium_100
OTU_10599	9.10E-01	8.68E-01	3.60E-01	4.85	5.88	Coprococcus_comes_97	Bacteria_Firmicutes_Clostridia_Clostridiales_Lachnospiraceae_Coprococcus_59
OTU_42132	7.89E-01	7.49E-01	1.33E-04	11.16	0.90	Bacteroides_uniformis_96	Bacteria_Bacteroidetes_Bacteroidia_Bacteroidales_Bacteroidaceae_Bacteroides_100
OTU_1471	8.48E-01	6.40E-01	3.74E-02	4.79	1.49	Bacteroides_uniformis_95	Bacteria_Bacteroidetes_Bacteroidia_Bacteroidales_Bacteroidaceae_Phocaeicola_88
OTU_2107	7.84E-01	6.04E-01	1.45E-01	3.21	7.13	Lachnoclostridium_pacaense_96	Bacteria_Firmicutes_Clostridia_Clostridiales_Lachnospiraceae_Clostridium XlVa_30
OTU_136	7.57E-01	6.96E-01	1.25E-01	2.92	0.64	Mitsuokella_jalaludinii_100	Bacteria_Firmicutes_Negativicutes_Selenomonadales_Selenomonadaceae_Mitsuokella_100
OTU_431	7.91E-01	9.35E-01	1.23E-02	1.94	8.53	Bacteroides_fragilis_100	Bacteria_Bacteroidetes_94_Bacteroidia_94_Bacteroidales_94_Bacteroidaceae_92_Bacteroides_74
OTU_929	7.59E-01	8.42E-01	1.97E-02	1.22	7.49	Clostridium_tepidum_94	Bacteria_Firmicutes_Clostridia_Clostridiales_Lachnospiraceae_44_Mobilitalea_7
OTU_4955	9.99E-01	4.60E-01	4.12E-02	3.26	4.92	Enterococcus_faecalis_97	Bacteria_Firmicutes_Bacilli_Lactobacillales_Enterococcaceae_87_Enterococcus_64
OTU_29847	9.06E-01	6.04E-01	1.30E-03	2.37	5.64	Enterococcus_faecalis_98	Bacteria_Firmicutes_Bacilli_Lactobacillales_99_Enterococcaceae_89_Enterococcus_52
OTU_15669	7.07E-01	6.04E-01	9.78E-02	8.27	0.36	Streptococcus_cristatus_98	Bacteria_Firmicutes_Bacilli_Lactobacillales_Streptococcaceae_Streptococcus_100
OTU_564	7.07E-01	6.04E-01	4.99E-04	8.26	1.30	Streptococcus_gordonii_100	Bacteria_Firmicutes_Bacilli_75_Lactobacillales_74_Streptococcaceae_72_Streptococcus_71
OTU_392	7.85E-01	6.04E-01	2.33E-02	1.54	3.02	Enterococcus_faecalis_90	Bacteria_Firmicutes_Clostridia_99_Clostridiales_99_Ruminococcaceae_99_Flavonifractor_99
OTU_371	7.57E-01	7.51E-01	2.05E-02	1.31	7.61	Clostridium_symbiosum_92	Bacteria_Firmicutes_Clostridia_Clostridiales_Lachnospiraceae_92_Clostridium XlVa_49
OTU_3711	8.29E-01	8.83E-01	1.48E-03	5.71	2.79	Dorea_longicatena_96	Bacteria_Firmicutes_Clostridia_Clostridiales_Lachnospiraceae_Dorea_82
OTU_349	9.38E-01	6.04E-01	5.50E-01	5.00	4.26	Ruthenibacterium_lactatiformans_100	Bacteria_Firmicutes_Clostridia_Clostridiales_Ruminococcaceae_Ruthenibacterium_100
OTU_12625	9.94E-01	6.04E-01	5.87E-04	7.68	2.54	Bacteroides_thetaiotaomicron_97	Bacteria_Bacteroidetes_Bacteroidia_Bacteroidales_Bacteroidaceae_Bacteroides_100
OTU_62340	7.07E-01	6.04E-01	5.06E-01	3.03	1.30	Veillonella_rogosae_98	Bacteria_Firmicutes_Negativicutes_Veillonellales_Veillonellaceae_Veillonella_100
OTU_476	7.07E-01	8.14E-01	1.40E-01	2.19	0.85	Bifidobacterium_catenulatum_90	Bacteria_Actinobacteria_Actinobacteria_Bifidobacteriales_Bifidobacteriaceae_Neoscardovia_38
OTU_710	7.57E-01	7.77E-01	3.70E-02	2.61	4.00	Flavonifractor_plautii_97	Bacteria_Firmicutes_Clostridia_Clostridiales_Ruminococcaceae_Flavonifractor_100
OTU_4592	9.67E-01	7.99E-01	4.59E-01	5.32	4.80	Pseudescherichia_vulneris_96	Bacteria_Proteobacteria_71_Gammaproteobacteria_71_Enterobacterales_71_Enterobacteriaceae_71_Pseudescherichia_66
OTU_11535	9.84E-01	6.04E-01	3.99E-01	4.11	4.95	Clostridium_scindens_97	Bacteria_Firmicutes_Clostridia_Clostridiales_Lachnospiraceae_Clostridium XlVa_95
OTU_1804	7.07E-01	6.51E-01	1.54E-01	4.36	6.18	Bacteroides_faecis_96	Bacteria_Bacteroidetes_Bacteroidia_Bacteroidales_Bacteroidaceae_Bacteroides_100
OTU_387	9.08E-01	6.76E-01	1.69E-01	1.22	0.63	Bifidobacterium_stercoris_97	Bacteria_Actinobacteria_Actinobacteria_Bifidobacteriales_Bifidobacteriaceae_Bifidobacterium_97
OTU_924	8.97E-01	9.61E-01	1.46E-02	1.26	7.11	Enterococcus_dispar_100	Bacteria_Firmicutes_94_Bacilli_94_Lactobacillales_92_Carnobacteriaceae_71_Isobaculum_63
OTU_634	8.85E-01	9.25E-01	2.48E-02	8.78	0.74	Bacteroides_kribbi_95	Bacteria_Bacteroidetes_Bacteroidia_Bacteroidales_Bacteroidaceae_97_Bacteroides_97
OTU_1048	7.07E-01	9.12E-01	1.49E-01	2.46	0.65	Phocaeicola_vulgatus_97	Bacteria_Bacteroidetes_Bacteroidia_Bacteroidales_Bacteroidaceae_Bacteroides_61
OTU_312	7.07E-01	6.04E-01	5.75E-01	3.65	5.70	Hafnia_paralvei_99	Bacteria_Proteobacteria_Gammaproteobacteria_Enterobacterales_Hafniaceae_57_Hafnia_57
OTU_1465	9.65E-01	6.76E-01	1.28E-02	8.02	2.49	Escherichia_marmotae_94	Bacteria_Proteobacteria_Gammaproteobacteria_Enterobacterales_Enterobacteriaceae_99_Pseudescherichia_41
OTU_2173	8.49E-01	7.99E-01	5.35E-02	6.74	4.19	Bacteroides_xylanisolvens_98	Bacteria_Bacteroidetes_84_Bacteroidia_84_Bacteroidales_84_Bacteroidaceae_84_Bacteroides_72
OTU_671	9.62E-01	6.14E-01	2.71E-03	9.68	0.92	Streptococcus_gordonii_97	Bacteria_Firmicutes_Clostridia_67_Clostridiales_67_Lachnospiraceae_67_Bariatricus_19
OTU_132	7.07E-01	6.08E-01	3.21E-01	8.60	0.75	Harryflintia_acetispora_88	Bacteria_Firmicutes_99_Clostridia_98_Clostridiales_98_Ruminococcaceae_81_Harryflintia_34
OTU_1382	7.57E-01	8.33E-01	2.23E-01	3.80	5.04	Enterococcus_faecalis_90	Bacteria_Firmicutes_Clostridia_94_Clostridiales_94_Lachnospiraceae_94_Bariatricus_75
OTU_464	7.81E-01	6.04E-01	2.14E-02	12.00	0.65	Clostridium_tarantellae_98	Bacteria_Firmicutes_Clostridia_Clostridiales_Clostridiaceae 1_Clostridium sensu stricto_98
OTU_832	9.68E-01	9.04E-01	2.85E-02	1.67	6.47	Clostridium_tepidum_91	Bacteria_Firmicutes_Bacilli_92_Lactobacillales_87_Carnobacteriaceae_63_Isobaculum_60
OTU_918	7.57E-01	6.49E-01	3.91E-03	3.54	0.98	Phocaeicola_vulgatus_97	Bacteria_Bacteroidetes_Bacteroidia_Bacteroidales_Bacteroidaceae_Phocaeicola_99
OTU_1140	7.07E-01	9.98E-01	7.56E-01	3.87	4.34	Pseudescherichia_vulneris_97	Bacteria_Proteobacteria_Gammaproteobacteria_Enterobacterales_Enterobacteriaceae_Escherichia/Shigella_91
OTU_354	7.07E-01	9.85E-01	2.32E-02	3.18	0.09	Prevotella_copri_95	Bacteria_Bacteroidetes_Bacteroidia_Bacteroidales_Prevotellaceae_67_Prevotella_66
OTU_432	7.07E-01	8.50E-01	7.61E-02	2.24	0.60	Bifidobacterium_stercoris_95	Bacteria_Actinobacteria_Actinobacteria_97_Bifidobacteriales_95_Bifidobacteriaceae_95_Bifidobacterium_46
OTU_1943	9.06E-01	7.07E-01	9.43E-01	4.83	4.93	Clostridium_symbiosum_96	Bacteria_Firmicutes_Clostridia_Clostridiales_Lachnospiraceae_Clostridium XlVa_83
OTU_1028	7.07E-01	6.04E-01	9.05E-03	7.44	1.83	Clostridium_perfringens_96	Bacteria_Firmicutes_Clostridia_Clostridiales_Clostridiaceae 1_99_Clostridium sensu stricto_79
OTU_284	9.06E-01	6.04E-01	8.86E-01	4.48	4.24	Acidaminococcus_intestini_93	Bacteria_Firmicutes_Negativicutes_99_Acidaminococcales_98_Acidaminococcaceae_98_Acidaminococcus_98
OTU_309	7.45E-01	6.04E-01	4.93E-01	1.10	3.20	Bacteroides_mediterraneensis_99	Bacteria_Bacteroidetes_Bacteroidia_Bacteroidales_Bacteroidaceae_Phocaeicola_100
OTU_344	8.22E-01	9.10E-01	2.58E-01	7.87	0.61	Bacteroides_stercoris_96	Bacteria_Bacteroidetes_Bacteroidia_Bacteroidales_Bacteroidaceae_Phocaeicola_69
OTU_1164	8.06E-01	9.67E-01	1.29E-01	4.71	3.38	Enterococcus_dispar_100	Bacteria_Firmicutes_Bacilli_Lactobacillales_93_Carnobacteriaceae_68_Isobaculum_57
OTU_2913	9.14E-01	6.04E-01	3.61E-04	7.37	2.54	Falcatimonas_natans_94	Bacteria_Firmicutes_Clostridia_Clostridiales_Lachnospiraceae_92_Enterocloster_23

OTU_4301	9.38E-01	7.73E-01	5.66E-05	0.85	8.96	Bacteroides_xylanisolvens_97	Bacteria_Bacteroidetes_Bacteroidia_Bacteroidales_Bacteroidaceae_Bacteroides_100
OTU_1115	9.67E-01	6.04E-01	6.36E-01	4.39	5.39	Acidaminococcus_fermentans_94	Bacteria_Firmicutes_Negativicutes_Acidaminococcales_Acidaminococcaceae_Acidaminococcus_100
OTU_1157	9.06E-01	6.04E-01	5.52E-01	1.01	1.48	Phocaeicola_coprocola_97	Bacteria_Bacteroidetes_Bacteroidia_Bacteroidales_Bacteroidaceae_Phocaeicola_77
OTU_1415	7.82E-01	7.94E-01	9.79E-03	3.28	5.86	Shigella_dysenteriae_91	Bacteria_Proteobacteria_96_Gammaproteobacteria_96_Enterobacterales_96_Enterobacteriaceae_96_Pseudescherichia_96
OTU_1890	7.59E-01	6.16E-01	2.66E-03	6.59	2.69	Clostridium_perfringens_96	Bacteria_Firmicutes_Clostridia_75_Clostridiales_75_Clostridiaceae 1_75_Sarcina_26
OTU_408	9.14E-01	6.05E-01	1.69E-01	0.98	1.47	Fusicatenibacter_saccharivorans_91	Bacteria_Firmicutes_99_Clostridia_99_Clostridiales_99_Lachnospiraceae_98_Merdimonas_12
OTU_1020	9.38E-01	6.04E-01	1.72E-01	2.53	5.71	Clostridium_tarantellae_98	Bacteria_Firmicutes_Clostridia_Clostridiales_Clostridiaceae 1_Clostridium sensu stricto_100
OTU_206	NA	7.25E-01	1.10E-01	0.00	7.34	Azohydromonas_lata_99	Bacteria_Proteobacteria_Betaproteobacteria_Burkholderiales_Alcaligenaceae_68_Azohydromonas_68
OTU_9303	7.12E-01	6.14E-01	3.54E-06	7.37	2.25	Bacteroides_uniformis_96	Bacteria_Bacteroidetes_Bacteroidia_Bacteroidales_Bacteroidaceae_Bacteroides_100
OTU_2318	7.07E-01	9.90E-01	4.33E-01	3.12	4.04	Phocaeicola_coprophilus_95	Bacteria_Bacteroidetes_Bacteroidia_Bacteroidales_Bacteroidaceae_Phocaeicola_64
OTU_27424	9.98E-01	7.66E-01	3.78E-01	4.53	6.60	Bacteroides_thetaiotaomicron_98	Bacteria_Bacteroidetes_Bacteroidia_Bacteroidales_Bacteroidaceae_Bacteroides_100
OTU_2329	9.08E-01	8.65E-01	1.68E-02	3.37	5.24	Pseudescherichia_vulneris_92	Bacteria_Proteobacteria_Gammaproteobacteria_Enterobacterales_Enterobacteriaceae_97_Escherichia/Shigella_79
OTU_236	9.60E-01	9.98E-01	5.92E-01	0.37	0.25	Collinsella_aerofaciens_100	Bacteria_Actinobacteria_85_Coriobacteriia_85_Coriobacteriales_84_Coriobacteriaceae_82_Collinsella_80
OTU_427	8.47E-01	8.12E-01	1.35E-03	8.32	1.11	Clostridium_perfringens_91	Bacteria_Firmicutes_99_Clostridia_99_Clostridiales_99_Clostridiaceae 1_99_Desnuesiella_39
OTU_488	8.25E-01	9.95E-01	2.42E-02	9.02	1.64	Sutterella_massiliensis_98	Bacteria_Proteobacteria_98_Betaproteobacteria_98_Burkholderiales_97_Sutterellaceae_92_Sutterella_92
OTU_5063	8.16E-01	8.10E-01	2.72E-01	5.26	4.32	Bacteroides_kribbi_97	Bacteria_Bacteroidetes_52_Bacteroidia_52_Bacteroidales_52_Bacteroidaceae_52_Mediterranea_27
OTU_1101	8.06E-01	8.65E-01	3.45E-02	10.43	1.00	Clostridium_perfringens_97	Bacteria_Firmicutes_Clostridia_Clostridiales_Clostridiaceae 1_Clostridium sensu stricto_100
OTU_252	9.16E-01	6.05E-01	6.70E-02	6.01	1.15	Parabacteroides_goldsteinii_100	Bacteria_Bacteroidetes_Bacteroidia_Bacteroidales_Porphyromonadaceae_Parabacteroides_100
OTU_770	8.57E-01	9.70E-01	5.10E-03	8.59	1.42	Clostridium_perfringens_97	Bacteria_Firmicutes_Clostridia_Clostridiales_Clostridiaceae 1_98_Sarcina_23
OTU_9673	7.19E-01	9.48E-01	2.14E-01	4.54	5.37	Shigella_dysenteriae_95	Bacteria_Proteobacteria_Gammaproteobacteria_Enterobacterales_Enterobacteriaceae_Pseudescherichia_74
OTU_27527	7.07E-01	6.04E-01	9.78E-02	1.97	4.74	Lachnoclostridium_pacaense_98	Bacteria_Firmicutes_Clostridia_Clostridiales_Lachnospiraceae_Enterocloster_44
OTU_32056	8.30E-01	9.69E-01	1.04E-02	7.08	3.54	Bacteroides_xylanisolvens_98	Bacteria_Bacteroidetes_Bacteroidia_Bacteroidales_Bacteroidaceae_Bacteroides_100
OTU_20257	7.12E-01	9.54E-01	4.33E-02	4.23	6.47	Bacteroides_ovatus_97	Bacteria_Bacteroidetes_Bacteroidia_Bacteroidales_Bacteroidaceae_Bacteroides_100
OTU_438	7.91E-01	6.10E-01	6.15E-01	1.72	2.54	Collinsella_aerofaciens_96	Bacteria_Actinobacteria_Coriobacteriia_Coriobacteriales_Coriobacteriaceae_98_Collinsella_74
OTU_511	9.41E-01	7.73E-01	2.07E-02	0.62	2.87	Phocaeicola_coprophilus_95	Bacteria_Bacteroidetes_Bacteroidia_Bacteroidales_Bacteroidaceae_Phocaeicola_55
OTU_213	9.67E-01	6.04E-01	4.63E-02	7.83	0.77	Faecalicatena_orotica_96	Bacteria_Firmicutes_Clostridia_Clostridiales_Lachnospiraceae_Schaedlerella_100
OTU_459	1.00E+00	8.68E-01	3.45E-01	0.46	0.76	Bifidobacterium_longum_92	Bacteria_Actinobacteria_98_Actinobacteria_98_Bifidobacteriales_98_Bifidobacteriaceae_98_Pseudoscardovia_33
OTU_840	7.07E-01	8.26E-01	5.87E-04	7.52	1.32	Streptococcus_lactarius_98	Bacteria_Firmicutes_Bacilli_Lactobacillales_Streptococcaceae_Streptococcus_100
OTU_10434	8.97E-01	8.11E-01	8.15E-01	4.66	4.31	Clostridium_symbiosum_96	Bacteria_Firmicutes_Clostridia_Clostridiales_Lachnospiraceae_Clostridium XlVa_47
OTU_3655	7.81E-01	6.04E-01	1.29E-02	5.60	1.77	Bifidobacterium_saguini_91	Bacteria_Actinobacteria_84_Actinobacteria_84_Bifidobacteriales_84_Bifidobacteriaceae_84_Pseudoscardovia_57
OTU_418	7.07E-01	8.34E-01	3.01E-02	2.27	0.57	Veillonella_parvula_90	Bacteria_Firmicutes_95_Negativicutes_95_Veillonellales_92_Veillonellaceae_92_Veillonella_91
OTU_20196	7.57E-01	9.25E-01	1.18E-02	3.16	4.61	Pseudescherichia_vulneris_97	Bacteria_Proteobacteria_Gammaproteobacteria_Enterobacterales_Enterobacteriaceae_Escherichia/Shigella_50
OTU_4045	8.06E-01	6.72E-01	4.30E-01	2.37	1.86	Faecalibacterium_prausnitzii_98	Bacteria_Firmicutes_Clostridia_Clostridiales_Ruminococcaceae_Faecalibacterium_100
OTU_731	7.07E-01	6.16E-01	3.83E-02	0.29	0.86	Kluyvera_cryocrescens_95	Bacteria_Proteobacteria_Gammaproteobacteria_Enterobacterales_98_Enterobacteriaceae_97_Lelliottia_33
OTU_923	7.85E-01	8.75E-01	6.01E-02	3.22	5.15	Clostridium_symbiosum_94	Bacteria_Firmicutes_Clostridia_Clostridiales_Lachnospiraceae_Clostridium XlVa_57
OTU_843	9.49E-01	7.40E-01	8.89E-01	2.97	2.84	Flintibacter_butyricus_93	Bacteria_Firmicutes_Clostridia_Clostridiales_Lachnospiraceae_54_Merdimonas_15
OTU_401	7.11E-01	6.04E-01	1.10E-01	6.53	1.04	Anaerotaenia_torta_98	Bacteria_Firmicutes_Clostridia_Clostridiales_Lachnospiraceae_Herbinix_49
OTU_340	7.07E-01	6.60E-01	2.16E-01	5.89	2.37	Collinsella_aerofaciens_94	Bacteria_Actinobacteria_98_Coriobacteriia_98_Coriobacteriales_98_Coriobacteriaceae_97_Collinsella_97
OTU_784	9.87E-01	7.31E-01	1.76E-03	2.99	6.18	Escherichia_coli_90	Bacteria_Proteobacteria_95_Gammaproteobacteria_95_Enterobacterales_95_Enterobacteriaceae_94_Pseudescherichia_93
OTU_1068	1.00E+00	9.09E-01	1.67E-03	0.97	3.88	Phocaeicola_coprophilus_98	Bacteria_Bacteroidetes_89_Bacteroidia_89_Bacteroidales_89_Bacteroidaceae_89_Phocaeicola_83
OTU_1432	8.17E-01	6.04E-01	1.35E-02	6.53	2.79	Peptoniphilus_harei_96	Bacteria_Firmicutes_Clostridia_Clostridiales_Peptoniphilaceae_Peptoniphilus_100
OTU_3324	9.87E-01	7.20E-01	4.12E-03	3.03	5.47	Enterococcus_faecalis_97	Bacteria_Firmicutes_Bacilli_99_Lactobacillales_98_Carnobacteriaceae_39_Isobaculum_32
OTU_1368	7.75E-01	6.58E-01	1.32E-03	6.75	2.06	Clostridium_tarantellae_92	Bacteria_Firmicutes_Clostridia_Clostridiales_Lachnospiraceae_76_Mobilitalea_15
OTU_217	9.42E-01	6.04E-01	4.47E-01	1.15	1.64	Prevotella_oralis_91	Bacteria_Bacteroidetes_Bacteroidia_Bacteroidales_Prevotellaceae_Prevotella_100
OTU_1008	1.00E+00	7.11E-01	1.89E-06	8.67	0.95	Bacteroides_xylanisolvens_96	Bacteria_Bacteroidetes_Bacteroidia_Bacteroidales_Bacteroidaceae_Bacteroides_100
OTU_4745	9.38E-01	6.04E-01	2.96E-03	7.20	1.98	Enterococcus_thailandicus_93	Bacteria_Firmicutes_Bacilli_61_Lactobacillales_60_Carnobacteriaceae_20_Isobaculum_8
OTU_595	7.91E-01	6.04E-01	3.72E-01	2.31	3.22	Clostridium_polysaccharolyticum_92	Bacteria_Firmicutes_99_Clostridia_99_Clostridiales_99_Lachnospiraceae_67_Anaerotaenia_10
OTU_2162	9.64E-01	9.18E-01	6.91E-04	7.50	2.35	Bacteroides_acidifaciens_96	Bacteria_Bacteroidetes_Bacteroidia_Bacteroidales_Bacteroidaceae_Bacteroides_100
OTU_457	9.60E-01	9.25E-01	8.55E-01	1.36	1.24	Faecalibacterium_prausnitzii_91	Bacteria_Firmicutes_Clostridia_Clostridiales_Lachnospiraceae_71_Merdimonas_15
OTU_1027	7.73E-01	6.04E-01	1.84E-01	4.56	3.13	Enterococcus_dispar_98	Bacteria_Firmicutes_Bacilli_97_Lactobacillales_91_Carnobacteriaceae_61_Isobaculum_59
OTU_1045	9.92E-01	8.83E-01	3.05E-02	7.41	3.15	Bacteroides_cellulosilyticus_88	Bacteria_Firmicutes_52_Clostridia_52_Clostridiales_52_Lachnospiraceae_52_Lactonifactor_17
OTU_1195	7.07E-01	6.04E-01	6.70E-02	3.54	0.84	Bacteroides_uniformis_96	Bacteria_Bacteroidetes_Bacteroidia_Bacteroidales_Bacteroidaceae_Phocaeicola_56
OTU_485	7.81E-01	9.46E-01	8.44E-03	4.71	0.37	Parabacteroides_distasonis_95	Bacteria_Bacteroidetes_Bacteroidia_Bacteroidales_Porphyromonadaceae_89_Parabacteroides_89
OTU_1436	7.07E-01	7.23E-01	3.52E-03	7.50	2.59	Roseburia_inulinivorans_97	Bacteria_Firmicutes_Clostridia_Clostridiales_Lachnospiraceae_Roseburia_73
OTU_934	7.57E-01	9.60E-01	9.37E-02	5.27	2.51	Collinsella_aerofaciens_100	Bacteria_Actinobacteria_88_Coriobacteriia_88_Coriobacteriales_88_Coriobacteriaceae_86_Collinsella_83
OTU_1377	7.07E-01	6.66E-01	1.84E-03	6.41	1.68	Streptococcus_mitis_98	Bacteria_Firmicutes_Bacilli_Lactobacillales_Streptococcaceae_Streptococcus_100
OTU_4225	9.08E-01	7.20E-01	7.42E-02	3.21	5.00	Pseudescherichia_vulneris_90	Bacteria_Proteobacteria_62_Gammaproteobacteria_62_Enterobacterales_62_Enterobacteriaceae_61_Pseudescherichia_61
OTU_1414	9.66E-01	6.04E-01	4.78E-01	4.08	3.47	Clostridium_symbiosum_92	Bacteria_Firmicutes_Clostridia_Clostridiales_Lachnospiraceae_53_Clostridium XlVa_30
OTU_1799	7.07E-01	7.73E-01	5.76E-03	5.68	2.68	Enterocloster_asparagiformis_95	Bacteria_Firmicutes_Clostridia_Clostridiales_Lachnospiraceae_97_Enterocloster_76
OTU_368	7.07E-01	9.69E-01	1.14E-02	4.20	1.83	Eubacterium_eligens_95	Bacteria_Firmicutes_Clostridia_Clostridiales_Lachnospiraceae_Lachnospira_36
OTU_1161	7.07E-01	6.04E-01	1.30E-02	5.43	1.83	Pseudescherichia_vulneris_89	Bacteria_Proteobacteria_75_Gammaproteobacteria_75_Enterobacterales_75_Enterobacteriaceae_75_Pseudescherichia_75
OTU_436	7.10E-01	9.61E-01	1.87E-02	4.80	0.31	Bifidobacterium_stercoris_91	Bacteria_Actinobacteria_95_Actinobacteria_95_Bifidobacteriales_95_Bifidobacteriaceae_95_Pseudoscardovia_43
OTU_13490	7.07E-01	9.62E-01	1.17E-02	6.62	0.81	Bacteroides_stercoris_96	Bacteria_Bacteroidetes_Bacteroidia_Bacteroidales_Bacteroidaceae_Bacteroides_90
OTU_313	9.04E-01	6.04E-01	8.94E-01	3.16	2.95	Acidaminococcus_intestini_93	Bacteria_Firmicutes_Negativicutes_Acidaminococcales_98_Acidaminococcaceae_98_Acidaminococcus_98
OTU_3143	8.23E-01	8.16E-01	9.01E-02	4.23	2.70	Dorea_longicatena_97	Bacteria_Firmicutes_Clostridia_Clostridiales_Lachnospiraceae_Dorea_39
OTU_4285	7.07E-01	7.97E-01	4.03E-03	9.26	0.77	Bacteroides_stercoris_97	Bacteria_Bacteroidetes_Bacteroidia_Bacteroidales_Bacteroidaceae_Bacteroides_99
OTU_591	8.49E-01	6.50E-01	1.34E-02	8.47	1.39	Clostridium_perfringens_98	Bacteria_Firmicutes_94_Clostridia_94_Clostridiales_94_Clostridiaceae 1_93_Anaerobacter_36

OTU_1495	7.07E-01	6.63E-01	7.53E-02	6.64	0.66	Bacteroides_stercoris_96	Bacteria_Bacteroidetes_Bacteroidia_Bacteroidales_Bacteroidaceae_Bacteroides_58
OTU_8660	9.38E-01	9.32E-01	3.93E-02	3.18	5.49	Pseudescherichia_vulneris_95	Bacteria_Proteobacteria_Gammaproteobacteria_Enterobacterales_Enterobacteriaceae_Pseudescherichia_49
OTU_9728	7.07E-01	6.05E-01	3.43E-06	7.57	1.41	Bacteroides_xylanisolvens_97	Bacteria_Bacteroidetes_Bacteroidia_Bacteroidales_Bacteroidaceae_Bacteroides_98
OTU_1470	1.00E+00	8.08E-01	1.16E-02	2.02	4.67	Vagococcus_humatus_90	Bacteria_Firmicutes_Bacilli_76_Lactobacillales_76_Enterococcaceae_43_Melissococcus_34
OTU_79218	9.87E-01	9.61E-01	4.65E-01	5.14	4.35	Roseburia_inulinivorans_87	Bacteria_Proteobacteria_89_Gammaproteobacteria_89_Enterobacterales_89_Enterobacteriaceae_87_Pseudescherichia_67
OTU_2919	8.57E-01	6.76E-01	9.47E-01	4.11	4.04	Escherichia_albertii_89	Bacteria_Firmicutes_64_Clostridia_64_Clostridiales_64_Lachnospiraceae_63_Extibacter_8
OTU_1721	7.07E-01	9.65E-01	5.84E-05	7.08	1.62	Coprococcus_comes_97	Bacteria_Firmicutes_Clostridia_Clostridiales_Lachnospiraceae_Coprococcus_42
OTU_706	9.87E-01	9.34E-01	1.73E-02	7.41	1.67	Merdimonas_faecis_92	Bacteria_Firmicutes_Clostridia_Clostridiales_Lachnospiraceae_Clostridium XlVa_49
OTU_1011	8.29E-01	6.04E-01	5.82E-01	3.70	2.78	Parabacteroides_chongii_100	Bacteria_Bacteroidetes_Bacteroidia_Bacteroidales_Porphyromonadaceae_Parabacteroides_100
OTU_728	7.07E-01	9.27E-01	1.62E-02	8.62	0.82	Bacteroides_stercoris_98	Bacteria_Bacteroidetes_77_Bacteroidia_77_Bacteroidales_77_Bacteroidaceae_77_Bacteroides_57
OTU_878	8.25E-01	6.06E-01	7.98E-01	3.50	3.27	Hungatella_effluvii_96	Bacteria_Firmicutes_Clostridia_Clostridiales_Lachnospiraceae_Clostridium XlVa_39
OTU_493	8.25E-01	7.06E-01	1.40E-02	1.38	7.65	Phocaeicola_plebeius_99	Bacteria_Bacteroidetes_79_Bacteroidia_79_Bacteroidales_79_Bacteroidaceae_79_Phocaeicola_62
OTU_1503	7.46E-01	6.04E-01	5.74E-08	6.65	0.87	Parabacteroides_distasonis_96	Bacteria_Bacteroidetes_Bacteroidia_Bacteroidales_Porphyromonadaceae_96_Parabacteroides_96
OTU_1384	9.73E-01	9.90E-01	1.17E-03	2.03	4.71	Bacteroides_fragilis_99	Bacteria_Bacteroidetes_84_Bacteroidia_84_Bacteroidales_84_Bacteroidaceae_84_Bacteroides_63
OTU_4204	7.57E-01	8.23E-01	1.35E-02	7.11	0.96	Bacteroides_stercoris_96	Bacteria_Bacteroidetes_Bacteroidia_Bacteroidales_Bacteroidaceae_Bacteroides_96
OTU_4716	8.79E-01	7.99E-01	9.16E-02	5.13	3.87	Roseburia_inulinivorans_96	Bacteria_Firmicutes_Clostridia_Clostridiales_Lachnospiraceae_Roseburia_53
OTU_568	8.08E-01	9.82E-01	1.94E-02	7.67	0.45	Parabacteroides_merdae_95	Bacteria_Bacteroidetes_Bacteroidia_Bacteroidales_Porphyromonadaceae_Parabacteroides_100
OTU_31788	7.39E-01	6.04E-01	6.40E-02	3.22	5.23	Bacteroides_thetaiotaomicron_99	Bacteria_Bacteroidetes_Bacteroidia_Bacteroidales_Bacteroidaceae_Bacteroides_100
OTU_374	7.07E-01	9.62E-01	1.12E-01	1.08	0.34	Bifidobacterium_catenulatum_97	Bacteria_Actinobacteria_Actinobacteria_Bifidobacteriales_Bifidobacteriaceae_Bifidobacterium_90
OTU_426	7.07E-01	9.25E-01	3.41E-02	4.98	0.53	Prevotella_copri_97	Bacteria_Bacteroidetes_Bacteroidia_Bacteroidales_Prevotellaceae_98_Prevotella_97
OTU_4953	8.82E-01	9.46E-01	3.96E-01	4.26	4.93	Enterocloster_clostridioformis_97	Bacteria_Firmicutes_Clostridia_Clostridiales_Lachnospiraceae_Enterocloster_61
OTU_1592	7.07E-01	6.04E-01	1.18E-01	5.39	3.45	Clostridium_perfringens_100	Bacteria_Firmicutes_Clostridia_Clostridiales_Clostridiaceae 1_Clostridium sensu stricto_99
OTU_3818	7.80E-01	6.04E-01	1.72E-01	3.09	5.01	Erysipelatoclostridium_ramosum_96	Bacteria_Firmicutes_Erysipelotrichia_Erysipelotrichales_Erysipelatoclostridiaceae_Erysipelatoclostridium_100
OTU_3544	7.07E-01	7.44E-01	4.87E-05	7.64	1.31	Bacteroides_uniformis_96	Bacteria_Bacteroidetes_Bacteroidia_Bacteroidales_Bacteroidaceae_Bacteroides_100
OTU_4291	1.00E+00	6.04E-01	6.48E-01	3.74	4.45	Erysipelatoclostridium_ramosum_97	Bacteria_Firmicutes_Erysipelotrichia_Erysipelotrichales_Erysipelatoclostridiaceae_Erysipelatoclostridium_100
OTU_1199	9.92E-01	9.69E-01	7.38E-02	5.67	3.13	Clostridium_symbiosum_98	Bacteria_Firmicutes_85_Clostridia_85_Clostridiales_85_Lachnospiraceae_85_Faecalimonas_21
OTU_24190	8.97E-01	8.14E-01	2.17E-01	3.34	4.06	Enterocloster_citroniae_98	Bacteria_Firmicutes_Clostridia_Clostridiales_Lachnospiraceae_Enterocloster_100
OTU_4728	9.43E-01	9.20E-01	1.59E-03	1.68	5.01	Escherichia_albertii_87	Bacteria_Proteobacteria_60_Gammaproteobacteria_60_Enterobacterales_60_Enterobacteriaceae_60_Pseudescherichia_60
OTU_1070	8.19E-01	6.04E-01	5.26E-02	2.21	4.77	Enterocloster_citroniae_96	Bacteria_Firmicutes_Clostridia_Clostridiales_Lachnospiraceae_Enterocloster_99
OTU_13176	7.07E-01	9.18E-01	6.19E-03	7.40	1.87	Bacteroides_xylanisolvens_98	Bacteria_Bacteroidetes_Bacteroidia_Bacteroidales_Bacteroidaceae_Bacteroides_100
OTU_3777	9.91E-01	6.04E-01	7.02E-01	4.06	4.39	Pseudescherichia_vulneris_92	Bacteria_Proteobacteria_65_Gammaproteobacteria_65_Enterobacterales_65_Enterobacteriaceae_65_Pseudescherichia_65
OTU_3227	1.00E+00	9.29E-01	1.43E-01	4.99	3.44	Escherichia_albertii_88	Bacteria_Proteobacteria_59_Gammaproteobacteria_59_Enterobacterales_59_Enterobacteriaceae_59_Pseudescherichia_59
OTU_34436	8.48E-01	7.99E-01	1.04E-03	1.26	5.11	Enterococcus_faecalis_98	Bacteria_Firmicutes_Bacilli_Lactobacillales_99_Enterococcaceae_98_Enterococcus_90
OTU_40765	9.31E-01	6.04E-01	7.49E-01	4.09	4.54	Clostridium_symbiosum_95	Bacteria_Firmicutes_Clostridia_Clostridiales_Lachnospiraceae_Clostridium XlVa_90
OTU_555	8.82E-01	6.04E-01	6.88E-01	2.49	2.98	Veillonella_dispar_94	Bacteria_Firmicutes_Negativicutes_Veillonellales_97_Veillonellaceae_97_Veillonella_96
OTU_859	9.38E-01	9.92E-01	8.45E-02	0.17	0.56	Phocaeicola_coprocola_96	Bacteria_Bacteroidetes_Bacteroidia_Bacteroidales_Bacteroidaceae_Phocaeicola_100
OTU_2556	9.52E-01	9.98E-01	2.13E-02	7.26	2.32	Bacteroides_acidifaciens_99	Bacteria_Bacteroidetes_Bacteroidia_Bacteroidales_Bacteroidaceae_Bacteroides_100
OTU_9627	8.06E-01	6.63E-01	1.81E-03	6.00	1.97	Hungatella_effluvii_94	Bacteria_Firmicutes_Clostridia_Clostridiales_Lachnospiraceae_98_Hungatella_93
OTU_9768	9.44E-01	8.71E-01	7.84E-01	3.49	3.73	Kineothrix_alysoides_97	Bacteria_Firmicutes_Clostridia_Clostridiales_Lachnospiraceae_Hungatella_65
OTU_1364	7.07E-01	6.04E-01	1.75E-02	5.35	1.82	Pseudescherichia_vulneris_95	Bacteria_Proteobacteria_Gammaproteobacteria_Enterobacterales_Enterobacteriaceae_Pseudescherichia_62
OTU_594	9.92E-01	6.62E-01	1.22E-03	5.25	2.00	Phocaeicola_vulgatus_100	Bacteria_Bacteroidetes_98_Bacteroidia_98_Bacteroidales_98_Bacteroidaceae_98_Phocaeicola_92
OTU_1731	7.45E-01	9.57E-01	2.06E-01	3.20	4.61	Coprococcus_comes_95	Bacteria_Firmicutes_Clostridia_Clostridiales_Lachnospiraceae_98_Coprococcus_49
OTU_856	7.07E-01	6.04E-01	6.55E-02	6.03	0.37	Bacteroides_stercoris_96	Bacteria_Bacteroidetes_Bacteroidia_Bacteroidales_Bacteroidaceae_Bacteroides_62
OTU_8148	8.85E-01	9.60E-01	5.46E-02	4.21	2.59	Faecalimonas_umbilicata_97	Bacteria_Firmicutes_Clostridia_Clostridiales_Lachnospiraceae_Coprococcus_57
OTU_6057	9.38E-01	7.80E-01	4.57E-06	6.49	1.79	Bacteroides_faecis_97	Bacteria_Bacteroidetes_Bacteroidia_Bacteroidales_Bacteroidaceae_Bacteroides_100
OTU_214	7.07E-01	6.04E-01	9.16E-02	4.62	0.27	Anaerotruncus_rubiinfantis_92	Bacteria_Firmicutes_Clostridia_Clostridiales_Ruminococcaceae_Phocea_32
OTU_1128	7.07E-01	6.52E-01	8.70E-01	3.44	3.24	Pseudescherichia_vulneris_99	Bacteria_Proteobacteria_Gammaproteobacteria_Enterobacterales_Enterobacteriaceae_Pseudescherichia_66
OTU_17717	1.00E+00	9.83E-01	2.94E-04	1.90	5.30	Gibbsiella_dentisursi_87	Bacteria_Proteobacteria_96_Gammaproteobacteria_96_Enterobacterales_95_Enterobacteriaceae_95_Pseudescherichia_95
OTU_1933	1.00E+00	8.02E-01	3.80E-01	3.12	3.79	Escherichia_coli_97	Bacteria_Proteobacteria_Gammaproteobacteria_Enterobacterales_Enterobacteriaceae_Escherichia/Shigella_85
OTU_288	7.15E-01	9.92E-01	1.75E-02	1.46	8.38	Ruminococcus_bromii_96	Bacteria_Firmicutes_Clostridia_Clostridiales_Ruminococcaceae_98_Acutalibacter_51
OTU_700	9.38E-01	6.04E-01	2.09E-02	1.45	0.42	Bacteroides_uniformis_92	Bacteria_Bacteroidetes_Bacteroidia_Bacteroidales_Bacteroidaceae_94_Phocaeicola_86
OTU_298	7.07E-01	6.81E-01	6.95E-01	0.81	0.63	Collinsella_aerofaciens_92	Bacteria_Actinobacteria_96_Coriobacteriia_96_Coriobacteriales_96_Coriobacteriaceae_96_Collinsella_96
OTU_1952	8.06E-01	6.62E-01	5.03E-01	4.94	4.07	Enterococcus_faecalis_93	Bacteria_Firmicutes_99_Bacilli_94_Lactobacillales_93_Carnobacteriaceae_56_Catellicoccus_33
OTU_3875	9.65E-01	7.95E-01	4.86E-01	4.02	3.53	Clostridium_symbiosum_98	Bacteria_Firmicutes_Clostridia_94_Clostridiales_94_Lachnospiraceae_93_Clostridium XlVa_37
OTU_6002	9.92E-01	7.31E-01	2.13E-01	4.49	3.07	Enterococcus_thailandicus_96	Bacteria_Firmicutes_Bacilli_Lactobacillales_97_Enterococcaceae_81_Enterococcus_46
OTU_804	7.94E-01	6.04E-01	2.37E-02	8.83	0.33	Clostridium_cadaveris_95	Bacteria_Firmicutes_Clostridia_Clostridiales_Clostridiaceae 1_56_Proteiniclasticum_33
OTU_11522	9.13E-01	6.04E-01	2.03E-01	4.20	3.04	Clostridium_perfringens_97	Bacteria_Firmicutes_Clostridia_Clostridiales_Clostridiaceae 1_92_Clostridium sensu stricto_63
OTU_187	9.76E-01	8.58E-01	7.86E-01	1.34	1.21	Gracilibacter_thermotolerans_88	Bacteria_Firmicutes_86_Negativicutes_29_Selenomonadales_29_Sporomusaceae_28_Dendrosporobacter_19
OTU_477	7.88E-01	9.54E-01	1.71E-02	0.02	5.00	Methylophilus_methylotrophus_97	Bacteria_Proteobacteria_Betaproteobacteria_Nitrosomonadales_Methylophilaceae_Methylophilus_72
OTU_999	7.93E-01	6.04E-01	9.57E-02	3.84	2.12	Veillonella_atypica_96	Bacteria_Firmicutes_Negativicutes_Veillonellales_98_Veillonellaceae_98_Veillonella_98
OTU_1171	9.50E-01	9.34E-01	1.39E-02	6.31	1.30	Bacteroides_nordii_86	Bacteria_Firmicutes_56_Clostridia_56_Clostridiales_56_Lachnospiraceae_56_Merdimonas_35
OTU_149	7.07E-01	9.28E-01	1.25E-01	1.11	0.40	Akkermansia_muciniphila_100	Bacteria_Verrucomicrobia_Verrucomicrobiae_Verrucomicrobiales_Akkermansiaceae_Akkermansia_100
OTU_1535	9.01E-01	6.04E-01	6.57E-02	1.79	5.86	Veillonella_tobetsuensis_96	Bacteria_Firmicutes_Negativicutes_Veillonellales_Veillonellaceae_Veillonella_100
OTU_1330	9.65E-01	6.04E-01	6.16E-01	3.65	4.07	Clostridium_scindens_96	Bacteria_Firmicutes_Clostridia_Clostridiales_Lachnospiraceae_Clostridium XlVa_93
OTU_717	8.16E-01	7.21E-01	2.71E-02	1.66	6.33	Romboutsia_timonensis_90	Bacteria_Bacteroidetes_62_Bacteroidia_62_Bacteroidales_62_Bacteroidaceae_62_Mediterranea_26
OTU_10008	8.67E-01	6.59E-01	5.06E-01	4.48	3.94	Pseudescherichia_vulneris_96	Bacteria_Proteobacteria_78_Gammaproteobacteria_78_Enterobacterales_78_Enterobacteriaceae_78_Pseudescherichia_62
OTU_345	7.59E-01	6.04E-01	3.60E-01	1.04	4.06	Bacteroides_faecis_96	Bacteria_Bacteroidetes_Bacteroidia_Bacteroidales_Bacteroidaceae_Bacteroides_79

OTU_3619	9.60E-01	9.97E-01	8.56E-01	3.65	3.85	Enterococcus_thailandicus_92	Bacteria_Firmicutes_Bacilli_86_Lactobacillales_85_Carnobacteriaceae_38_Catellicoccus_19
OTU_414	7.14E-01	7.11E-01	2.43E-02	7.83	0.30	Fusobacterium_mortiferum_89	Bacteria_Fusobacteria_96_Fusobacteriia_96_Fusobacteriales_96_Fusobacteriaceae_95_Cetobacterium_69
OTU_59405	9.45E-01	6.47E-01	9.49E-02	2.60	5.00	Clostridium_symbiosum_95	Bacteria_Firmicutes_Clostridia_Clostridiales_Lachnospiraceae_Clostridium XlVa_69
OTU_7328	9.32E-01	9.25E-01	9.96E-03	3.30	5.39	Pseudescherichia_vulneris_99	Bacteria_Proteobacteria_98_Gammaproteobacteria_98_Enterobacterales_98_Enterobacteriaceae_98_Pseudescherichia_93
OTU_7520	9.38E-01	8.97E-01	1.85E-01	3.38	4.48	Pseudescherichia_vulneris_95	Bacteria_Proteobacteria_Gammaproteobacteria_Enterobacterales_Enterobacteriaceae_Pseudescherichia_68
OTU_218	9.37E-01	6.10E-01	4.59E-02	7.74	0.23	Phocaeicola_barnesiae_88	Bacteria_Bacteroidetes_Bacteroidia_Bacteroidales_Prevotellaceae_86_Paraprevotella_86
OTU_658	8.67E-01	9.32E-01	7.87E-03	1.50	5.39	Phascolarctobacterium_faecium_96	Bacteria_Firmicutes_Negativicutes_Acidaminococcales_Acidaminococcaceae_Phascolarctobacterium_100
OTU_103815	7.07E-01	6.04E-01	3.10E-01	3.93	0.14	Veillonella_rogosae_98	Bacteria_Firmicutes_Negativicutes_Veillonellales_Veillonellaceae_Veillonella_100
OTU_976	8.19E-01	6.05E-01	2.07E-02	7.00	0.71	Petrocella_atlantisensis_88	Bacteria_Firmicutes_99_Clostridia_99_Clostridiales_99_Lachnospiraceae_39_Robinsoniella_29
OTU_2260	9.31E-01	8.14E-01	2.71E-01	3.64	2.64	Sellimonas_intestinalis_96	Bacteria_Firmicutes_Clostridia_Clostridiales_Lachnospiraceae_Sellimonas_74
OTU_1662	8.79E-01	9.25E-01	6.41E-02	4.24	2.69	Coprococcus_comes_97	Bacteria_Firmicutes_Clostridia_Clostridiales_Lachnospiraceae_Coprococcus_43
OTU_377	8.82E-01	9.04E-01	6.14E-02	2.35	0.75	Actinobacillus_delphinicola_95	Bacteria_Proteobacteria_Gammaproteobacteria_Pasteurellales_92_Pasteurellaceae_92_Conservatibacter_27
OTU_1431	9.43E-01	6.04E-01	4.44E-03	7.26	1.05	Clostridium_perfringens_94	Bacteria_Firmicutes_Clostridia_Clostridiales_Clostridiaceae 1_42_Sarcina_21
OTU_160	7.85E-01	6.66E-01	8.45E-02	6.04	0.11	Eubacterium_eligens_96	Bacteria_Firmicutes_Clostridia_Clostridiales_Lachnospiraceae_Lachnospira_47
OTU_10716	7.22E-01	8.36E-01	6.96E-01	2.98	3.20	Pseudescherichia_vulneris_96	Bacteria_Proteobacteria_Gammaproteobacteria_Enterobacterales_Enterobacteriaceae_Escherichia/Shigella_55
OTU_230	9.08E-01	6.04E-01	4.10E-02	5.43	0.12	Muribaculum_intestinale_88	Bacteria_Bacteroidetes_Bacteroidia_Bacteroidales_Muribaculaceae_97_Duncaniella_40
OTU_203	9.66E-01	6.47E-01	4.96E-02	0.54	0.14	Megasphaera_elsdenii_92	Bacteria_Firmicutes_Negativicutes_93_Veillonellales_93_Veillonellaceae_93_Megasphaera_93
OTU_1040	7.07E-01	6.04E-01	2.23E-01	4.26	0.44	Streptococcus_salivarius_89	Bacteria_Firmicutes_Bacilli_88_Lactobacillales_73_Enterococcaceae_21_Bavariicoccus_10
OTU_1210	7.75E-01	8.70E-01	3.73E-01	1.78	2.49	Enterocloster_clostridioformis_94	Bacteria_Firmicutes_Clostridia_86_Clostridiales_86_Lachnospiraceae_84_Fusicatenibacter_19
OTU_13837	9.92E-01	9.70E-01	3.74E-02	4.79	3.07	Ruminococcus_faecis_96	Bacteria_Firmicutes_Clostridia_Clostridiales_Lachnospiraceae_Coprococcus_79
OTU_238	NA	7.99E-01	7.27E-02	0.00	5.42	Azospirillum_soli_98	Bacteria_Proteobacteria_Alphaproteobacteria_Rhodospirillales_Azospirillaceae_Azospirillum_99
OTU_98414	9.99E-01	7.97E-01	7.49E-03	1.61	4.21	Pseudescherichia_vulneris_89	Bacteria_Proteobacteria_50_Gammaproteobacteria_50_Enterobacterales_50_Enterobacteriaceae_47_Pseudescherichia_47
OTU_1145	9.78E-01	8.79E-01	8.84E-01	3.49	3.65	Pseudescherichia_vulneris_91	Bacteria_Proteobacteria_60_Gammaproteobacteria_60_Enterobacterales_60_Enterobacteriaceae_60_Pseudescherichia_59
OTU_367	9.92E-01	6.04E-01	6.92E-02	10.18	1.07	Allobaculum_stercoricanis_94	Bacteria_Firmicutes_Erysipelotrichia_Erysipelotrichales_Erysipelotrichaceae_Allobaculum_91
OTU_452	7.07E-01	9.32E-01	1.98E-01	7.02	0.28	Bifidobacterium_rousetti_90	Bacteria_Actinobacteria_81_Actinobacteria_81_Bifidobacteriales_81_Bifidobacteriaceae_81_Pseudoscardovia_44
OTU_1190	7.07E-01	6.04E-01	2.21E-01	3.70	2.58	Enterocloster_clostridioformis_94	Bacteria_Firmicutes_Clostridia_Clostridiales_Lachnospiraceae_Enterocloster_94
OTU_2021	9.06E-01	6.04E-01	5.22E-02	3.96	2.19	Clostridium_carnis_90	Bacteria_Firmicutes_97_Bacilli_82_Lactobacillales_65_Carnobacteriaceae_61_Isobaculum_56
OTU_3095	9.08E-01	6.06E-01	1.07E-01	2.70	3.82	Paraclostridium_benzoelyticum_97	Bacteria_Firmicutes_Clostridia_Clostridiales_Peptostreptococcaceae_Paraclostridium_89
OTU_4681	9.64E-01	7.97E-01	6.95E-01	3.19	3.52	Morganella_morganii_86	Bacteria_Proteobacteria_87_Gammaproteobacteria_87_Enterobacterales_86_Enterobacteriaceae_85_Pseudescherichia_84
OTU_515	7.07E-01	7.80E-01	3.93E-02	1.15	2.54	Anaerofilum_pentosovorans_90	Bacteria_Firmicutes_Clostridia_Clostridiales_Lachnospiraceae_49_Catonella_40
OTU_66750	7.57E-01	6.04E-01	2.64E-02	5.61	1.25	Bacteroides_kribbi_97	Bacteria_Bacteroidetes_Bacteroidia_Bacteroidales_Bacteroidaceae_Bacteroides_100
OTU_10340	8.85E-01	7.75E-01	1.32E-02	2.40	5.30	Pseudescherichia_vulneris_96	Bacteria_Proteobacteria_93_Gammaproteobacteria_93_Enterobacterales_92_Enterobacteriaceae_91_Pseudescherichia_90
OTU_1485	7.07E-01	6.04E-01	4.81E-07	6.66	1.78	Bacteroides_uniformis_95	Bacteria_Bacteroidetes_Bacteroidia_Bacteroidales_Bacteroidaceae_Bacteroides_76
OTU_666	7.14E-01	6.04E-01	3.77E-02	4.56	2.53	Peptoniphilus_grossensis_95	Bacteria_Firmicutes_Clostridia_Clostridiales_Peptoniphilaceae_Peptoniphilus_100
OTU_293	9.98E-01	6.55E-01	1.69E-01	0.55	0.20	Catenibacterium_mitsuokai_91	Bacteria_Firmicutes_Erysipelotrichia_Erysipelotrichales_Erysipelotrichaceae_Catenibacterium_69
OTU_659	7.75E-01	8.36E-01	8.76E-02	1.77	0.86	Bifidobacterium_saguini_94	Bacteria_Actinobacteria_93_Actinobacteria_93_Bifidobacteriales_93_Bifidobacteriaceae_93_Bifidobacterium_56
OTU_210	8.06E-01	6.04E-01	4.02E-02	4.91	0.45	Faecalicatena_fissicatena_98	Bacteria_Firmicutes_Clostridia_Clostridiales_Lachnospiraceae_Faecalicatena_65
OTU_8462	8.79E-01	9.00E-01	9.21E-02	3.01	4.40	Paraclostridium_benzoelyticum_97	Bacteria_Firmicutes_Clostridia_Clostridiales_Peptostreptococcaceae_Paraclostridium_100
OTU_33827	7.07E-01	6.04E-01	5.10E-03	1.82	4.26	Enterococcus_faecalis_98	Bacteria_Firmicutes_Bacilli_Lactobacillales_Enterococcaceae_Enterococcus_93
OTU_84744	7.07E-01	7.81E-01	5.91E-02	7.14	0.90	Bacteroides_stercoris_96	Bacteria_Bacteroidetes_Bacteroidia_Bacteroidales_Bacteroidaceae_Bacteroides_100
OTU_439	7.07E-01	7.73E-01	5.03E-02	6.13	1.79	Escherichia_coli_88	Bacteria_Proteobacteria_70_Gammaproteobacteria_70_Enterobacterales_70_Enterobacteriaceae_70_Pseudescherichia_70
OTU_2124	9.87E-01	7.11E-01	1.04E-02	1.95	4.06	Shigella_dysenteriae_91	Bacteria_Proteobacteria_97_Gammaproteobacteria_97_Enterobacterales_97_Enterobacteriaceae_95_Pseudescherichia_93
OTU_497	7.81E-01	9.90E-01	6.50E-02	7.03	0.15	Blautia_faecicola_98	Bacteria_Firmicutes_Clostridia_Clostridiales_Lachnospiraceae_Mediterraneibacter_65
OTU_3647	8.25E-01	9.53E-01	7.68E-01	3.31	3.06	Falcatimonas_natans_93	Bacteria_Firmicutes_Clostridia_86_Clostridiales_86_Lachnospiraceae_85_Falcatimonas_25
OTU_2749	7.07E-01	9.17E-01	1.97E-02	1.26	0.43	Bacteroides_caecimuris_96	Bacteria_Bacteroidetes_Bacteroidia_Bacteroidales_Bacteroidaceae_Bacteroides_100
OTU_347	9.38E-01	6.61E-01	9.61E-01	1.91	1.87	Agathobaculum_butyriciproducens_100	Bacteria_Firmicutes_Clostridia_Clostridiales_Ruminococcaceae_Agathobaculum_75
OTU_254	9.38E-01	6.04E-01	4.52E-02	8.04	0.34	Faecalibaculum_rodentium_89	Bacteria_Firmicutes_Erysipelotrichia_Erysipelotrichales_Erysipelotrichaceae_Allobaculum_80
OTU_534	7.46E-01	6.05E-01	3.34E-02	1.00	4.51	Pseudescherichia_vulneris_92	Bacteria_Proteobacteria_90_Gammaproteobacteria_90_Enterobacterales_90_Enterobacteriaceae_90_Pseudescherichia_89
OTU_557	8.16E-01	6.04E-01	9.33E-02	4.82	1.51	Escherichia_coli_89	Bacteria_Proteobacteria_61_Gammaproteobacteria_61_Enterobacterales_61_Enterobacteriaceae_59_Pseudescherichia_59
OTU_420	8.60E-01	6.04E-01	3.60E-01	1.88	4.59	Erysipelatoclostridium_ramosum_97	Bacteria_Firmicutes_Erysipelotrichia_Erysipelotrichales_Erysipelatoclostridiaceae_Erysipelatoclostridium_100
OTU_538	7.07E-01	8.59E-01	2.47E-02	1.77	0.36	Bifidobacterium_stercoris_97	Bacteria_Actinobacteria_Actinobacteria_Bifidobacteriales_Bifidobacteriaceae_Bifidobacterium_96
OTU_2017	7.07E-01	7.68E-01	9.96E-06	7.59	0.59	Bacteroides_uniformis_96	Bacteria_Bacteroidetes_Bacteroidia_Bacteroidales_Bacteroidaceae_Bacteroides_98
OTU_265	7.59E-01	6.04E-01	6.15E-01	2.59	4.94	Bacteroides_fragilis_96	Bacteria_Bacteroidetes_66_Bacteroidia_66_Bacteroidales_66_Bacteroidaceae_66_Mediterranea_34
OTU_287	9.04E-01	8.83E-01	1.33E-01	6.51	0.17	Blautia_caecimuris_97	Bacteria_Firmicutes_Clostridia_Clostridiales_Lachnospiraceae_Blautia_46
OTU_13344	7.07E-01	6.04E-01	5.99E-02	4.26	2.17	Bacteroides_stercoris_97	Bacteria_Bacteroidetes_Bacteroidia_Bacteroidales_Bacteroidaceae_Bacteroides_100
OTU_1033	8.89E-01	6.04E-01	4.89E-01	3.70	3.07	Bacteroides_thetaiotaomicron_100	Bacteria_Bacteroidetes_98_Bacteroidia_98_Bacteroidales_98_Bacteroidaceae_97_Bacteroides_67
OTU_561	7.07E-01	8.53E-01	3.22E-02	0.98	4.63	Clostridium_tepidum_97	Bacteria_Proteobacteria_65_Gammaproteobacteria_65_Enterobacterales_65_Enterobacteriaceae_65_Pseudescherichia_62
OTU_1146	7.07E-01	6.27E-01	2.91E-02	1.17	4.47	Eubacterium_limosum_92	Bacteria_Firmicutes_Clostridia_Clostridiales_Eubacteriaceae_77_Eubacterium_67
OTU_504	7.12E-01	6.04E-01	1.96E-01	4.91	2.02	Fusobacterium_perfoetens_86	Bacteria_Proteobacteria_52_Gammaproteobacteria_52_Enterobacterales_52_Enterobacteriaceae_51_Pseudescherichia_48
OTU_7854	8.25E-01	8.83E-01	1.10E-01	2.03	1.26	Bacteroides_caccae_98	Bacteria_Bacteroidetes_Bacteroidia_Bacteroidales_Bacteroidaceae_Bacteroides_100
OTU_1469	7.85E-01	9.73E-01	2.20E-02	2.13	5.41	Veillonella_dispar_97	Bacteria_Firmicutes_43_Negativicutes_40_Selenomonadales_28_Sporomusaceae_27_Anaerosinus_22
OTU_4485	9.75E-01	6.51E-01	3.30E-01	2.75	4.16	Kineothrix_alysoides_96	Bacteria_Firmicutes_Clostridia_Clostridiales_Lachnospiraceae_Coprococcus_75
OTU_1056	9.99E-01	6.04E-01	1.84E-01	0.48	3.35	Phocaeicola_coprocola_96	Bacteria_Bacteroidetes_Bacteroidia_Bacteroidales_Bacteroidaceae_Bacteroides_75
OTU_1659	9.67E-01	6.04E-01	5.66E-05	3.62	0.64	Phocaeicola_vulgatus_97	Bacteria_Bacteroidetes_Bacteroidia_Bacteroidales_Bacteroidaceae_Phocaeicola_100
OTU_412	7.07E-01	9.49E-01	3.18E-02	1.51	4.08	Enterococcus_dispar_99	Bacteria_Firmicutes_Bacilli_95_Lactobacillales_91_Carnobacteriaceae_60_Isobaculum_56
OTU_429	9.43E-01	9.75E-01	8.51E-01	0.88	1.02	Catenibacterium_mitsuokai_94	Bacteria_Firmicutes_Erysipelotrichia_Erysipelotrichales_Erysipelotrichaceae_Catenibacterium_100
OTU_540	9.87E-01	6.76E-01	2.40E-01	1.33	0.79	Lachnospira_pectinoschiza_98	Bacteria_Firmicutes_Clostridia_Clostridiales_Lachnospiraceae_Lachnospira_95

OTU_6539	7.07E-01	8.28E-01	2.15E-01	3.29	2.54	Pseudescherichia_vulneris_97	Bacteria_Proteobacteria_Gammaproteobacteria_Enterobacterales_Enterobacteriaceae_99_Escherichia/Shigella_57
OTU_2133	7.94E-01	6.50E-01	3.50E-04	1.56	4.64	Escherichia_albertii_89	Bacteria_Proteobacteria_72_Gammaproteobacteria_72_Enterobacterales_72_Enterobacteriaceae_72_Pseudescherichia_72
OTU_774	8.85E-01	6.04E-01	7.38E-02	3.63	1.83	Peptoniphilus_grossensis_98	Bacteria_Firmicutes_Clostridia_82_Clostridiales_82_Peptoniphilaceae_80_Peptoniphilus_80
OTU_22518	9.14E-01	9.98E-01	7.84E-01	3.38	3.58	Pseudescherichia_vulneris_97	Bacteria_Proteobacteria_83_Gammaproteobacteria_83_Enterobacterales_83_Enterobacteriaceae_83_Pseudescherichia_71
OTU_560	8.57E-01	6.81E-01	1.95E-02	0.84	4.81	Clostridium_tepidum_96	Bacteria_Firmicutes_99_Clostridia_99_Clostridiales_99_Clostridiaceae 1_98_Clostridium sensu stricto_63
OTU_703	8.25E-01	6.04E-01	3.76E-01	2.46	3.92	Clostridium_symbiosum_96	Bacteria_Firmicutes_Erysipelotrichia_54_Erysipelotrichales_54_Erysipelatoclostridiaceae_54_Erysipelatoclostridium_54
OTU_818	7.07E-01	6.04E-01	9.87E-03	6.28	1.01	Bifidobacterium_stercoris_99	Bacteria_Actinobacteria_89_Actinobacteria_89_Bifidobacteriales_89_Bifidobacteriaceae_89_Pseudoscardovia_50
OTU_11814	8.97E-01	8.64E-01	2.48E-01	2.66	3.29	Shigella_dysenteriae_91	Bacteria_Proteobacteria_77_Gammaproteobacteria_77_Enterobacterales_77_Enterobacteriaceae_76_Pseudescherichia_76
OTU_319	8.06E-01	6.50E-01	5.40E-01	0.39	0.52	Enterocloster_citroniae_89	Bacteria_Firmicutes_98_Clostridia_98_Clostridiales_98_Lachnospiraceae_97_Merdimonas_7
OTU_508	7.74E-01	6.04E-01	1.18E-02	3.07	0.49	Sutterella_wadsworthensis_91	Bacteria_Proteobacteria_99_Betaproteobacteria_99_Burkholderiales_99_Sutterellaceae_99_Sutterella_96
OTU_16362	7.46E-01	6.04E-01	1.45E-02	4.21	0.89	Bacteroides_caccae_98	Bacteria_Bacteroidetes_Bacteroidia_Bacteroidales_Bacteroidaceae_Bacteroides_100
OTU_1037	7.57E-01	6.10E-01	3.38E-03	1.14	4.00	Flavonifractor_plautii_98	Bacteria_Firmicutes_Clostridia_99_Clostridiales_99_Ruminococcaceae_99_Flavonifractor_99
OTU_1771	7.07E-01	8.09E-01	1.83E-02	3.89	1.33	Bifidobacterium_saguini_93	Bacteria_Actinobacteria_91_Actinobacteria_91_Bifidobacteriales_91_Bifidobacteriaceae_91_Pseudoscardovia_38
OTU_793	NA	6.88E-01	9.49E-02	0.00	5.85	Rosenbergiella_epipactidis_92	Bacteria_Proteobacteria_Gammaproteobacteria_76_Enterobacterales_75_Enterobacteriaceae_75_Pseudescherichia_62
OTU_861	7.07E-01	9.34E-01	7.23E-01	0.22	0.28	Bifidobacterium_callitrichidarum_91	Bacteria_Actinobacteria_94_Actinobacteria_94_Bifidobacteriales_94_Bifidobacteriaceae_94_Bifidobacterium_45
OTU_9754	9.00E-01	6.48E-01	6.33E-04	5.03	1.60	Pseudescherichia_vulneris_97	Bacteria_Proteobacteria_86_Gammaproteobacteria_86_Enterobacterales_86_Enterobacteriaceae_85_Pseudescherichia_70
OTU_15028	8.06E-01	6.04E-01	8.69E-02	5.18	2.70	Coprococcus_comes_97	Bacteria_Firmicutes_Clostridia_Clostridiales_Lachnospiraceae_Bariatricus_72
OTU_261	9.78E-01	6.04E-01	5.20E-02	6.82	0.24	Rhabdanaerobium_thermarum_87	Bacteria_Firmicutes_88_Clostridia_70_Clostridiales_69_Catabacteriaceae_12_Catabacter_12
OTU_4831	9.31E-01	7.26E-01	8.05E-02	2.25	3.34	Stomatobaculum_longum_88	Bacteria_Proteobacteria_74_Gammaproteobacteria_74_Enterobacterales_74_Enterobacteriaceae_74_Pseudescherichia_74
OTU_1187	7.07E-01	6.04E-01	5.39E-01	1.65	3.70	Flintibacter_butyricus_97	Bacteria_Firmicutes_Clostridia_Clostridiales_Ruminococcaceae_Flintibacter_70
OTU_2973	9.38E-01	9.25E-01	2.50E-01	2.85	4.07	Hungatella_hathewayi_96	Bacteria_Firmicutes_Clostridia_Clostridiales_Lachnospiraceae_99_Hungatella_94
OTU_655	7.85E-01	8.64E-01	4.51E-03	0.56	6.25	Bacteroides_fragilis_100	Bacteria_Bacteroidetes_61_Bacteroidia_61_Bacteroidales_61_Bacteroidaceae_61_Mediterranea_30
OTU_1481	7.75E-01	7.20E-01	2.64E-02	5.10	1.00	Enterococcus_pallens_96	Bacteria_Firmicutes_Bacilli_Lactobacillales_Enterococcaceae_90_Melissococcus_55
OTU_4692	9.93E-01	8.85E-01	1.63E-02	3.84	2.37	Enterocloster_clostridioformis_97	Bacteria_Firmicutes_Clostridia_Clostridiales_Lachnospiraceae_Enterocloster_93
OTU_470	7.57E-01	9.21E-01	1.46E-02	0.50	5.68	Bacteroides_fragilis_100	Bacteria_Bacteroidetes_99_Bacteroidia_99_Bacteroidales_99_Bacteroidaceae_93_Bacteroides_54
OTU_813	7.84E-01	6.04E-01	2.74E-02	4.94	1.40	Sutterella_wadsworthensis_97	Bacteria_Proteobacteria_Betaproteobacteria_Burkholderiales_Sutterellaceae_Sutterella_100
OTU_952	9.65E-01	9.41E-01	6.25E-02	4.41	2.53	Veillonella_dispar_99	Bacteria_Firmicutes_59_Negativicutes_59_Selenomonadales_22_Sporomusaceae_22_Anaerosinus_16
OTU_3743	8.97E-01	7.46E-01	4.28E-01	2.82	3.35	Enterococcus_faecalis_94	Bacteria_Firmicutes_99_Bacilli_99_Lactobacillales_98_Carnobacteriaceae_56_Isobaculum_33
OTU_10607	9.52E-01	6.04E-01	1.72E-02	1.84	3.18	Enterococcus_faecalis_97	Bacteria_Firmicutes_Bacilli_Lactobacillales_Enterococcaceae_91_Enterococcus_81
OTU_866	9.14E-01	6.04E-01	8.81E-01	3.21	3.04	Pseudescherichia_vulneris_99	Bacteria_Proteobacteria_82_Gammaproteobacteria_82_Enterobacterales_82_Enterobacteriaceae_81_Pseudescherichia_68
OTU_1703	7.07E-01	7.61E-01	3.73E-01	3.24	2.52	Dorea_longicatena_97	Bacteria_Firmicutes_Clostridia_Clostridiales_Lachnospiraceae_Dorea_100
OTU_1813	7.07E-01	6.04E-01	1.66E-01	2.63	1.66	Collinsella_aerofaciens_96	Bacteria_Actinobacteria_Coriobacteriia_Coriobacteriales_99_Coriobacteriaceae_99_Collinsella_98
OTU_308	7.91E-01	8.14E-01	4.91E-01	0.44	0.28	Catenibacterium_mitsuokai_97	Bacteria_Firmicutes_99_Erysipelotrichia_99_Erysipelotrichales_99_Erysipelotrichaceae_99_Catenibacterium_71
OTU_820	7.46E-01	9.61E-01	4.72E-04	5.11	0.66	Streptococcus_mitis_96	Bacteria_Firmicutes_Bacilli_Lactobacillales_Streptococcaceae_Streptococcus_99
OTU_450	7.57E-01	7.70E-01	1.92E-02	4.20	0.25	Prevotella_copri_96	Bacteria_Bacteroidetes_Bacteroidia_Bacteroidales_Prevotellaceae_88_Prevotella_88
OTU_67345	9.01E-01	9.34E-01	6.26E-01	2.16	2.45	Enterococcus_faecalis_98	Bacteria_Firmicutes_Bacilli_Lactobacillales_Enterococcaceae_Enterococcus_82
OTU_3679	8.74E-01	6.49E-01	1.66E-02	3.49	1.74	Veillonella_dispar_98	Bacteria_Firmicutes_Negativicutes_55_Veillonellales_50_Veillonellaceae_50_Veillonella_49
OTU_1505	7.46E-01	9.75E-01	6.04E-03	1.57	3.98	Gibbsiella_greigii_92	Bacteria_Proteobacteria_Gammaproteobacteria_Enterobacterales_Enterobacteriaceae_Pseudescherichia_41
OTU_283	8.57E-01	9.38E-01	6.83E-01	1.00	0.80	Eubacterium_eligens_100	Bacteria_Firmicutes_Clostridia_Clostridiales_Lachnospiraceae_Lachnospira_100
OTU_483	7.07E-01	6.04E-01	2.72E-01	5.05	2.08	Enterococcus_asini_87	Bacteria_Fusobacteria_77_Fusobacteriia_77_Fusobacteriales_77_Fusobacteriaceae_75_Cetobacterium_45
OTU_761	9.43E-01	9.70E-01	1.60E-02	1.19	4.63	Bacteroides_fragilis_89	Bacteria_Proteobacteria_80_Gammaproteobacteria_80_Enterobacterales_80_Enterobacteriaceae_80_Pseudescherichia_80
OTU_449	7.07E-01	6.04E-01	3.96E-01	1.43	0.73	Collinsella_aerofaciens_99	Bacteria_Actinobacteria_79_Coriobacteriia_79_Coriobacteriales_78_Coriobacteriaceae_74_Collinsella_73
OTU_686	7.87E-01	6.04E-01	8.02E-02	4.94	1.09	Intestinimonas_butyriciproducens_95	Bacteria_Firmicutes_Clostridia_Clostridiales_Ruminococcaceae_Intestinimonas_86
OTU_1446	8.83E-01	6.04E-01	1.82E-03	4.31	1.05	Clostridium_perfringens_92	Bacteria_Firmicutes_Clostridia_96_Clostridiales_96_Clostridiaceae 1_96_Sarcina_47
OTU_4533	8.16E-01	9.94E-01	3.15E-03	1.93	3.40	Flavonifractor_plautii_97	Bacteria_Firmicutes_Clostridia_Clostridiales_Ruminococcaceae_Flavonifractor_100
OTU_908	7.07E-01	6.04E-01	9.17E-01	1.61	1.51	Senegalimassilia_anaerobia_96	Bacteria_Actinobacteria_Coriobacteriia_Coriobacteriales_97_Coriobacteriaceae_97_Senegalimassilia_94
OTU_2037	7.46E-01	8.28E-01	2.10E-01	3.37	2.07	Bacteroides_fragilis_96	Bacteria_Bacteroidetes_Bacteroidia_Bacteroidales_Bacteroidaceae_Bacteroides_100
OTU_23658	7.86E-01	7.70E-01	2.49E-01	2.93	1.87	Veillonella_parvula_98	Bacteria_Firmicutes_Negativicutes_Veillonellales_Veillonellaceae_Veillonella_100
OTU_380	7.07E-01	6.04E-01	1.76E-01	5.11	0.45	Acetivibrio_thermocellus_89	Bacteria_Firmicutes_Clostridia_98_Clostridiales_98_Ruminococcaceae_91_Monoglobus_28
OTU_42361	9.99E-01	6.04E-01	6.27E-04	6.60	0.37	Bacteroides_xylanisolvens_97	Bacteria_Bacteroidetes_Bacteroidia_Bacteroidales_Bacteroidaceae_Bacteroides_100
OTU_627	7.07E-01	9.37E-01	1.72E-03	5.10	0.34	Enterococcus_diestrammenae_89	Bacteria_Firmicutes_Clostridia_68_Clostridiales_68_Clostridiaceae 1_68_Desnuesiella_29
OTU_799	7.07E-01	9.12E-01	1.73E-02	1.60	3.29	Shigella_dysenteriae_93	Bacteria_Proteobacteria_Deltaproteobacteria_86_Desulfovibrionales_86_Desulfovibrionaceae_86_Bilophila_86
OTU_937	8.54E-01	7.34E-01	2.07E-02	4.76	1.51	Erwinia_iniecta_94	Bacteria_Proteobacteria_Gammaproteobacteria_99_Enterobacterales_99_Enterobacteriaceae_98_Pseudescherichia_49
OTU_1435	8.60E-01	9.62E-01	1.68E-02	5.54	1.22	Sutterella_massiliensis_97	Bacteria_Proteobacteria_Betaproteobacteria_Burkholderiales_Sutterellaceae_Sutterella_100
OTU_548	8.74E-01	6.04E-01	3.33E-02	7.19	0.51	Muribaculum_intestinale_88	Bacteria_Bacteroidetes_Bacteroidia_97_Bacteroidales_97_Muribaculaceae_84_Muribaculum_51
OTU_137	9.53E-01	9.84E-01	1.37E-01	0.83	0.27	Clostridium_spiroforme_93	Bacteria_Firmicutes_Erysipelotrichia_Erysipelotrichales_Erysipelotrichaceae_Faecalibacillus_100
OTU_747	7.57E-01	6.04E-01	2.18E-02	5.50	1.04	Clostridium_quinii_90	Bacteria_Proteobacteria_92_Gammaproteobacteria_92_Enterobacterales_92_Enterobacteriaceae_91_Pseudescherichia_78
OTU_951	7.11E-01	6.39E-01	1.07E-01	5.30	1.43	Bacteroides_intestinalis_97	Bacteria_Bacteroidetes_Bacteroidia_Bacteroidales_Bacteroidaceae_Bacteroides_100
OTU_10457	9.62E-01	9.04E-01	9.49E-01	3.11	3.05	Falcatimonas_natans_97	Bacteria_Firmicutes_Clostridia_Clostridiales_Lachnospiraceae_Clostridium XlVa_25
OTU_1905	7.07E-01	7.65E-01	2.84E-05	5.14	1.41	Clostridium_perfringens_96	Bacteria_Firmicutes_Clostridia_Clostridiales_Lachnospiraceae_59_Fusicatenibacter_10
OTU_29125	7.81E-01	6.50E-01	5.03E-01	3.85	3.23	Bacteroides_thetaiotaomicron_98	Bacteria_Bacteroidetes_Bacteroidia_Bacteroidales_Bacteroidaceae_Bacteroides_100
OTU_68715	7.07E-01	6.56E-01	1.19E-01	0.77	0.19	Bifidobacterium_stercoris_98	Bacteria_Actinobacteria_Actinobacteria_Bifidobacteriales_Bifidobacteriaceae_Bifidobacterium_97
OTU_1138	7.93E-01	9.57E-01	6.46E-02	4.56	1.80	Blautia_coccoides_94	Bacteria_Firmicutes_Clostridia_Clostridiales_Lachnospiraceae_94_Blautia_75
OTU_1151	8.25E-01	6.56E-01	2.57E-01	2.41	4.26	Roseburia_inulinivorans_94	Bacteria_Firmicutes_Clostridia_Clostridiales_Lachnospiraceae_73_Agathobacter_33
OTU_3565	8.94E-01	6.76E-01	2.02E-03	5.52	1.07	Clostridium_uliginosum_90	Bacteria_Firmicutes_Negativicutes_49_Selenomonadales_34_Sporomusaceae_34_Anaerosinus_34
OTU_844	7.12E-01	6.04E-01	7.59E-01	1.62	1.41	Slackia_isoflavoniconvertens_94	Bacteria_Actinobacteria_96_Coriobacteriia_96_Eggerthellales_96_Eggerthellaceae_96_Slackia_96
OTU_845	7.07E-01	6.08E-01	6.06E-01	1.59	1.19	Collinsella_aerofaciens_93	Bacteria_Actinobacteria_92_Coriobacteriia_92_Coriobacteriales_92_Coriobacteriaceae_91_Collinsella_91

OTU_1402	7.46E-01	8.34E-01	1.50E-01	4.04	2.62	Coprococcus_comes_97	Bacteria_Firmicutes_Clostridia_Clostridiales_Lachnospiraceae_Coprococcus_34
OTU_2038	7.57E-01	9.47E-01	7.10E-04	5.67	0.35	Parabacteroides_distasonis_93	Bacteria_Bacteroidetes_Bacteroidia_Bacteroidales_Bacteroidaceae_73_Bacteroides_65
OTU_938	9.45E-01	6.10E-01	7.54E-02	3.99	1.94	Sutterella_massiliensis_95	Bacteria_Proteobacteria_Betaproteobacteria_98_Burkholderiales_98_Sutterellaceae_98_Sutterella_98
OTU_1332	8.57E-01	4.60E-01	3.12E-01	3.45	2.57	Clostridium_perfringens_97	Bacteria_Firmicutes_Clostridia_95_Clostridiales_95_Clostridiaceae 1_95_Clostridium sensu stricto_78
OTU_3237	9.10E-01	6.04E-01	8.56E-05	5.17	1.90	Bacteroides_uniformis_97	Bacteria_Bacteroidetes_Bacteroidia_Bacteroidales_Bacteroidaceae_Bacteroides_100
OTU_410	9.59E-01	9.97E-01	9.20E-03	3.03	0.69	Pseudoflavonifractor_phocaeensis_96	Bacteria_Firmicutes_Clostridia_Clostridiales_Ruminococcaceae_Intestinimonas_100
OTU_1425	7.07E-01	6.04E-01	8.94E-01	2.82	2.44	Raoultella_planticola_91	Bacteria_Proteobacteria_99_Gammaproteobacteria_99_Enterobacterales_98_Enterobacteriaceae_98_Pseudescherichia_95
OTU_1107	7.91E-01	6.04E-01	5.05E-02	1.58	0.36	Megamonas_funiformis_99	Bacteria_Firmicutes_Negativicutes_Selenomonadales_Selenomonadaceae_Megamonas_100
OTU_4007	7.07E-01	6.04E-01	1.68E-02	4.19	2.13	Clostridium_perfringens_97	Bacteria_Firmicutes_Clostridia_Clostridiales_Clostridiaceae 1_96_Sarcina_27
OTU_4032	9.38E-01	6.04E-01	2.47E-02	2.25	0.96	Phocaeicola_vulgatus_95	Bacteria_Bacteroidetes_Bacteroidia_Bacteroidales_Bacteroidaceae_Phocaeicola_99
OTU_725	7.07E-01	6.04E-01	1.31E-01	5.72	0.71	Vagococcus_carniphilus_92	Bacteria_Firmicutes_Bacilli_Lactobacillales_Streptococcaceae_58_Streptococcus_56
OTU_1390	9.67E-01	9.53E-01	2.48E-01	1.24	2.50	Enterocloster_clostridioformis_96	Bacteria_Firmicutes_Clostridia_Clostridiales_Lachnospiraceae_Enterocloster_54
OTU_3975	9.64E-01	6.14E-01	1.48E-02	2.19	3.56	Enterocloster_citroniae_97	Bacteria_Firmicutes_Clostridia_Clostridiales_Lachnospiraceae_Enterocloster_99
OTU_5325	7.07E-01	6.14E-01	4.23E-01	1.54	1.19	Hungatella_xylanolytica_96	Bacteria_Firmicutes_Clostridia_Clostridiales_Lachnospiraceae_Kineothrix_45
OTU_7115	7.85E-01	6.04E-01	2.63E-02	3.49	2.23	Clostridium_perfringens_97	Bacteria_Firmicutes_Clostridia_Clostridiales_Clostridiaceae 1_Clostridium sensu stricto_69
OTU_850	7.07E-01	5.32E-01	1.42E-01	2.72	0.54	Streptococcus_vestibularis_98	Bacteria_Firmicutes_Clostridia_61_Clostridiales_61_Lachnospiraceae_61_Catonella_44
OTU_8912	7.46E-01	6.73E-01	1.70E-02	1.45	4.25	Faecalibacterium_prausnitzii_94	Bacteria_Firmicutes_Clostridia_Clostridiales_Ruminococcaceae_Faecalibacterium_65
OTU_1454	7.91E-01	6.81E-01	1.45E-01	1.04	0.58	Parabacteroides_chongii_94	Bacteria_Bacteroidetes_Bacteroidia_Bacteroidales_Porphyromonadaceae_98_Parabacteroides_96
OTU_676	9.38E-01	8.42E-01	1.21E-02	0.91	5.02	Pseudescherichia_vulneris_99	Bacteria_Proteobacteria_96_Gammaproteobacteria_96_Enterobacterales_96_Enterobacteriaceae_96_Pseudescherichia_88
OTU_2312	8.31E-01	8.42E-01	9.28E-02	1.92	2.89	Enterocloster_citroniae_96	Bacteria_Firmicutes_Clostridia_Clostridiales_Lachnospiraceae_Enterocloster_100
OTU_307	7.07E-01	6.04E-01	9.96E-02	5.04	1.36	Olsenella_profusa_97	Bacteria_Actinobacteria_Coriobacteriia_Coriobacteriales_Atopobiaceae_Olsenella_100
OTU_33157	7.07E-01	7.95E-01	7.30E-01	2.71	2.38	Veillonella_dispar_97	Bacteria_Firmicutes_Negativicutes_Veillonellales_Veillonellaceae_Veillonella_100
OTU_9908	7.80E-01	6.04E-01	4.02E-01	3.38	2.79	Clostridium_perfringens_96	Bacteria_Firmicutes_Clostridia_Clostridiales_Clostridiaceae 1_83_Desnuesiella_34
OTU_417	8.06E-01	7.07E-01	1.64E-01	0.56	0.16	Collinsella_aerofaciens_100	Bacteria_Actinobacteria_92_Coriobacteriia_92_Coriobacteriales_92_Coriobacteriaceae_92_Collinsella_90
OTU_12155	8.85E-01	9.12E-01	9.81E-01	1.26	1.25	Dorea_formicigenerans_96	Bacteria_Firmicutes_Clostridia_Clostridiales_Lachnospiraceae_Dorea_64
OTU_2161	9.78E-01	8.52E-01	4.57E-01	3.68	2.90	Roseburia_inulinivorans_91	Bacteria_Proteobacteria_41_Gammaproteobacteria_41_Enterobacterales_40_Enterobacteriaceae_40_Pseudescherichia_37
OTU_851	8.06E-01	6.72E-01	1.81E-01	0.77	0.32	Bacteroides_uniformis_96	Bacteria_Bacteroidetes_Bacteroidia_Bacteroidales_Bacteroidaceae_Bacteroides_82
OTU_14783	7.07E-01	9.90E-01	1.26E-02	1.34	5.01	Veillonella_dispar_95	Bacteria_Firmicutes_Negativicutes_Veillonellales_94_Veillonellaceae_94_Veillonella_93
OTU_4158	7.07E-01	6.04E-01	1.71E-02	3.85	1.70	Bacteroides_xylanisolvens_98	Bacteria_Bacteroidetes_Bacteroidia_Bacteroidales_Bacteroidaceae_Bacteroides_100
OTU_4717	7.73E-01	6.04E-01	1.11E-04	4.29	0.92	Bacteroides_caecimuris_95	Bacteria_Bacteroidetes_Bacteroidia_Bacteroidales_Bacteroidaceae_Bacteroides_93
OTU_5618	7.91E-01	9.34E-01	2.22E-01	2.51	3.43	Serratia_surfactantfaciens_94	Bacteria_Proteobacteria_Gammaproteobacteria_Enterobacterales_Enterobacteriaceae_Escherichia/Shigella_85
OTU_1178	8.25E-01	8.27E-01	2.12E-02	3.50	1.24	Clostridium_perfringens_97	Bacteria_Bacteroidetes_55_Bacteroidia_54_Bacteroidales_54_Bacteroidaceae_53_Phocaeicola_42
OTU_7139	7.46E-01	8.45E-01	3.14E-01	2.90	2.21	Blautia_hominis_98	Bacteria_Firmicutes_Clostridia_Clostridiales_Lachnospiraceae_Blautia_84
OTU_1290	8.30E-01	6.04E-01	9.00E-02	3.52	0.89	Allobaculum_stercoricanis_88	Bacteria_Firmicutes_Erysipelotrichia_Erysipelotrichales_Erysipelotrichaceae_Faecalibaculum_20
OTU_17689	8.06E-01	9.85E-01	6.05E-01	3.42	3.09	Sellimonas_intestinalis_96	Bacteria_Firmicutes_Clostridia_Clostridiales_Lachnospiraceae_Mediterraneibacter_37
OTU_3223	8.06E-01	6.63E-01	1.23E-01	2.33	3.28	Paraclostridium_benzoelyticum_96	Bacteria_Firmicutes_Clostridia_Clostridiales_Peptostreptococcaceae_Paraclostridium_96
OTU_2181	7.57E-01	9.46E-01	3.61E-04	4.62	0.73	Streptococcus_porcorum_88	Bacteria_Proteobacteria_50_Gammaproteobacteria_50_Enterobacterales_50_Enterobacteriaceae_50_Pseudescherichia_50
OTU_4794	9.93E-01	8.83E-01	6.30E-01	2.83	2.57	Enterocloster_citroniae_96	Bacteria_Firmicutes_Clostridia_Clostridiales_Lachnospiraceae_Enterocloster_60
OTU_810	7.07E-01	9.54E-01	2.71E-02	1.78	4.02	Lutispora_thermophila_88	Bacteria_Proteobacteria_92_Gammaproteobacteria_92_Enterobacterales_91_Enterobacteriaceae_91_Pseudescherichia_83
OTU_815	9.69E-01	6.63E-01	4.25E-04	5.08	0.53	Clostridium_symbiosum_93	Bacteria_Firmicutes_Clostridia_Clostridiales_Lachnospiraceae_99_Clostridium XlVa_56
OTU_1135	8.83E-01	5.09E-01	1.97E-03	4.94	1.19	Clostridium_perfringens_95	Bacteria_Firmicutes_Clostridia_Clostridiales_Clostridiaceae 1_48_Clostridium sensu stricto_28
OTU_25591	8.53E-01	6.04E-01	5.05E-02	2.08	3.43	Enterococcus_faecalis_98	Bacteria_Firmicutes_Bacilli_Lactobacillales_Enterococcaceae_99_Enterococcus_93
OTU_1009	9.04E-01	9.82E-01	3.13E-04	4.59	0.79	Pseudescherichia_vulneris_99	Bacteria_Proteobacteria_98_Gammaproteobacteria_98_Enterobacterales_97_Enterobacteriaceae_97_Pseudescherichia_79
OTU_11600	9.38E-01	9.74E-01	2.06E-02	1.84	2.98	Pseudescherichia_vulneris_89	Bacteria_Proteobacteria_95_Gammaproteobacteria_95_Enterobacterales_95_Enterobacteriaceae_95_Pseudescherichia_80
OTU_4111	9.38E-01	9.46E-01	3.74E-03	3.76	0.63	Collinsella_aerofaciens_98	Bacteria_Actinobacteria_76_Coriobacteriia_76_Coriobacteriales_76_Coriobacteriaceae_75_Collinsella_71
OTU_642	9.59E-01	6.43E-01	2.23E-02	2.94	0.58	Lacrimispora_indolis_97	Bacteria_Firmicutes_Clostridia_Clostridiales_Lachnospiraceae_Lacrimispora_48
OTU_1153	7.75E-01	6.66E-01	4.62E-01	2.58	2.09	Blautia_producta_98	Bacteria_Firmicutes_71_Clostridia_71_Clostridiales_71_Lachnospiraceae_71_Fusicatenibacter_13
OTU_2285	8.94E-01	7.33E-01	3.34E-01	3.00	2.53	Clostridium_symbiosum_95	Bacteria_Firmicutes_Clostridia_Clostridiales_Lachnospiraceae_Clostridium XlVa_61
OTU_47936	7.11E-01	7.73E-01	7.06E-02	1.80	3.70	Bacteroides_thetaiotaomicron_99	Bacteria_Bacteroidetes_Bacteroidia_Bacteroidales_Bacteroidaceae_Bacteroides_100
OTU_933	7.07E-01	6.57E-01	3.30E-01	3.14	1.96	Slackia_isoflavoniconvertens_96	Bacteria_Proteobacteria_59_Gammaproteobacteria_59_Enterobacterales_59_Enterobacteriaceae_59_Pseudescherichia_59
OTU_14549	8.68E-01	9.54E-01	1.60E-02	1.91	3.08	Pseudescherichia_vulneris_92	Bacteria_Proteobacteria_96_Gammaproteobacteria_96_Enterobacterales_96_Enterobacteriaceae_96_Pseudescherichia_96
OTU_1467	8.82E-01	9.62E-01	9.21E-02	1.43	4.83	Veillonella_tobetsuensis_89	Bacteria_Firmicutes_Clostridia_93_Clostridiales_93_Lachnospiraceae_93_Merdimonas_40
OTU_15493	7.07E-01	7.02E-01	4.66E-01	4.08	1.36	Cronobacter_sakazakii_98	Bacteria_Proteobacteria_Gammaproteobacteria_Enterobacterales_Enterobacteriaceae_Cronobacter_38
OTU_2137	7.07E-01	9.94E-01	1.86E-02	5.25	0.48	Mobilitalea_sibirica_88	Bacteria_Bacteroidetes_68_Bacteroidia_67_Bacteroidales_67_Bacteroidaceae_67_Mediterranea_31
OTU_50095	9.38E-01	9.12E-01	2.34E-03	1.59	3.36	Pseudescherichia_vulneris_97	Bacteria_Proteobacteria_Gammaproteobacteria_Enterobacterales_Enterobacteriaceae_Escherichia/Shigella_83
OTU_848	7.07E-01	6.04E-01	4.25E-01	1.91	3.07	Veillonella_dispar_98	Bacteria_Firmicutes_Negativicutes_64_Veillonellales_50_Veillonellaceae_50_Veillonella_46
OTU_1727	8.11E-01	6.04E-01	1.31E-07	5.05	1.04	Parabacteroides_distasonis_96	Bacteria_Bacteroidetes_Bacteroidia_Bacteroidales_Porphyromonadaceae_Parabacteroides_100
OTU_1765	9.61E-01	9.75E-01	9.50E-01	3.04	3.12	Clostridium_perfringens_97	Bacteria_Firmicutes_Clostridia_Clostridiales_Clostridiaceae 1_Clostridium sensu stricto_46
OTU_3363	9.45E-01	6.04E-01	1.43E-01	3.32	2.14	Clostridium_perfringens_99	Bacteria_Firmicutes_73_Clostridia_73_Clostridiales_73_Clostridiaceae 1_73_Anaerobacter_26
OTU_3590	9.60E-01	6.22E-01	1.72E-02	3.65	1.57	Pseudescherichia_vulneris_95	Bacteria_Proteobacteria_99_Gammaproteobacteria_99_Enterobacterales_99_Enterobacteriaceae_99_Escherichia/Shigella_55
OTU_821	7.07E-01	6.07E-01	1.45E-04	5.29	0.55	Hungatella_effluvii_94	Bacteria_Firmicutes_Clostridia_Clostridiales_Lachnospiraceae_Clostridium XlVa_31
OTU_2468	8.06E-01	6.62E-01	2.17E-04	3.19	0.26	Bacteroides_finegoldii_98	Bacteria_Bacteroidetes_Bacteroidia_Bacteroidales_Bacteroidaceae_Bacteroides_100
OTU_3482	7.07E-01	6.16E-01	4.96E-02	2.32	0.33	Bifidobacterium_stercoris_98	Bacteria_Actinobacteria_Actinobacteria_Bifidobacteriales_Bifidobacteriaceae_Bifidobacterium_99
OTU_680	9.87E-01	7.40E-01	9.05E-03	1.00	2.43	Desulfovibrio_intestinalis_92	Bacteria_Proteobacteria_Deltaproteobacteria_Desulfovibrionales_Desulfovibrionaceae_Bilophila_100
OTU_837	7.07E-01	9.74E-01	1.12E-02	5.87	0.70	Bacteroides_stercoris_96	Bacteria_Bacteroidetes_Bacteroidia_Bacteroidales_Bacteroidaceae_Bacteroides_95
OTU_597	7.07E-01	6.04E-01	6.18E-01	2.64	4.05	Veillonella_dispar_96	Bacteria_Firmicutes_Negativicutes_Veillonellales_Veillonellaceae_Veillonella_82
OTU_10445	8.41E-01	6.04E-01	4.12E-03	3.92	1.60	Clostridium_uliginosum_89	Bacteria_Firmicutes_Negativicutes_62_Selenomonadales_24_Sporomusaceae_21_Anaerosinus_16

OTU_2010	7.46E-01	8.65E-01	1.49E-02	4.71	0.81	Bacteroides_clarus_98	Bacteria_Bacteroidetes_Bacteroidia_Bacteroidales_Bacteroidaceae_Bacteroides_97
OTU_269	NA	6.76E-01	7.45E-02	0.00	5.80	Oharaeibacter_diazotrophicus_100	Bacteria_Proteobacteria_Alphaproteobacteria_Rhizobiales_Pleomorphomonadaceae_96_Oharaeibacter_96
OTU_30088	8.57E-01	9.41E-01	3.75E-04	4.20	0.57	Bacteroides_thetaiotaomicron_96	Bacteria_Bacteroidetes_Bacteroidia_Bacteroidales_Bacteroidaceae_Bacteroides_93
OTU_48536	7.85E-01	6.04E-01	3.21E-01	1.76	2.40	Enterococcus_faecalis_98	Bacteria_Firmicutes_Bacilli_Lactobacillales_Enterococcaceae_98_Enterococcus_84
OTU_2078	7.57E-01	7.04E-01	1.15E-02	2.95	0.88	Enterocloster_citroniae_91	Bacteria_Firmicutes_Clostridia_Clostridiales_Lachnospiraceae_Enterocloster_94
OTU_2880	9.92E-01	6.04E-01	1.72E-01	2.00	3.40	Clostridium_symbiosum_95	Bacteria_Firmicutes_Clostridia_Clostridiales_Lachnospiraceae_Enterocloster_50
OTU_4754	8.53E-01	6.72E-01	1.67E-01	3.35	2.22	Veillonella_parvula_99	Bacteria_Firmicutes_77_Negativicutes_75_Selenomonadales_32_Sporomusaceae_30_Anaerosinus_22
OTU_749	8.67E-01	6.63E-01	6.41E-02	2.92	1.85	Faecalicatena_orotica_96	Bacteria_Firmicutes_Clostridia_Clostridiales_Lachnospiraceae_92_Lachnospiracea_incertae_sedis_38
OTU_1335	9.58E-01	7.31E-01	1.43E-04	4.99	0.39	Bacteroides_thetaiotaomicron_97	Bacteria_Bacteroidetes_Bacteroidia_Bacteroidales_Bacteroidaceae_Bacteroides_100
OTU_15945	9.31E-01	9.62E-01	5.30E-01	3.28	2.89	Roseburia_inulinivorans_96	Bacteria_Firmicutes_Clostridia_Clostridiales_Lachnospiraceae_Mediterraneibacter_25
OTU_321	8.25E-01	6.04E-01	3.77E-02	3.76	0.51	Kineothrix_alysoides_97	Bacteria_Firmicutes_Clostridia_Clostridiales_Lachnospiraceae_Kineothrix_82
OTU_530	8.31E-01	9.62E-01	2.98E-01	3.17	2.39	Anaerobium_acetethylicum_96	Bacteria_Firmicutes_Clostridia_Clostridiales_Lachnospiraceae_Clostridium XlVa_26
OTU_886	7.94E-01	6.05E-01	8.80E-03	4.49	0.58	Veillonella_parvula_89	Bacteria_Firmicutes_Negativicutes_90_Veillonellales_87_Veillonellaceae_87_Veillonella_87
OTU_39776	9.32E-01	9.41E-01	3.74E-03	5.27	0.22	Parabacteroides_distasonis_98	Bacteria_Bacteroidetes_Bacteroidia_Bacteroidales_Porphyromonadaceae_Parabacteroides_100
OTU_66375	8.97E-01	9.62E-01	1.15E-02	4.55	0.90	Hungatella_effluvii_94	Bacteria_Firmicutes_79_Clostridia_79_Clostridiales_79_Lachnospiraceae_79_Merdimonas_24
OTU_3924	7.34E-01	8.65E-01	1.27E-02	1.95	3.59	Escherichia_coli_88	Bacteria_Firmicutes_85_Clostridia_85_Clostridiales_85_Lachnospiraceae_85_Eisenbergiella_82
OTU_520	NA	9.25E-01	6.25E-02	0.00	3.98	Polyangium_sorediatum_96	Bacteria_Proteobacteria_Deltaproteobacteria_Myxococcales_Sorangiineae_Polyangiaceae_Polyangium_56
OTU_727	7.07E-01	8.50E-01	4.27E-02	5.43	0.41	Bacteroides_stercoris_96	Bacteria_Bacteroidetes_Bacteroidia_Bacteroidales_Bacteroidaceae_91_Bacteroides_89
OTU_405	9.32E-01	8.79E-01	1.50E-02	0.47	4.55	Phascolarctobacterium_faecium_99	Bacteria_Firmicutes_Negativicutes_99_Acidaminococcales_99_Acidaminococcaceae_99_Phascolarctobacterium_98
OTU_707	7.07E-01	9.46E-01	5.75E-02	5.95	0.31	Bacteroides_stercoris_96	Bacteria_Bacteroidetes_Bacteroidia_Bacteroidales_Bacteroidaceae_Bacteroides_95
OTU_272	7.93E-01	6.04E-01	1.78E-01	5.53	1.78	Anaerostipes_caccae_100	Bacteria_Firmicutes_Clostridia_Clostridiales_Lachnospiraceae_Anaerostipes_100
OTU_606	9.43E-01	6.10E-01	4.68E-02	5.28	0.50	Ruminiclostridium_cellobioparum_86	Bacteria_Firmicutes_96_Clostridia_91_Clostridiales_91_Lachnospiraceae_32_Lactonifactor_12
OTU_1761	9.05E-01	7.65E-01	2.53E-01	3.44	2.40	Clostridium_perfringens_92	Bacteria_Firmicutes_Clostridia_Clostridiales_Lachnospiraceae_81_Fusicatenibacter_17
OTU_1866	8.57E-01	6.04E-01	3.14E-02	4.40	1.33	Pseudescherichia_vulneris_97	Bacteria_Proteobacteria_Gammaproteobacteria_Enterobacterales_Enterobacteriaceae_Escherichia/Shigella_70
OTU_4691	9.87E-01	9.66E-01	4.03E-03	4.39	0.13	Veillonella_atypica_91	Bacteria_Firmicutes_Negativicutes_Veillonellales_55_Veillonellaceae_55_Veillonella_53
OTU_705	7.07E-01	6.81E-01	1.97E-01	1.97	0.28	Collinsella_aerofaciens_98	Bacteria_Actinobacteria_74_Coriobacteriia_74_Coriobacteriales_74_Coriobacteriaceae_72_Collinsella_69
OTU_1751	7.07E-01	9.73E-01	1.62E-02	2.23	3.87	Bacteroides_kribbi_94	Bacteria_Bacteroidetes_98_Bacteroidia_98_Bacteroidales_98_Bacteroidaceae_98_Bacteroides_95
OTU_363	NA	7.95E-01	1.75E-02	0.00	2.96	Methylotenera_mobilis_97	Bacteria_Proteobacteria_Betaproteobacteria_Nitrosomonadales_Methylophilaceae_Methylophilus_81
OTU_2199	9.38E-01	8.73E-01	3.87E-02	1.85	2.93	Enterocloster_aldenensis_97	Bacteria_Firmicutes_Clostridia_Clostridiales_Lachnospiraceae_Enterocloster_91
OTU_263	9.92E-01	7.99E-01	4.59E-01	1.20	0.88	Oscillibacter_ruminantium_92	Bacteria_Firmicutes_Clostridia_Clostridiales_Ruminococcaceae_Dysosmobacter_21
OTU_27510	8.25E-01	6.66E-01	2.11E-02	4.00	1.59	Clostridium_perfringens_96	Bacteria_Firmicutes_Clostridia_Clostridiales_Clostridiaceae 1_69_Clostridium sensu stricto_49
OTU_2976	9.90E-01	9.03E-01	6.83E-03	1.76	4.93	Bacteroides_timonensis_98	Bacteria_Bacteroidetes_Bacteroidia_Bacteroidales_Bacteroidaceae_Bacteroides_100
OTU_768	7.11E-01	6.10E-01	5.98E-02	4.01	0.60	Collinsella_aerofaciens_100	Bacteria_Actinobacteria_82_Coriobacteriia_82_Coriobacteriales_82_Coriobacteriaceae_80_Collinsella_79
OTU_10230	9.67E-01	9.17E-01	1.98E-02	3.75	1.85	Enterococcus_thailandicus_97	Bacteria_Firmicutes_Bacilli_Lactobacillales_98_Enterococcaceae_87_Vagococcus_31
OTU_1265	7.23E-01	9.32E-01	2.94E-02	4.06	0.34	Bacteroides_uniformis_96	Bacteria_Bacteroidetes_Bacteroidia_Bacteroidales_Bacteroidaceae_85_Bacteroides_78
OTU_11828	7.45E-01	4.60E-01	1.92E-02	3.45	1.41	Peptoniphilus_grossensis_97	Bacteria_Firmicutes_Clostridia_Clostridiales_Peptoniphilaceae_Peptoniphilus_100
OTU_305	7.07E-01	6.04E-01	7.68E-01	0.29	0.36	Kluyvera_intermedia_91	Bacteria_Proteobacteria_Gammaproteobacteria_Enterobacterales_Enterobacteriaceae_97_Leclercia_59
OTU_5090	1.00E+00	6.04E-01	7.55E-03	1.93	3.20	Pseudescherichia_vulneris_90	Bacteria_Proteobacteria_78_Gammaproteobacteria_78_Enterobacterales_78_Enterobacteriaceae_78_Pseudescherichia_78
OTU_1941	7.07E-01	9.42E-01	3.40E-02	1.36	3.29	Phascolarctobacterium_faecium_96	Bacteria_Firmicutes_Negativicutes_99_Acidaminococcales_99_Acidaminococcaceae_99_Phascolarctobacterium_99
OTU_653	9.99E-01	9.69E-01	5.31E-03	5.37	0.35	Sutterella_wadsworthensis_98	Bacteria_Proteobacteria_99_Betaproteobacteria_99_Burkholderiales_99_Sutterellaceae_96_Sutterella_96
OTU_20677	9.32E-01	7.02E-01	1.24E-01	3.01	1.64	Veillonella_parvula_98	Bacteria_Firmicutes_Negativicutes_Veillonellales_Veillonellaceae_Veillonella_100
OTU_2267	7.07E-01	7.73E-01	8.93E-03	0.72	4.21	Bacteroides_kribbi_96	Bacteria_Bacteroidetes_Bacteroidia_Bacteroidales_Bacteroidaceae_Bacteroides_90
OTU_234	8.16E-01	6.04E-01	1.38E-01	4.54	1.10	Eubacterium_rectale_95	Bacteria_Firmicutes_Clostridia_Clostridiales_Lachnospiraceae_Mediterraneibacter_63
OTU_5786	9.92E-01	8.40E-01	8.87E-01	2.31	2.22	Veillonella_parvula_98	Bacteria_Firmicutes_Negativicutes_Veillonellales_Veillonellaceae_Veillonella_100
OTU_583	9.60E-01	6.04E-01	6.29E-01	1.97	2.52	Acidaminococcus_fermentans_98	Bacteria_Firmicutes_Negativicutes_99_Acidaminococcales_99_Acidaminococcaceae_99_Acidaminococcus_98
OTU_1122	8.64E-01	6.50E-01	1.69E-01	2.29	3.24	Acidaminococcus_fermentans_88	Bacteria_Firmicutes_80_Negativicutes_80_Acidaminococcales_80_Acidaminococcaceae_80_Acidaminococcus_78
OTU_2115	1.00E+00	6.65E-01	5.91E-02	1.99	3.56	Lachnoclostridium_pacaense_95	Bacteria_Firmicutes_Clostridia_Clostridiales_Lachnospiraceae_Kineothrix_28
OTU_506	7.07E-01	6.63E-01	4.57E-01	0.55	0.83	Collinsella_aerofaciens_93	Bacteria_Actinobacteria_Coriobacteriia_Coriobacteriales_Coriobacteriaceae_Collinsella_98
OTU_7202	7.46E-01	6.04E-01	3.89E-01	2.36	3.11	Paraclostridium_benzoelyticum_97	Bacteria_Firmicutes_Clostridia_Clostridiales_Peptostreptococcaceae_Paraclostridium_95
OTU_720	8.64E-01	6.04E-01	1.43E-01	3.02	1.89	Peptoniphilus_tyrrelliae_95	Bacteria_Firmicutes_Clostridia_Clostridiales_Peptoniphilaceae_99_Peptoniphilus_99
OTU_1043	7.39E-01	6.20E-01	5.33E-02	4.70	0.81	Pantoea_beijingensis_88	Bacteria_Proteobacteria_97_Gammaproteobacteria_97_Enterobacterales_97_Enterobacteriaceae_92_Pseudescherichia_92
OTU_2844	7.07E-01	6.93E-01	8.49E-03	1.54	3.47	Phascolarctobacterium_faecium_96	Bacteria_Firmicutes_Negativicutes_Acidaminococcales_99_Acidaminococcaceae_99_Phascolarctobacterium_99
OTU_778	7.07E-01	8.28E-01	1.14E-02	5.21	0.38	Bacteroides_stercoris_100	Bacteria_Bacteroidetes_84_Bacteroidia_84_Bacteroidales_84_Bacteroidaceae_84_Mediterranea_68
OTU_899	9.67E-01	9.18E-01	2.77E-02	1.36	3.89	Enterocloster_lavalensis_92	Bacteria_Firmicutes_Clostridia_Clostridiales_Lachnospiraceae_78_Clostridium XlVa_24
OTU_1484	9.66E-01	6.04E-01	6.54E-03	4.83	1.35	Roseburia_inulinivorans_96	Bacteria_Firmicutes_Clostridia_Clostridiales_Lachnospiraceae_Roseburia_99
OTU_1702	7.07E-01	6.04E-01	1.60E-02	3.77	0.63	Bifidobacterium_longum_99	Bacteria_Actinobacteria_75_Actinobacteria_75_Bifidobacteriales_75_Bifidobacteriaceae_75_Pseudoscardovia_34
OTU_237	7.07E-01	9.94E-01	1.66E-01	5.87	0.82	Anaerococcus_mediterraneensis_100	Bacteria_Firmicutes_Clostridia_Clostridiales_Peptoniphilaceae_Anaerococcus_100
OTU_2985	8.79E-01	6.78E-01	1.90E-03	3.90	1.40	Bacteroides_faecis_89	Bacteria_Bacteroidetes_52_Bacteroidia_52_Bacteroidales_52_Bacteroidaceae_43_Bacteroides_32
OTU_5247	9.09E-01	9.49E-01	1.10E-01	2.08	1.16	Ruminococcus_gnavus_96	Bacteria_Firmicutes_Clostridia_Clostridiales_Lachnospiraceae_Dorea_63
OTU_6370	7.12E-01	7.24E-01	1.53E-03	4.03	1.49	Roseburia_inulinivorans_97	Bacteria_Firmicutes_Clostridia_Clostridiales_Lachnospiraceae_Roseburia_79
OTU_1130	8.68E-01	9.35E-01	8.37E-04	3.27	0.96	Pseudescherichia_vulneris_99	Bacteria_Proteobacteria_95_Gammaproteobacteria_95_Enterobacterales_95_Enterobacteriaceae_95_Pseudescherichia_88
OTU_129100	NA	9.25E-01	2.38E-02	0.00	6.01	Bacteroides_faecis_97	Bacteria_Bacteroidetes_Bacteroidia_Bacteroidales_Bacteroidaceae_Bacteroides_100
OTU_161	NA	9.41E-01	7.64E-02	0.00	2.37	Geobacter_sulfurreducens_87	Bacteria_Proteobacteria_94_Deltaproteobacteria_84_Myxococcales_65_Nannocystineae_38_Kofleriaceae_34_Kofleria_34
OTU_702	7.12E-01	6.04E-01	6.74E-02	0.45	3.31	Enterococcus_faecalis_92	Bacteria_Firmicutes_Bacilli_41_Lactobacillales_41_Carnobacteriaceae_39_Catellicoccus_35
OTU_823	7.07E-01	8.65E-01	2.51E-02	2.85	0.34	Bacteroides_fragilis_100	Bacteria_Bacteroidetes_Bacteroidia_Bacteroidales_Bacteroidaceae_93_Mediterranea_50
OTU_900	7.57E-01	9.54E-01	1.00E-01	0.99	1.93	Blautia_schinkii_92	Bacteria_Firmicutes_Clostridia_Clostridiales_Lachnospiraceae_Clostridium XlVa_31
OTU_1054	8.61E-01	6.04E-01	1.21E-01	1.53	0.52	Phocaeicola_plebeius_96	Bacteria_Bacteroidetes_Bacteroidia_Bacteroidales_Bacteroidaceae_Phocaeicola_50

OTU_4739	7.07E-01	7.40E-01	6.48E-03	4.75	1.34	Roseburia_inulinivorans_98	Bacteria_Firmicutes_70_Clostridia_70_Clostridiales_70_Lachnospiraceae_70_Roseburia_36
OTU_53500	8.06E-01	6.76E-01	2.03E-01	1.70	2.38	Enterococcus_faecalis_98	Bacteria_Firmicutes_Bacilli_Lactobacillales_99_Enterococcaceae_98_Enterococcus_94
OTU_667	7.82E-01	8.14E-01	8.25E-04	2.33	0.17	Bifidobacterium_catenulatum_96	Bacteria_Actinobacteria_Actinobacteria_Bifidobacteriales_Bifidobacteriaceae_Bifidobacterium_80
OTU_704	7.07E-01	9.89E-01	1.32E-02	1.94	0.26	Bifidobacterium_stercoris_98	Bacteria_Actinobacteria_90_Actinobacteria_90_Bifidobacteriales_90_Bifidobacteriaceae_90_Pseudoscardovia_44
OTU_11756	9.38E-01	6.50E-01	3.83E-02	1.77	2.84	Pseudescherichia_vulneris_92	Bacteria_Proteobacteria_98_Gammaproteobacteria_98_Enterobacterales_98_Enterobacteriaceae_94_Pseudescherichia_92
OTU_19483	7.57E-01	6.04E-01	2.46E-02	7.34	0.09	Prevotella_copri_96	Bacteria_Bacteroidetes_Bacteroidia_Bacteroidales_Prevotellaceae_Prevotella_98
OTU_2831	7.07E-01	7.99E-01	3.21E-02	3.35	2.08	Bacteroides_kribbi_96	Bacteria_Bacteroidetes_Bacteroidia_Bacteroidales_Bacteroidaceae_Bacteroides_99
OTU_6526	8.19E-01	9.12E-01	9.35E-03	3.49	1.06	Streptococcus_gordonii_98	Bacteria_Firmicutes_Bacilli_Lactobacillales_Streptococcaceae_Streptococcus_100
OTU_723	8.89E-01	8.28E-01	7.47E-01	0.70	0.60	Clostridium_perfringens_98	Bacteria_Firmicutes_62_Clostridia_62_Clostridiales_62_Clostridiaceae 1_62_Desnuesiella_26
OTU_1403	7.07E-01	7.16E-01	3.25E-01	2.37	3.26	Coprococcus_comes_97	Bacteria_Firmicutes_Clostridia_Clostridiales_Lachnospiraceae_Bariatricus_71
OTU_1438	1.00E+00	8.23E-01	1.74E-02	4.06	1.00	Hungatella_effluvii_96	Bacteria_Firmicutes_99_Clostridia_99_Clostridiales_99_Lachnospiraceae_99_Merdimonas_24
OTU_2195	1.00E+00	6.83E-01	2.48E-02	1.85	0.58	Phocaeicola_vulgatus_100	Bacteria_Bacteroidetes_88_Bacteroidia_88_Bacteroidales_88_Bacteroidaceae_88_Phocaeicola_69
OTU_611	8.23E-01	6.04E-01	2.43E-02	3.53	0.24	Vallitalea_guaymasensis_87	Bacteria_Firmicutes_92_Clostridia_91_Clostridiales_89_Ruminococcaceae_54_Pseudoclostridium_25
OTU_670	7.07E-01	6.04E-01	1.54E-01	3.19	0.19	Streptococcus_salivarius_97	Bacteria_Firmicutes_Bacilli_Lactobacillales_Streptococcaceae_Streptococcus_99
OTU_1148	9.64E-01	9.53E-01	3.40E-02	1.34	3.51	Mobilitalea_sibirica_89	Bacteria_Bacteroidetes_82_Bacteroidia_82_Bacteroidales_82_Bacteroidaceae_82_Bacteroides_74
OTU_983	9.67E-01	8.83E-01	6.66E-04	4.70	0.70	Oscillibacter_ruminantium_97	Bacteria_Firmicutes_Clostridia_Clostridiales_Ruminococcaceae_Oscillibacter_93
OTU_10637	7.07E-01	6.04E-01	1.83E-03	3.46	1.55	Pseudescherichia_vulneris_97	Bacteria_Proteobacteria_Gammaproteobacteria_Enterobacterales_Enterobacteriaceae_Escherichia/Shigella_74
OTU_2290	1.00E+00	6.04E-01	1.35E-01	1.56	3.30	Enterocloster_citroniae_98	Bacteria_Firmicutes_Erysipelotrichia_51_Erysipelotrichales_51_Erysipelatoclostridiaceae_51_Erysipelatoclostridium_51
OTU_3238	7.07E-01	7.73E-01	3.46E-02	2.31	3.55	Bacteroides_thetaiotaomicron_93	Bacteria_Bacteroidetes_97_Bacteroidia_97_Bacteroidales_97_Bacteroidaceae_97_Bacteroides_95
OTU_332	7.07E-01	6.04E-01	1.12E-01	5.22	0.57	Eubacterium_siraeum_93	Bacteria_Firmicutes_Clostridia_Clostridiales_Ruminococcaceae_98_Clostridium IV_47
OTU_584	7.07E-01	9.32E-01	8.88E-01	2.58	2.72	Holdemania_filiformis_100	Bacteria_Firmicutes_Erysipelotrichia_Erysipelotrichales_Erysipelotrichaceae_Holdemania_100
OTU_1379	8.25E-01	6.04E-01	9.20E-01	2.48	2.60	Senegalimassilia_anaerobia_100	Bacteria_Actinobacteria_Coriobacteriia_Coriobacteriales_Coriobacteriaceae_Senegalimassilia_100
OTU_1186	9.75E-01	9.35E-01	2.42E-02	1.58	3.90	Paraclostridium_benzoelyticum_96	Bacteria_Firmicutes_Clostridia_Clostridiales_Peptostreptococcaceae_Paraclostridium_98
OTU_1950	7.57E-01	9.25E-01	4.05E-02	1.46	3.68	Romboutsia_sedimentorum_88	Bacteria_Proteobacteria_95_Gammaproteobacteria_95_Enterobacterales_95_Enterobacteriaceae_94_Pseudescherichia_69
OTU_2888	9.93E-01	9.70E-01	2.01E-03	1.53	3.71	Phascolarctobacterium_faecium_97	Bacteria_Firmicutes_Negativicutes_Acidaminococcales_Acidaminococcaceae_Phascolarctobacterium_100
OTU_33498	7.12E-01	6.04E-01	2.58E-01	2.71	1.80	Pseudescherichia_vulneris_87	Bacteria_Proteobacteria_86_Gammaproteobacteria_86_Enterobacterales_86_Enterobacteriaceae_86_Pseudescherichia_85
OTU_718	7.07E-01	6.46E-01	5.39E-01	2.06	3.52	Veillonella_dispar_96	Bacteria_Firmicutes_Negativicutes_Veillonellales_Veillonellaceae_Veillonella_54
OTU_792	NA	6.27E-01	8.23E-02	0.00	4.40	Methyloversatilis_universalis_93	Bacteria_Proteobacteria_Gammaproteobacteria_53_Enterobacterales_52_Enterobacteriaceae_52_Pseudescherichia_43
OTU_1331	9.86E-01	6.04E-01	3.40E-04	4.34	0.95	Ruminococcus_gnavus_94	Bacteria_Firmicutes_Clostridia_Clostridiales_Lachnospiraceae_97_Dorea_40
OTU_5974	1.00E+00	8.83E-01	1.22E-01	0.83	3.54	Ralstonia_pickettii_97	Bacteria_Proteobacteria_Betaproteobacteria_Burkholderiales_Burkholderiaceae_Ralstonia_100
OTU_1124	7.57E-01	6.05E-01	1.35E-02	1.02	0.24	Phocaeicola_massiliensis_97	Bacteria_Bacteroidetes_Bacteroidia_Bacteroidales_Bacteroidaceae_Phocaeicola_99
OTU_1516	7.89E-01	8.11E-01	4.10E-03	1.24	3.55	Veillonella_dispar_91	Bacteria_Firmicutes_86_Negativicutes_86_Veillonellales_82_Veillonellaceae_82_Veillonella_82
OTU_366	7.07E-01	6.04E-01	4.14E-01	3.02	1.69	Kineothrix_alysoides_98	Bacteria_Firmicutes_Clostridia_Clostridiales_Lachnospiraceae_Enterocloster_27
OTU_5253	8.67E-01	6.05E-01	1.87E-02	3.14	0.79	Flintibacter_butyricus_99	Bacteria_Firmicutes_Clostridia_Clostridiales_Ruminococcaceae_Flintibacter_100
OTU_855	7.07E-01	6.94E-01	4.03E-03	5.76	0.23	Streptococcus_peroris_98	Bacteria_Bacteroidetes_91_Bacteroidia_90_Bacteroidales_90_Bacteroidaceae_76_Mediterranea_67
OTU_1039	8.06E-01	6.04E-01	1.46E-01	0.90	0.40	Phocaeicola_vulgatus_94	Bacteria_Bacteroidetes_Bacteroidia_Bacteroidales_Bacteroidaceae_Phocaeicola_100
OTU_26852	9.87E-01	9.61E-01	6.63E-02	0.73	0.32	Bacteroides_caecimuris_94	Bacteria_Bacteroidetes_Bacteroidia_Bacteroidales_Bacteroidaceae_Bacteroides_96
OTU_15569	8.82E-01	9.46E-01	8.33E-01	1.97	2.10	Phocaeicola_plebeius_97	Bacteria_Bacteroidetes_Bacteroidia_Bacteroidales_Bacteroidaceae_Phocaeicola_67
OTU_18074	9.78E-01	9.75E-01	2.69E-01	2.63	2.01	Enterocloster_citroniae_97	Bacteria_Firmicutes_Clostridia_Clostridiales_Lachnospiraceae_Enterocloster_66
OTU_25764	8.06E-01	8.99E-01	4.68E-01	2.12	2.54	Enterococcus_faecalis_97	Bacteria_Firmicutes_Bacilli_Lactobacillales_Enterococcaceae_82_Enterococcus_45
OTU_357	7.57E-01	8.65E-01	2.50E-01	1.42	0.57	Mitsuokella_multacida_98	Bacteria_Firmicutes_Negativicutes_Selenomonadales_Selenomonadaceae_Mitsuokella_100
OTU_849	7.85E-01	9.42E-01	3.57E-01	2.94	2.10	Paraclostridium_benzoelyticum_94	Bacteria_Firmicutes_Clostridia_Clostridiales_Peptostreptococcaceae_96_Paraclostridium_96
OTU_87577	9.67E-01	9.12E-01	9.08E-03	4.15	0.41	Streptococcus_mitis_98	Bacteria_Firmicutes_Bacilli_Lactobacillales_Streptococcaceae_Streptococcus_100
OTU_2177	7.91E-01	6.63E-01	7.06E-01	2.48	2.91	Veillonella_parvula_90	Bacteria_Firmicutes_Clostridia_98_Clostridiales_98_Lachnospiraceae_98_Bariatricus_78
OTU_2803	7.46E-01	7.36E-01	4.74E-02	1.54	3.06	Enterococcus_hirae_96	Bacteria_Firmicutes_Bacilli_91_Lactobacillales_91_Enterococcaceae_74_Vagococcus_20
OTU_6606	7.12E-01	8.59E-01	2.16E-01	2.04	2.69	Pseudescherichia_vulneris_100	Bacteria_Proteobacteria_Gammaproteobacteria_Enterobacterales_Enterobacteriaceae_99_Pseudescherichia_88
OTU_4643	7.94E-01	7.06E-01	5.78E-02	2.20	0.43	Bacteroides_uniformis_97	Bacteria_Bacteroidetes_Bacteroidia_Bacteroidales_Bacteroidaceae_Bacteroides_54
OTU_7189	9.59E-01	9.17E-01	3.61E-03	0.25	4.62	Phocaeicola_plebeius_96	Bacteria_Bacteroidetes_Bacteroidia_Bacteroidales_Bacteroidaceae_Phocaeicola_84
OTU_14508	7.85E-01	6.99E-01	4.58E-01	2.28	2.60	Enterocloster_clostridioformis_97	Bacteria_Firmicutes_Clostridia_Clostridiales_Lachnospiraceae_Enterocloster_65
OTU_1570	7.57E-01	9.17E-01	3.28E-01	2.85	2.18	Veillonella_parvula_96	Bacteria_Firmicutes_Negativicutes_Veillonellales_Veillonellaceae_Veillonella_100
OTU_1869	8.25E-01	6.61E-01	2.25E-03	3.98	0.63	Shigella_dysenteriae_88	Bacteria_Proteobacteria_84_Gammaproteobacteria_84_Enterobacterales_81_Enterobacteriaceae_79_Pseudescherichia_78
OTU_2148	7.07E-01	7.31E-01	7.08E-01	0.26	0.43	Haemophilus_parainfluenzae_98	Bacteria_Proteobacteria_Gammaproteobacteria_Pasteurellales_Pasteurellaceae_Haemophilus_99
OTU_7544	7.07E-01	6.04E-01	4.81E-09	4.79	0.44	Bacteroides_kribbi_96	Bacteria_Bacteroidetes_Bacteroidia_Bacteroidales_Bacteroidaceae_97_Bacteroides_97
OTU_4649	7.88E-01	7.88E-01	3.28E-04	1.89	0.23	Phocaeicola_vulgatus_96	Bacteria_Bacteroidetes_Bacteroidia_Bacteroidales_Bacteroidaceae_Phocaeicola_61
OTU_619	7.39E-01	7.30E-01	1.50E-02	5.28	0.26	Clostridium_perfringens_91	Bacteria_Firmicutes_81_Clostridia_81_Clostridiales_81_Clostridiaceae 1_80_Sarcina_32
OTU_1150	9.67E-01	6.04E-01	6.80E-02	0.75	2.28	Enterococcus_faecalis_93	Bacteria_Firmicutes_Bacilli_91_Lactobacillales_88_Carnobacteriaceae_49_Catellicoccus_23
OTU_12605	8.60E-01	9.70E-01	9.34E-01	2.22	2.18	Lachnoclostridium_pacaense_97	Bacteria_Firmicutes_Clostridia_Clostridiales_Lachnospiraceae_Enterocloster_39
OTU_2085	9.87E-01	6.04E-01	5.02E-01	0.55	0.38	Alistipes_shahii_100	Bacteria_Bacteroidetes_Bacteroidia_Bacteroidales_Rikenellaceae_Alistipes_100
OTU_22668	7.14E-01	7.07E-01	2.63E-01	2.09	2.91	Veillonella_tobetsuensis_99	Bacteria_Firmicutes_Negativicutes_Veillonellales_Veillonellaceae_Veillonella_100
OTU_3019	7.62E-01	6.47E-01	5.16E-02	3.31	1.44	Vagococcus_humatus_90	Bacteria_Firmicutes_Negativicutes_52_Selenomonadales_48_Sporomusaceae_47_Anaerosinus_44
OTU_4087	7.80E-01	6.63E-01	7.87E-02	1.30	3.39	Pseudescherichia_vulneris_97	Bacteria_Proteobacteria_Gammaproteobacteria_Enterobacterales_Enterobacteriaceae_Escherichia/Shigella_62
OTU_72010	7.07E-01	6.24E-01	1.43E-01	1.13	0.25	Faecalibacterium_prausnitzii_98	Bacteria_Firmicutes_Clostridia_Clostridiales_Ruminococcaceae_Faecalibacterium_100
OTU_2707	8.06E-01	7.30E-01	7.92E-02	2.93	1.80	Clostridium_symbiosum_96	Bacteria_Firmicutes_Clostridia_Clostridiales_Lachnospiraceae_Clostridium XlVa_58
OTU_35794	7.57E-01	9.17E-01	5.79E-05	1.05	4.01	Bacteroides_thetaiotaomicron_100	Bacteria_Bacteroidetes_80_Bacteroidia_80_Bacteroidales_80_Bacteroidaceae_80_Mediterranea_47
OTU_40831	8.06E-01	9.34E-01	3.67E-04	5.30	0.28	Bacteroides_xylanisolvens_97	Bacteria_Bacteroidetes_Bacteroidia_Bacteroidales_Bacteroidaceae_Bacteroides_100
OTU_41756	8.06E-01	6.10E-01	3.11E-02	4.38	0.34	Veillonella_parvula_98	Bacteria_Firmicutes_Negativicutes_Veillonellales_Veillonellaceae_Veillonella_100
OTU_10023	7.10E-01	6.05E-01	5.47E-01	2.59	1.26	Citrobacter_koseri_98	Bacteria_Proteobacteria_Gammaproteobacteria_Enterobacterales_Enterobacteriaceae_Cronobacter_44

OTU_1385	1.00E+00	6.96E-01	1.08E-04	3.56	0.69	Parabacteroides_distasonis_98	Bacteria_Proteobacteria_56_Gammaproteobacteria_56_Enterobacterales_56_Enterobacteriaceae_56_Pseudescherichia_56
OTU_29627	7.59E-01	6.04E-01	1.06E-01	3.04	1.36	Veillonella_tobetsuensis_98	Bacteria_Firmicutes_Negativicutes_Veillonellales_Veillonellaceae_Veillonella_100
OTU_638	7.07E-01	7.36E-01	7.45E-01	0.89	0.75	Klebsiella_pneumoniae_98	Bacteria_Proteobacteria_92_Gammaproteobacteria_92_Enterobacterales_90_Enterobacteriaceae_89_Leclercia_52
OTU_6492	9.14E-01	7.21E-01	9.42E-01	1.96	2.01	Enterocloster_clostridioformis_96	Bacteria_Firmicutes_Clostridia_Clostridiales_Lachnospiraceae_Clostridium XlVa_61
OTU_2250	9.73E-01	8.62E-01	2.41E-02	2.12	3.31	Roseburia_inulinivorans_94	Bacteria_Firmicutes_99_Clostridia_99_Clostridiales_99_Lachnospiraceae_99_Falcatimonas_17
OTU_1400	8.97E-01	9.35E-01	1.07E-02	0.61	3.89	Clostridium_symbiosum_96	Bacteria_Firmicutes_Negativicutes_44_Acidaminococcales_43_Acidaminococcaceae_43_Phascolarctobacterium_43
OTU_9775	8.57E-01	9.32E-01	9.58E-04	1.43	2.83	Escherichia_albertii_90	Bacteria_Proteobacteria_86_Gammaproteobacteria_86_Enterobacterales_86_Enterobacteriaceae_86_Pseudescherichia_84
OTU_1166	9.43E-01	6.04E-01	9.16E-02	2.44	1.46	Pseudescherichia_vulneris_93	Bacteria_Proteobacteria_70_Gammaproteobacteria_70_Enterobacterales_70_Enterobacteriaceae_69_Pseudescherichia_66
OTU_2455	8.97E-01	8.62E-01	7.31E-01	2.01	1.80	Falcatimonas_natans_91	Bacteria_Firmicutes_Clostridia_76_Clostridiales_76_Lachnospiraceae_76_Eisenbergiella_14
OTU_2461	9.66E-01	9.53E-01	1.72E-02	2.75	1.57	Pseudescherichia_vulneris_96	Bacteria_Proteobacteria_86_Gammaproteobacteria_86_Enterobacterales_86_Enterobacteriaceae_86_Pseudescherichia_80
OTU_608	8.25E-01	6.04E-01	2.70E-02	3.52	0.24	Vallitalea_pronyensis_86	Bacteria_Firmicutes_99_Clostridia_90_Clostridiales_85_Clostridiales_Incertae Sedis XIII_14_Anaerovorax_13
OTU_3583	8.32E-01	9.75E-01	3.15E-03	3.79	0.63	Veillonella_dispar_89	Bacteria_Firmicutes_91_Negativicutes_83_Veillonellales_67_Veillonellaceae_67_Veillonella_65
OTU_480	7.57E-01	6.14E-01	2.13E-01	3.85	1.14	Oscillibacter_ruminantium_94	Bacteria_Firmicutes_Clostridia_Clostridiales_Ruminococcaceae_Oscillibacter_58
OTU_967	7.07E-01	6.04E-01	1.40E-02	1.26	3.68	Bacteroides_thetaiotaomicron_100	Bacteria_Bacteroidetes_73_Bacteroidia_73_Bacteroidales_73_Bacteroidaceae_73_Bacteroides_62
OTU_2259	7.07E-01	6.16E-01	2.97E-02	0.62	3.44	Bacteroides_kribbi_96	Bacteria_Bacteroidetes_Bacteroidia_Bacteroidales_Bacteroidaceae_Bacteroides_95
OTU_589	9.67E-01	7.80E-01	8.70E-01	0.32	0.27	Bacteroides_gallinaceum_93	Bacteria_Bacteroidetes_Bacteroidia_Bacteroidales_Bacteroidaceae_Phocaeicola_76
OTU_592	7.97E-01	9.59E-01	4.45E-02	4.28	1.01	Clostridium_carnis_90	Bacteria_Proteobacteria_59_Betaproteobacteria_58_Burkholderiales_56_Sutterellaceae_53_Sutterella_50
OTU_949	9.72E-01	6.41E-01	3.82E-03	3.30	0.14	Acidaminococcus_intestini_92	Bacteria_Firmicutes_Negativicutes_Acidaminococcales_Acidaminococcaceae_Phascolarctobacterium_99
OTU_959	8.33E-01	6.04E-01	8.43E-01	1.03	1.15	Faecalibacterium_prausnitzii_97	Bacteria_Firmicutes_Clostridia_Clostridiales_Ruminococcaceae_Faecalibacterium_98
OTU_9739	8.48E-01	9.12E-01	6.24E-03	1.32	2.41	Pseudescherichia_vulneris_95	Bacteria_Proteobacteria_Gammaproteobacteria_Enterobacterales_Enterobacteriaceae_Pseudescherichia_64
OTU_16000	1.00E+00	6.04E-01	2.37E-02	1.92	0.24	Bacteroides_stercoris_93	Bacteria_Bacteroidetes_Bacteroidia_Bacteroidales_Bacteroidaceae_Phocaeicola_97
OTU_3990	1.00E+00	7.97E-01	2.59E-02	2.54	1.55	Enterocloster_asparagiformis_89	Bacteria_Proteobacteria_71_Gammaproteobacteria_71_Enterobacterales_71_Enterobacteriaceae_70_Pseudescherichia_66
OTU_719	7.57E-01	6.04E-01	7.26E-01	2.31	1.96	Peptoniphilus_grossensis_96	Bacteria_Firmicutes_Clostridia_Clostridiales_Peptoniphilaceae_Peptoniphilus_100
OTU_7247	7.91E-01	6.50E-01	3.53E-02	3.68	1.36	Roseburia_inulinivorans_91	Bacteria_Firmicutes_Clostridia_63_Clostridiales_63_Lachnospiraceae_62_Bariatricus_14
OTU_13609	8.85E-01	6.04E-01	1.10E-01	2.93	2.05	Pseudescherichia_vulneris_100	Bacteria_Proteobacteria_99_Gammaproteobacteria_99_Enterobacterales_99_Enterobacteriaceae_99_Pseudescherichia_80
OTU_954	7.12E-01	6.15E-01	6.49E-03	4.45	0.87	Clostridium_symbiosum_95	Bacteria_Bacteroidetes_64_Bacteroidia_63_Bacteroidales_63_Bacteroidaceae_61_Mediterranea_39
OTU_1597	8.48E-01	6.72E-01	8.52E-02	2.68	1.83	Coprococcus_comes_96	Bacteria_Firmicutes_Clostridia_Clostridiales_Lachnospiraceae_Bariatricus_79
OTU_352	7.62E-01	6.04E-01	6.96E-01	0.71	0.57	Sutterella_stercoricanis_98	Bacteria_Proteobacteria_Betaproteobacteria_Burkholderiales_Sutterellaceae_Sutterella_100
OTU_51294	9.05E-01	9.97E-01	1.30E-03	3.18	0.50	Parabacteroides_distasonis_95	Bacteria_Bacteroidetes_Bacteroidia_Bacteroidales_Porphyromonadaceae_90_Parabacteroides_90
OTU_596	8.25E-01	9.09E-01	4.87E-02	0.89	0.20	Sutterella_wadsworthensis_97	Bacteria_Proteobacteria_Betaproteobacteria_Burkholderiales_Sutterellaceae_Sutterella_100
OTU_652	7.45E-01	6.05E-01	1.09E-01	3.25	1.00	Fusobacterium_mortiferum_92	Bacteria_Fusobacteria_Fusobacteriia_Fusobacteriales_Fusobacteriaceae_91_Cetobacterium_53
OTU_875	7.82E-01	6.04E-01	9.84E-01	2.00	2.01	Lacrimispora_xylanolytica_97	Bacteria_Firmicutes_Clostridia_Clostridiales_Lachnospiraceae_Ruminococcus2_19
OTU_782	7.39E-01	6.63E-01	7.60E-02	0.54	2.17	Phocaeicola_plebeius_97	Bacteria_Bacteroidetes_84_Bacteroidia_84_Bacteroidales_84_Bacteroidaceae_84_Phocaeicola_75
OTU_902	7.10E-01	6.14E-01	6.82E-01	1.88	2.27	Pseudescherichia_vulneris_97	Bacteria_Proteobacteria_Gammaproteobacteria_Enterobacterales_Enterobacteriaceae_Escherichia/Shigella_78
OTU_11321	9.59E-01	9.81E-01	6.77E-04	4.00	0.44	Bacteroides_caccae_97	Bacteria_Bacteroidetes_Bacteroidia_Bacteroidales_Bacteroidaceae_Bacteroides_100
OTU_1345	7.07E-01	6.04E-01	4.37E-01	1.63	2.87	Dialister_propionicifaciens_93	Bacteria_Firmicutes_Negativicutes_Veillonellales_99_Veillonellaceae_99_Dialister_98
OTU_1406	7.46E-01	6.72E-01	8.33E-01	2.10	1.94	Clostridium_perfringens_100	Bacteria_Firmicutes_84_Clostridia_84_Clostridiales_84_Clostridiaceae 1_84_Desnuesiella_37
OTU_1455	7.07E-01	6.04E-01	5.64E-01	1.54	2.10	Dialister_propionicifaciens_97	Bacteria_Firmicutes_Negativicutes_70_Veillonellales_70_Veillonellaceae_70_Dialister_69
OTU_1475	8.48E-01	6.04E-01	2.32E-02	1.26	3.27	Enterococcus_faecalis_95	Bacteria_Firmicutes_99_Bacilli_87_Lactobacillales_82_Enterococcaceae_51_Melissococcus_23
OTU_1954	9.37E-01	8.38E-01	6.72E-01	0.98	0.79	Blautia_wexlerae_96	Bacteria_Firmicutes_Clostridia_Clostridiales_Lachnospiraceae_Clostridium XlVa_26
OTU_2156	7.52E-01	6.04E-01	6.54E-02	3.88	0.33	Roseburia_inulinivorans_90	Bacteria_Firmicutes_Clostridia_86_Clostridiales_86_Lachnospiraceae_85_Bariatricus_36
OTU_7427	8.16E-01	6.04E-01	1.47E-01	2.48	1.72	Clostridium_perfringens_97	Bacteria_Firmicutes_Clostridia_Clostridiales_Clostridiaceae 1_90_Sarcina_32
OTU_961	7.10E-01	8.28E-01	3.57E-01	0.05	0.12	Bifidobacterium_callitrichidarum_90	Bacteria_Actinobacteria_98_Actinobacteria_98_Bifidobacteriales_96_Bifidobacteriaceae_96_Neoscardovia_46
OTU_14132	8.49E-01	6.05E-01	2.07E-01	2.83	2.01	Hungatella_effluvii_95	Bacteria_Firmicutes_84_Clostridia_84_Clostridiales_84_Lachnospiraceae_84_Merdimonas_24
OTU_925	7.07E-01	6.04E-01	3.03E-01	1.48	2.38	Clostridium_perfringens_97	Bacteria_Firmicutes_Clostridia_99_Clostridiales_99_Clostridiaceae 1_79_Clostridium sensu stricto_61
OTU_1132	1.00E+00	6.76E-01	2.79E-03	4.46	0.39	Clostridium_perfringens_92	Bacteria_Firmicutes_Clostridia_Clostridiales_Lachnospiraceae_56_Abyssivirga_20
OTU_18083	9.23E-01	9.72E-01	3.68E-01	2.31	1.92	Clostridium_symbiosum_96	Bacteria_Firmicutes_Clostridia_Clostridiales_Lachnospiraceae_Clostridium XlVa_43
OTU_2301	9.99E-01	6.63E-01	3.27E-02	0.96	2.87	Drancourtella_massiliensis_98	Bacteria_Firmicutes_98_Clostridia_98_Clostridiales_98_Lachnospiraceae_98_Sellimonas_98
OTU_2889	7.07E-01	4.60E-01	1.68E-02	3.43	1.54	Clostridium_scindens_94	Bacteria_Firmicutes_Clostridia_Clostridiales_Lachnospiraceae_90_Clostridium XlVa_70
OTU_3192	7.07E-01	9.35E-01	9.32E-01	1.13	1.08	Faecalibacterium_prausnitzii_97	Bacteria_Firmicutes_Clostridia_Clostridiales_Ruminococcaceae_Faecalibacterium_82
OTU_4755	9.06E-01	8.13E-01	1.81E-01	2.90	2.01	Clostridium_symbiosum_94	Bacteria_Firmicutes_Clostridia_Clostridiales_Lachnospiraceae_Coprococcus_52
OTU_1785	8.83E-01	9.18E-01	3.36E-03	0.88	4.67	Phocaeicola_coprocola_95	Bacteria_Bacteroidetes_Bacteroidia_Bacteroidales_Bacteroidaceae_Bacteroides_74
OTU_3501	9.38E-01	9.76E-01	5.55E-01	2.70	2.37	Roseburia_inulinivorans_97	Bacteria_Firmicutes_Clostridia_Clostridiales_Lachnospiraceae_Roseburia_66
OTU_64956	7.07E-01	6.04E-01	6.54E-01	0.92	1.20	Veillonella_denticariosi_98	Bacteria_Firmicutes_Negativicutes_Veillonellales_Veillonellaceae_Veillonella_100
OTU_8089	9.64E-01	6.37E-01	2.63E-02	2.78	1.29	Enterocloster_citroniae_96	Bacteria_Firmicutes_Clostridia_Clostridiales_Lachnospiraceae_Enterocloster_65
OTU_10673	9.43E-01	6.82E-01	8.10E-03	2.75	1.53	Hungatella_effluvii_97	Bacteria_Firmicutes_Clostridia_Clostridiales_Lachnospiraceae_Hungatella_80
OTU_2136	7.91E-01	8.65E-01	4.17E-01	2.20	1.76	Shigella_boydii_89	Bacteria_Proteobacteria_Gammaproteobacteria_Enterobacterales_Enterobacteriaceae_Pseudescherichia_85
OTU_586	7.46E-01	6.04E-01	8.59E-02	3.63	1.12	Enterococcus_dispar_98	Bacteria_Firmicutes_57_Bacilli_56_Lactobacillales_53_Carnobacteriaceae_46_Isobaculum_37
OTU_691	1.00E+00	6.04E-01	3.03E-01	0.87	0.45	Catenibacterium_mitsuokai_96	Bacteria_Firmicutes_Erysipelotrichia_92_Erysipelotrichales_92_Erysipelotrichaceae_92_Catenibacterium_69
OTU_775	7.07E-01	7.99E-01	1.64E-02	0.38	1.43	Phocaeicola_coprophilus_99	Bacteria_Bacteroidetes_88_Bacteroidia_88_Bacteroidales_88_Bacteroidaceae_88_Phocaeicola_62
OTU_1437	7.46E-01	9.64E-01	5.25E-03	4.53	0.26	Bacteroides_uniformis_95	Bacteria_Bacteroidetes_Bacteroidia_Bacteroidales_Bacteroidaceae_80_Bacteroides_77
OTU_386	9.14E-01	9.32E-01	9.68E-01	0.79	0.81	Veillonella_dispar_98	Bacteria_Firmicutes_Negativicutes_76_Veillonellales_65_Veillonellaceae_65_Veillonella_62
OTU_4100	7.07E-01	9.46E-01	3.33E-01	3.05	1.60	Veillonella_atypica_96	Bacteria_Firmicutes_Negativicutes_Veillonellales_Veillonellaceae_Veillonella_99
OTU_468	9.87E-01	9.77E-01	3.36E-03	4.60	0.30	Clostridium_perfringens_95	Bacteria_Firmicutes_Clostridia_Clostridiales_Clostridiaceae 1_82_Sarcina_43
OTU_8745	8.06E-01	6.72E-01	6.72E-01	2.39	2.10	Ruminococcus_torques_97	Bacteria_Firmicutes_Clostridia_Clostridiales_Lachnospiraceae_Mediterraneibacter_78
OTU_903	9.31E-01	7.58E-01	2.23E-01	1.49	2.30	Enterococcus_faecalis_90	Bacteria_Firmicutes_Bacilli_40_Lactobacillales_39_Carnobacteriaceae_36_Catellicoccus_36
OTU_1555	9.43E-01	8.14E-01	1.82E-01	2.29	1.65	Enterococcus_hirae_96	Bacteria_Firmicutes_99_Bacilli_98_Lactobacillales_98_Enterococcaceae_90_Vagococcus_40

OTU_294	9.56E-01	9.46E-01	8.04E-01	0.22	0.26	Haemophilus_parainfluenzae_90	Bacteria_Proteobacteria_Gammaproteobacteria_Pasteurellales_Pasteurellaceae_Conservatibacter_55
OTU_764	7.07E-01	6.10E-01	9.89E-02	1.02	2.82	Clostridium_symbiosum_92	Bacteria_Firmicutes_Clostridia_Clostridiales_Lachnospiraceae_78_Clostridium XlVa_46
OTU_877	8.44E-01	9.17E-01	5.52E-03	0.45	1.18	Enterococcus_dispar_97	Bacteria_Firmicutes_59_Bacilli_59_Lactobacillales_56_Carnobacteriaceae_54_Isobaculum_44
OTU_4206	7.23E-01	9.19E-01	3.81E-02	3.02	1.66	Coprococcus_comes_98	Bacteria_Firmicutes_94_Clostridia_94_Clostridiales_94_Lachnospiraceae_94_Bariatricus_62
OTU_4601	9.04E-01	6.04E-01	5.05E-01	0.48	0.67	Fusicatenibacter_saccharivorans_95	Bacteria_Firmicutes_Clostridia_Clostridiales_Lachnospiraceae_Coprococcus_17
OTU_835	7.07E-01	6.04E-01	5.81E-01	2.60	1.97	Paraclostridium_benzoelyticum_94	Bacteria_Firmicutes_Clostridia_Clostridiales_Peptostreptococcaceae_98_Paraclostridium_98
OTU_1748	7.10E-01	9.54E-01	1.31E-01	2.67	1.41	Coprococcus_comes_92	Bacteria_Firmicutes_97_Clostridia_97_Clostridiales_97_Lachnospiraceae_97_Bariatricus_72
OTU_503	7.07E-01	8.65E-01	1.84E-01	3.86	0.17	Fusobacterium_mortiferum_96	Bacteria_Fusobacteria_Fusobacteriia_Fusobacteriales_Fusobacteriaceae_99_Cetobacterium_70
OTU_34033	7.59E-01	6.65E-01	6.92E-02	1.07	1.86	Enterococcus_faecalis_98	Bacteria_Firmicutes_Bacilli_Lactobacillales_99_Enterococcaceae_97_Enterococcus_68
OTU_448	7.07E-01	6.05E-01	2.32E-02	2.75	0.89	Adlercreutzia_equolifaciens_100	Bacteria_Actinobacteria_Coriobacteriia_Eggerthellales_Eggerthellaceae_Adlercreutzia_100
OTU_469	9.99E-01	9.62E-01	6.04E-03	1.85	0.21	Megasphaera_elsdenii_90	Bacteria_Firmicutes_68_Negativicutes_68_Veillonellales_68_Veillonellaceae_68_Megasphaera_64
OTU_104221	7.07E-01	6.74E-01	6.29E-01	2.73	1.58	Veillonella_tobetsuensis_98	Bacteria_Firmicutes_Negativicutes_Veillonellales_Veillonellaceae_Veillonella_100
OTU_662	7.07E-01	9.18E-01	1.87E-01	2.19	0.34	Phocaeicola_vulgatus_100	Bacteria_Bacteroidetes_Bacteroidia_Bacteroidales_Bacteroidaceae_Phocaeicola_95
OTU_8109	7.93E-01	9.35E-01	8.20E-02	1.01	2.29	Lachnoclostridium_pacaense_94	Bacteria_Firmicutes_Clostridia_Clostridiales_Lachnospiraceae_Clostridium XlVa_39
OTU_1188	7.57E-01	8.10E-01	3.34E-02	1.09	3.58	Paraclostridium_bifermentans_98	Bacteria_Firmicutes_Clostridia_Clostridiales_Peptostreptococcaceae_Paraclostridium_98
OTU_1499	7.10E-01	8.14E-01	5.88E-01	2.35	1.98	Clostridium_scindens_95	Bacteria_Firmicutes_Clostridia_Clostridiales_Lachnospiraceae_Clostridium XlVa_87
OTU_18446	8.25E-01	9.90E-01	5.96E-02	1.75	2.42	Escherichia_albertii_88	Bacteria_Proteobacteria_60_Gammaproteobacteria_60_Enterobacterales_60_Enterobacteriaceae_60_Pseudescherichia_60
OTU_2127	9.45E-01	9.28E-01	8.38E-05	0.72	4.03	Bacteroides_thetaiotaomicron_96	Bacteria_Bacteroidetes_Bacteroidia_Bacteroidales_Bacteroidaceae_Bacteroides_93
OTU_2206	7.57E-01	7.73E-01	2.80E-02	2.26	0.86	Dorea_longicatena_96	Bacteria_Firmicutes_Clostridia_Clostridiales_Lachnospiraceae_Dorea_91
OTU_331	8.85E-01	6.04E-01	9.64E-03	4.36	0.18	Desulfovibrio_intestinalis_96	Bacteria_Proteobacteria_Deltaproteobacteria_Desulfovibrionales_Desulfovibrionaceae_97_Desulfovibrio_75
OTU_7220	9.31E-01	6.05E-01	7.76E-01	1.91	1.68	Acidaminococcus_fermentans_95	Bacteria_Firmicutes_Negativicutes_Acidaminococcales_Acidaminococcaceae_Acidaminococcus_100
OTU_726	7.59E-01	7.66E-01	5.83E-02	4.69	0.42	Roseburia_inulinivorans_96	Bacteria_Firmicutes_Clostridia_Clostridiales_Lachnospiraceae_Roseburia_76
OTU_7542	7.57E-01	8.60E-01	1.89E-06	1.08	3.60	Bacteroides_thetaiotaomicron_90	Bacteria_Proteobacteria_38_Gammaproteobacteria_38_Enterobacterales_38_Enterobacteriaceae_38_Pseudescherichia_38
OTU_79802	7.10E-01	6.04E-01	8.03E-01	1.96	2.18	Veillonella_parvula_98	Bacteria_Firmicutes_Negativicutes_Veillonellales_Veillonellaceae_Veillonella_100
OTU_1835	7.85E-01	6.66E-01	3.81E-01	1.75	2.13	Enterocloster_clostridioformis_95	Bacteria_Firmicutes_Clostridia_Clostridiales_Lachnospiraceae_Enterocloster_97
OTU_2242	7.57E-01	7.08E-01	4.31E-03	1.10	3.23	Gibbsiella_dentisursi_90	Bacteria_Proteobacteria_Gammaproteobacteria_Enterobacterales_Enterobacteriaceae_Pseudescherichia_95
OTU_7207	8.85E-01	6.04E-01	1.20E-01	3.18	1.78	Enterocloster_aldenensis_96	Bacteria_Firmicutes_Clostridia_Clostridiales_Lachnospiraceae_Coprococcus_11
OTU_853	7.07E-01	6.06E-01	1.89E-01	3.62	0.53	Streptococcus_salivarius_97	Bacteria_Firmicutes_Bacilli_Lactobacillales_Streptococcaceae_97_Streptococcus_96
OTU_17734	7.62E-01	9.42E-01	8.52E-04	0.81	3.15	Veillonella_dispar_96	Bacteria_Firmicutes_99_Negativicutes_95_Veillonellales_92_Veillonellaceae_92_Veillonella_92
OTU_19764	9.71E-01	8.94E-01	3.96E-02	2.08	3.23	Bacteroides_thetaiotaomicron_98	Bacteria_Bacteroidetes_Bacteroidia_Bacteroidales_Bacteroidaceae_Bacteroides_100
OTU_231	7.46E-01	NA	1.24E-01	1.28	0.00	Bacteroides_thetaiotaomicron_97	Bacteria_Bacteroidetes_Bacteroidia_Bacteroidales_Bacteroidaceae_Bacteroides_100
OTU_1380	7.12E-01	9.89E-01	1.64E-02	3.07	0.16	Clostridium_symbiosum_98	Bacteria_Firmicutes_Clostridia_99_Clostridiales_99_Lachnospiraceae_99_Clostridium XlVa_32
OTU_1392	8.85E-01	9.77E-01	5.63E-01	0.93	0.56	Megasphaera_elsdenii_98	Bacteria_Firmicutes_Negativicutes_Veillonellales_Veillonellaceae_Megasphaera_100
OTU_242	9.38E-01	9.94E-01	6.49E-02	6.32	0.14	Lactobacillus_intestinalis_100	Bacteria_Firmicutes_Bacilli_Lactobacillales_Lactobacillaceae_Lactobacillus_100
OTU_324	1.00E+00	8.89E-01	5.17E-01	0.22	0.36	Phocaeicola_vulgatus_93	Bacteria_Bacteroidetes_Bacteroidia_Bacteroidales_Bacteroidaceae_Phocaeicola_84
OTU_373	7.07E-01	9.00E-01	3.21E-02	0.87	0.24	Bifidobacterium_longum_92	Bacteria_Actinobacteria_Actinobacteria_99_Bifidobacteriales_99_Bifidobacteriaceae_99_Pseudoscardovia_44
OTU_8917	8.06E-01	6.05E-01	3.03E-03	1.13	2.89	Enterococcus_faecalis_94	Bacteria_Firmicutes_Bacilli_Lactobacillales_94_Carnobacteriaceae_47_Isobaculum_35
OTU_1957	8.91E-01	9.75E-01	1.46E-02	0.88	3.35	Pseudescherichia_vulneris_91	Bacteria_Proteobacteria_67_Gammaproteobacteria_67_Enterobacterales_67_Enterobacteriaceae_67_Pseudescherichia_66
OTU_639	8.29E-01	6.04E-01	8.55E-01	0.45	0.39	Haemophilus_parainfluenzae_98	Bacteria_Proteobacteria_98_Gammaproteobacteria_98_Pasteurellales_98_Pasteurellaceae_98_Conservatibacter_62
OTU_8557	9.65E-01	8.36E-01	5.24E-01	2.05	2.41	Bacteroides_uniformis_95	Bacteria_Bacteroidetes_Bacteroidia_Bacteroidales_Bacteroidaceae_Bacteroides_98
OTU_3109	7.07E-01	5.62E-01	2.53E-01	1.96	2.83	Erysipelatoclostridium_ramosum_95	Bacteria_Firmicutes_Erysipelotrichia_84_Erysipelotrichales_84_Erysipelatoclostridiaceae_82_Erysipelatoclostridium_82
OTU_34827	7.11E-01	9.41E-01	1.33E-02	2.45	0.28	Bifidobacterium_stercoris_98	Bacteria_Actinobacteria_Actinobacteria_Bifidobacteriales_Bifidobacteriaceae_Bifidobacterium_95
OTU_3868	8.82E-01	9.45E-01	6.48E-01	2.06	1.77	Coprococcus_comes_95	Bacteria_Firmicutes_Clostridia_Clostridiales_Lachnospiraceae_Coprococcus_39
OTU_169	9.69E-01	NA	2.06E-01	2.36	0.00	Butyrivibrio_crossotus_97	Bacteria_Firmicutes_Clostridia_Clostridiales_Lachnospiraceae_Clostridium XlVa_49
OTU_478	7.11E-01	6.14E-01	1.85E-01	3.56	0.90	Kineothrix_alysoides_97	Bacteria_Firmicutes_Clostridia_Clostridiales_Lachnospiraceae_Kineothrix_78
OTU_697	7.07E-01	6.46E-01	3.18E-02	2.22	0.11	Fusobacterium_perfoetens_91	Bacteria_Fusobacteria_Fusobacteriia_Fusobacteriales_Fusobacteriaceae_92_Cetobacterium_55
OTU_1098	8.61E-01	6.04E-01	4.12E-02	3.80	0.45	Intestinimonas_butyriciproducens_97	Bacteria_Firmicutes_Clostridia_Clostridiales_Ruminococcaceae_Intestinimonas_61
OTU_1871	9.61E-01	6.05E-01	4.79E-03	3.56	1.06	Clostridium_gasigenes_89	Bacteria_Bacteroidetes_59_Bacteroidia_59_Bacteroidales_59_Bacteroidaceae_57_Mediterranea_37
OTU_3281	9.39E-01	9.57E-01	7.91E-02	1.20	2.03	Enterocloster_clostridioformis_96	Bacteria_Firmicutes_Clostridia_Clostridiales_Lachnospiraceae_Enterocloster_97
OTU_399	9.75E-01	6.05E-01	2.96E-02	4.39	0.32	Acetivibrio_alkalicellulosi_92	Bacteria_Firmicutes_98_Clostridia_98_Clostridiales_98_Ruminococcaceae_85_Colidextribacter_42
OTU_1889	8.61E-01	8.83E-01	5.25E-03	3.25	0.66	Clostridium_symbiosum_90	Bacteria_Firmicutes_Clostridia_Clostridiales_Lachnospiraceae_Clostridium XlVa_60
OTU_27573	7.07E-01	7.02E-01	1.04E-04	3.43	0.70	Bacteroides_xylanisolvens_98	Bacteria_Bacteroidetes_76_Bacteroidia_76_Bacteroidales_76_Bacteroidaceae_76_Bacteroides_63
OTU_605	8.05E-01	9.69E-01	2.59E-01	0.02	1.76	Sphingobium_phenoxybenzoativorans_99	Bacteria_Proteobacteria_Alphaproteobacteria_Sphingomonadales_Sphingomonadaceae_Sphingobium_93
OTU_7287	9.84E-01	8.83E-01	3.47E-03	1.09	3.07	Enterocloster_citroniae_100	Bacteria_Firmicutes_93_Clostridia_93_Clostridiales_93_Lachnospiraceae_93_Enterocloster_58
OTU_809	7.07E-01	6.04E-01	2.93E-01	1.71	3.21	Dialister_propionicifaciens_91	Bacteria_Firmicutes_Negativicutes_87_Veillonellales_87_Veillonellaceae_87_Dialister_84
OTU_1418	7.07E-01	8.28E-01	1.45E-02	2.17	0.49	Bifidobacterium_stercoris_97	Bacteria_Actinobacteria_Actinobacteria_Bifidobacteriales_Bifidobacteriaceae_Bifidobacterium_87
OTU_14648	9.38E-01	9.18E-01	1.01E-02	2.26	0.23	Bacteroides_uniformis_94	Bacteria_Bacteroidetes_Bacteroidia_Bacteroidales_Bacteroidaceae_Phocaeicola_77
OTU_3622	8.60E-01	9.94E-01	2.43E-02	1.76	2.58	Pseudescherichia_vulneris_93	Bacteria_Proteobacteria_99_Gammaproteobacteria_99_Enterobacterales_99_Enterobacteriaceae_98_Pseudescherichia_75
OTU_486	9.38E-01	9.60E-01	7.63E-01	1.23	1.01	Pseudescherichia_vulneris_99	Bacteria_Proteobacteria_90_Gammaproteobacteria_90_Enterobacterales_90_Enterobacteriaceae_90_Pseudescherichia_81
OTU_2114	7.82E-01	8.68E-01	5.50E-02	1.62	2.54	Bacteroides_faecis_91	Bacteria_Bacteroidetes_88_Bacteroidia_87_Bacteroidales_87_Bacteroidaceae_82_Bacteroides_47
OTU_27058	8.25E-01	6.72E-01	5.83E-02	1.39	2.56	Pseudescherichia_vulneris_97	Bacteria_Proteobacteria_Gammaproteobacteria_Enterobacterales_Enterobacteriaceae_Escherichia/Shigella_91
OTU_10263	7.07E-01	9.90E-01	1.67E-01	1.78	0.82	Collinsella_aerofaciens_96	Bacteria_Actinobacteria_Coriobacteriia_Coriobacteriales_Coriobacteriaceae_Collinsella_99
OTU_1201	7.57E-01	7.42E-01	3.60E-01	2.88	2.08	Faecalicatena_orotica_97	Bacteria_Firmicutes_Clostridia_Clostridiales_Lachnospiraceae_Clostridium XlVa_52
OTU_30118	7.07E-01	8.85E-01	2.03E-02	1.00	3.90	Bacteroides_thetaiotaomicron_97	Bacteria_Bacteroidetes_Bacteroidia_Bacteroidales_Bacteroidaceae_Bacteroides_100
OTU_3473	7.07E-01	6.04E-01	8.10E-03	3.16	0.66	Bifidobacterium_stercoris_96	Bacteria_Actinobacteria_89_Actinobacteria_89_Bifidobacteriales_89_Bifidobacteriaceae_89_Pseudoscardovia_40
OTU_912	9.52E-01	6.63E-01	6.29E-03	4.19	0.42	Clostridium_tarantellae_90	Bacteria_Firmicutes_92_Clostridia_92_Clostridiales_92_Clostridiaceae 1_91_Sarcina_51
OTU_105653	7.07E-01	9.17E-01	2.72E-01	1.32	0.14	Veillonella_rogosae_98	Bacteria_Firmicutes_Negativicutes_Veillonellales_Veillonellaceae_Veillonella_100

OTU_1480	8.54E-01	9.12E-01	9.17E-01	1.61	1.56	Phocea_massiliensis_92	Bacteria_Firmicutes_Clostridia_Clostridiales_Ruminococcaceae_Clostridium IV_56
OTU_1629	7.91E-01	6.04E-01	1.35E-02	2.03	0.68	Phocaeicola_vulgatus_94	Bacteria_Bacteroidetes_Bacteroidia_Bacteroidales_Bacteroidaceae_Phocaeicola_99
OTU_2015	8.06E-01	6.04E-01	4.46E-03	3.08	0.94	Bacteroides_uniformis_99	Bacteria_Bacteroidetes_80_Bacteroidia_80_Bacteroidales_80_Bacteroidaceae_77_Mediterranea_62
OTU_3234	7.07E-01	8.36E-01	9.87E-03	4.23	0.35	Bacteroides_stercoris_97	Bacteria_Bacteroidetes_Bacteroidia_Bacteroidales_Bacteroidaceae_Bacteroides_100
OTU_421	9.92E-01	6.14E-01	8.37E-02	2.67	0.61	Oscillibacter_valericigenes_95	Bacteria_Firmicutes_Clostridia_Clostridiales_Ruminococcaceae_Oscillibacter_53
OTU_4929	9.10E-01	9.13E-01	8.92E-01	2.23	2.32	Enterocloster_clostridioformis_96	Bacteria_Firmicutes_Clostridia_Clostridiales_Lachnospiraceae_Clostridium XlVa_89
OTU_51071	8.25E-01	8.83E-01	1.92E-01	1.47	0.63	Parabacteroides_distasonis_97	Bacteria_Bacteroidetes_Bacteroidia_Bacteroidales_Porphyromonadaceae_Parabacteroides_100
OTU_7222	9.96E-01	6.10E-01	5.43E-02	2.48	1.60	Enterocloster_aldenensis_97	Bacteria_Firmicutes_Clostridia_Clostridiales_Lachnospiraceae_Clostridium XlVa_44
OTU_84567	7.07E-01	7.07E-01	7.46E-02	4.66	0.34	Bacteroides_stercoris_96	Bacteria_Bacteroidetes_Bacteroidia_Bacteroidales_Bacteroidaceae_Bacteroides_100
OTU_1915	7.59E-01	7.11E-01	2.34E-02	0.66	2.87	Enterocloster_citroniae_100	Bacteria_Firmicutes_94_Clostridia_94_Clostridiales_94_Lachnospiraceae_94_Enterocloster_56
OTU_2955	7.57E-01	7.40E-01	1.95E-05	3.68	0.83	Parabacteroides_distasonis_96	Bacteria_Bacteroidetes_Bacteroidia_Bacteroidales_Porphyromonadaceae_81_Parabacteroides_81
OTU_440	9.35E-01	8.65E-01	3.64E-01	0.17	0.32	Haemophilus_parainfluenzae_99	Bacteria_Proteobacteria_Gammaproteobacteria_Pasteurellales_Pasteurellaceae_Conservatibacter_87
OTU_58010	8.06E-01	9.97E-01	4.37E-03	0.26	4.02	Bacteroides_kribbi_97	Bacteria_Bacteroidetes_Bacteroidia_Bacteroidales_Bacteroidaceae_Bacteroides_100
OTU_1139	7.59E-01	9.41E-01	7.83E-03	1.91	0.75	Faecalibacterium_prausnitzii_99	Bacteria_Firmicutes_Clostridia_Clostridiales_Ruminococcaceae_Faecalibacterium_100
OTU_268	7.07E-01	9.13E-01	3.52E-01	0.90	3.90	Granulicatella_adiacens_100	Bacteria_Firmicutes_Bacilli_Lactobacillales_Carnobacteriaceae_99_Granulicatella_98
OTU_3491	7.07E-01	7.31E-01	2.20E-02	2.83	0.86	Pseudescherichia_vulneris_89	Bacteria_Proteobacteria_Gammaproteobacteria_Enterobacterales_Enterobacteriaceae_Pseudescherichia_84
OTU_4145	9.46E-01	6.96E-01	5.52E-02	2.44	1.41	Pseudescherichia_vulneris_88	Bacteria_Proteobacteria_89_Gammaproteobacteria_89_Enterobacterales_89_Enterobacteriaceae_88_Pseudescherichia_85
OTU_524	8.97E-01	6.04E-01	2.04E-02	3.40	0.48	Flintibacter_butyricus_94	Bacteria_Firmicutes_Clostridia_Clostridiales_Ruminococcaceae_Colidextribacter_56
OTU_9973	8.58E-01	9.69E-01	7.48E-01	1.98	1.78	Rheinheimera_baltica_86	Bacteria_Proteobacteria_93_Gammaproteobacteria_93_Enterobacterales_93_Enterobacteriaceae_92_Pseudescherichia_91
OTU_1413	9.92E-01	7.40E-01	3.76E-02	0.69	2.59	Enterocloster_citroniae_94	Bacteria_Firmicutes_Clostridia_Clostridiales_Lachnospiraceae_Enterocloster_88
OTU_37426	7.07E-01	8.50E-01	2.91E-01	2.19	1.29	Pseudescherichia_vulneris_96	Bacteria_Proteobacteria_Gammaproteobacteria_Enterobacterales_Enterobacteriaceae_Escherichia/Shigella_59
OTU_454	9.67E-01	6.04E-01	3.63E-01	0.62	3.64	Bacteroides_fragilis_87	Bacteria_Fusobacteria_87_Fusobacteriia_87_Fusobacteriales_87_Fusobacteriaceae_85_Cetobacterium_61
OTU_472	7.07E-01	6.14E-01	2.54E-01	1.20	0.37	Bifidobacterium_stercoris_92	Bacteria_Actinobacteria_98_Actinobacteria_98_Bifidobacteriales_98_Bifidobacteriaceae_98_Pseudoscardovia_48
OTU_54955	9.94E-01	8.57E-01	9.42E-02	1.01	2.60	Lachnoclostridium_pacaense_97	Bacteria_Firmicutes_Clostridia_Clostridiales_Lachnospiraceae_Enterocloster_80
OTU_1032	7.07E-01	8.14E-01	9.22E-02	1.12	0.34	Phocaeicola_vulgatus_98	Bacteria_Bacteroidetes_Bacteroidia_Bacteroidales_Bacteroidaceae_Phocaeicola_98
OTU_1208	8.83E-01	6.66E-01	4.01E-01	0.13	0.26	Collinsella_aerofaciens_99	Bacteria_Actinobacteria_97_Coriobacteriia_97_Coriobacteriales_97_Coriobacteriaceae_97_Collinsella_96
OTU_62482	8.97E-01	9.46E-01	8.10E-03	1.03	2.31	Shigella_dysenteriae_95	Bacteria_Proteobacteria_Gammaproteobacteria_Enterobacterales_Enterobacteriaceae_Pseudescherichia_81
OTU_7032	7.57E-01	6.39E-01	8.76E-03	1.38	0.57	Blautia_luti_98	Bacteria_Firmicutes_Clostridia_Clostridiales_Lachnospiraceae_Blautia_96
OTU_7315	7.07E-01	6.04E-01	3.60E-01	1.57	2.19	Eubacterium_limosum_95	Bacteria_Firmicutes_Clostridia_Clostridiales_Eubacteriaceae_94_Eubacterium_60
OTU_8050	9.50E-01	7.34E-01	4.67E-02	1.62	3.07	Bacteroides_xylanisolvens_97	Bacteria_Bacteroidetes_Bacteroidia_Bacteroidales_Bacteroidaceae_Bacteroides_100
OTU_80939	9.38E-01	6.04E-01	1.50E-02	3.36	0.74	Bacteroides_xylanisolvens_98	Bacteria_Bacteroidetes_Bacteroidia_Bacteroidales_Bacteroidaceae_Bacteroides_100
OTU_83802	7.07E-01	8.28E-01	2.52E-01	2.83	0.39	Bacteroides_uniformis_94	Bacteria_Bacteroidetes_Bacteroidia_Bacteroidales_Bacteroidaceae_Phocaeicola_67
OTU_1532	9.87E-01	6.04E-01	1.41E-01	1.04	2.68	Pseudescherichia_vulneris_91	Bacteria_Proteobacteria_99_Gammaproteobacteria_99_Enterobacterales_99_Enterobacteriaceae_99_Pseudescherichia_70
OTU_1914	9.37E-01	9.17E-01	1.20E-03	2.54	0.88	Bacteroides_uniformis_96	Bacteria_Bacteroidetes_Bacteroidia_Bacteroidales_Bacteroidaceae_Bacteroides_100
OTU_282	9.31E-01	6.04E-01	4.86E-01	2.76	1.19	Anaeroplasma_abactoclasticum_91	Bacteria_Tenericutes_99_Mollicutes_99_Anaeroplasmatales_99_Anaeroplasmataceae_99_Anaeroplasma_99
OTU_3129	7.07E-01	9.90E-01	2.94E-02	2.93	0.29	Erysipelatoclostridium_ramosum_89	Bacteria_Firmicutes_Erysipelotrichia_98_Erysipelotrichales_98_Erysipelatoclostridiaceae_98_Erysipelatoclostridium_98
OTU_637	8.42E-01	5.32E-01	4.32E-01	3.30	1.05	Bacteroides_stercoris_97	Bacteria_Bacteroidetes_Bacteroidia_Bacteroidales_Bacteroidaceae_Bacteroides_78
OTU_7692	7.07E-01	9.85E-01	1.51E-04	3.68	0.80	Roseburia_inulinivorans_94	Bacteria_Firmicutes_Clostridia_Clostridiales_Lachnospiraceae_87_Roseburia_28
OTU_9545	8.85E-01	4.60E-01	1.04E-01	2.35	1.62	Pseudescherichia_vulneris_100	Bacteria_Proteobacteria_Gammaproteobacteria_Enterobacterales_Enterobacteriaceae_99_Escherichia/Shigella_63
OTU_96721	8.06E-01	9.89E-01	2.64E-01	0.98	1.69	Enterocloster_citroniae_97	Bacteria_Firmicutes_Clostridia_Clostridiales_Lachnospiraceae_Enterocloster_96
OTU_1292	8.06E-01	6.04E-01	2.04E-02	3.03	0.39	Lactobacillus_johnsonii_89	Bacteria_Proteobacteria_95_Gammaproteobacteria_95_Enterobacterales_95_Enterobacteriaceae_95_Pseudescherichia_93
OTU_13064	7.73E-01	6.04E-01	6.14E-03	3.96	0.40	Bacteroides_stercoris_96	Bacteria_Bacteroidetes_Bacteroidia_Bacteroidales_Bacteroidaceae_Bacteroides_83
OTU_28622	9.23E-01	6.04E-01	1.77E-02	2.54	1.18	Enterocloster_clostridioformis_96	Bacteria_Firmicutes_Clostridia_Clostridiales_Lachnospiraceae_Enterocloster_80
OTU_2819	7.07E-01	7.07E-01	8.33E-01	2.39	2.18	Clostridium_scindens_97	Bacteria_Firmicutes_Clostridia_Clostridiales_Lachnospiraceae_Clostridium XlVa_90
OTU_446	7.07E-01	6.16E-01	7.60E-01	2.43	1.69	Lacrimispora_saccharolytica_95	Bacteria_Firmicutes_Clostridia_Clostridiales_Lachnospiraceae_Hungatella_24
OTU_6316	7.91E-01	7.97E-01	2.05E-01	2.78	1.70	Clostridium_symbiosum_95	Bacteria_Firmicutes_Clostridia_Clostridiales_Lachnospiraceae_Clostridium XlVa_70
OTU_12648	7.07E-01	9.94E-01	4.42E-03	3.91	0.25	Bacteroides_stercoris_95	Bacteria_Bacteroidetes_Bacteroidia_Bacteroidales_Bacteroidaceae_97_Bacteroides_90
OTU_1789	8.16E-01	7.07E-01	9.06E-01	1.83	1.76	Kineothrix_alysoides_98	Bacteria_Firmicutes_Clostridia_Clostridiales_Lachnospiraceae_Clostridium XlVa_63
OTU_376	7.07E-01	8.00E-01	1.68E-02	4.12	0.33	Bacteroides_stercoris_94	Bacteria_Bacteroidetes_Bacteroidia_99_Bacteroidales_99_Bacteroidaceae_99_Bacteroides_78
OTU_753	7.10E-01	6.04E-01	9.49E-01	0.56	0.58	Kineothrix_alysoides_97	Bacteria_Firmicutes_Clostridia_Clostridiales_Lachnospiraceae_Kineothrix_30
OTU_1444	9.38E-01	6.04E-01	1.44E-01	2.11	1.23	Pseudescherichia_vulneris_97	Bacteria_Proteobacteria_Gammaproteobacteria_Enterobacterales_Enterobacteriaceae_Escherichia/Shigella_82
OTU_4296	7.07E-01	4.60E-01	2.49E-03	3.29	1.16	Roseburia_inulinivorans_95	Bacteria_Firmicutes_Clostridia_Clostridiales_Lachnospiraceae_99_Roseburia_91
OTU_920	9.43E-01	6.42E-01	1.64E-01	0.65	3.40	Coprococcus_comes_97	Bacteria_Firmicutes_95_Clostridia_95_Clostridiales_95_Lachnospiraceae_95_Bariatricus_70
OTU_17942	9.38E-01	6.72E-01	1.70E-01	2.53	1.67	Enterocloster_clostridioformis_96	Bacteria_Firmicutes_Clostridia_Clostridiales_Lachnospiraceae_Ruminococcus2_40
OTU_4433	9.65E-01	7.22E-01	5.75E-01	0.56	0.74	Enterocloster_clostridioformis_96	Bacteria_Firmicutes_Clostridia_Clostridiales_Lachnospiraceae_Lachnospiracea_incertae_sedis_43
OTU_661	7.46E-01	9.62E-01	1.40E-02	0.72	0.10	Faecalibacterium_prausnitzii_92	Bacteria_Firmicutes_Clostridia_Clostridiales_Ruminococcaceae_Faecalibacterium_90
OTU_9262	8.94E-01	6.26E-01	1.30E-02	2.38	0.64	Bifidobacterium_callitrichidarum_92	Bacteria_Actinobacteria_92_Actinobacteria_92_Bifidobacteriales_91_Bifidobacteriaceae_91_Pseudoscardovia_67
OTU_1369	8.59E-01	8.36E-01	2.01E-03	3.45	0.43	Anaerotignum_faecicola_94	Bacteria_Firmicutes_Clostridia_Clostridiales_Lachnospiraceae_98_Anaerotignum_98
OTU_1724	9.65E-01	9.18E-01	2.34E-01	0.93	1.55	Clostridium_methylpentosum_89	Bacteria_Firmicutes_96_Clostridia_96_Clostridiales_96_Ruminococcaceae_84_Clostridium IV_61
OTU_1783	7.07E-01	6.04E-01	6.99E-02	1.97	1.17	Enterococcus_hirae_93	Bacteria_Firmicutes_Bacilli_Lactobacillales_99_Enterococcaceae_94_Enterococcus_52
OTU_202	9.46E-01	7.11E-01	9.96E-02	0.94	0.42	Flintibacter_butyricus_94	Bacteria_Firmicutes_Clostridia_Clostridiales_Ruminococcaceae_97_Intestinimonas_59
OTU_2106	1.00E+00	7.25E-01	8.90E-03	0.71	2.35	Bacteroides_thetaiotaomicron_95	Bacteria_Bacteroidetes_75_Bacteroidia_75_Bacteroidales_75_Bacteroidaceae_75_Mediterranea_51
OTU_253	7.57E-01	NA	1.22E-01	4.92	0.00	Falcatimonas_natans_96	Bacteria_Firmicutes_Clostridia_Clostridiales_Lachnospiraceae_Mediterraneibacter_44
OTU_1599	9.73E-01	6.04E-01	1.75E-02	2.94	0.78	Limosilactobacillus_reuteri_95	Bacteria_Firmicutes_Bacilli_Lactobacillales_99_Lactobacillaceae_76_Limosilactobacillus_73
OTU_17270	7.07E-01	8.63E-01	4.15E-04	0.84	3.16	Mobilitalea_sibirica_89	Bacteria_Bacteroidetes_60_Bacteroidia_60_Bacteroidales_60_Bacteroidaceae_60_Mediterranea_28
OTU_1828	7.07E-01	8.68E-01	7.63E-03	0.83	3.28	Faecalibacterium_prausnitzii_94	Bacteria_Firmicutes_Clostridia_Clostridiales_Ruminococcaceae_Faecalibacterium_54
OTU_226	9.14E-01	7.02E-01	6.15E-01	0.83	0.61	Succinivibrio_dextrinosolvens_93	Bacteria_Proteobacteria_Gammaproteobacteria_Aeromonadales_Succinivibrionaceae_Succinivibrio_100

OTU_24279	9.43E-01	8.42E-01	4.44E-03	3.64	0.95	Bacteroides_xylanisolvens_98	Bacteria_Bacteroidetes_Bacteroidia_Bacteroidales_Bacteroidaceae_Bacteroides_100
OTU_4156	7.12E-01	7.25E-01	1.35E-02	3.33	0.93	Roseburia_inulinivorans_92	Bacteria_Firmicutes_Clostridia_Clostridiales_Lachnospiraceae_82_Ruminococcus2_21
OTU_66368	7.53E-01	6.63E-01	7.81E-01	1.59	1.89	Romboutsia_sedimentorum_89	Bacteria_Proteobacteria_74_Gammaproteobacteria_74_Enterobacterales_74_Enterobacteriaceae_74_Pseudescherichia_66
OTU_765	9.08E-01	7.16E-01	1.36E-01	0.79	2.31	Eubacterium_callanderi_91	Bacteria_Firmicutes_Bacilli_82_Lactobacillales_82_Carnobacteriaceae_44_Isobaculum_33
OTU_8504	7.23E-01	7.16E-01	5.05E-02	3.43	1.12	Bacteroides_timonensis_97	Bacteria_Bacteroidetes_Bacteroidia_Bacteroidales_Bacteroidaceae_Bacteroides_100
OTU_953	9.07E-01	8.65E-01	3.31E-01	2.69	1.86	Roseburia_inulinivorans_97	Bacteria_Firmicutes_Clostridia_Clostridiales_Lachnospiraceae_Roseburia_99
OTU_10545	7.57E-01	8.65E-01	7.71E-01	1.79	2.01	Veillonella_tobetsuensis_96	Bacteria_Firmicutes_Negativicutes_Veillonellales_Veillonellaceae_Veillonella_100
OTU_17243	9.64E-01	9.62E-01	1.57E-02	0.91	2.60	Bacteroides_caccae_96	Bacteria_Bacteroidetes_Bacteroidia_Bacteroidales_Bacteroidaceae_Bacteroides_64
OTU_4577	8.73E-01	9.46E-01	2.12E-01	2.97	1.39	Roseburia_inulinivorans_92	Bacteria_Firmicutes_Clostridia_84_Clostridiales_84_Lachnospiraceae_84_Ruminococcus2_14
OTU_9584	8.44E-01	6.04E-01	3.87E-03	2.67	1.14	Clostridium_carnis_89	Bacteria_Proteobacteria_83_Gammaproteobacteria_83_Enterobacterales_83_Enterobacteriaceae_83_Pseudescherichia_76
OTU_14810	9.87E-01	8.28E-01	1.49E-03	0.86	3.04	Veillonella_tobetsuensis_89	Bacteria_Firmicutes_Clostridia_92_Clostridiales_92_Lachnospiraceae_92_Hungatella_89
OTU_2135	9.84E-01	7.73E-01	6.53E-01	1.32	1.60	Flavonifractor_plautii_93	Bacteria_Firmicutes_Clostridia_Clostridiales_Ruminococcaceae_98_Flavonifractor_97
OTU_3949	8.60E-01	8.65E-01	6.08E-02	1.64	2.66	Enterocloster_citroniae_96	Bacteria_Firmicutes_Clostridia_Clostridiales_Lachnospiraceae_Enterocloster_64
OTU_786	9.19E-01	8.14E-01	4.63E-02	1.12	2.15	Falcatimonas_natans_89	Bacteria_Firmicutes_99_Clostridia_76_Clostridiales_76_Lachnospiraceae_75_Falcatimonas_21
OTU_8588	7.70E-01	8.42E-01	1.36E-03	2.59	0.23	Enterocloster_clostridioformis_99	Bacteria_Firmicutes_Clostridia_98_Clostridiales_98_Lachnospiraceae_98_Enterocloster_90
OTU_1230	9.38E-01	6.99E-01	3.46E-02	0.40	1.22	Enterocloster_citroniae_98	Bacteria_Firmicutes_86_Clostridia_86_Clostridiales_86_Lachnospiraceae_86_Enterocloster_55
OTU_1383	9.59E-01	8.71E-01	8.41E-01	1.59	1.50	Coprococcus_comes_92	Bacteria_Firmicutes_Clostridia_Clostridiales_Lachnospiraceae_77_Coprococcus_37
OTU_4567	8.31E-01	7.41E-01	3.57E-01	1.25	1.74	Pseudescherichia_vulneris_96	Bacteria_Proteobacteria_Gammaproteobacteria_Enterobacterales_Enterobacteriaceae_Escherichia/Shigella_75
OTU_5025	9.63E-01	9.75E-01	2.26E-01	2.46	1.55	Coprococcus_comes_97	Bacteria_Firmicutes_Clostridia_Clostridiales_Lachnospiraceae_Coprococcus_38
OTU_509	7.07E-01	9.85E-01	2.29E-01	0.83	0.07	Bifidobacterium_stercoris_90	Bacteria_Actinobacteria_Actinobacteria_Bifidobacteriales_Bifidobacteriaceae_Pseudoscardovia_41
OTU_60424	9.05E-01	8.78E-01	7.04E-02	1.34	2.09	Pseudescherichia_vulneris_95	Bacteria_Proteobacteria_Gammaproteobacteria_Enterobacterales_Enterobacteriaceae_Pseudescherichia_64
OTU_6656	7.07E-01	6.04E-01	3.57E-01	1.60	2.23	Erysipelatoclostridium_ramosum_97	Bacteria_Firmicutes_Erysipelotrichia_Erysipelotrichales_Erysipelatoclostridiaceae_Erysipelatoclostridium_100
OTU_693	7.46E-01	6.04E-01	2.60E-01	2.49	0.96	Pseudescherichia_vulneris_96	Bacteria_Proteobacteria_Gammaproteobacteria_Enterobacterales_Enterobacteriaceae_Pseudescherichia_55
OTU_8849	7.07E-01	9.72E-01	2.39E-03	2.96	0.65	Shimwellia_blattae_87	Bacteria_Proteobacteria_Gammaproteobacteria_Enterobacterales_Enterobacteriaceae_97_Pseudescherichia_95
OTU_1474	9.56E-01	9.97E-01	5.83E-01	1.19	1.40	Pseudescherichia_vulneris_94	Bacteria_Proteobacteria_Gammaproteobacteria_Enterobacterales_Enterobacteriaceae_Pseudescherichia_64
OTU_2907	8.16E-01	9.70E-01	7.64E-02	1.32	2.50	Enterocloster_clostridioformis_100	Bacteria_Firmicutes_Clostridia_Clostridiales_Lachnospiraceae_Enterocloster_97
OTU_3344	9.06E-01	6.58E-01	6.22E-02	2.55	1.49	Clostridium_perfringens_98	Bacteria_Firmicutes_71_Clostridia_71_Clostridiales_71_Clostridiaceae 1_71_Desnuesiella_31
OTU_3644	9.46E-01	6.04E-01	3.10E-03	2.97	0.87	Pseudescherichia_vulneris_97	Bacteria_Proteobacteria_Gammaproteobacteria_Enterobacterales_Enterobacteriaceae_99_Escherichia/Shigella_66
OTU_398	7.98E-01	6.14E-01	1.65E-01	3.09	0.79	Lacrimispora_xylanolytica_95	Bacteria_Firmicutes_Clostridia_Clostridiales_Lachnospiraceae_Coprococcus_16
OTU_2230	9.31E-01	7.44E-01	9.21E-02	1.12	2.23	Escherichia_coli_96	Bacteria_Proteobacteria_Gammaproteobacteria_Enterobacterales_Enterobacteriaceae_99_Escherichia/Shigella_90
OTU_1479	7.07E-01	6.68E-01	7.46E-02	0.66	0.13	Bifidobacterium_catenulatum_97	Bacteria_Actinobacteria_Actinobacteria_Bifidobacteriales_Bifidobacteriaceae_Bifidobacterium_96
OTU_16233	7.88E-01	7.17E-01	6.42E-01	1.44	1.62	Enterocloster_clostridioformis_97	Bacteria_Firmicutes_Clostridia_Clostridiales_Lachnospiraceae_Clostridium XlVa_58
OTU_4269	7.59E-01	6.16E-01	3.90E-03	1.78	0.59	Escherichia_coli_87	Bacteria_Proteobacteria_52_Gammaproteobacteria_52_Enterobacterales_52_Enterobacteriaceae_52_Pseudescherichia_52
OTU_4686	9.38E-01	8.65E-01	7.11E-01	2.21	1.99	Roseburia_inulinivorans_96	Bacteria_Firmicutes_Clostridia_Clostridiales_Lachnospiraceae_Roseburia_72
OTU_711	7.14E-01	9.46E-01	1.70E-01	0.74	0.23	Phocaeicola_vulgatus_96	Bacteria_Bacteroidetes_Bacteroidia_Bacteroidales_Bacteroidaceae_Bacteroides_49
OTU_100628	9.64E-01	6.04E-01	5.91E-02	2.70	1.02	Clostridium_perfringens_97	Bacteria_Firmicutes_Clostridia_Clostridiales_Clostridiaceae 1_Clostridium sensu stricto_92
OTU_101215	9.48E-01	9.37E-01	1.34E-01	2.67	1.50	Roseburia_inulinivorans_97	Bacteria_Firmicutes_Clostridia_Clostridiales_Lachnospiraceae_Roseburia_83
OTU_1489	9.87E-01	9.69E-01	9.54E-01	2.08	2.05	Pseudescherichia_vulneris_95	Bacteria_Proteobacteria_Gammaproteobacteria_Enterobacterales_Enterobacteriaceae_Pseudescherichia_53
OTU_2366	8.85E-01	6.04E-01	1.38E-01	2.26	0.72	Lactobacillus_johnsonii_97	Bacteria_Firmicutes_Bacilli_Lactobacillales_Lactobacillaceae_Lactobacillus_95
OTU_4708	8.29E-01	6.08E-01	3.37E-01	0.22	0.40	Phocaeicola_coprophilus_97	Bacteria_Bacteroidetes_Bacteroidia_Bacteroidales_Bacteroidaceae_Phocaeicola_100
OTU_47198	7.07E-01	8.23E-01	4.89E-02	2.07	0.28	Veillonella_atypica_96	Bacteria_Firmicutes_Negativicutes_Veillonellales_Veillonellaceae_Veillonella_100
OTU_49472	7.59E-01	9.57E-01	8.45E-02	1.70	2.56	Escherichia_albertii_92	Bacteria_Proteobacteria_97_Gammaproteobacteria_97_Enterobacterales_97_Enterobacteriaceae_97_Pseudescherichia_95
OTU_12390	9.64E-01	8.08E-01	1.87E-01	2.21	1.64	Enterocloster_citroniae_96	Bacteria_Firmicutes_Clostridia_Clostridiales_Lachnospiraceae_Coprococcus_31
OTU_20380	7.11E-01	6.04E-01	3.08E-08	3.59	0.59	Bacteroides_uniformis_97	Bacteria_Bacteroidetes_Bacteroidia_Bacteroidales_Bacteroidaceae_Bacteroides_85
OTU_2327	9.14E-01	9.90E-01	3.64E-01	1.48	1.94	Pseudescherichia_vulneris_88	Bacteria_Proteobacteria_33_Gammaproteobacteria_33_Enterobacterales_33_Enterobacteriaceae_33_Pseudescherichia_33
OTU_2940	9.23E-01	7.65E-01	2.26E-06	3.54	0.46	Bacteroides_uniformis_88	Bacteria_Bacteroidetes_68_Bacteroidia_68_Bacteroidales_68_Bacteroidaceae_65_Mediterranea_45
OTU_13674	7.93E-01	8.33E-01	4.49E-02	1.58	2.59	Bacteroides_thetaiotaomicron_98	Bacteria_Bacteroidetes_Bacteroidia_Bacteroidales_Bacteroidaceae_Bacteroides_100
OTU_1847	7.07E-01	6.04E-01	2.83E-02	1.57	0.49	Collinsella_aerofaciens_97	Bacteria_Actinobacteria_Coriobacteriia_Coriobacteriales_Coriobacteriaceae_Collinsella_98
OTU_2182	9.05E-01	6.63E-01	1.60E-02	3.26	0.84	Bacteroides_caccae_96	Bacteria_Bacteroidetes_Bacteroidia_Bacteroidales_Bacteroidaceae_Bacteroides_84
OTU_35236	7.07E-01	6.04E-01	6.31E-02	0.96	1.79	Enterococcus_faecalis_98	Bacteria_Firmicutes_Bacilli_Lactobacillales_Enterococcaceae_98_Enterococcus_65
OTU_353	7.46E-01	6.04E-01	1.14E-01	2.02	0.15	Muribaculum_intestinale_91	Bacteria_Bacteroidetes_Bacteroidia_Bacteroidales_Muribaculaceae_Duncaniella_79
OTU_4789	8.54E-01	7.02E-01	2.07E-01	0.65	0.27	Phocaeicola_plebeius_94	Bacteria_Bacteroidetes_Bacteroidia_Bacteroidales_Bacteroidaceae_Phocaeicola_100
OTU_67190	9.87E-01	8.51E-01	7.30E-02	1.06	1.57	Pseudescherichia_vulneris_96	Bacteria_Proteobacteria_Gammaproteobacteria_Enterobacterales_Enterobacteriaceae_Escherichia/Shigella_73
OTU_8457	9.45E-01	6.04E-01	4.20E-02	2.09	0.91	Veillonella_atypica_95	Bacteria_Firmicutes_Negativicutes_Veillonellales_Veillonellaceae_Veillonella_100
OTU_901	7.57E-01	9.85E-01	4.20E-02	0.51	2.71	Catellicoccus_marimammalium_86	Bacteria_Firmicutes_Clostridia_88_Clostridiales_88_Ruminococcaceae_88_Faecalibacterium_52
OTU_26319	7.57E-01	9.60E-01	8.70E-01	1.23	1.17	Merdimonas_faecis_95	Bacteria_Firmicutes_Clostridia_Clostridiales_Lachnospiraceae_Clostridium XlVa_76
OTU_3575	9.87E-01	9.13E-01	1.58E-02	3.05	0.95	Clostridium_symbiosum_95	Bacteria_Firmicutes_Clostridia_Clostridiales_Lachnospiraceae_99_Clostridium XlVa_55
OTU_533	7.12E-01	8.59E-01	3.87E-01	0.41	0.11	Fusobacterium_mortiferum_95	Bacteria_Fusobacteria_Fusobacteriia_Fusobacteriales_Fusobacteriaceae_95_Cetobacterium_63
OTU_63215	8.25E-01	6.11E-01	8.21E-01	1.85	1.99	Enterococcus_faecalis_97	Bacteria_Firmicutes_Bacilli_Lactobacillales_Enterococcaceae_84_Enterococcus_50
OTU_1655	8.43E-01	6.14E-01	2.53E-02	2.55	0.32	Lactobacillus_johnsonii_97	Bacteria_Firmicutes_Bacilli_Lactobacillales_Lactobacillaceae_Lactobacillus_97
OTU_1694	7.45E-01	6.04E-01	5.69E-01	1.25	1.72	Dialister_succinatiphilus_94	Bacteria_Firmicutes_Negativicutes_98_Veillonellales_96_Veillonellaceae_96_Dialister_96
OTU_558	7.07E-01	6.04E-01	8.70E-01	1.64	1.96	Fusobacterium_perfoetens_91	Bacteria_Fusobacteria_Fusobacteriia_Fusobacteriales_Fusobacteriaceae_89_Cetobacterium_58
OTU_722	7.10E-01	7.77E-01	1.33E-01	1.12	0.65	Flintibacter_butyricus_95	Bacteria_Firmicutes_Clostridia_Clostridiales_Ruminococcaceae_Intestinimonas_33
OTU_1426	7.07E-01	9.35E-01	5.05E-02	1.60	0.22	Hungatella_effluvii_95	Bacteria_Firmicutes_62_Clostridia_62_Clostridiales_62_Lachnospiraceae_62_Merdimonas_33
OTU_1898	9.60E-01	9.54E-01	9.37E-02	2.15	1.27	Clostridium_symbiosum_94	Bacteria_Firmicutes_Clostridia_Clostridiales_Lachnospiraceae_Clostridium XlVa_55
OTU_333	7.11E-01	6.04E-01	6.29E-01	1.34	2.03	Pseudoflavonifractor_capillosus_97	Bacteria_Firmicutes_Clostridia_Clostridiales_Ruminococcaceae_Pseudoflavonifractor_51
OTU_3558	8.31E-01	6.04E-01	9.27E-04	3.12	0.79	Enterocloster_citroniae_100	Bacteria_Firmicutes_78_Clostridia_78_Clostridiales_78_Lachnospiraceae_78_Enterocloster_52

OTU_4020	9.42E-01	6.04E-01	6.48E-03	3.67	0.53	Bacteroides_xylanisolvens_98	Bacteria_Bacteroidetes_89_Bacteroidia_89_Bacteroidales_89_Bacteroidaceae_89_Bacteroides_81
OTU_4154	8.54E-01	7.36E-01	4.02E-02	2.24	0.73	Clostridium_perfringens_96	Bacteria_Firmicutes_Clostridia_Clostridiales_Clostridiaceae 1_90_Sarcina_39
OTU_4281	7.11E-01	6.04E-01	8.58E-01	1.58	1.50	Pseudescherichia_vulneris_98	Bacteria_Proteobacteria_Gammaproteobacteria_Enterobacterales_Enterobacteriaceae_Pseudescherichia_83
OTU_4488	7.07E-01	9.37E-01	5.02E-03	2.65	0.53	Streptococcus_cristatus_97	Bacteria_Firmicutes_Bacilli_Lactobacillales_Streptococcaceae_97_Streptococcus_87
OTU_481	9.87E-01	8.08E-01	8.70E-01	0.35	0.31	Duncaniella_freteri_86	Bacteria_Bacteroidetes_Bacteroidia_Bacteroidales_Muribaculaceae_71_Muribaculum_69
OTU_1013	7.91E-01	6.04E-01	4.31E-03	1.81	0.32	Bacteroides_fragilis_96	Bacteria_Bacteroidetes_Bacteroidia_Bacteroidales_Bacteroidaceae_Bacteroides_100
OTU_209	7.07E-01	6.14E-01	3.12E-01	4.74	0.26	Ruminococcus_albus_92	Bacteria_Firmicutes_Clostridia_Clostridiales_Ruminococcaceae_Tepidibaculum_43
OTU_2282	7.07E-01	8.36E-01	2.50E-02	1.02	2.51	Falcatimonas_natans_90	Bacteria_Firmicutes_Clostridia_63_Clostridiales_63_Lachnospiraceae_63_Falcatimonas_13
OTU_3439	8.60E-01	6.04E-01	3.34E-01	1.57	1.09	Senegalimassilia_anaerobia_93	Bacteria_Actinobacteria_Coriobacteriia_Coriobacteriales_Coriobacteriaceae_Senegalimassilia_100
OTU_701	7.59E-01	9.54E-01	1.84E-01	1.34	0.54	Pseudescherichia_vulneris_91	Bacteria_Proteobacteria_99_Gammaproteobacteria_99_Enterobacterales_99_Enterobacteriaceae_99_Pseudescherichia_95
OTU_1096	9.31E-01	6.05E-01	1.71E-02	3.64	0.35	Enterococcus_dispar_100	Bacteria_Firmicutes_Bacilli_98_Lactobacillales_98_Carnobacteriaceae_45_Isobaculum_44
OTU_1278	8.08E-01	6.04E-01	1.44E-02	4.02	0.24	Clostridium_cadaveris_97	Bacteria_Proteobacteria_55_Gammaproteobacteria_55_Enterobacterales_55_Enterobacteriaceae_54_Pseudescherichia_54
OTU_1805	9.08E-01	6.76E-01	6.06E-01	1.53	1.82	Clostridium_scindens_96	Bacteria_Firmicutes_Clostridia_Clostridiales_Lachnospiraceae_Clostridium XlVa_96
OTU_21418	7.07E-01	9.43E-01	1.55E-02	2.20	1.14	Merdimonas_faecis_96	Bacteria_Firmicutes_Clostridia_Clostridiales_Lachnospiraceae_Clostridium XlVa_83
OTU_381	9.99E-01	6.50E-01	3.53E-01	0.77	0.49	Flintibacter_butyricus_93	Bacteria_Firmicutes_Clostridia_Clostridiales_Ruminococcaceae_99_Sporobacter_40
OTU_914	7.57E-01	6.04E-01	1.05E-01	2.78	1.03	Clostridium_symbiosum_94	Bacteria_Firmicutes_70_Clostridia_70_Clostridiales_70_Lachnospiraceae_70_Coprococcus_21
OTU_10024	9.73E-01	6.04E-01	1.34E-03	2.73	0.39	Streptococcus_gordonii_98	Bacteria_Firmicutes_Bacilli_Lactobacillales_Streptococcaceae_Streptococcus_100
OTU_10712	9.08E-01	9.15E-01	2.99E-02	3.10	0.37	Veillonella_atypica_97	Bacteria_Firmicutes_Negativicutes_Veillonellales_Veillonellaceae_Veillonella_100
OTU_1177	7.07E-01	8.83E-01	3.76E-02	1.14	0.37	Phocaeicola_vulgatus_99	Bacteria_Bacteroidetes_93_Bacteroidia_93_Bacteroidales_93_Bacteroidaceae_93_Phocaeicola_93
OTU_13335	1.00E+00	7.29E-01	1.74E-01	2.07	1.39	Ruminococcus_torques_97	Bacteria_Firmicutes_Clostridia_Clostridiales_Lachnospiraceae_Ruminococcus2_70
OTU_2451	9.92E-01	6.29E-01	6.47E-01	1.80	2.06	Paraclostridium_benzoelyticum_100	Bacteria_Firmicutes_Clostridia_Clostridiales_Peptostreptococcaceae_Paraclostridium_100
OTU_2899	8.25E-01	8.50E-01	5.59E-01	1.85	1.55	Enterocloster_aldenensis_93	Bacteria_Firmicutes_Clostridia_Clostridiales_Lachnospiraceae_85_Enterocloster_22
OTU_5105	9.78E-01	9.32E-01	2.31E-01	1.66	1.19	Lachnoclostridium_pacaense_96	Bacteria_Firmicutes_Clostridia_Clostridiales_Lachnospiraceae_Clostridium XlVa_32
OTU_69974	7.07E-01	6.04E-01	4.01E-01	1.85	0.93	Pseudescherichia_vulneris_97	Bacteria_Proteobacteria_Gammaproteobacteria_Enterobacterales_Enterobacteriaceae_89_Escherichia/Shigella_68
OTU_836	7.57E-01	6.63E-01	3.50E-02	3.06	0.63	Collinsella_aerofaciens_93	Bacteria_Actinobacteria_98_Coriobacteriia_98_Coriobacteriales_98_Coriobacteriaceae_97_Collinsella_97
OTU_8968	9.04E-01	7.66E-01	9.32E-02	2.62	1.10	Bacteroides_xylanisolvens_97	Bacteria_Bacteroidetes_Bacteroidia_Bacteroidales_Bacteroidaceae_Bacteroides_100
OTU_1329	7.59E-01	6.57E-01	1.75E-01	2.83	1.35	Prevotella_histicola_92	Bacteria_Bacteroidetes_Bacteroidia_Bacteroidales_Prevotellaceae_96_Prevotella_95
OTU_13478	1.00E+00	6.04E-01	9.37E-01	1.46	1.52	Enterocloster_citroniae_97	Bacteria_Firmicutes_Clostridia_Clostridiales_Lachnospiraceae_Enterocloster_85
OTU_2926	8.25E-01	6.04E-01	3.89E-02	1.04	2.39	Phascolarctobacterium_faecium_97	Bacteria_Firmicutes_Negativicutes_99_Acidaminococcales_99_Acidaminococcaceae_99_Phascolarctobacterium_99
OTU_824	7.10E-01	6.04E-01	2.77E-01	0.65	0.34	Collinsella_aerofaciens_96	Bacteria_Actinobacteria_Coriobacteriia_Coriobacteriales_Coriobacteriaceae_Collinsella_99
OTU_15927	9.08E-01	9.69E-01	4.02E-01	1.53	1.20	Enterocloster_citroniae_97	Bacteria_Firmicutes_Clostridia_Clostridiales_Lachnospiraceae_Enterocloster_99
OTU_3397	9.31E-01	7.01E-01	5.26E-01	1.16	1.39	Enterococcus_faecalis_92	Bacteria_Firmicutes_65_Bacilli_65_Lactobacillales_65_Carnobacteriaceae_40_Catellicoccus_30
OTU_6904	7.07E-01	7.99E-01	3.57E-01	0.47	1.33	Pseudescherichia_vulneris_97	Bacteria_Proteobacteria_Gammaproteobacteria_Enterobacterales_Enterobacteriaceae_Escherichia/Shigella_61
OTU_7370	8.82E-01	9.25E-01	1.36E-01	1.32	2.06	Pseudoflavonifractor_capillosus_97	Bacteria_Firmicutes_Clostridia_Clostridiales_Ruminococcaceae_Flavonifractor_56
OTU_8578	7.81E-01	9.25E-01	3.40E-02	1.19	2.38	Paraclostridium_benzoelyticum_96	Bacteria_Firmicutes_Clostridia_Clostridiales_Peptostreptococcaceae_99_Paraclostridium_96
OTU_1361	7.46E-01	6.14E-01	2.42E-02	3.06	0.44	Fusobacterium_equinum_91	Bacteria_Fusobacteria_Fusobacteriia_Fusobacteriales_Fusobacteriaceae_90_Cetobacterium_51
OTU_14617	7.91E-01	9.46E-01	4.02E-02	0.07	0.33	Haemophilus_pittmaniae_99	Bacteria_Proteobacteria_Gammaproteobacteria_Pasteurellales_Pasteurellaceae_Haemophilus_94
OTU_14689	9.67E-01	9.74E-01	4.58E-01	1.84	2.22	Roseburia_inulinivorans_96	Bacteria_Firmicutes_Clostridia_Clostridiales_Lachnospiraceae_99_Roseburia_65
OTU_2521	9.04E-01	6.04E-01	7.88E-02	2.22	0.57	Allobaculum_stercoricanis_87	Bacteria_Firmicutes_Erysipelotrichia_Erysipelotrichales_Erysipelotrichaceae_Faecalibaculum_24
OTU_4059	7.10E-01	9.79E-01	5.82E-01	0.45	0.59	Kineothrix_alysoides_95	Bacteria_Firmicutes_Clostridia_Clostridiales_Lachnospiraceae_Clostridium XlVa_50
OTU_4213	7.46E-01	9.81E-01	4.03E-03	2.94	0.71	Clostridium_perfringens_93	Bacteria_Proteobacteria_44_Gammaproteobacteria_44_Enterobacterales_44_Enterobacteriaceae_44_Pseudescherichia_44
OTU_10439	7.46E-01	8.22E-01	9.27E-04	2.86	0.22	Streptococcus_mitis_98	Bacteria_Firmicutes_Bacilli_Lactobacillales_Streptococcaceae_Streptococcus_100
OTU_1173	7.07E-01	9.06E-01	9.14E-02	1.76	0.35	Streptococcus_gordonii_91	Bacteria_Firmicutes_Bacilli_93_Lactobacillales_84_Carnobacteriaceae_29_Isobaculum_12
OTU_1191	7.91E-01	7.60E-01	3.27E-01	1.20	1.80	Paraclostridium_benzoelyticum_93	Bacteria_Firmicutes_Clostridia_Clostridiales_Peptostreptococcaceae_92_Paraclostridium_90
OTU_1196	7.12E-01	9.24E-01	3.48E-02	3.40	0.30	Bacteroides_xylanisolvens_99	Bacteria_Bacteroidetes_89_Bacteroidia_89_Bacteroidales_89_Bacteroidaceae_88_Mediterranea_46
OTU_291	7.07E-01	9.57E-01	2.64E-01	0.16	1.55	Novosphingobium_fontis_99	Bacteria_Proteobacteria_Alphaproteobacteria_Sphingomonadales_Erythrobacteraceae_94_Altererythrobacter_46
OTU_3545	8.42E-01	6.04E-01	3.69E-03	2.54	0.85	Clostridium_tarantellae_93	Bacteria_Firmicutes_Clostridia_Clostridiales_Lachnospiraceae_75_Mobilitalea_22
OTU_4546	8.25E-01	6.04E-01	6.41E-02	0.84	2.50	Vibrio_porteresiae_86	Bacteria_Proteobacteria_70_Gammaproteobacteria_70_Enterobacterales_70_Enterobacteriaceae_69_Pseudescherichia_66
OTU_6828	7.73E-01	9.20E-01	4.28E-06	2.83	0.56	Escherichia_coli_90	Bacteria_Proteobacteria_87_Gammaproteobacteria_87_Enterobacterales_87_Enterobacteriaceae_87_Pseudescherichia_87
OTU_79063	7.07E-01	8.02E-01	2.87E-02	4.00	0.30	Bacteroides_stercoris_97	Bacteria_Bacteroidetes_Bacteroidia_Bacteroidales_Bacteroidaceae_Bacteroides_100
OTU_13638	8.96E-01	7.01E-01	1.87E-02	2.81	0.81	Sutterella_massiliensis_94	Bacteria_Proteobacteria_Gammaproteobacteria_60_Enterobacterales_59_Enterobacteriaceae_58_Pseudescherichia_55
OTU_3956	9.84E-01	7.73E-01	4.26E-01	1.68	2.09	Roseburia_inulinivorans_92	Bacteria_Firmicutes_Clostridia_95_Clostridiales_95_Lachnospiraceae_95_Roseburia_30
OTU_322	7.57E-01	9.13E-01	7.47E-01	0.76	0.63	Parasutterella_secunda_94	Bacteria_Proteobacteria_Betaproteobacteria_Burkholderiales_Sutterellaceae_98_Duodenibacillus_88
OTU_4326	9.38E-01	6.04E-01	7.31E-01	1.48	1.36	Enterococcus_faecalis_90	Bacteria_Proteobacteria_86_Gammaproteobacteria_86_Enterobacterales_86_Enterobacteriaceae_86_Pseudescherichia_84
OTU_690	7.11E-01	6.04E-01	2.98E-01	1.12	0.52	Fusobacterium_mortiferum_94	Bacteria_Fusobacteria_Fusobacteriia_Fusobacteriales_Fusobacteriaceae_99_Cetobacterium_62
OTU_814	7.07E-01	6.04E-01	6.60E-02	3.52	0.20	Bacteroides_xylanisolvens_86	Bacteria_Fusobacteria_80_Fusobacteriia_80_Fusobacteriales_80_Fusobacteriaceae_80_Cetobacterium_53
OTU_9524	9.08E-01	6.32E-01	6.49E-01	1.37	1.63	Xenorhabdus_ehlersii_88	Bacteria_Proteobacteria_99_Gammaproteobacteria_99_Enterobacterales_98_Enterobacteriaceae_98_Pseudescherichia_96
OTU_10026	7.93E-01	6.04E-01	4.56E-01	1.01	1.61	Bacteroides_faecichinchillae_95	Bacteria_Bacteroidetes_Bacteroidia_Bacteroidales_Bacteroidaceae_98_Bacteroides_98
OTU_1024	7.12E-01	9.25E-01	4.77E-01	0.38	0.25	Faecalibacterium_prausnitzii_95	Bacteria_Firmicutes_Clostridia_Clostridiales_Ruminococcaceae_Faecalibacterium_98
OTU_107455	7.46E-01	8.42E-01	9.84E-01	0.22	0.22	Veillonella_tobetsuensis_97	Bacteria_Firmicutes_Negativicutes_Veillonellales_Veillonellaceae_Veillonella_100
OTU_2125	7.73E-01	6.04E-01	8.24E-01	1.89	2.05	Erysipelatoclostridium_ramosum_96	Bacteria_Firmicutes_Erysipelotrichia_Erysipelotrichales_Erysipelatoclostridiaceae_Erysipelatoclostridium_100
OTU_2490	7.59E-01	6.04E-01	1.11E-01	2.30	1.18	Faecalibacterium_prausnitzii_96	Bacteria_Firmicutes_Clostridia_Clostridiales_Ruminococcaceae_Faecalibacterium_95
OTU_3740	8.49E-01	6.63E-01	5.10E-01	1.18	1.44	Flavonifractor_plautii_95	Bacteria_Firmicutes_Clostridia_Clostridiales_Ruminococcaceae_95_Flavonifractor_93
OTU_41212	7.07E-01	6.04E-01	3.21E-02	3.59	0.42	Bacteroides_finegoldii_97	Bacteria_Bacteroidetes_Bacteroidia_Bacteroidales_Bacteroidaceae_Bacteroides_100
OTU_978	7.07E-01	7.10E-01	1.22E-01	2.62	0.87	Kosakonia_sacchari_91	Bacteria_Proteobacteria_96_Gammaproteobacteria_96_Enterobacterales_95_Enterobacteriaceae_95_Pseudescherichia_92
OTU_2048	7.10E-01	6.04E-01	3.00E-02	3.23	0.69	Bacteroides_clarus_98	Bacteria_Bacteroidetes_Bacteroidia_Bacteroidales_Bacteroidaceae_Bacteroides_100
OTU_27719	7.59E-01	9.42E-01	1.19E-02	1.78	0.22	Bifidobacterium_stercoris_98	Bacteria_Actinobacteria_Actinobacteria_Bifidobacteriales_Bifidobacteriaceae_Bifidobacterium_88

OTU_30480	9.48E-01	9.19E-01	5.15E-03	0.74	2.56	Veillonella_dispar_96	Bacteria_Firmicutes_Negativicutes_Veillonellales_Veillonellaceae_Veillonella_98
OTU_3118	9.78E-01	8.28E-01	6.00E-01	1.75	2.05	Coprococcus_comes_96	Bacteria_Firmicutes_Clostridia_Clostridiales_Lachnospiraceae_Bariatricus_60
OTU_3627	8.31E-01	9.18E-01	5.67E-01	1.52	1.81	Escherichia_albertii_89	Bacteria_Proteobacteria_80_Gammaproteobacteria_80_Enterobacterales_80_Enterobacteriaceae_78_Pseudescherichia_78
OTU_553	7.57E-01	7.50E-01	8.00E-01	1.32	1.66	Anaerotignum_aminivorans_97	Bacteria_Firmicutes_Clostridia_Clostridiales_Lachnospiraceae_Anaerotignum_100
OTU_8125	9.14E-01	6.04E-01	4.41E-02	1.29	2.20	Enterocloster_clostridioformis_96	Bacteria_Firmicutes_Clostridia_Clostridiales_Lachnospiraceae_Enterocloster_76
OTU_9554	9.67E-01	6.83E-01	7.21E-01	1.90	1.74	Enterocloster_clostridioformis_89	Bacteria_Proteobacteria_58_Gammaproteobacteria_58_Enterobacterales_58_Enterobacteriaceae_58_Pseudescherichia_57
OTU_1003	8.67E-01	6.05E-01	1.78E-01	1.11	0.49	Catenibacterium_mitsuokai_96	Bacteria_Firmicutes_90_Erysipelotrichia_90_Erysipelotrichales_90_Erysipelotrichaceae_90_Catenibacterium_73
OTU_2058	9.99E-01	6.04E-01	1.75E-01	1.73	0.97	Peptoniphilus_grossensis_98	Bacteria_Firmicutes_Clostridia_90_Clostridiales_90_Peptoniphilaceae_89_Peptoniphilus_88
OTU_4335	7.59E-01	6.04E-01	9.60E-01	1.71	1.67	Pseudoflavonifractor_capillosus_98	Bacteria_Firmicutes_Clostridia_Clostridiales_Ruminococcaceae_Pseudoflavonifractor_100
OTU_1271	7.57E-01	6.04E-01	1.18E-01	4.30	0.36	Vallitalea_pronyensis_86	Bacteria_Firmicutes_98_Clostridia_81_Clostridiales_81_Tissierellaceae_18_Gudongella_12
OTU_1482	7.07E-01	6.04E-01	1.81E-01	2.86	0.62	Streptococcus_porcorum_88	Bacteria_Proteobacteria_88_Gammaproteobacteria_88_Enterobacterales_87_Enterobacteriaceae_87_Pseudescherichia_84
OTU_1511	7.07E-01	7.42E-01	7.67E-02	0.47	1.20	Veillonella_atypica_98	Bacteria_Firmicutes_Negativicutes_85_Veillonellales_79_Veillonellaceae_79_Veillonella_75
OTU_31940	7.07E-01	6.66E-01	7.26E-02	1.68	0.71	Veillonella_atypica_94	Bacteria_Firmicutes_Negativicutes_98_Veillonellales_97_Veillonellaceae_97_Veillonella_97
OTU_3425	9.60E-01	7.99E-01	5.04E-02	2.20	0.88	Enterocloster_clostridioformis_97	Bacteria_Firmicutes_Clostridia_Clostridiales_Lachnospiraceae_Enterocloster_98
OTU_3881	9.37E-01	6.04E-01	3.04E-01	1.30	1.84	Escherichia_albertii_90	Bacteria_Firmicutes_54_Clostridia_54_Clostridiales_54_Lachnospiraceae_54_Hungatella_49
OTU_400	8.25E-01	6.04E-01	4.71E-02	3.74	0.42	Intestinimonas_butyriciproducens_93	Bacteria_Firmicutes_Clostridia_Clostridiales_Ruminococcaceae_Lawsonibacter_16
OTU_81311	7.07E-01	9.58E-01	9.22E-02	3.70	0.39	Bacteroides_stercoris_97	Bacteria_Bacteroidetes_Bacteroidia_Bacteroidales_Bacteroidaceae_Bacteroides_100
OTU_1109	8.77E-01	7.51E-01	2.96E-01	1.94	1.16	Shigella_boydii_90	Bacteria_Proteobacteria_Gammaproteobacteria_Enterobacterales_Enterobacteriaceae_Pseudescherichia_89
OTU_1491	7.07E-01	6.14E-01	5.24E-02	3.51	0.16	Bacteroides_stercoris_99	Bacteria_Bacteroidetes_86_Bacteroidia_86_Bacteroidales_86_Bacteroidaceae_85_Mediterranea_65
OTU_39297	8.16E-01	8.23E-01	6.66E-04	3.70	0.09	Bacteroides_uniformis_96	Bacteria_Bacteroidetes_Bacteroidia_Bacteroidales_Bacteroidaceae_Bacteroides_99
OTU_50204	7.46E-01	9.74E-01	7.31E-03	0.78	1.96	Shigella_dysenteriae_95	Bacteria_Proteobacteria_Gammaproteobacteria_Enterobacterales_Enterobacteriaceae_Escherichia/Shigella_60
OTU_5948	7.59E-01	6.14E-01	2.42E-01	2.08	1.24	Phocaeicola_vulgatus_97	Bacteria_Bacteroidetes_Bacteroidia_Bacteroidales_Bacteroidaceae_Phocaeicola_58
OTU_621	9.92E-01	6.04E-01	1.05E-01	0.37	0.07	Collinsella_aerofaciens_100	Bacteria_Actinobacteria_92_Coriobacteriia_92_Coriobacteriales_91_Coriobacteriaceae_90_Collinsella_87
OTU_622	9.26E-01	6.04E-01	1.35E-02	1.16	0.16	Megasphaera_elsdenii_98	Bacteria_Firmicutes_Negativicutes_69_Veillonellales_69_Veillonellaceae_69_Megasphaera_68
OTU_10292	9.95E-01	8.73E-01	5.50E-01	1.63	1.90	Coprococcus_comes_92	Bacteria_Firmicutes_96_Clostridia_96_Clostridiales_96_Lachnospiraceae_96_Bariatricus_59
OTU_11432	8.06E-01	7.99E-01	9.22E-02	0.85	2.28	Dickeya_chrysanthemi_89	Bacteria_Proteobacteria_87_Gammaproteobacteria_87_Enterobacterales_87_Enterobacteriaceae_86_Pseudescherichia_83
OTU_114383	7.07E-01	NA	1.05E-01	4.24	0.00	Bacteroides_stercoris_97	Bacteria_Bacteroidetes_Bacteroidia_Bacteroidales_Bacteroidaceae_Bacteroides_100
OTU_13300	1.00E+00	7.88E-01	9.53E-02	1.01	2.11	Nitrincola_lacisaponensis_87	Bacteria_Proteobacteria_93_Gammaproteobacteria_93_Enterobacterales_92_Enterobacteriaceae_89_Pseudescherichia_88
OTU_15690	9.64E-01	9.46E-01	8.37E-02	1.30	1.92	Hungatella_effluvii_97	Bacteria_Firmicutes_Clostridia_Clostridiales_Lachnospiraceae_Hungatella_72
OTU_1707	8.97E-01	6.04E-01	9.72E-01	1.41	1.43	Ruminococcus_gnavus_94	Bacteria_Firmicutes_Clostridia_Clostridiales_Lachnospiraceae_94_Dorea_89
OTU_1768	9.78E-01	6.04E-01	6.30E-01	1.46	1.81	Acidaminococcus_fermentans_98	Bacteria_Firmicutes_Negativicutes_93_Acidaminococcales_93_Acidaminococcaceae_93_Acidaminococcus_92
OTU_273	7.91E-01	6.04E-01	1.87E-01	1.20	0.21	Kineothrix_alysoides_90	Bacteria_Firmicutes_Clostridia_Clostridiales_Lachnospiraceae_Eisenbergiella_17
OTU_36382	7.59E-01	8.28E-01	1.92E-03	2.51	0.72	Clostridium_perfringens_93	Bacteria_Firmicutes_Clostridia_Clostridiales_Lachnospiraceae_59_Mobilitalea_9
OTU_4319	7.07E-01	6.72E-01	3.66E-05	2.45	0.58	Ruminococcus_torques_94	Bacteria_Firmicutes_Clostridia_Clostridiales_Lachnospiraceae_98_Coprococcus_50
OTU_48983	8.85E-01	8.02E-01	1.67E-03	3.65	0.16	Bacteroides_xylanisolvens_98	Bacteria_Bacteroidetes_Bacteroidia_Bacteroidales_Bacteroidaceae_Bacteroides_100
OTU_5172	9.84E-01	9.75E-01	4.10E-01	1.56	2.04	Coprococcus_comes_95	Bacteria_Firmicutes_Clostridia_Clostridiales_Lachnospiraceae_Coprococcus_59
OTU_6070	1.00E+00	9.42E-01	4.58E-01	1.59	1.28	Hungatella_hathewayi_92	Bacteria_Firmicutes_Clostridia_75_Clostridiales_75_Lachnospiraceae_75_Falcatimonas_43
OTU_1038	9.06E-01	6.04E-01	8.30E-01	0.52	0.58	Phocaeicola_coprophilus_99	Bacteria_Bacteroidetes_98_Bacteroidia_98_Bacteroidales_98_Bacteroidaceae_98_Phocaeicola_89
OTU_10453	8.82E-01	9.73E-01	9.96E-03	2.29	0.94	Escherichia_albertii_90	Bacteria_Proteobacteria_76_Gammaproteobacteria_76_Enterobacterales_75_Enterobacteriaceae_75_Pseudescherichia_74
OTU_11625	1.00E+00	8.45E-01	3.34E-01	1.55	1.23	Bacteroides_fragilis_96	Bacteria_Bacteroidetes_Bacteroidia_Bacteroidales_Bacteroidaceae_Bacteroides_100
OTU_14889	8.06E-01	8.14E-01	1.16E-02	0.65	2.14	Bacteroides_finegoldii_95	Bacteria_Bacteroidetes_Bacteroidia_Bacteroidales_Bacteroidaceae_Bacteroides_83
OTU_1513	7.93E-01	6.04E-01	5.24E-01	0.56	0.77	Bacteroides_kribbi_97	Bacteria_Bacteroidetes_Bacteroidia_Bacteroidales_Bacteroidaceae_Bacteroides_89
OTU_3038	7.97E-01	8.83E-01	4.09E-01	1.35	1.72	Enterocloster_aldenensis_95	Bacteria_Firmicutes_Clostridia_Clostridiales_Lachnospiraceae_Enterocloster_50
OTU_3567	7.57E-01	6.76E-01	2.08E-01	2.21	1.48	Roseburia_inulinivorans_96	Bacteria_Firmicutes_Clostridia_Clostridiales_Lachnospiraceae_Roseburia_56
OTU_816	9.99E-01	6.10E-01	1.95E-02	2.85	0.58	Clostridium_symbiosum_97	Bacteria_Firmicutes_97_Clostridia_97_Clostridiales_97_Lachnospiraceae_96_Clostridium XlVa_34
OTU_8612	9.43E-01	6.04E-01	1.87E-01	1.57	1.10	Faecalicatena_orotica_96	Bacteria_Firmicutes_Clostridia_Clostridiales_Lachnospiraceae_Clostridium XlVa_89
OTU_1076	9.80E-01	8.14E-01	6.59E-03	1.64	0.68	Enterococcus_pseudoavium_90	Bacteria_Firmicutes_Clostridia_93_Clostridiales_93_Lachnospiraceae_93_Dorea_85
OTU_11077	7.07E-01	6.30E-01	5.05E-02	1.26	2.08	Enterocloster_aldenensis_94	Bacteria_Firmicutes_Clostridia_Clostridiales_Lachnospiraceae_Enterocloster_51
OTU_1554	1.00E+00	9.54E-01	8.51E-01	1.36	1.28	Dorea_longicatena_92	Bacteria_Firmicutes_85_Clostridia_85_Clostridiales_85_Lachnospiraceae_85_Dorea_81
OTU_2399	8.82E-01	9.14E-01	3.74E-02	1.85	0.94	Ruminococcus_bromii_98	Bacteria_Firmicutes_Clostridia_Clostridiales_Ruminococcaceae_Ruminococcus_88
OTU_65108	8.70E-01	6.57E-01	8.70E-04	0.55	2.94	Mobilitalea_sibirica_88	Bacteria_Bacteroidetes_68_Bacteroidia_68_Bacteroidales_68_Bacteroidaceae_68_Bacteroides_55
OTU_930	9.72E-01	6.05E-01	4.59E-02	0.78	0.25	Sutterella_wadsworthensis_99	Bacteria_Proteobacteria_98_Betaproteobacteria_98_Burkholderiales_98_Sutterellaceae_98_Sutterella_95
OTU_9405	9.38E-01	6.04E-01	1.32E-02	2.52	0.77	Pseudescherichia_vulneris_97	Bacteria_Proteobacteria_Gammaproteobacteria_Enterobacterales_Enterobacteriaceae_Escherichia/Shigella_89
OTU_25004	7.07E-01	9.94E-01	1.03E-01	1.02	1.75	Enterococcus_faecalis_98	Bacteria_Firmicutes_Bacilli_Lactobacillales_99_Enterococcaceae_93_Enterococcus_72
OTU_45442	8.85E-01	9.46E-01	1.99E-01	1.35	1.87	Shigella_dysenteriae_97	Bacteria_Proteobacteria_Gammaproteobacteria_Enterobacterales_Enterobacteriaceae_Escherichia/Shigella_67
OTU_525	7.24E-01	7.67E-01	1.34E-01	2.97	0.14	Lacrimispora_saccharolytica_95	Bacteria_Firmicutes_Clostridia_Clostridiales_Lachnospiraceae_Clostridium XlVa_32
OTU_646	7.14E-01	6.04E-01	6.92E-02	3.10	0.33	Clostridium_cadaveris_96	Bacteria_Firmicutes_Clostridia_Clostridiales_Clostridiaceae 1_Anaerobacter_41
OTU_7015	9.98E-01	7.24E-01	9.96E-01	0.51	0.51	Faecalibacterium_prausnitzii_92	Bacteria_Firmicutes_Clostridia_Clostridiales_Ruminococcaceae_86_Faecalibacterium_72
OTU_92099	9.31E-01	7.20E-01	4.53E-02	2.76	0.62	Bacteroides_xylanisolvens_97	Bacteria_Bacteroidetes_Bacteroidia_Bacteroidales_Bacteroidaceae_Bacteroides_100
OTU_1916	8.60E-01	9.44E-01	4.32E-03	0.75	2.13	Enterococcus_faecalis_91	Bacteria_Firmicutes_75_Bacilli_74_Lactobacillales_73_Carnobacteriaceae_51_Catellicoccus_29
OTU_2838	9.83E-01	6.04E-01	3.92E-02	2.49	1.26	Bacteroides_xylanisolvens_96	Bacteria_Firmicutes_53_Bacilli_53_Lactobacillales_49_Carnobacteriaceae_43_Catellicoccus_39
OTU_3033	9.75E-01	7.02E-01	7.31E-02	1.79	1.04	Enterococcus_dispar_100	Bacteria_Firmicutes_Bacilli_93_Lactobacillales_91_Carnobacteriaceae_58_Isobaculum_57
OTU_3833	9.20E-01	9.06E-01	1.53E-01	1.15	2.36	Mobilitalea_sibirica_89	Bacteria_Firmicutes_73_Clostridia_73_Clostridiales_73_Lachnospiraceae_73_Bariatricus_65
OTU_4248	9.87E-01	8.46E-01	4.12E-02	2.13	1.18	Roseburia_inulinivorans_93	Bacteria_Firmicutes_99_Clostridia_99_Clostridiales_99_Lachnospiraceae_99_Roseburia_74
OTU_4625	8.24E-01	9.35E-01	5.61E-01	0.24	0.38	Phocaeicola_coprocola_96	Bacteria_Bacteroidetes_Bacteroidia_Bacteroidales_Bacteroidaceae_Phocaeicola_100
OTU_1891	9.00E-01	7.66E-01	9.49E-03	3.29	0.16	Bifidobacterium_breve_89	Bacteria_Actinobacteria_96_Actinobacteria_96_Bifidobacteriales_96_Bifidobacteriaceae_96_Pseudoscardovia_41
OTU_3775	7.07E-01	8.22E-01	1.52E-02	2.20	0.26	Parabacteroides_merdae_97	Bacteria_Bacteroidetes_Bacteroidia_Bacteroidales_Porphyromonadaceae_99_Parabacteroides_99

OTU_4570	9.62E-01	6.10E-01	5.27E-02	2.37	1.08	Roseburia_inulinivorans_96	Bacteria_Firmicutes_Clostridia_Clostridiales_Lachnospiraceae_Bariatricus_53
OTU_665	7.62E-01	8.68E-01	1.57E-01	0.57	0.19	Collinsella_aerofaciens_99	Bacteria_Actinobacteria_99_Coriobacteriia_99_Coriobacteriales_99_Coriobacteriaceae_99_Collinsella_98
OTU_7369	8.33E-01	7.30E-01	1.71E-02	2.15	1.03	Clostridium_perfringens_94	Bacteria_Firmicutes_Clostridia_Clostridiales_Clostridiaceae 1_78_Desnuesiella_40
OTU_26296	9.84E-01	7.77E-01	5.09E-01	1.49	1.13	Bacteroides_thetaiotaomicron_96	Bacteria_Bacteroidetes_Bacteroidia_Bacteroidales_Bacteroidaceae_Bacteroides_94
OTU_3315	7.80E-01	9.25E-01	1.29E-01	2.16	1.37	Clostridium_perfringens_96	Bacteria_Firmicutes_Clostridia_Clostridiales_Clostridiaceae 1_88_Sarcina_49
OTU_3886	7.94E-01	9.41E-01	8.41E-02	0.94	2.16	Pseudescherichia_vulneris_94	Bacteria_Proteobacteria_98_Gammaproteobacteria_98_Enterobacterales_98_Enterobacteriaceae_97_Pseudescherichia_52
OTU_4792	9.87E-01	6.65E-01	8.23E-01	1.53	1.42	Hungatella_hathewayi_100	Bacteria_Firmicutes_Clostridia_Clostridiales_Lachnospiraceae_Hungatella_100
OTU_1577	8.33E-01	6.04E-01	1.27E-01	2.02	0.87	Veillonella_parvula_98	Bacteria_Firmicutes_Negativicutes_Veillonellales_Veillonellaceae_Veillonella_100
OTU_24213	8.73E-01	9.46E-01	8.26E-03	0.82	2.71	Bacteroides_kribbi_97	Bacteria_Bacteroidetes_Bacteroidia_Bacteroidales_Bacteroidaceae_Bacteroides_100
OTU_2728	7.57E-01	6.05E-01	1.30E-01	1.36	0.75	Blautia_hydrogenotrophica_92	Bacteria_Firmicutes_Clostridia_78_Clostridiales_78_Lachnospiraceae_78_Lactonifactor_13
OTU_3216	8.74E-01	7.40E-01	2.01E-03	2.19	0.71	Clostridium_carnis_90	Bacteria_Proteobacteria_85_Gammaproteobacteria_85_Enterobacterales_83_Enterobacteriaceae_83_Pseudescherichia_77
OTU_3520	7.07E-01	6.14E-01	1.95E-04	2.43	0.37	Bifidobacterium_dentium_87	Bacteria_Proteobacteria_89_Gammaproteobacteria_89_Enterobacterales_89_Enterobacteriaceae_89_Pseudescherichia_81
OTU_40735	9.43E-01	9.32E-01	6.25E-04	3.14	0.39	Bacteroides_faecis_97	Bacteria_Bacteroidetes_Bacteroidia_Bacteroidales_Bacteroidaceae_Bacteroides_100
OTU_4501	8.43E-01	6.72E-01	1.83E-01	1.43	2.11	Blautia_faecicola_95	Bacteria_Firmicutes_Clostridia_Clostridiales_Lachnospiraceae_Kineothrix_23
OTU_492	NA	8.46E-01	1.75E-01	0.00	2.48	Hydrogenophaga_laconesensis_100	Bacteria_Proteobacteria_Betaproteobacteria_Burkholderiales_Comamonadaceae_Hydrogenophaga_100
OTU_572	NA	9.25E-01	2.21E-01	0.00	3.55	Desulfovibrio_butyratiphilus_97	Bacteria_Proteobacteria_Deltaproteobacteria_Desulfovibrionales_Desulfovibrionaceae_Desulfovibrio_99
OTU_712	7.07E-01	6.72E-01	1.47E-01	0.56	0.21	Slackia_isoflavoniconvertens_91	Bacteria_Actinobacteria_Coriobacteriia_97_Eggerthellales_97_Eggerthellaceae_97_Slackia_97
OTU_11056	9.45E-01	6.05E-01	3.82E-02	2.39	0.82	Pseudescherichia_vulneris_94	Bacteria_Proteobacteria_Gammaproteobacteria_83_Enterobacterales_83_Enterobacteriaceae_83_Pseudescherichia_80
OTU_1179	9.08E-01	8.95E-01	8.33E-01	0.31	0.28	Faecalibacterium_prausnitzii_94	Bacteria_Firmicutes_Clostridia_Clostridiales_Ruminococcaceae_91_Faecalibacterium_86
OTU_13185	7.07E-01	7.78E-01	3.74E-02	3.48	0.38	Bacteroides_stercoris_97	Bacteria_Bacteroidetes_Bacteroidia_Bacteroidales_Bacteroidaceae_Bacteroides_100
OTU_1417	7.07E-01	8.46E-01	9.29E-02	0.58	0.16	Phocaeicola_vulgatus_94	Bacteria_Bacteroidetes_Bacteroidia_Bacteroidales_Bacteroidaceae_89_Phocaeicola_89
OTU_1527	9.65E-01	9.35E-01	4.27E-01	1.97	1.60	Roseburia_inulinivorans_93	Bacteria_Firmicutes_Clostridia_Clostridiales_Lachnospiraceae_59_Roseburia_46
OTU_16829	7.22E-01	6.72E-01	1.71E-02	2.00	0.50	Collinsella_aerofaciens_91	Bacteria_Actinobacteria_68_Coriobacteriia_68_Coriobacteriales_68_Coriobacteriaceae_62_Collinsella_61
OTU_1713	7.91E-01	9.09E-01	1.25E-01	1.62	2.30	Bacteroides_thetaiotaomicron_95	Bacteria_Bacteroidetes_Bacteroidia_Bacteroidales_Bacteroidaceae_Bacteroides_99
OTU_20100	7.57E-01	9.89E-01	2.06E-01	2.01	1.21	Veillonella_parvula_98	Bacteria_Firmicutes_Negativicutes_Veillonellales_Veillonellaceae_Veillonella_100
OTU_3617	7.80E-01	7.14E-01	1.76E-03	2.69	0.75	Clostridium_tarantellae_92	Bacteria_Firmicutes_Clostridia_Clostridiales_Lachnospiraceae_79_Ruminococcus2_22
OTU_397	NA	6.72E-01	2.01E-01	0.00	3.18	Methylotenera_mobilis_96	Bacteria_Proteobacteria_Betaproteobacteria_Nitrosomonadales_Methylophilaceae_Methylotenera_86
OTU_1050	9.96E-01	8.75E-01	2.13E-01	0.34	0.18	Bifidobacterium_callitrichidarum_92	Bacteria_Actinobacteria_91_Actinobacteria_91_Bifidobacteriales_91_Bifidobacteriaceae_91_Bifidobacterium_55
OTU_64838	8.57E-01	7.38E-01	7.86E-02	0.94	1.45	Enterococcus_faecalis_98	Bacteria_Firmicutes_Bacilli_Lactobacillales_Enterococcaceae_99_Enterococcus_78
OTU_8337	8.60E-01	9.75E-01	5.52E-01	1.46	1.74	Anaerotaenia_torta_89	Bacteria_Firmicutes_55_Clostridia_55_Clostridiales_55_Lachnospiraceae_55_Murimonas_12
OTU_1023	7.07E-01	8.18E-01	6.71E-01	0.09	0.16	Bifidobacterium_stercoris_97	Bacteria_Actinobacteria_81_Actinobacteria_81_Bifidobacteriales_81_Bifidobacteriaceae_81_Pseudoscardovia_42
OTU_13998	7.91E-01	6.35E-01	3.58E-01	1.12	1.44	Pseudescherichia_vulneris_96	Bacteria_Proteobacteria_Gammaproteobacteria_Enterobacterales_Enterobacteriaceae_Escherichia/Shigella_60
OTU_1711	7.07E-01	6.83E-01	1.71E-02	2.38	0.93	Coprococcus_comes_95	Bacteria_Firmicutes_Clostridia_Clostridiales_Lachnospiraceae_Coprococcus_47
OTU_1773	7.07E-01	8.45E-01	9.22E-02	1.06	2.14	Kosakonia_sacchari_88	Bacteria_Firmicutes_61_Clostridia_61_Clostridiales_61_Ruminococcaceae_61_Faecalibacterium_37
OTU_19875	7.07E-01	6.04E-01	3.89E-01	1.18	1.51	Enterococcus_faecalis_98	Bacteria_Firmicutes_Bacilli_Lactobacillales_Enterococcaceae_93_Enterococcus_58
OTU_2066	9.99E-01	6.04E-01	1.50E-02	0.99	0.17	Enterocloster_citroniae_98	Bacteria_Firmicutes_74_Clostridia_74_Clostridiales_74_Lachnospiraceae_74_Enterocloster_36
OTU_28616	9.39E-01	8.14E-01	5.32E-04	0.69	2.53	Bacteroides_thetaiotaomicron_100	Bacteria_Bacteroidetes_98_Bacteroidia_98_Bacteroidales_98_Bacteroidaceae_98_Bacteroides_98
OTU_2953	8.31E-01	6.04E-01	1.50E-03	1.71	0.24	Bifidobacterium_stercoris_95	Bacteria_Actinobacteria_92_Actinobacteria_92_Bifidobacteriales_92_Bifidobacteriaceae_92_Bifidobacterium_59
OTU_30180	8.68E-01	7.22E-01	1.12E-01	0.81	1.37	Veillonella_dispar_96	Bacteria_Firmicutes_Negativicutes_Veillonellales_70_Veillonellaceae_70_Veillonella_70
OTU_30592	9.99E-01	9.41E-01	4.46E-03	0.72	0.07	Bacteroides_caccae_96	Bacteria_Bacteroidetes_Bacteroidia_Bacteroidales_Bacteroidaceae_Bacteroides_97
OTU_565	7.07E-01	6.04E-01	3.06E-02	1.61	0.16	Clostridium_tarantellae_91	Bacteria_Firmicutes_Clostridia_77_Clostridiales_77_Clostridiaceae 1_77_Sarcina_31
OTU_885	9.37E-01	8.51E-01	1.07E-01	2.79	0.31	Kineothrix_alysoides_97	Bacteria_Firmicutes_Clostridia_Clostridiales_Lachnospiraceae_Kineothrix_58
OTU_1955	7.57E-01	6.04E-01	8.23E-01	1.63	1.78	Erysipelatoclostridium_ramosum_97	Bacteria_Firmicutes_Erysipelotrichia_Erysipelotrichales_Erysipelatoclostridiaceae_Erysipelatoclostridium_100
OTU_70254	7.85E-01	6.63E-01	3.74E-03	2.92	0.51	Roseburia_inulinivorans_93	Bacteria_Firmicutes_Clostridia_Clostridiales_Lachnospiraceae_77_Coprococcus_14
OTU_1031	9.04E-01	7.36E-01	6.33E-01	1.04	0.88	Flavonifractor_plautii_97	Bacteria_Firmicutes_Clostridia_Clostridiales_Ruminococcaceae_Flavonifractor_100
OTU_1152	7.85E-01	8.37E-01	8.51E-01	1.17	1.25	Blautia_coccoides_96	Bacteria_Firmicutes_Clostridia_Clostridiales_Lachnospiraceae_Blautia_100
OTU_1463	7.57E-01	6.73E-01	1.43E-01	1.23	2.25	Erysipelatoclostridium_ramosum_98	Bacteria_Firmicutes_Erysipelotrichia_94_Erysipelotrichales_94_Erysipelatoclostridiaceae_92_Erysipelatoclostridium_92
OTU_14693	9.14E-01	9.27E-01	8.15E-02	1.01	1.97	Anaerotaenia_torta_89	Bacteria_Proteobacteria_81_Gammaproteobacteria_81_Enterobacterales_81_Enterobacteriaceae_80_Pseudescherichia_75
OTU_19704	7.50E-01	6.04E-01	1.62E-02	1.95	0.70	Veillonella_parvula_98	Bacteria_Firmicutes_Negativicutes_Veillonellales_Veillonellaceae_Veillonella_100
OTU_2661	7.57E-01	9.72E-01	6.15E-01	1.92	1.64	Bacteroides_xylanisolvens_96	Bacteria_Bacteroidetes_Bacteroidia_Bacteroidales_Bacteroidaceae_Bacteroides_100
OTU_4148	1.00E+00	6.04E-01	1.60E-02	2.47	0.84	Escherichia_albertii_88	Bacteria_Proteobacteria_60_Gammaproteobacteria_60_Enterobacterales_60_Enterobacteriaceae_60_Pseudescherichia_60
OTU_453	7.10E-01	6.04E-01	1.15E-01	0.65	0.09	Megasphaera_elsdenii_98	Bacteria_Firmicutes_79_Negativicutes_79_Veillonellales_79_Veillonellaceae_79_Megasphaera_76
OTU_1163	7.16E-01	9.73E-01	1.50E-02	1.82	0.37	Escherichia_coli_88	Bacteria_Proteobacteria_64_Gammaproteobacteria_64_Enterobacterales_64_Enterobacteriaceae_64_Pseudescherichia_64
OTU_1279	7.57E-01	6.04E-01	1.40E-02	2.85	0.12	Lactobacillus_johnsonii_95	Bacteria_Firmicutes_Bacilli_Lactobacillales_96_Lactobacillaceae_62_Lactobacillus_60
OTU_19891	8.57E-01	9.69E-01	4.69E-01	1.76	1.40	Enterococcus_thailandicus_97	Bacteria_Firmicutes_Bacilli_Lactobacillales_Enterococcaceae_85_Enterococcus_47
OTU_31631	8.85E-01	9.61E-01	5.31E-01	1.22	0.91	Coprococcus_comes_96	Bacteria_Firmicutes_Clostridia_Clostridiales_Lachnospiraceae_Coprococcus_38
OTU_32402	7.07E-01	6.04E-01	5.10E-01	1.13	1.38	Enterococcus_faecalis_98	Bacteria_Firmicutes_Bacilli_Lactobacillales_Enterococcaceae_96_Enterococcus_76
OTU_4748	9.87E-01	6.04E-01	1.43E-01	0.91	1.44	Enterococcus_faecalis_93	Bacteria_Firmicutes_83_Bacilli_83_Lactobacillales_81_Carnobacteriaceae_50_Catellicoccus_42
OTU_55027	9.34E-01	8.51E-01	4.07E-03	0.48	1.75	Pseudoflavonifractor_capillosus_97	Bacteria_Firmicutes_Clostridia_Clostridiales_Ruminococcaceae_Flavonifractor_50
OTU_907	9.06E-01	9.94E-01	9.57E-02	0.63	0.15	Breznakia_blatticola_88	Bacteria_Firmicutes_Clostridia_84_Clostridiales_84_Lachnospiraceae_84_Merdimonas_22
OTU_2511	7.07E-01	9.35E-01	4.02E-04	1.50	0.19	Bifidobacterium_catenulatum_97	Bacteria_Actinobacteria_Actinobacteria_Bifidobacteriales_Bifidobacteriaceae_Bifidobacterium_99
OTU_2965	9.09E-01	6.04E-01	5.34E-01	1.83	1.38	Acidaminococcus_fermentans_93	Bacteria_Firmicutes_Negativicutes_91_Acidaminococcales_91_Acidaminococcaceae_91_Acidaminococcus_91
OTU_541	9.99E-01	6.47E-01	2.17E-01	0.62	0.27	Streptococcus_thermophilus_100	Bacteria_Firmicutes_88_Bacilli_82_Lactobacillales_80_Streptococcaceae_51_Streptococcus_47
OTU_760	8.47E-01	9.83E-01	3.84E-02	0.24	2.31	Clostridium_combesii_99	Bacteria_Firmicutes_Negativicutes_67_Acidaminococcales_67_Acidaminococcaceae_67_Phascolarctobacterium_67
OTU_9099	7.57E-01	8.18E-01	9.38E-06	3.33	0.05	Parabacteroides_distasonis_94	Bacteria_Bacteroidetes_Bacteroidia_Bacteroidales_Porphyromonadaceae_84_Parabacteroides_84
OTU_1978	7.45E-01	6.57E-01	8.80E-02	1.25	0.54	Kineothrix_alysoides_94	Bacteria_Firmicutes_Clostridia_Clostridiales_Lachnospiraceae_Clostridium XlVa_56
OTU_3905	9.87E-01	7.51E-01	1.68E-01	1.23	1.73	Acetivibrio_ethanolgignens_93	Bacteria_Firmicutes_Clostridia_96_Clostridiales_96_Lachnospiraceae_96_Mediterraneibacter_8

OTU_4094	7.07E-01	7.01E-01	5.52E-01	1.63	1.32	Pseudescherichia_vulneris_93	Bacteria_Proteobacteria_Gammaproteobacteria_Enterobacterales_Enterobacteriaceae_99_Escherichia/Shigella_85
OTU_4231	9.08E-01	8.83E-01	4.12E-01	1.98	1.54	Hungatella_effluvii_100	Bacteria_Firmicutes_92_Clostridia_92_Clostridiales_92_Lachnospiraceae_92_Hungatella_91
OTU_5331	8.74E-01	6.04E-01	4.10E-01	1.10	0.80	Ruminococcus_torques_99	Bacteria_Firmicutes_Clostridia_Clostridiales_Lachnospiraceae_Ruminococcus2_74
OTU_625	7.07E-01	9.69E-01	1.00E+00	0.22	0.22	Collinsella_aerofaciens_98	Bacteria_Actinobacteria_85_Coriobacteriia_85_Coriobacteriales_85_Coriobacteriaceae_85_Collinsella_81
OTU_1129	7.46E-01	6.04E-01	5.06E-01	1.83	1.28	Pseudescherichia_vulneris_97	Bacteria_Proteobacteria_Gammaproteobacteria_Enterobacterales_Enterobacteriaceae_Escherichia/Shigella_82
OTU_1881	9.04E-01	6.14E-01	4.39E-03	1.86	0.18	Bifidobacterium_stercoris_97	Bacteria_Actinobacteria_Actinobacteria_Bifidobacteriales_Bifidobacteriaceae_Bifidobacterium_90
OTU_3097	7.93E-01	6.04E-01	8.81E-01	1.27	1.20	Lachnoclostridium_pacaense_95	Bacteria_Firmicutes_Clostridia_Clostridiales_Lachnospiraceae_Kineothrix_24
OTU_3113	7.57E-01	6.04E-01	5.57E-01	1.70	1.22	Collinsella_aerofaciens_98	Bacteria_Actinobacteria_81_Coriobacteriia_81_Coriobacteriales_81_Coriobacteriaceae_78_Collinsella_75
OTU_330	NA	6.04E-01	1.42E-01	0.00	0.92	Pseudomonas_paralactis_100	Bacteria_Proteobacteria_Gammaproteobacteria_Pseudomonadales_Pseudomonadaceae_Pseudomonas_100
OTU_45606	7.07E-01	9.18E-01	2.01E-03	2.44	0.42	Streptococcus_mitis_97	Bacteria_Firmicutes_Bacilli_Lactobacillales_Streptococcaceae_98_Streptococcus_97
OTU_49224	7.07E-01	9.73E-01	7.98E-01	1.39	1.22	Clostridium_symbiosum_95	Bacteria_Firmicutes_Clostridia_Clostridiales_Lachnospiraceae_Clostridium XlVa_33
OTU_751	8.06E-01	6.04E-01	5.34E-02	2.16	0.36	Clostridium_viride_97	Bacteria_Firmicutes_Clostridia_Clostridiales_Ruminococcaceae_Intestinimonas_72
OTU_1198	8.60E-01	7.32E-01	1.77E-01	2.14	1.36	Roseburia_inulinivorans_96	Bacteria_Firmicutes_Clostridia_Clostridiales_Lachnospiraceae_Roseburia_98
OTU_1672	7.11E-01	6.43E-01	9.63E-01	0.44	0.42	Haemophilus_parainfluenzae_95	Bacteria_Proteobacteria_Gammaproteobacteria_Pasteurellales_79_Pasteurellaceae_79_Haemophilus_63
OTU_21725	7.82E-01	6.04E-01	6.39E-03	2.10	0.21	Phocaeicola_vulgatus_95	Bacteria_Bacteroidetes_Bacteroidia_Bacteroidales_Bacteroidaceae_Phocaeicola_99
OTU_2777	7.84E-01	8.14E-01	9.79E-03	1.81	0.44	Parabacteroides_merdae_97	Bacteria_Bacteroidetes_Bacteroidia_Bacteroidales_Porphyromonadaceae_Parabacteroides_100
OTU_2834	7.74E-01	6.04E-01	7.13E-01	1.44	1.60	Hungatella_effluvii_95	Bacteria_Firmicutes_99_Clostridia_99_Clostridiales_99_Lachnospiraceae_99_Hungatella_99
OTU_29575	7.91E-01	6.04E-01	1.10E-01	0.99	1.61	Enterococcus_faecalis_98	Bacteria_Firmicutes_Bacilli_Lactobacillales_Enterococcaceae_95_Enterococcus_75
OTU_618	7.07E-01	9.79E-01	5.01E-03	2.53	0.23	Lactonifactor_longoviformis_100	Bacteria_Firmicutes_Clostridia_Clostridiales_Lachnospiraceae_Lactonifactor_92
OTU_64472	9.61E-01	9.41E-01	4.65E-03	1.65	0.38	Bacteroides_faecis_95	Bacteria_Bacteroidetes_Bacteroidia_Bacteroidales_Bacteroidaceae_Bacteroides_80
OTU_1017	7.07E-01	6.04E-01	4.17E-02	0.68	2.47	Phascolarctobacterium_faecium_98	Bacteria_Firmicutes_Negativicutes_96_Acidaminococcales_96_Acidaminococcaceae_96_Phascolarctobacterium_95
OTU_1165	9.83E-01	7.34E-01	3.49E-02	2.14	0.64	Kineothrix_alysoides_94	Bacteria_Firmicutes_Clostridia_Clostridiales_Lachnospiraceae_Clostridium XlVa_68
OTU_11759	8.25E-01	9.75E-01	3.86E-01	1.31	1.64	Pseudescherichia_vulneris_98	Bacteria_Proteobacteria_95_Gammaproteobacteria_95_Enterobacterales_94_Enterobacteriaceae_94_Pseudescherichia_74
OTU_22967	7.07E-01	9.75E-01	2.21E-01	1.32	1.92	Veillonella_dispar_96	Bacteria_Firmicutes_Negativicutes_Veillonellales_Veillonellaceae_Veillonella_100
OTU_27734	9.29E-01	6.66E-01	5.67E-02	2.36	0.84	Clostridium_symbiosum_95	Bacteria_Firmicutes_Clostridia_Clostridiales_Lachnospiraceae_Clostridium XlVa_62
OTU_63037	7.07E-01	6.04E-01	5.04E-01	1.01	1.28	Enterococcus_faecalis_98	Bacteria_Firmicutes_Bacilli_Lactobacillales_Enterococcaceae_98_Enterococcus_83
OTU_11250	7.45E-01	9.91E-01	7.55E-02	0.83	1.80	Mixta_theicola_88	Bacteria_Proteobacteria_57_Gammaproteobacteria_57_Enterobacterales_57_Enterobacteriaceae_57_Pseudescherichia_57
OTU_1718	9.59E-01	6.04E-01	8.21E-01	1.77	1.59	Acidaminococcus_fermentans_93	Bacteria_Firmicutes_Negativicutes_Acidaminococcales_Acidaminococcaceae_Acidaminococcus_100
OTU_2033	7.75E-01	6.04E-01	3.93E-04	2.81	0.36	Fusicatenibacter_saccharivorans_92	Bacteria_Firmicutes_Clostridia_Clostridiales_Lachnospiraceae_38_Anaerotaenia_15
OTU_3486	7.07E-01	6.50E-01	3.46E-03	2.00	0.39	Enterococcus_dispar_98	Bacteria_Firmicutes_96_Bacilli_95_Lactobacillales_86_Carnobacteriaceae_65_Isobaculum_54
OTU_79355	7.07E-01	9.90E-01	3.84E-02	3.39	0.23	Bacteroides_stercoris_97	Bacteria_Bacteroidetes_Bacteroidia_Bacteroidales_Bacteroidaceae_Bacteroides_100
OTU_794	7.07E-01	6.04E-01	9.65E-02	0.77	2.03	Erysipelatoclostridium_ramosum_91	Bacteria_Firmicutes_Erysipelotrichia_99_Erysipelotrichales_99_Erysipelatoclostridiaceae_99_Erysipelatoclostridium_99
OTU_808	8.05E-01	9.32E-01	6.85E-02	0.06	0.54	Limosilactobacillus_mucosae_96	Bacteria_Firmicutes_Bacilli_Lactobacillales_Lactobacillaceae_Limosilactobacillus_58
OTU_97727	1.00E+00	9.74E-01	2.99E-03	0.29	3.03	Veillonella_dispar_96	Bacteria_Firmicutes_Negativicutes_Veillonellales_Veillonellaceae_Veillonella_100
OTU_1022	7.10E-01	9.57E-01	3.27E-02	1.79	0.18	Collinsella_aerofaciens_99	Bacteria_Actinobacteria_98_Coriobacteriia_98_Coriobacteriales_98_Coriobacteriaceae_98_Collinsella_96
OTU_1197	7.07E-01	6.04E-01	7.64E-02	3.24	0.19	Streptococcus_salivarius_90	Bacteria_Firmicutes_99_Clostridia_86_Clostridiales_86_Lachnospiraceae_86_Roseburia_60
OTU_13151	7.14E-01	6.04E-01	2.67E-04	2.05	0.25	Serratia_marcescens_91	Bacteria_Proteobacteria_Gammaproteobacteria_Enterobacterales_Enterobacteriaceae_Pseudescherichia_89
OTU_1409	8.57E-01	9.62E-01	6.88E-02	0.77	2.25	Paraclostridium_benzoelyticum_92	Bacteria_Firmicutes_Clostridia_Clostridiales_Peptostreptococcaceae_56_Paraclostridium_54
OTU_245	9.99E-01	9.34E-01	9.82E-03	0.64	0.15	Eubacterium_ruminantium_98	Bacteria_Firmicutes_Clostridia_Clostridiales_Lachnospiraceae_Lachnospiracea_incertae_sedis_85
OTU_2544	7.59E-01	6.04E-01	3.23E-02	3.26	0.12	Clostridium_cadaveris_97	Bacteria_Firmicutes_Clostridia_Clostridiales_Clostridiaceae 1_Clostridium sensu stricto_56
OTU_31022	7.81E-01	9.81E-01	5.16E-02	0.76	1.59	Enterococcus_faecalis_98	Bacteria_Firmicutes_Bacilli_Lactobacillales_95_Enterococcaceae_87_Enterococcus_78
OTU_32971	7.07E-01	9.41E-01	2.47E-02	1.60	0.19	Bifidobacterium_stercoris_98	Bacteria_Actinobacteria_Actinobacteria_Bifidobacteriales_Bifidobacteriaceae_Bifidobacterium_99
OTU_3978	7.57E-01	6.06E-01	3.51E-02	2.22	0.73	Pseudescherichia_vulneris_96	Bacteria_Proteobacteria_93_Gammaproteobacteria_91_Enterobacterales_91_Enterobacteriaceae_91_Pseudescherichia_79
OTU_441	NA	8.79E-01	2.10E-01	0.00	2.83	Azospira_restricta_99	Bacteria_Proteobacteria_Betaproteobacteria_Rhodocyclales_Rhodocyclaceae_Azospira_99
OTU_4713	7.57E-01	8.65E-01	1.21E-02	2.90	0.29	Bacteroides_xylanisolvens_97	Bacteria_Bacteroidetes_89_Bacteroidia_88_Bacteroidales_88_Bacteroidaceae_85_Mediterranea_63
OTU_52131	9.45E-01	8.68E-01	2.42E-04	0.67	2.05	Veillonella_dispar_96	Bacteria_Firmicutes_Negativicutes_99_Veillonellales_96_Veillonellaceae_96_Veillonella_95
OTU_629	7.46E-01	7.11E-01	1.00E-01	1.57	0.37	Solobacterium_moorei_98	Bacteria_Firmicutes_Erysipelotrichia_Erysipelotrichales_Erysipelotrichaceae_Solobacterium_100
OTU_11917	7.62E-01	8.65E-01	3.37E-01	1.11	1.40	Clostridium_symbiosum_96	Bacteria_Firmicutes_Clostridia_Clostridiales_Lachnospiraceae_Clostridium XlVa_24
OTU_295	9.92E-01	9.46E-01	7.85E-01	0.63	0.52	Bacteroides_pectinophilus_100	Bacteria_Firmicutes_Clostridia_Clostridiales_Lachnospiraceae_99_Clostridium XlVa_64
OTU_3032	8.97E-01	7.58E-01	8.94E-02	1.04	2.16	Bariatricus_massiliensis_97	Bacteria_Firmicutes_Clostridia_Clostridiales_Lachnospiraceae_Bariatricus_74
OTU_4037	9.60E-01	6.63E-01	3.50E-01	1.10	1.62	Paraclostridium_benzoelyticum_92	Bacteria_Firmicutes_Clostridia_Clostridiales_Lachnospiraceae_58_Coprococcus_23
OTU_4316	7.81E-01	8.73E-01	6.66E-04	2.28	0.37	Streptococcus_rubneri_89	Bacteria_Firmicutes_Clostridia_89_Clostridiales_89_Lachnospiraceae_89_Enterocloster_79
OTU_479	7.11E-01	7.31E-01	1.43E-01	3.66	0.03	Prevotella_copri_94	Bacteria_Bacteroidetes_Bacteroidia_Bacteroidales_Prevotellaceae_Prevotella_100
OTU_997	7.46E-01	9.62E-01	6.62E-01	1.69	1.22	Succinivibrio_dextrinosolvens_94	Bacteria_Proteobacteria_Gammaproteobacteria_Aeromonadales_Succinivibrionaceae_Succinivibrio_100
OTU_1147	7.07E-01	6.04E-01	8.72E-02	0.36	2.68	Clostridium_combesii_95	Bacteria_Firmicutes_Clostridia_Clostridiales_Peptostreptococcaceae_56_Peptacetobacter_29
OTU_14133	7.88E-01	9.82E-01	1.16E-05	2.67	0.31	Bacteroides_ovatus_96	Bacteria_Bacteroidetes_Bacteroidia_Bacteroidales_Bacteroidaceae_Bacteroides_94
OTU_1440	8.97E-01	9.17E-01	1.68E-02	2.82	0.49	Pseudescherichia_vulneris_93	Bacteria_Proteobacteria_Gammaproteobacteria_95_Enterobacterales_95_Enterobacteriaceae_95_Pseudescherichia_94
OTU_2166	9.05E-01	9.94E-01	6.83E-03	2.61	0.58	Clostridium_symbiosum_95	Bacteria_Firmicutes_Clostridia_Clostridiales_Lachnospiraceae_Clostridium XlVa_59
OTU_4372	9.45E-01	7.22E-01	8.57E-02	1.69	0.89	Pseudescherichia_vulneris_97	Bacteria_Proteobacteria_Gammaproteobacteria_Enterobacterales_Enterobacteriaceae_98_Pseudescherichia_84
OTU_4832	NA	6.50E-01	5.85E-02	0.00	2.26	Methylotenera_mobilis_93	Bacteria_Proteobacteria_Gammaproteobacteria_54_Enterobacterales_52_Enterobacteriaceae_52_Pseudescherichia_45
OTU_7639	7.46E-01	6.72E-01	3.19E-01	1.28	1.69	Paraclostridium_benzoelyticum_96	Bacteria_Firmicutes_Clostridia_Clostridiales_Peptostreptococcaceae_98_Paraclostridium_98
OTU_830	7.07E-01	6.04E-01	9.37E-02	0.41	1.96	Enterococcus_faecalis_90	Bacteria_Firmicutes_99_Clostridia_66_Clostridiales_66_Eubacteriaceae_64_Pseudoramibacter_46
OTU_1137	7.07E-01	8.46E-01	3.00E-01	1.51	0.97	Blautia_coccoides_98	Bacteria_Firmicutes_Clostridia_Clostridiales_Lachnospiraceae_Blautia_99
OTU_1298	8.74E-01	8.59E-01	9.25E-01	1.79	1.88	Bacteroides_timonensis_95	Bacteria_Bacteroidetes_Bacteroidia_Bacteroidales_Bacteroidaceae_Bacteroides_100
OTU_13968	9.67E-01	8.72E-01	2.09E-02	1.54	0.88	Pseudescherichia_vulneris_91	Bacteria_Proteobacteria_97_Gammaproteobacteria_97_Enterobacterales_96_Enterobacteriaceae_96_Pseudescherichia_96
OTU_1745	9.99E-01	8.28E-01	1.38E-01	1.20	1.90	Enterocloster_citroniae_100	Bacteria_Firmicutes_Clostridia_Clostridiales_Lachnospiraceae_Enterocloster_100
OTU_36277	8.06E-01	9.90E-01	3.15E-02	1.62	0.95	Pseudescherichia_vulneris_96	Bacteria_Proteobacteria_Gammaproteobacteria_Enterobacterales_Enterobacteriaceae_Escherichia/Shigella_91

OTU_3922	9.08E-01	6.39E-01	7.65E-02	0.66	1.62	Eubacterium_callanderi_92	Bacteria_Firmicutes_87_Clostridia_87_Clostridiales_87_Eubacteriaceae_87_Pseudoramibacter_86
OTU_4668	7.07E-01	6.04E-01	7.84E-01	1.68	1.35	Kluyvera_cryocrescens_99	Bacteria_Proteobacteria_Gammaproteobacteria_Enterobacterales_Enterobacteriaceae_99_Kluyvera_65
OTU_580	9.99E-01	NA	3.11E-02	2.61	0.00	Duncaniella_freteri_87	Bacteria_Bacteroidetes_Bacteroidia_Bacteroidales_Muribaculaceae_96_Muribaculum_94
OTU_6138	7.22E-01	8.36E-01	3.39E-01	1.29	1.79	Bacteroides_thetaiotaomicron_99	Bacteria_Bacteroidetes_87_Bacteroidia_87_Bacteroidales_87_Bacteroidaceae_86_Mediterranea_67
OTU_7124	9.38E-01	8.65E-01	8.86E-01	1.41	1.48	Pseudescherichia_vulneris_94	Bacteria_Proteobacteria_Gammaproteobacteria_Enterobacterales_Enterobacteriaceae_99_Pseudescherichia_68
OTU_7586	7.07E-01	6.04E-01	2.05E-02	1.00	1.93	Enterococcus_dispar_96	Bacteria_Firmicutes_46_Bacilli_46_Lactobacillales_43_Carnobacteriaceae_36_Isobaculum_29
OTU_9840	9.35E-01	6.06E-01	1.75E-02	2.63	0.66	Clostridium_perfringens_93	Bacteria_Firmicutes_Clostridia_Clostridiales_Lachnospiraceae_78_Clostridium XlVa_32
OTU_10571	7.59E-01	6.66E-01	1.04E-02	1.84	0.68	Veillonella_atypica_95	Bacteria_Firmicutes_Negativicutes_Veillonellales_Veillonellaceae_Veillonella_100
OTU_13489	9.14E-01	5.62E-01	1.65E-01	1.07	0.65	Collinsella_aerofaciens_94	Bacteria_Actinobacteria_98_Coriobacteriia_98_Coriobacteriales_98_Coriobacteriaceae_98_Collinsella_97
OTU_1433	1.00E+00	9.89E-01	7.43E-02	2.02	0.92	Kineothrix_alysoides_94	Bacteria_Firmicutes_Clostridia_Clostridiales_Lachnospiraceae_Clostridium XlVa_58
OTU_3125	9.57E-01	6.06E-01	3.33E-01	1.10	1.74	Acidaminococcus_fermentans_95	Bacteria_Firmicutes_Negativicutes_Acidaminococcales_Acidaminococcaceae_Acidaminococcus_100
OTU_63523	8.57E-01	9.24E-01	2.47E-02	0.70	1.48	Enterococcus_faecalis_97	Bacteria_Firmicutes_Bacilli_Lactobacillales_Enterococcaceae_87_Enterococcus_65
OTU_7431	1.00E+00	7.31E-01	7.89E-01	1.50	1.63	Faecalimonas_umbilicata_96	Bacteria_Firmicutes_Clostridia_Clostridiales_Lachnospiraceae_Coprococcus_66
OTU_8346	7.07E-01	9.46E-01	1.10E-01	0.99	2.02	Pseudescherichia_vulneris_92	Bacteria_Proteobacteria_97_Gammaproteobacteria_97_Enterobacterales_97_Enterobacteriaceae_97_Pseudescherichia_89
OTU_8913	9.04E-01	9.25E-01	8.33E-01	1.45	1.36	Bacteroides_thetaiotaomicron_99	Bacteria_Bacteroidetes_95_Bacteroidia_95_Bacteroidales_95_Bacteroidaceae_95_Bacteroides_95
OTU_12840	9.14E-01	8.18E-01	1.92E-02	1.84	0.84	Enterocloster_clostridioformis_96	Bacteria_Firmicutes_Clostridia_Clostridiales_Lachnospiraceae_Coprococcus_49
OTU_1958	9.62E-01	6.72E-01	9.27E-03	0.41	2.49	Clostridium_cocleatum_90	Bacteria_Firmicutes_Erysipelotrichia_86_Erysipelotrichales_86_Erysipelatoclostridiaceae_84_Erysipelatoclostridium_84
OTU_2134	9.38E-01	9.66E-01	1.12E-02	0.21	1.05	Phocaeicola_coprophilus_88	Bacteria_Proteobacteria_48_Gammaproteobacteria_48_Enterobacterales_48_Enterobacteriaceae_48_Pseudescherichia_48
OTU_2189	9.06E-01	9.88E-01	7.99E-03	2.32	0.21	Pseudescherichia_vulneris_99	Bacteria_Proteobacteria_83_Gammaproteobacteria_83_Enterobacterales_83_Enterobacteriaceae_83_Pseudescherichia_80
OTU_33599	7.07E-01	6.76E-01	2.35E-01	1.08	1.60	Enterococcus_faecalis_98	Bacteria_Firmicutes_Bacilli_Lactobacillales_Enterococcaceae_94_Enterococcus_88
OTU_3624	7.07E-01	6.04E-01	1.74E-02	2.12	0.99	Eisenbergiella_tayi_95	Bacteria_Firmicutes_Clostridia_Clostridiales_Lachnospiraceae_95_Eisenbergiella_94
OTU_64910	8.53E-01	7.02E-01	2.49E-01	0.98	1.37	Pseudescherichia_vulneris_96	Bacteria_Proteobacteria_Gammaproteobacteria_Enterobacterales_Enterobacteriaceae_Escherichia/Shigella_89
OTU_8069	7.07E-01	8.51E-01	7.19E-02	1.76	0.84	Veillonella_atypica_96	Bacteria_Firmicutes_Negativicutes_99_Veillonellales_98_Veillonellaceae_98_Veillonella_98
OTU_1074	9.73E-01	6.04E-01	2.63E-02	0.39	1.23	Escherichia_coli_88	Bacteria_Proteobacteria_78_Gammaproteobacteria_78_Enterobacterales_78_Enterobacteriaceae_78_Pseudescherichia_78
OTU_17520	7.07E-01	6.65E-01	1.42E-01	0.48	1.14	Phocaeicola_coprophilus_94	Bacteria_Bacteroidetes_Bacteroidia_Bacteroidales_Bacteroidaceae_Bacteroides_48
OTU_1870	7.91E-01	7.40E-01	4.03E-03	3.12	0.17	Clostridium_perfringens_96	Bacteria_Firmicutes_88_Clostridia_88_Clostridiales_88_Clostridiaceae 1_87_Anaerobacter_26
OTU_23253	8.20E-01	8.08E-01	1.13E-01	1.13	1.86	Veillonella_dispar_100	Bacteria_Firmicutes_81_Negativicutes_81_Veillonellales_57_Veillonellaceae_57_Veillonella_54
OTU_51095	7.85E-01	6.39E-01	8.36E-03	2.20	0.21	Streptococcus_mitis_98	Bacteria_Firmicutes_Bacilli_Lactobacillales_Streptococcaceae_Streptococcus_100
OTU_80179	9.32E-01	9.25E-01	6.14E-01	1.21	1.42	Pseudescherichia_vulneris_95	Bacteria_Proteobacteria_Gammaproteobacteria_Enterobacterales_Enterobacteriaceae_Pseudescherichia_76
OTU_9355	7.07E-01	8.50E-01	9.99E-03	2.03	0.31	Bacteroides_kribbi_94	Bacteria_Bacteroidetes_Bacteroidia_Bacteroidales_Bacteroidaceae_Bacteroides_52
OTU_1886	9.98E-01	6.04E-01	5.32E-03	2.25	0.37	Enterococcus_hirae_94	Bacteria_Firmicutes_Bacilli_93_Lactobacillales_93_Enterococcaceae_65_Melissococcus_38
OTU_2297	7.07E-01	8.68E-01	7.32E-01	0.92	0.78	Pseudescherichia_vulneris_97	Bacteria_Proteobacteria_Gammaproteobacteria_Enterobacterales_Enterobacteriaceae_Escherichia/Shigella_80
OTU_3285	7.07E-01	9.84E-01	3.49E-02	1.95	0.63	Veillonella_atypica_96	Bacteria_Firmicutes_Negativicutes_95_Veillonellales_94_Veillonellaceae_94_Veillonella_94
OTU_3408	1.00E+00	7.88E-01	2.32E-02	1.44	0.21	Bacteroides_uniformis_96	Bacteria_Bacteroidetes_Bacteroidia_Bacteroidales_Bacteroidaceae_99_Bacteroides_98
OTU_3726	9.64E-01	7.40E-01	3.43E-01	1.58	1.09	Enterococcus_hirae_92	Bacteria_Firmicutes_Bacilli_68_Lactobacillales_67_Carnobacteriaceae_34_Catellicoccus_15
OTU_3827	9.46E-01	9.83E-01	4.84E-02	0.94	1.65	Enterococcus_hirae_95	Bacteria_Firmicutes_Bacilli_83_Lactobacillales_83_Enterococcaceae_61_Vagococcus_40
OTU_4487	9.37E-01	6.66E-01	5.30E-01	1.26	1.62	Escherichia_coli_91	Bacteria_Proteobacteria_91_Gammaproteobacteria_91_Enterobacterales_91_Enterobacteriaceae_90_Pseudescherichia_89
OTU_523	7.46E-01	NA	2.54E-02	2.90	0.00	Desulfovibrio_desulfuricans_89	Bacteria_Proteobacteria_Deltaproteobacteria_99_Desulfovibrionales_99_Desulfovibrionaceae_99_Mailhella_77
OTU_542	7.07E-01	6.04E-01	3.17E-01	0.43	0.09	Bifidobacterium_apri_99	Bacteria_Actinobacteria_94_Actinobacteria_94_Bifidobacteriales_94_Bifidobacteriaceae_94_Neoscardovia_38
OTU_10443	9.87E-01	7.42E-01	2.08E-02	1.92	0.63	Bacteroides_stercorirosoris_97	Bacteria_Bacteroidetes_Bacteroidia_Bacteroidales_Bacteroidaceae_Bacteroides_100
OTU_11241	8.43E-01	6.57E-01	3.49E-02	2.26	0.64	Sutterella_massiliensis_97	Bacteria_Proteobacteria_Betaproteobacteria_Burkholderiales_Sutterellaceae_Sutterella_100
OTU_1501	8.57E-01	8.65E-01	8.20E-03	1.57	0.40	Enterococcus_dispar_98	Bacteria_Firmicutes_61_Bacilli_61_Lactobacillales_61_Carnobacteriaceae_58_Isobaculum_33
OTU_1818	7.94E-01	7.11E-01	6.22E-01	0.96	1.19	Pseudescherichia_vulneris_88	Bacteria_Firmicutes_85_Clostridia_85_Clostridiales_85_Lachnospiraceae_85_Dorea_69
OTU_2219	7.07E-01	6.04E-01	4.84E-02	1.37	0.48	Veillonella_tobetsuensis_97	Bacteria_Firmicutes_Negativicutes_Veillonellales_Veillonellaceae_Veillonella_100
OTU_320	NA	6.06E-01	1.54E-01	0.00	2.43	Kofleria_flava_92	Bacteria_Proteobacteria_Deltaproteobacteria_Myxococcales_99_Nannocystineae_98_Kofleriaceae_83_Kofleria_83
OTU_3839	9.22E-01	9.25E-01	4.34E-02	0.51	2.71	Bacteroides_fragilis_88	Bacteria_Firmicutes_53_Clostridia_53_Clostridiales_53_Lachnospiraceae_53_Lactonifactor_15
OTU_5098	8.54E-01	8.14E-01	1.07E-01	0.90	1.35	Enterocloster_citroniae_97	Bacteria_Firmicutes_Clostridia_Clostridiales_Lachnospiraceae_Enterocloster_96
OTU_10192	8.51E-01	9.35E-01	4.03E-03	2.22	0.42	Bacteroides_fragilis_98	Bacteria_Bacteroidetes_56_Bacteroidia_56_Bacteroidales_56_Bacteroidaceae_55_Mediterranea_44
OTU_1118	7.73E-01	7.36E-01	1.91E-02	2.76	0.64	Bacteroides_faecichinchillae_96	Bacteria_Bacteroidetes_Bacteroidia_Bacteroidales_Bacteroidaceae_96_Bacteroides_96
OTU_1896	9.65E-01	9.52E-01	4.12E-04	2.17	0.56	Clostridium_tarantellae_91	Bacteria_Firmicutes_99_Clostridia_99_Clostridiales_99_Lachnospiraceae_69_Anaerotaenia_24
OTU_2044	7.07E-01	8.18E-01	1.36E-01	2.38	0.82	Roseburia_inulinivorans_94	Bacteria_Firmicutes_Clostridia_Clostridiales_Lachnospiraceae_Roseburia_66
OTU_235	8.29E-01	6.04E-01	3.33E-01	1.01	0.38	Olsenella_profusa_95	Bacteria_Actinobacteria_Coriobacteriia_Coriobacteriales_99_Atopobiaceae_98_Olsenella_98
OTU_2952	8.60E-01	6.63E-01	6.69E-01	1.52	1.34	Pseudescherichia_vulneris_94	Bacteria_Proteobacteria_95_Gammaproteobacteria_95_Enterobacterales_95_Enterobacteriaceae_95_Pseudescherichia_76
OTU_3675	8.06E-01	8.28E-01	3.06E-01	1.33	1.95	Enterococcus_hirae_93	Bacteria_Firmicutes_99_Bacilli_86_Lactobacillales_86_Enterococcaceae_52_Melissococcus_32
OTU_714	7.07E-01	6.04E-01	4.59E-01	1.37	0.58	Dialister_propionicifaciens_95	Bacteria_Firmicutes_71_Negativicutes_71_Veillonellales_71_Veillonellaceae_71_Dialister_65
OTU_10671	9.97E-01	6.04E-01	2.83E-02	2.01	0.42	Tyzzerella_nexilis_98	Bacteria_Firmicutes_Clostridia_Clostridiales_Lachnospiraceae_Tyzzerella_49
OTU_1169	7.12E-01	6.04E-01	1.80E-02	2.68	0.43	Dialister_pneumosintes_99	Bacteria_Firmicutes_Negativicutes_84_Veillonellales_84_Veillonellaceae_84_Dialister_79
OTU_12576	9.10E-01	9.46E-01	9.96E-02	0.82	1.32	Desemzia_incerta_88	Bacteria_Firmicutes_Negativicutes_70_Selenomonadales_41_Sporomusaceae_41_Anaerosinus_39
OTU_2284	8.85E-01	6.66E-01	1.11E-01	0.91	1.74	Pseudoflavonifractor_capillosus_97	Bacteria_Firmicutes_Clostridia_Clostridiales_Ruminococcaceae_Flavonifractor_47
OTU_61498	9.67E-01	6.04E-01	6.97E-02	0.25	2.30	Erysipelatoclostridium_ramosum_92	Bacteria_Firmicutes_Erysipelotrichia_Erysipelotrichales_Erysipelatoclostridiaceae_99_Erysipelatoclostridium_99
OTU_66014	7.10E-01	9.77E-01	7.32E-02	0.91	1.52	Enterocloster_clostridioformis_96	Bacteria_Firmicutes_Clostridia_Clostridiales_Lachnospiraceae_Enterocloster_92
OTU_817	8.87E-01	6.55E-01	1.19E-02	2.76	0.14	Clostridium_carnis_89	Bacteria_Firmicutes_63_Clostridia_62_Clostridiales_62_Clostridiaceae 1_55_Anaerobacter_43
OTU_936	9.31E-01	8.17E-01	2.63E-02	2.06	0.73	Peptoniphilus_tyrrelliae_94	Bacteria_Firmicutes_99_Clostridia_99_Clostridiales_99_Peptoniphilaceae_99_Peptoniphilus_99
OTU_11657	7.07E-01	8.50E-01	2.18E-01	0.87	1.60	Bacteroides_fragilis_97	Bacteria_Bacteroidetes_Bacteroidia_Bacteroidales_Bacteroidaceae_Bacteroides_100
OTU_14320	1.00E+00	9.52E-01	7.35E-01	1.14	1.30	Escherichia_albertii_87	Bacteria_Proteobacteria_48_Gammaproteobacteria_48_Enterobacterales_48_Enterobacteriaceae_48_Pseudescherichia_48
OTU_1534	8.25E-01	7.34E-01	2.03E-02	0.68	1.53	Bacteroides_intestinalis_88	Bacteria_Firmicutes_98_Clostridia_98_Clostridiales_98_Ruminococcaceae_98_Flavonifractor_91
OTU_2109	7.57E-01	6.04E-01	3.77E-02	0.67	2.38	Bacteroides_acidifaciens_89	Bacteria_Bacteroidetes_61_Bacteroidia_61_Bacteroidales_61_Bacteroidaceae_60_Bacteroides_56

OTU_31807	7.16E-01	7.96E-01	3.27E-03	0.71	2.34	Bacteroides_thetaiotaomicron_98	Bacteria_Bacteroidetes_Bacteroidia_Bacteroidales_Bacteroidaceae_Bacteroides_100
OTU_4027	9.31E-01	7.70E-01	9.52E-03	2.16	0.66	Pseudescherichia_vulneris_97	Bacteria_Proteobacteria_94_Gammaproteobacteria_94_Enterobacterales_94_Enterobacteriaceae_94_Pseudescherichia_83
OTU_422	8.20E-01	NA	6.43E-02	3.86	0.00	Natranaerovirga_pectinivora_90	Bacteria_Firmicutes_98_Clostridia_98_Clostridiales_98_Ruminococcaceae_44_Ercella_15
OTU_4464	7.07E-01	6.04E-01	7.30E-01	1.17	1.42	Klebsiella_pneumoniae_98	Bacteria_Proteobacteria_Gammaproteobacteria_Enterobacterales_Erwiniaceae_36_Erwinia_35
OTU_4705	7.07E-01	9.18E-01	1.99E-02	1.85	0.51	Bacteroides_xylanisolvens_97	Bacteria_Bacteroidetes_Bacteroidia_Bacteroidales_Bacteroidaceae_Bacteroides_100
OTU_4958	7.10E-01	8.34E-01	4.70E-03	0.63	2.23	Bacteroides_faecis_91	Bacteria_Bacteroidetes_91_Bacteroidia_91_Bacteroidales_91_Bacteroidaceae_91_Bacteroides_38
OTU_750	8.94E-01	6.04E-01	1.33E-01	1.36	0.15	Clostridium_populeti_95	Bacteria_Firmicutes_Clostridia_Clostridiales_Lachnospiraceae_Ruminococcus2_35
OTU_7563	8.12E-01	6.04E-01	5.75E-01	1.69	1.31	Veillonella_criceti_89	Bacteria_Firmicutes_Clostridia_93_Clostridiales_93_Peptostreptococcaceae_93_Paraclostridium_88
OTU_1149	7.16E-01	6.04E-01	9.49E-02	0.38	2.31	Paraclostridium_benzoelyticum_96	Bacteria_Firmicutes_Clostridia_Clostridiales_Peptostreptococcaceae_Paraclostridium_100
OTU_1427	7.68E-01	6.04E-01	8.58E-01	1.30	1.15	Mixta_theicola_96	Bacteria_Proteobacteria_Gammaproteobacteria_Enterobacterales_Enterobacteriaceae_93_Atlantibacter_15
OTU_14402	7.86E-01	7.32E-01	4.45E-02	0.62	2.33	Bacteroides_thetaiotaomicron_97	Bacteria_Bacteroidetes_Bacteroidia_Bacteroidales_Bacteroidaceae_Bacteroides_99
OTU_24730	7.07E-01	8.51E-01	1.30E-01	0.89	1.42	Enterococcus_faecalis_98	Bacteria_Firmicutes_Bacilli_Lactobacillales_Enterococcaceae_97_Enterococcus_77
OTU_3079	7.07E-01	7.66E-01	1.71E-02	1.60	0.55	Coprococcus_comes_91	Bacteria_Firmicutes_Clostridia_Clostridiales_Lachnospiraceae_Bariatricus_77
OTU_5409	7.46E-01	9.82E-01	1.48E-01	0.91	1.59	Veillonella_dispar_97	Bacteria_Firmicutes_Negativicutes_Veillonellales_98_Veillonellaceae_98_Veillonella_98
OTU_6337	7.07E-01	7.02E-01	1.72E-01	1.81	0.46	Pseudoflavonifractor_phocaeensis_97	Bacteria_Firmicutes_Clostridia_Clostridiales_Ruminococcaceae_Intestinimonas_68
OTU_864	NA	8.36E-01	1.03E-02	0.00	2.13	Piscinibacter_aquaticus_100	Bacteria_Proteobacteria_Betaproteobacteria_Burkholderiales_Comamonadaceae_Piscinibacter_100
OTU_1176	7.07E-01	8.14E-01	5.38E-01	0.94	1.15	Flavonifractor_plautii_97	Bacteria_Firmicutes_Clostridia_Clostridiales_Ruminococcaceae_Flavonifractor_100
OTU_1792	7.07E-01	6.04E-01	1.14E-01	1.58	0.73	Enterococcus_faecalis_96	Bacteria_Firmicutes_Bacilli_Lactobacillales_99_Enterococcaceae_75_Enterococcus_59
OTU_2132	7.85E-01	9.32E-01	4.20E-01	1.23	0.97	Enterocloster_clostridioformis_91	Bacteria_Firmicutes_98_Clostridia_98_Clostridiales_98_Lachnospiraceae_94_Enterocloster_44
OTU_2974	8.97E-01	6.04E-01	9.35E-01	1.51	1.45	Ruminococcus_gnavus_97	Bacteria_Firmicutes_Clostridia_Clostridiales_Lachnospiraceae_Lachnospiracea_incertae_sedis_81
OTU_1015	7.07E-01	6.05E-01	2.51E-01	1.46	0.52	Fusobacterium_mortiferum_95	Bacteria_Fusobacteria_99_Fusobacteriia_99_Fusobacteriales_99_Fusobacteriaceae_92_Cetobacterium_57
OTU_13473	7.07E-01	8.49E-01	1.32E-01	0.93	1.51	Bacteroides_thetaiotaomicron_97	Bacteria_Bacteroidetes_79_Bacteroidia_79_Bacteroidales_79_Bacteroidaceae_78_Mediterranea_64
OTU_16574	7.07E-01	6.04E-01	1.42E-01	1.27	0.59	Collinsella_aerofaciens_96	Bacteria_Actinobacteria_Coriobacteriia_Coriobacteriales_Coriobacteriaceae_Collinsella_100
OTU_1917	9.65E-01	7.84E-01	4.44E-01	1.27	1.61	Roseburia_inulinivorans_94	Bacteria_Firmicutes_95_Clostridia_95_Clostridiales_95_Lachnospiraceae_95_Roseburia_67
OTU_2244	8.75E-01	8.72E-01	5.91E-02	1.57	0.80	Clostridium_perfringens_90	Bacteria_Firmicutes_Bacilli_79_Lactobacillales_62_Carnobacteriaceae_57_Isobaculum_48
OTU_311	NA	9.70E-01	1.24E-01	0.00	1.09	Aquisediminimonas_profunda_100	Bacteria_Proteobacteria_Alphaproteobacteria_Sphingomonadales_Sphingomonadaceae_Aquisediminimonas_92
OTU_32765	8.57E-01	8.45E-01	1.46E-02	0.48	2.00	Lachnoclostridium_pacaense_93	Bacteria_Firmicutes_Clostridia_Clostridiales_Lachnospiraceae_Clostridium XlVa_42
OTU_7305	8.25E-01	6.14E-01	8.36E-02	0.90	1.76	Vagococcus_carniphilus_92	Bacteria_Firmicutes_Erysipelotrichia_73_Erysipelotrichales_73_Erysipelatoclostridiaceae_73_Erysipelatoclostridium_73
OTU_811	8.25E-01	6.73E-01	1.79E-03	0.11	1.04	Fusicatenibacter_saccharivorans_91	Bacteria_Firmicutes_Clostridia_Clostridiales_Lachnospiraceae_98_Fusicatenibacter_14
OTU_9247	9.38E-01	6.62E-01	6.69E-02	0.87	0.31	Faecalicatena_orotica_95	Bacteria_Firmicutes_Clostridia_Clostridiales_Lachnospiraceae_Clostridium XlVa_76
OTU_1046	7.91E-01	8.68E-01	1.47E-01	2.90	0.07	Bacteroides_stercoris_100	Bacteria_Bacteroidetes_Bacteroidia_Bacteroidales_Bacteroidaceae_98_Mediterranea_78
OTU_2002	8.89E-01	9.54E-01	3.51E-01	0.69	0.45	Faecalibacterium_prausnitzii_94	Bacteria_Firmicutes_Clostridia_Clostridiales_Ruminococcaceae_Faecalibacterium_98
OTU_2211	8.57E-01	9.18E-01	9.65E-01	0.22	0.23	Eubacterium_ramulus_95	Bacteria_Firmicutes_Clostridia_Clostridiales_Lachnospiraceae_Lachnospiracea_incertae_sedis_47
OTU_2240	8.49E-01	8.72E-01	5.06E-03	0.36	2.10	Gibbsiella_dentisursi_86	Bacteria_Proteobacteria_59_Gammaproteobacteria_59_Enterobacterales_59_Enterobacteriaceae_59_Pseudescherichia_59
OTU_4774	7.07E-01	6.04E-01	9.57E-02	1.35	0.50	Pseudescherichia_vulneris_97	Bacteria_Proteobacteria_Gammaproteobacteria_Enterobacterales_Enterobacteriaceae_Escherichia/Shigella_73
OTU_482	7.46E-01	6.04E-01	2.33E-01	0.20	0.05	Fusobacterium_mortiferum_95	Bacteria_Fusobacteria_87_Fusobacteriia_87_Fusobacteriales_87_Fusobacteriaceae_82_Cetobacterium_47
OTU_1228	7.07E-01	9.46E-01	6.80E-02	0.05	1.72	Kluyvera_georgiana_92	Bacteria_Proteobacteria_Gammaproteobacteria_86_Enterobacterales_86_Enterobacteriaceae_86_Pseudescherichia_64
OTU_1374	7.12E-01	9.84E-01	4.94E-04	1.98	0.30	Enterocloster_citroniae_95	Bacteria_Firmicutes_Clostridia_Clostridiales_Lachnospiraceae_99_Enterocloster_64
OTU_1490	7.11E-01	9.41E-01	2.84E-03	2.37	0.36	Streptococcus_rubneri_90	Bacteria_Firmicutes_Clostridia_81_Clostridiales_81_Lachnospiraceae_81_Clostridium XlVa_36
OTU_16892	8.06E-01	6.04E-01	7.71E-01	1.35	1.05	Bifidobacterium_breve_89	Bacteria_Actinobacteria_91_Actinobacteria_91_Bifidobacteriales_91_Bifidobacteriaceae_91_Neoscardovia_45
OTU_16955	9.31E-01	9.35E-01	5.80E-02	0.87	1.48	Pseudescherichia_vulneris_91	Bacteria_Proteobacteria_85_Gammaproteobacteria_85_Enterobacterales_85_Enterobacteriaceae_83_Pseudescherichia_83
OTU_2253	9.93E-01	7.24E-01	1.80E-02	0.64	1.39	Flavonifractor_plautii_97	Bacteria_Firmicutes_Clostridia_Clostridiales_Ruminococcaceae_Flavonifractor_100
OTU_8534	7.59E-01	7.34E-01	3.88E-02	0.33	1.33	Enterococcus_faecalis_91	Bacteria_Firmicutes_Negativicutes_90_Acidaminococcales_90_Acidaminococcaceae_90_Phascolarctobacterium_84
OTU_10218	7.97E-01	6.04E-01	1.98E-02	1.97	0.84	Bacteroides_xylanisolvens_98	Bacteria_Bacteroidetes_91_Bacteroidia_91_Bacteroidales_91_Bacteroidaceae_90_Bacteroides_61
OTU_11123	7.07E-01	6.73E-01	1.46E-02	1.94	0.34	Bacteroides_uniformis_99	Bacteria_Bacteroidetes_Bacteroidia_Bacteroidales_Bacteroidaceae_95_Mediterranea_70
OTU_1119	7.68E-01	9.25E-01	3.65E-01	1.70	1.16	Pseudescherichia_vulneris_96	Bacteria_Proteobacteria_Gammaproteobacteria_Enterobacterales_93_Enterobacteriaceae_93_Escherichia/Shigella_69
OTU_18697	7.12E-01	8.50E-01	3.00E-02	1.21	0.10	Phocaeicola_vulgatus_97	Bacteria_Bacteroidetes_Bacteroidia_Bacteroidales_Bacteroidaceae_Phocaeicola_65
OTU_18863	9.38E-01	9.25E-01	1.21E-01	1.73	0.87	Kineothrix_alysoides_94	Bacteria_Firmicutes_Clostridia_Clostridiales_Lachnospiraceae_Clostridium XlVa_41
OTU_2830	7.93E-01	6.04E-01	2.84E-04	0.50	2.15	Bacteroides_thetaiotaomicron_96	Bacteria_Bacteroidetes_Bacteroidia_Bacteroidales_Bacteroidaceae_Bacteroides_100
OTU_3327	9.14E-01	9.70E-01	2.77E-03	0.51	2.46	Bacteroides_ovatus_94	Bacteria_Bacteroidetes_Bacteroidia_Bacteroidales_Bacteroidaceae_Bacteroides_81
OTU_3560	7.57E-01	6.57E-01	1.84E-01	1.69	1.07	Roseburia_inulinivorans_91	Bacteria_Firmicutes_Clostridia_67_Clostridiales_67_Lachnospiraceae_62_Tyzzerella_8
OTU_383	8.48E-01	6.04E-01	6.87E-02	0.45	0.09	Megasphaera_elsdenii_100	Bacteria_Firmicutes_98_Negativicutes_98_Veillonellales_98_Veillonellaceae_98_Megasphaera_94
OTU_39720	7.07E-01	8.17E-01	3.32E-04	2.20	0.18	Bacteroides_caecimuris_95	Bacteria_Bacteroidetes_Bacteroidia_Bacteroidales_Bacteroidaceae_Bacteroides_83
OTU_4925	8.06E-01	9.35E-01	4.04E-01	1.24	1.56	Enterocloster_aldenensis_97	Bacteria_Firmicutes_Clostridia_Clostridiales_Lachnospiraceae_Enterocloster_74
OTU_62631	9.03E-01	6.72E-01	8.39E-01	1.30	1.22	Coprococcus_comes_96	Bacteria_Firmicutes_Clostridia_Clostridiales_Lachnospiraceae_Coprococcus_58
OTU_683	7.75E-01	6.63E-01	3.95E-02	2.38	0.23	Anaerotignum_lactatifermentans_97	Bacteria_Firmicutes_Clostridia_Clostridiales_Lachnospiraceae_Anaerotignum_100
OTU_7433	9.59E-01	6.04E-01	9.43E-01	1.15	1.17	Escherichia_albertii_89	Bacteria_Proteobacteria_74_Gammaproteobacteria_74_Enterobacterales_74_Enterobacteriaceae_74_Pseudescherichia_72
OTU_7630	7.07E-01	6.04E-01	3.53E-01	0.96	0.64	Parabacteroides_distasonis_93	Bacteria_Bacteroidetes_Bacteroidia_Bacteroidales_Porphyromonadaceae_73_Parabacteroides_73
OTU_1172	7.12E-01	9.12E-01	2.55E-02	2.88	0.25	Enterococcus_faecalis_91	Bacteria_Firmicutes_57_Bacilli_57_Lactobacillales_55_Carnobacteriaceae_49_Catellicoccus_47
OTU_12069	8.23E-01	9.46E-01	1.13E-01	0.75	1.56	Clostridium_tepidum_97	Bacteria_Firmicutes_Clostridia_Clostridiales_Clostridiaceae 1_97_Clostridium sensu stricto_96
OTU_14764	7.07E-01	6.04E-01	5.82E-02	0.84	1.73	Pseudescherichia_vulneris_95	Bacteria_Proteobacteria_Gammaproteobacteria_Enterobacterales_Enterobacteriaceae_Escherichia/Shigella_65
OTU_14779	7.57E-01	9.09E-01	2.07E-02	0.62	1.84	Veillonella_tobetsuensis_90	Bacteria_Firmicutes_Clostridia_88_Clostridiales_88_Lachnospiraceae_88_Enterocloster_42
OTU_241	7.91E-01	NA	2.84E-01	2.81	0.00	Prevotella_loescheii_92	Bacteria_Bacteroidetes_Bacteroidia_Bacteroidales_Prevotellaceae_Prevotella_100
OTU_28019	7.57E-01	8.14E-01	6.66E-04	2.33	0.56	Clostridium_perfringens_99	Bacteria_Firmicutes_90_Clostridia_90_Clostridiales_90_Clostridiaceae 1_90_Sarcina_36
OTU_3634	7.93E-01	7.02E-01	2.91E-02	1.97	0.57	Clostridium_perfringens_96	Bacteria_Firmicutes_Clostridia_Clostridiales_Clostridiaceae 1_Clostridium sensu stricto_68
OTU_4119	9.35E-01	9.54E-01	2.58E-02	2.35	0.30	Veillonella_atypica_89	Bacteria_Firmicutes_Clostridia_89_Clostridiales_89_Lachnospiraceae_89_Merdimonas_28
OTU_551	9.67E-01	6.21E-01	1.67E-02	2.71	0.17	Desulfovibrio_desulfuricans_97	Bacteria_Proteobacteria_Deltaproteobacteria_Desulfovibrionales_Desulfovibrionaceae_Desulfovibrio_98

OTU_841	7.07E-01	7.61E-01	1.26E-02	1.70	0.09	Dialister_pneumosintes_94	Bacteria_Firmicutes_Negativicutes_Veillonellales_Veillonellaceae_Dialister_71
OTU_9784	7.07E-01	9.85E-01	9.58E-04	1.47	0.18	Anaerobacillus_alkalilacustris_92	Bacteria_Firmicutes_Bacilli_92_Lactobacillales_75_Carnobacteriaceae_74_Isobaculum_55
OTU_15391	7.82E-01	6.99E-01	4.54E-02	2.25	0.16	Veillonella_tobetsuensis_98	Bacteria_Firmicutes_Negativicutes_Veillonellales_Veillonellaceae_Veillonella_100
OTU_15552	7.07E-01	7.80E-01	2.77E-01	0.78	1.26	Bacteroides_kribbi_97	Bacteria_Bacteroidetes_Bacteroidia_Bacteroidales_Bacteroidaceae_Bacteroides_98
OTU_16236	7.73E-01	8.14E-01	7.02E-01	1.06	1.20	Falcatimonas_natans_91	Bacteria_Firmicutes_Clostridia_60_Clostridiales_60_Lachnospiraceae_60_Falcatimonas_16
OTU_1854	9.37E-01	6.49E-01	7.00E-01	0.86	0.71	Lachnoclostridium_pacaense_94	Bacteria_Firmicutes_Clostridia_Clostridiales_Lachnospiraceae_Enterocloster_45
OTU_27132	9.84E-01	7.83E-01	4.51E-03	1.88	0.75	Bacteroides_xylanisolvens_96	Bacteria_Proteobacteria_53_Gammaproteobacteria_53_Enterobacterales_53_Enterobacteriaceae_53_Pseudescherichia_52
OTU_27475	7.07E-01	9.01E-01	2.89E-03	2.83	0.36	Bacteroides_xylanisolvens_97	Bacteria_Bacteroidetes_Bacteroidia_Bacteroidales_Bacteroidaceae_Bacteroides_100
OTU_27764	9.38E-01	8.02E-01	3.15E-01	1.59	1.07	Enterococcus_hirae_96	Bacteria_Firmicutes_Bacilli_Lactobacillales_98_Enterococcaceae_73_Enterococcus_52
OTU_35148	8.48E-01	6.96E-01	1.92E-02	0.67	2.04	Paraclostridium_benzoelyticum_97	Bacteria_Firmicutes_Clostridia_Clostridiales_Peptostreptococcaceae_Paraclostridium_95
OTU_3663	7.10E-01	8.50E-01	5.97E-02	2.09	0.40	Clostridium_perfringens_96	Bacteria_Firmicutes_Clostridia_62_Clostridiales_62_Clostridiaceae 1_62_Anaerobacter_24
OTU_3796	8.83E-01	8.14E-01	9.19E-01	1.30	1.35	Lachnoclostridium_pacaense_97	Bacteria_Firmicutes_Clostridia_Clostridiales_Lachnospiraceae_Enterocloster_56
OTU_3974	7.07E-01	4.60E-01	1.36E-01	0.83	1.46	Loigolactobacillus_bifermentans_89	Bacteria_Firmicutes_Erysipelotrichia_57_Erysipelotrichales_57_Erysipelatoclostridiaceae_56_Erysipelatoclostridium_56
OTU_4904	7.91E-01	9.46E-01	8.38E-03	0.05	1.83	Burkholderia_rinojensis_91	Bacteria_Proteobacteria_Gammaproteobacteria_79_Enterobacterales_79_Enterobacteriaceae_79_Pseudescherichia_58
OTU_6324	7.07E-01	8.14E-01	1.83E-02	1.99	0.51	Hungatella_hathewayi_90	Bacteria_Firmicutes_Clostridia_66_Clostridiales_66_Lachnospiraceae_66_Falcatimonas_29
OTU_7613	9.00E-01	6.50E-01	2.76E-02	0.83	1.89	Veillonella_dispar_96	Bacteria_Firmicutes_Negativicutes_99_Veillonellales_99_Veillonellaceae_99_Veillonella_99
OTU_9603	8.19E-01	6.04E-01	9.67E-02	1.74	1.00	Clostridium_perfringens_96	Bacteria_Firmicutes_Clostridia_99_Clostridiales_99_Clostridiaceae 1_99_Clostridium sensu stricto_64
OTU_11713	9.62E-01	9.35E-01	3.07E-01	1.76	1.19	Coprococcus_comes_97	Bacteria_Firmicutes_Clostridia_Clostridiales_Lachnospiraceae_Coprococcus_36
OTU_18368	7.86E-01	6.14E-01	9.72E-01	1.15	1.14	Lacrimispora_xylanolytica_91	Bacteria_Firmicutes_Clostridia_43_Clostridiales_43_Lachnospiraceae_43_Anaerotaenia_8
OTU_2320	9.79E-01	6.93E-01	3.99E-02	0.26	1.60	Enterococcus_faecalis_92	Bacteria_Firmicutes_Negativicutes_65_Acidaminococcales_65_Acidaminococcaceae_65_Phascolarctobacterium_65
OTU_28330	7.81E-01	7.34E-01	1.74E-02	1.64	0.75	Clostridium_perfringens_96	Bacteria_Firmicutes_Clostridia_Clostridiales_Clostridiaceae 1_80_Sarcina_23
OTU_32416	7.07E-01	6.68E-01	2.80E-01	1.74	1.25	Bacteroides_thetaiotaomicron_97	Bacteria_Bacteroidetes_Bacteroidia_Bacteroidales_Bacteroidaceae_Bacteroides_100
OTU_5443	8.06E-01	6.72E-01	1.40E-02	3.16	0.23	Streptococcus_sinensis_98	Bacteria_Firmicutes_Bacilli_Lactobacillales_Streptococcaceae_Streptococcus_100
OTU_61655	9.38E-01	6.04E-01	1.63E-02	0.62	1.86	Bacteroides_thetaiotaomicron_97	Bacteria_Bacteroidetes_Bacteroidia_Bacteroidales_Bacteroidaceae_Bacteroides_100
OTU_713	8.25E-01	6.63E-01	2.94E-01	0.77	0.39	Collinsella_intestinalis_100	Bacteria_Actinobacteria_Coriobacteriia_Coriobacteriales_Coriobacteriaceae_Collinsella_100
OTU_101243	8.48E-01	6.04E-01	1.25E-02	3.05	0.15	Bacteroides_xylanisolvens_98	Bacteria_Bacteroidetes_Bacteroidia_Bacteroidales_Bacteroidaceae_Bacteroides_100
OTU_12469	8.54E-01	9.34E-01	1.34E-02	1.59	0.16	Bacteroides_stercoris_95	Bacteria_Bacteroidetes_Bacteroidia_Bacteroidales_Bacteroidaceae_Bacteroides_72
OTU_1492	7.07E-01	9.90E-01	3.73E-02	2.19	0.14	Bacteroides_stercoris_94	Bacteria_Bacteroidetes_Bacteroidia_Bacteroidales_Porphyromonadaceae_56_Parabacteroides_56
OTU_1508	7.91E-01	7.73E-01	3.21E-02	1.56	0.53	Bacteroides_thetaiotaomicron_90	Bacteria_Bacteroidetes_99_Bacteroidia_99_Bacteroidales_99_Bacteroidaceae_97_Bacteroides_71
OTU_346	8.33E-01	NA	1.37E-01	2.40	0.00	Anaerostipes_hadrus_96	Bacteria_Firmicutes_Clostridia_Clostridiales_Lachnospiraceae_Anaerostipes_36
OTU_364	NA	7.46E-01	2.04E-01	0.00	1.88	Simplicispira_soli_98	Bacteria_Proteobacteria_Betaproteobacteria_Burkholderiales_Comamonadaceae_Simplicispira_96
OTU_39644	8.61E-01	6.47E-01	3.08E-05	2.66	0.23	Bacteroides_xylanisolvens_98	Bacteria_Bacteroidetes_Bacteroidia_Bacteroidales_Bacteroidaceae_Bacteroides_100
OTU_46554	7.07E-01	6.04E-01	3.80E-02	0.56	1.70	Erysipelatoclostridium_ramosum_96	Bacteria_Firmicutes_Erysipelotrichia_Erysipelotrichales_Erysipelatoclostridiaceae_Erysipelatoclostridium_100
OTU_5028	9.87E-01	8.87E-01	1.31E-01	0.79	1.30	Flavonifractor_plautii_92	Bacteria_Firmicutes_97_Clostridia_97_Clostridiales_97_Ruminococcaceae_97_Flavonifractor_97
OTU_672	8.29E-01	6.04E-01	1.70E-02	2.66	0.20	Bacteroides_stercoris_91	Bacteria_Bacteroidetes_96_Bacteroidia_95_Bacteroidales_95_Bacteroidaceae_95_Mediterranea_53
OTU_7275	9.31E-01	9.25E-01	2.61E-01	0.92	1.34	Lachnoclostridium_pacaense_95	Bacteria_Firmicutes_Clostridia_Clostridiales_Lachnospiraceae_Clostridium XlVa_73
OTU_75479	7.07E-01	6.35E-01	2.07E-01	2.20	0.55	Collinsella_aerofaciens_96	Bacteria_Actinobacteria_Coriobacteriia_Coriobacteriales_Coriobacteriaceae_Collinsella_100
OTU_960	8.47E-01	9.53E-01	2.77E-01	0.39	0.20	Enterocloster_asparagiformis_96	Bacteria_Firmicutes_Clostridia_Clostridiales_Lachnospiraceae_Clostridium XlVa_63
OTU_10594	9.61E-01	9.64E-01	2.52E-02	0.55	1.65	Bacteroides_fragilis_94	Bacteria_Bacteroidetes_98_Bacteroidia_98_Bacteroidales_98_Bacteroidaceae_98_Bacteroides_91
OTU_1144	7.07E-01	8.14E-01	6.69E-03	0.14	2.75	Phascolarctobacterium_faecium_100	Bacteria_Firmicutes_Negativicutes_Acidaminococcales_Acidaminococcaceae_Phascolarctobacterium_100
OTU_1156	9.52E-01	8.73E-01	9.69E-01	0.40	0.39	Megamonas_funiformis_100	Bacteria_Firmicutes_Negativicutes_94_Selenomonadales_94_Selenomonadaceae_94_Megamonas_94
OTU_13348	7.57E-01	9.90E-01	9.14E-01	1.24	1.19	Coprococcus_comes_97	Bacteria_Firmicutes_Clostridia_Clostridiales_Lachnospiraceae_Coprococcus_45
OTU_1378	9.91E-01	9.57E-01	1.12E-01	1.73	0.87	Clostridium_symbiosum_90	Bacteria_Firmicutes_99_Clostridia_99_Clostridiales_99_Lachnospiraceae_89_Moryella_17
OTU_1767	9.05E-01	9.97E-01	8.44E-01	1.27	1.45	Limosilactobacillus_mucosae_90	Bacteria_Proteobacteria_85_Gammaproteobacteria_85_Enterobacterales_85_Enterobacteriaceae_85_Pseudescherichia_84
OTU_40547	7.85E-01	9.70E-01	8.49E-01	1.08	1.15	Pseudescherichia_vulneris_90	Bacteria_Proteobacteria_91_Gammaproteobacteria_91_Enterobacterales_90_Enterobacteriaceae_87_Pseudescherichia_86
OTU_4173	8.37E-01	9.53E-01	1.18E-01	1.72	1.08	Pseudescherichia_vulneris_96	Bacteria_Proteobacteria_83_Gammaproteobacteria_83_Enterobacterales_83_Enterobacteriaceae_81_Pseudescherichia_71
OTU_69035	7.07E-01	6.04E-01	5.05E-02	0.34	0.04	Veillonella_denticariosi_98	Bacteria_Firmicutes_Negativicutes_Veillonellales_Veillonellaceae_Veillonella_100
OTU_982	8.12E-01	9.12E-01	7.23E-02	2.29	0.24	Lacrimispora_aerotolerans_95	Bacteria_Firmicutes_Clostridia_Clostridiales_Lachnospiraceae_99_Hungatella_49
OTU_11995	8.61E-01	9.25E-01	3.94E-01	1.12	0.85	Pseudescherichia_vulneris_96	Bacteria_Proteobacteria_Gammaproteobacteria_Enterobacterales_Enterobacteriaceae_Escherichia/Shigella_79
OTU_1909	8.82E-01	7.76E-01	2.70E-01	1.69	1.11	Bacteroides_kribbi_97	Bacteria_Bacteroidetes_Bacteroidia_Bacteroidales_Bacteroidaceae_Bacteroides_100
OTU_2060	9.01E-01	9.75E-01	2.71E-02	1.30	0.44	Peptoniphilus_koenoeneniae_96	Bacteria_Firmicutes_Clostridia_Clostridiales_Peptoniphilaceae_Peptoniphilus_100
OTU_43208	7.59E-01	7.02E-01	2.11E-02	1.82	0.30	Veillonella_atypica_96	Bacteria_Firmicutes_Negativicutes_Veillonellales_97_Veillonellaceae_97_Veillonella_97
OTU_444	9.37E-01	8.05E-01	8.00E-02	3.42	0.08	Vallitalea_pronyensis_87	Bacteria_Firmicutes_97_Clostridia_95_Clostridiales_95_Peptostreptococcaceae_27_Criibacterium_27
OTU_4771	9.07E-01	7.01E-01	6.91E-01	0.94	1.20	Escherichia_albertii_91	Bacteria_Proteobacteria_99_Gammaproteobacteria_99_Enterobacterales_99_Enterobacteriaceae_99_Pseudescherichia_92
OTU_585	7.07E-01	6.04E-01	2.14E-01	1.63	0.34	Cetobacterium_somerae_92	Bacteria_Fusobacteria_Fusobacteriia_Fusobacteriales_Fusobacteriaceae_98_Cetobacterium_72
OTU_8536	7.80E-01	8.28E-01	2.96E-03	2.00	0.68	Clostridium_tarantellae_94	Bacteria_Firmicutes_Clostridia_Clostridiales_Lachnospiraceae_67_Robinsoniella_11
OTU_906	1.00E+00	9.41E-01	2.21E-01	0.46	0.21	Catenibacterium_mitsuokai_94	Bacteria_Firmicutes_Erysipelotrichia_Erysipelotrichales_Erysipelotrichaceae_Catenibacterium_97
OTU_9789	8.85E-01	7.31E-01	5.70E-03	2.01	0.63	Bacteroides_caccae_97	Bacteria_Bacteroidetes_Bacteroidia_Bacteroidales_Bacteroidaceae_Bacteroides_100
OTU_9842	8.16E-01	6.04E-01	6.91E-04	2.07	0.39	Bacteroides_faecichinchillae_97	Bacteria_Bacteroidetes_Bacteroidia_Bacteroidales_Bacteroidaceae_Bacteroides_100
OTU_1030	7.07E-01	6.04E-01	2.19E-01	0.36	0.10	Phocaeicola_vulgatus_95	Bacteria_Bacteroidetes_Bacteroidia_Bacteroidales_Bacteroidaceae_Phocaeicola_64
OTU_10686	9.60E-01	6.04E-01	1.24E-02	1.47	0.07	Phascolarctobacterium_succinatutens_97	Bacteria_Firmicutes_Negativicutes_Acidaminococcales_Acidaminococcaceae_Phascolarctobacterium_100
OTU_1408	7.07E-01	6.35E-01	1.66E-01	0.81	1.46	Enterococcus_hirae_97	Bacteria_Firmicutes_99_Bacilli_98_Lactobacillales_97_Enterococcaceae_84_Vagococcus_30
OTU_233	NA	7.61E-01	2.93E-01	0.00	1.96	Elstera_cyanobacteriorum_100	Bacteria_Proteobacteria_Alphaproteobacteria_Rhodospirillales_Rhodospirillaceae_Elstera_100
OTU_30066	7.07E-01	4.60E-01	1.82E-01	1.60	0.98	Erysipelatoclostridium_ramosum_96	Bacteria_Firmicutes_Erysipelotrichia_Erysipelotrichales_Erysipelatoclostridiaceae_Erysipelatoclostridium_100
OTU_34989	9.04E-01	7.31E-01	5.05E-02	1.58	0.61	Bacteroides_thetaiotaomicron_95	Bacteria_Bacteroidetes_Bacteroidia_Bacteroidales_Bacteroidaceae_Bacteroides_88
OTU_47747	7.07E-01	6.76E-01	2.48E-02	1.50	0.09	Veillonella_atypica_97	Bacteria_Firmicutes_Negativicutes_Veillonellales_Veillonellaceae_Veillonella_100
OTU_51719	7.57E-01	6.83E-01	3.96E-01	0.84	1.15	Enterococcus_faecalis_96	Bacteria_Firmicutes_Bacilli_Lactobacillales_99_Enterococcaceae_76_Enterococcus_51

OTU_5303	9.78E-01	8.65E-01	1.65E-02	1.29	0.29	Hungatella_effluvii_96	Bacteria_Firmicutes_Clostridia_Clostridiales_Lachnospiraceae_Hungatella_61
OTU_8811	8.25E-01	6.04E-01	1.92E-02	0.61	1.42	Enterococcus_faecalis_97	Bacteria_Firmicutes_Bacilli_Lactobacillales_99_Enterococcaceae_89_Enterococcus_74
OTU_1982	9.14E-01	6.04E-01	7.37E-01	0.17	0.12	Faecalibacterium_prausnitzii_96	Bacteria_Firmicutes_Clostridia_78_Clostridiales_78_Ruminococcaceae_78_Faecalibacterium_65
OTU_2300	7.86E-01	9.72E-01	6.40E-03	0.41	1.41	Pseudescherichia_vulneris_95	Bacteria_Proteobacteria_Gammaproteobacteria_Enterobacterales_Enterobacteriaceae_Pseudescherichia_51
OTU_27204	7.07E-01	9.70E-01	3.68E-02	0.81	1.38	Pseudescherichia_vulneris_94	Bacteria_Proteobacteria_Gammaproteobacteria_Enterobacterales_Enterobacteriaceae_Pseudescherichia_83
OTU_28142	7.80E-01	6.04E-01	1.68E-02	0.25	1.57	Phascolarctobacterium_faecium_94	Bacteria_Firmicutes_Negativicutes_99_Acidaminococcales_99_Acidaminococcaceae_99_Phascolarctobacterium_98
OTU_29633	7.07E-01	9.41E-01	9.21E-02	0.77	2.07	Bacteroides_nordii_97	Bacteria_Bacteroidetes_Bacteroidia_Bacteroidales_Bacteroidaceae_Bacteroides_100
OTU_3729	9.14E-01	8.02E-01	1.34E-01	0.72	1.17	Shigella_dysenteriae_88	Bacteria_Proteobacteria_73_Gammaproteobacteria_73_Enterobacterales_73_Enterobacteriaceae_73_Pseudescherichia_73
OTU_4205	7.57E-01	6.04E-01	7.03E-03	2.27	0.26	Dialister_pneumosintes_90	Bacteria_Firmicutes_68_Negativicutes_68_Veillonellales_68_Veillonellaceae_68_Dialister_50
OTU_46529	7.91E-01	8.71E-01	4.09E-02	0.76	1.50	Pseudescherichia_vulneris_95	Bacteria_Proteobacteria_98_Gammaproteobacteria_98_Enterobacterales_98_Enterobacteriaceae_97_Pseudescherichia_88
OTU_4682	7.46E-01	6.04E-01	1.69E-01	2.01	0.46	Streptococcus_peroris_96	Bacteria_Firmicutes_Bacilli_Lactobacillales_Streptococcaceae_Streptococcus_100
OTU_4744	7.07E-01	6.04E-01	1.64E-02	2.47	0.17	Parabacteroides_merdae_95	Bacteria_Bacteroidetes_Bacteroidia_Bacteroidales_Porphyromonadaceae_90_Parabacteroides_90
OTU_5595	9.27E-01	8.94E-01	3.82E-05	2.24	0.45	Mediterraneibacter_glycyrrhizinilyticus_100	Bacteria_Firmicutes_Clostridia_Clostridiales_Lachnospiraceae_Mediterraneibacter_100
OTU_6593	7.07E-01	7.51E-01	2.84E-01	2.51	0.34	Kineothrix_alysoides_98	Bacteria_Firmicutes_Clostridia_Clostridiales_Lachnospiraceae_Kineothrix_78
OTU_77824	8.48E-01	6.05E-01	1.10E-02	1.89	0.60	Bacteroides_faecis_96	Bacteria_Bacteroidetes_Bacteroidia_Bacteroidales_Bacteroidaceae_Bacteroides_100
OTU_1347	9.53E-01	6.04E-01	5.27E-03	0.82	2.14	Eisenbergiella_tayi_96	Bacteria_Firmicutes_Clostridia_Clostridiales_Lachnospiraceae_Eisenbergiella_100
OTU_2024	7.07E-01	7.02E-01	6.69E-03	1.51	0.10	Veillonella_dispar_95	Bacteria_Firmicutes_Negativicutes_Veillonellales_Veillonellaceae_Dialister_55
OTU_2480	9.23E-01	6.04E-01	9.61E-01	1.04	1.02	Lachnoclostridium_pacaense_94	Bacteria_Firmicutes_Clostridia_Clostridiales_Lachnospiraceae_84_Clostridium XlVa_32
OTU_403	7.57E-01	6.21E-01	2.28E-01	0.81	0.16	Mitsuokella_jalaludinii_96	Bacteria_Firmicutes_Negativicutes_Selenomonadales_Selenomonadaceae_Mitsuokella_100
OTU_4712	9.78E-01	9.74E-01	8.37E-03	2.30	0.26	Phascolarctobacterium_succinatutens_89	Bacteria_Firmicutes_79_Negativicutes_79_Acidaminococcales_79_Acidaminococcaceae_79_Phascolarctobacterium_79
OTU_13666	7.07E-01	9.25E-01	9.33E-01	0.39	0.43	Kluyvera_georgiana_94	Bacteria_Proteobacteria_Gammaproteobacteria_Enterobacterales_93_Enterobacteriaceae_92_Pseudescherichia_62
OTU_1388	7.57E-01	6.77E-01	2.67E-01	0.35	0.16	Parabacteroides_distasonis_96	Bacteria_Bacteroidetes_Bacteroidia_Bacteroidales_Porphyromonadaceae_Parabacteroides_100
OTU_14917	7.07E-01	8.28E-01	3.49E-01	1.12	1.72	Veillonella_tobetsuensis_94	Bacteria_Firmicutes_Negativicutes_93_Veillonellales_86_Veillonellaceae_86_Veillonella_78
OTU_1595	9.66E-01	8.08E-01	7.16E-01	1.17	1.03	Lachnoclostridium_phytofermentans_90	Bacteria_Firmicutes_72_Clostridia_72_Clostridiales_72_Lachnospiraceae_72_Anaerotaenia_14
OTU_19030	7.07E-01	6.14E-01	4.57E-06	2.49	0.37	Bacteroides_faecis_97	Bacteria_Bacteroidetes_Bacteroidia_Bacteroidales_Bacteroidaceae_Bacteroides_100
OTU_1904	8.94E-01	6.04E-01	1.52E-02	1.74	0.15	Parabacteroides_distasonis_97	Bacteria_Bacteroidetes_Bacteroidia_Bacteroidales_Porphyromonadaceae_98_Parabacteroides_98
OTU_27501	7.46E-01	7.49E-01	6.08E-02	1.86	0.91	Bacteroides_uniformis_97	Bacteria_Bacteroidetes_Bacteroidia_Bacteroidales_Bacteroidaceae_Bacteroides_100
OTU_28714	9.79E-01	6.04E-01	7.84E-01	1.07	1.21	Hungatella_hathewayi_97	Bacteria_Firmicutes_Clostridia_Clostridiales_Lachnospiraceae_Hungatella_90
OTU_33561	9.67E-01	4.60E-01	1.83E-03	2.34	0.15	Bacteroides_caecimuris_95	Bacteria_Bacteroidetes_Bacteroidia_Bacteroidales_Bacteroidaceae_Bacteroides_79
OTU_3582	9.65E-01	8.24E-01	4.01E-02	1.82	0.53	Enterococcus_thailandicus_90	Bacteria_Firmicutes_Clostridia_42_Clostridiales_42_Clostridiaceae 1_42_Anaerobacter_23
OTU_3938	9.87E-01	8.65E-01	7.73E-01	1.29	1.43	Roseburia_inulinivorans_96	Bacteria_Firmicutes_Clostridia_Clostridiales_Lachnospiraceae_Roseburia_57
OTU_7438	7.07E-01	9.69E-01	4.31E-02	0.92	1.52	Escherichia_coli_96	Bacteria_Proteobacteria_Gammaproteobacteria_Enterobacterales_Enterobacteriaceae_96_Escherichia/Shigella_66
OTU_9708	9.64E-01	9.32E-01	9.71E-01	1.06	1.05	Shigella_dysenteriae_95	Bacteria_Proteobacteria_92_Gammaproteobacteria_92_Enterobacterales_92_Enterobacteriaceae_92_Pseudescherichia_82
OTU_13083	8.36E-01	7.89E-01	3.36E-02	0.78	1.54	Pseudescherichia_vulneris_91	Bacteria_Proteobacteria_97_Gammaproteobacteria_97_Enterobacterales_96_Enterobacteriaceae_90_Pseudescherichia_88
OTU_13407	9.72E-01	9.69E-01	3.16E-03	1.88	0.27	Bacteroides_caecimuris_95	Bacteria_Bacteroidetes_Bacteroidia_Bacteroidales_Bacteroidaceae_Bacteroides_89
OTU_1879	9.29E-01	6.61E-01	3.38E-03	1.83	0.42	Sutterella_wadsworthensis_97	Bacteria_Proteobacteria_Betaproteobacteria_Burkholderiales_Sutterellaceae_Sutterella_100
OTU_2008	7.07E-01	6.04E-01	4.50E-01	1.32	0.85	Pseudescherichia_vulneris_89	Bacteria_Proteobacteria_98_Gammaproteobacteria_98_Enterobacterales_98_Enterobacteriaceae_98_Pseudescherichia_96
OTU_3096	7.57E-01	6.68E-01	2.28E-01	1.70	1.03	Enterocloster_clostridioformis_96	Bacteria_Firmicutes_Clostridia_Clostridiales_Lachnospiraceae_Clostridium XlVa_27
OTU_3356	7.07E-01	6.04E-01	2.29E-01	0.79	1.41	Eubacterium_callanderi_95	Bacteria_Firmicutes_Clostridia_Clostridiales_Eubacteriaceae_Pseudoramibacter_60
OTU_39331	9.04E-01	9.97E-01	6.48E-02	0.78	1.33	Shigella_dysenteriae_91	Bacteria_Proteobacteria_89_Gammaproteobacteria_89_Enterobacterales_89_Enterobacteriaceae_89_Pseudescherichia_89
OTU_44288	7.07E-01	6.90E-01	1.16E-03	2.02	0.18	Streptococcus_mitis_98	Bacteria_Firmicutes_Bacilli_Lactobacillales_Streptococcaceae_Streptococcus_100
OTU_4674	8.57E-01	6.35E-01	5.62E-03	2.14	0.36	Streptococcus_cristatus_95	Bacteria_Firmicutes_Bacilli_97_Lactobacillales_97_Streptococcaceae_93_Streptococcus_87
OTU_48086	7.07E-01	9.75E-01	1.66E-01	0.78	1.27	Veillonella_dispar_86	Bacteria_Proteobacteria_85_Gammaproteobacteria_85_Enterobacterales_84_Enterobacteriaceae_83_Pseudescherichia_82
OTU_9669	8.06E-01	9.21E-01	2.07E-02	1.73	0.74	Enterocloster_citroniae_96	Bacteria_Firmicutes_Clostridia_Clostridiales_Lachnospiraceae_Enterocloster_78
OTU_105384	7.07E-01	9.94E-01	5.91E-01	0.24	0.10	Veillonella_tobetsuensis_98	Bacteria_Firmicutes_Negativicutes_Veillonellales_Veillonellaceae_Veillonella_100
OTU_11698	8.25E-01	6.04E-01	1.26E-01	1.22	0.53	Enterocloster_asparagiformis_96	Bacteria_Firmicutes_Clostridia_Clostridiales_Lachnospiraceae_Clostridium XlVa_62
OTU_14573	9.08E-01	8.53E-01	2.76E-02	1.71	0.71	Pseudescherichia_vulneris_97	Bacteria_Proteobacteria_Gammaproteobacteria_Enterobacterales_Enterobacteriaceae_Escherichia/Shigella_53
OTU_1457	7.10E-01	8.85E-01	2.52E-01	0.88	0.33	Sutterella_massiliensis_92	Bacteria_Proteobacteria_98_Betaproteobacteria_98_Burkholderiales_98_Sutterellaceae_96_Sutterella_96
OTU_27375	8.63E-01	8.65E-01	4.94E-02	0.81	1.35	Escherichia_albertii_93	Bacteria_Proteobacteria_97_Gammaproteobacteria_97_Enterobacterales_97_Enterobacteriaceae_95_Pseudescherichia_86
OTU_716	8.51E-01	6.04E-01	9.76E-01	0.53	0.54	Blautia_schinkii_92	Bacteria_Firmicutes_Clostridia_Clostridiales_Lachnospiraceae_Merdimonas_12
OTU_8055	8.48E-01	6.04E-01	5.37E-01	1.26	1.02	Ruminococcus_gnavus_96	Bacteria_Firmicutes_Clostridia_Clostridiales_Lachnospiraceae_97_Dorea_52
OTU_944	7.07E-01	6.04E-01	6.51E-01	1.08	1.62	Enterococcus_dispar_91	Bacteria_Firmicutes_Bacilli_Lactobacillales_86_Carnobacteriaceae_73_Isobaculum_59
OTU_13780	7.10E-01	6.62E-01	1.45E-01	1.34	0.79	Escherichia_albertii_90	Bacteria_Proteobacteria_86_Gammaproteobacteria_86_Enterobacterales_84_Enterobacteriaceae_82_Pseudescherichia_81
OTU_1395	7.07E-01	6.63E-01	3.34E-02	0.53	2.02	Phascolarctobacterium_faecium_90	Bacteria_Firmicutes_Negativicutes_77_Acidaminococcales_77_Acidaminococcaceae_77_Phascolarctobacterium_77
OTU_2296	1.00E+00	6.04E-01	8.33E-01	1.03	1.12	Clostridium_scindens_96	Bacteria_Firmicutes_Clostridia_Clostridiales_Lachnospiraceae_Clostridium XlVa_88
OTU_24502	9.34E-01	6.76E-01	5.28E-02	1.31	0.62	Dorea_longicatena_96	Bacteria_Firmicutes_Clostridia_Clostridiales_Lachnospiraceae_Dorea_64
OTU_3672	7.07E-01	9.46E-01	2.93E-01	1.18	0.86	Clostridium_symbiosum_96	Bacteria_Firmicutes_Clostridia_Clostridiales_Lachnospiraceae_Clostridium XlVa_77
OTU_4370	7.74E-01	6.80E-01	1.25E-01	1.01	0.30	Peptoniphilus_tyrrelliae_97	Bacteria_Firmicutes_Clostridia_Clostridiales_Peptoniphilaceae_Peptoniphilus_100
OTU_48666	7.07E-01	7.42E-01	1.58E-02	0.64	1.44	Enterocloster_clostridioformis_97	Bacteria_Firmicutes_Clostridia_Clostridiales_Lachnospiraceae_Enterocloster_97
OTU_5657	8.06E-01	6.04E-01	5.26E-02	2.26	0.42	Turicibacter_sanguinis_93	Bacteria_Firmicutes_Erysipelotrichia_88_Erysipelotrichales_88_Erysipelotrichaceae_88_Turicibacter_88
OTU_6095	9.87E-01	6.04E-01	2.58E-02	2.17	0.33	Turicibacter_sanguinis_93	Bacteria_Firmicutes_96_Erysipelotrichia_92_Erysipelotrichales_92_Erysipelotrichaceae_92_Turicibacter_92
OTU_8123	8.06E-01	7.99E-01	1.07E-02	1.59	0.63	Veillonella_atypica_95	Bacteria_Firmicutes_Negativicutes_Veillonellales_Veillonellaceae_Veillonella_100
OTU_11663	7.07E-01	6.50E-01	9.51E-01	1.29	1.27	Hungatella_effluvii_97	Bacteria_Firmicutes_Clostridia_Clostridiales_Lachnospiraceae_Hungatella_98
OTU_12289	8.06E-01	9.34E-01	4.04E-03	2.02	0.28	Bacteroides_stercoris_97	Bacteria_Bacteroidetes_Bacteroidia_Bacteroidales_Bacteroidaceae_Bacteroides_95
OTU_14609	7.07E-01	9.65E-01	2.07E-02	1.31	0.53	Bacteroides_kribbi_96	Bacteria_Bacteroidetes_Bacteroidia_Bacteroidales_Bacteroidaceae_Bacteroides_100
OTU_15738	8.23E-01	8.36E-01	4.89E-01	1.49	1.15	Roseburia_inulinivorans_97	Bacteria_Firmicutes_Clostridia_Clostridiales_Lachnospiraceae_Roseburia_57
OTU_2120	7.59E-01	6.63E-01	9.78E-01	1.24	1.23	Bacteroides_thetaiotaomicron_98	Bacteria_Bacteroidetes_Bacteroidia_99_Bacteroidales_99_Bacteroidaceae_99_Bacteroides_89

OTU_21558	8.85E-01	7.30E-01	3.61E-03	1.56	0.35	Phocaeicola_vulgatus_97	Bacteria_Bacteroidetes_Bacteroidia_Bacteroidales_Bacteroidaceae_Phocaeicola_54
OTU_2200	1.00E+00	8.28E-01	3.33E-02	0.67	1.35	Gibbsiella_greigii_87	Bacteria_Proteobacteria_90_Gammaproteobacteria_90_Enterobacterales_89_Enterobacteriaceae_89_Pseudescherichia_89
OTU_370	9.06E-01	8.48E-01	9.78E-01	0.45	0.46	Adlercreutzia_muris_95	Bacteria_Actinobacteria_99_Coriobacteriia_99_Eggerthellales_84_Eggerthellaceae_84_Adlercreutzia_79
OTU_58537	7.85E-01	9.61E-01	9.94E-02	0.81	1.45	Hungatella_hathewayi_97	Bacteria_Firmicutes_Clostridia_Clostridiales_Lachnospiraceae_Hungatella_69
OTU_6687	7.57E-01	7.31E-01	9.85E-01	1.01	1.00	Flavonifractor_plautii_95	Bacteria_Firmicutes_Clostridia_Clostridiales_Ruminococcaceae_Flavonifractor_100
OTU_724	8.67E-01	9.25E-01	6.76E-01	0.18	0.11	Streptococcus_thermophilus_100	Bacteria_Firmicutes_75_Bacilli_72_Lactobacillales_71_Enterococcaceae_30_Pilibacter_25
OTU_8729	8.82E-01	7.79E-01	2.80E-02	0.78	1.66	Enterocloster_clostridioformis_96	Bacteria_Firmicutes_Clostridia_Clostridiales_Lachnospiraceae_Enterocloster_98
OTU_1168	7.07E-01	8.14E-01	3.03E-02	2.12	0.53	Enterococcus_faecalis_97	Bacteria_Firmicutes_Bacilli_Lactobacillales_Enterococcaceae_82_Enterococcus_61
OTU_1192	9.66E-01	9.85E-01	3.10E-01	0.19	0.36	Phocaeicola_coprophilus_90	Bacteria_Bacteroidetes_91_Bacteroidia_91_Bacteroidales_91_Bacteroidaceae_91_Phocaeicola_85
OTU_1229	9.92E-01	6.66E-01	8.27E-03	0.48	1.48	Phocaeicola_coprophilus_95	Bacteria_Bacteroidetes_73_Bacteroidia_73_Bacteroidales_73_Bacteroidaceae_73_Phocaeicola_51
OTU_1363	9.38E-01	9.42E-01	6.19E-03	1.51	0.38	Pseudescherichia_vulneris_97	Bacteria_Proteobacteria_75_Gammaproteobacteria_75_Enterobacterales_75_Enterobacteriaceae_75_Pseudescherichia_57
OTU_13942	7.07E-01	8.73E-01	1.31E-02	1.71	0.35	Bacteroides_caecimuris_96	Bacteria_Bacteroidetes_Bacteroidia_Bacteroidales_Bacteroidaceae_Bacteroides_69
OTU_1405	9.67E-01	9.41E-01	7.59E-01	1.04	1.14	Pseudescherichia_vulneris_99	Bacteria_Proteobacteria_Gammaproteobacteria_Enterobacterales_Enterobacteriaceae_Escherichia/Shigella_99
OTU_16806	7.12E-01	7.31E-01	5.27E-01	0.58	0.35	Veillonella_rogosae_97	Bacteria_Firmicutes_Negativicutes_Veillonellales_Veillonellaceae_Veillonella_100
OTU_2063	7.11E-01	6.72E-01	9.22E-02	1.19	0.28	Peptoniphilus_duerdenii_96	Bacteria_Firmicutes_Clostridia_Clostridiales_Peptoniphilaceae_93_Peptoniphilus_92
OTU_2482	9.14E-01	8.42E-01	1.36E-01	0.80	1.40	Hungatella_effluvii_96	Bacteria_Firmicutes_Clostridia_Clostridiales_Lachnospiraceae_Hungatella_99
OTU_2870	9.59E-01	6.59E-01	1.18E-01	0.71	1.35	Falcatimonas_natans_90	Bacteria_Firmicutes_Clostridia_68_Clostridiales_68_Lachnospiraceae_68_Falcatimonas_20
OTU_29225	9.87E-01	9.54E-01	7.98E-01	1.10	1.20	Enterococcus_hirae_96	Bacteria_Firmicutes_99_Bacilli_99_Lactobacillales_95_Enterococcaceae_78_Vagococcus_38
OTU_3559	7.62E-01	8.36E-01	7.35E-01	1.11	0.95	Enterocloster_clostridioformis_97	Bacteria_Firmicutes_Clostridia_Clostridiales_Lachnospiraceae_Enterocloster_87
OTU_3707	8.82E-01	8.09E-01	3.59E-02	1.02	0.35	Veillonella_dispar_100	Bacteria_Firmicutes_78_Negativicutes_78_Selenomonadales_30_Sporomusaceae_30_Anaerosinus_21
OTU_4725	8.97E-01	7.55E-01	1.64E-02	1.61	0.30	Listeria_grayi_91	Bacteria_Firmicutes_Bacilli_53_Lactobacillales_45_Carnobacteriaceae_40_Isobaculum_30
OTU_647	9.87E-01	6.04E-01	5.05E-02	1.27	0.09	Limosilactobacillus_secaliphilus_97	Bacteria_Firmicutes_Bacilli_Lactobacillales_Lactobacillaceae_Limosilactobacillus_56
OTU_7426	7.07E-01	7.70E-01	2.58E-01	0.75	1.19	Eubacterium_callanderi_98	Bacteria_Firmicutes_83_Clostridia_83_Clostridiales_83_Eubacteriaceae_81_Pseudoramibacter_49
OTU_927	9.78E-01	6.14E-01	1.73E-02	0.51	1.85	Eubacterium_callanderi_95	Bacteria_Firmicutes_Clostridia_Clostridiales_Eubacteriaceae_Eubacterium_41
OTU_92918	7.91E-01	9.37E-01	1.21E-02	2.72	0.14	Bacteroides_xylanisolvens_98	Bacteria_Bacteroidetes_Bacteroidia_Bacteroidales_Bacteroidaceae_Bacteroides_100
OTU_970	9.99E-01	7.80E-01	8.85E-02	0.47	0.97	Gibbsiella_greigii_90	Bacteria_Proteobacteria_Gammaproteobacteria_50_Enterobacterales_50_Enterobacteriaceae_50_Pseudescherichia_42
OTU_9959	7.19E-01	6.04E-01	1.80E-01	0.89	0.43	Collinsella_aerofaciens_97	Bacteria_Actinobacteria_Coriobacteriia_Coriobacteriales_Coriobacteriaceae_Collinsella_100
OTU_13073	9.87E-01	9.61E-01	2.43E-03	2.30	0.49	Clostridium_perfringens_97	Bacteria_Firmicutes_74_Clostridia_74_Clostridiales_74_Clostridiaceae 1_74_Sarcina_30
OTU_1429	9.65E-01	8.41E-01	2.45E-02	2.31	0.37	Clostridium_perfringens_96	Bacteria_Firmicutes_Clostridia_Clostridiales_Clostridiaceae 1_63_Sarcina_34
OTU_15169	9.60E-01	8.68E-01	2.49E-01	0.69	1.18	Lachnoclostridium_pacaense_96	Bacteria_Firmicutes_Clostridia_Clostridiales_Lachnospiraceae_Enterocloster_47
OTU_1872	7.07E-01	6.65E-01	4.92E-03	2.06	0.12	Bacteroides_uniformis_96	Bacteria_Bacteroidetes_99_Bacteroidia_99_Bacteroidales_99_Bacteroidaceae_92_Mediterranea_71
OTU_1911	9.46E-01	8.51E-01	9.43E-01	0.95	0.98	Enterococcus_devriesei_89	Bacteria_Firmicutes_97_Bacilli_52_Lactobacillales_44_Carnobacteriaceae_34_Catellicoccus_18
OTU_33595	1.00E+00	9.74E-01	8.35E-03	1.60	0.65	Bacteroides_acidifaciens_94	Bacteria_Bacteroidetes_99_Bacteroidia_99_Bacteroidales_99_Bacteroidaceae_99_Bacteroides_93
OTU_3459	7.73E-01	6.83E-01	1.95E-01	1.27	0.60	Fusobacterium_equinum_91	Bacteria_Fusobacteria_96_Fusobacteriia_96_Fusobacteriales_96_Fusobacteriaceae_85_Cetobacterium_44
OTU_4652	7.91E-01	4.60E-01	1.83E-02	1.72	0.58	Bacteroides_uniformis_96	Bacteria_Bacteroidetes_Bacteroidia_Bacteroidales_Bacteroidaceae_Bacteroides_100
OTU_49888	7.07E-01	6.47E-01	1.95E-01	0.84	1.32	Clostridium_symbiosum_96	Bacteria_Firmicutes_Clostridia_Clostridiales_Lachnospiraceae_Clostridium XlVa_63
OTU_5027	7.91E-01	6.04E-01	4.12E-02	0.64	1.28	Lachnoclostridium_pacaense_92	Bacteria_Firmicutes_Clostridia_96_Clostridiales_96_Lachnospiraceae_96_Schaedlerella_23
OTU_5739	8.82E-01	9.80E-01	9.40E-02	1.40	0.79	Peptoniphilus_tyrrelliae_92	Bacteria_Firmicutes_Clostridia_Clostridiales_Lachnospiraceae_58_Falcatimonas_17
OTU_14985	9.60E-01	8.85E-01	3.89E-01	1.08	1.37	Bacteroides_faecis_96	Bacteria_Bacteroidetes_Bacteroidia_Bacteroidales_Bacteroidaceae_Bacteroides_100
OTU_16074	9.75E-01	6.57E-01	3.46E-01	0.91	1.23	Pseudescherichia_vulneris_95	Bacteria_Proteobacteria_Gammaproteobacteria_Enterobacterales_Enterobacteriaceae_Pseudescherichia_58
OTU_2165	7.07E-01	8.14E-01	2.67E-01	1.55	1.09	Roseburia_inulinivorans_97	Bacteria_Firmicutes_Clostridia_Clostridiales_Lachnospiraceae_Roseburia_83
OTU_2324	9.59E-01	7.87E-01	7.57E-03	0.23	1.30	Phocaeicola_coprophilus_99	Bacteria_Bacteroidetes_99_Bacteroidia_99_Bacteroidales_99_Bacteroidaceae_97_Phocaeicola_72
OTU_411	7.07E-01	6.04E-01	3.75E-01	2.07	0.40	Intestinimonas_butyriciproducens_97	Bacteria_Firmicutes_Clostridia_Clostridiales_Ruminococcaceae_Intestinimonas_100
OTU_4401	7.60E-01	6.04E-01	2.01E-01	1.12	0.57	Kineothrix_alysoides_93	Bacteria_Firmicutes_99_Clostridia_99_Clostridiales_99_Lachnospiraceae_99_Clostridium XlVa_40
OTU_4547	9.72E-01	7.34E-01	1.97E-01	1.71	0.72	Enterocloster_clostridioformis_94	Bacteria_Firmicutes_Clostridia_Clostridiales_Lachnospiraceae_99_Coprococcus_37
OTU_4887	7.57E-01	6.04E-01	3.89E-01	0.84	1.25	Clostridium_symbiosum_95	Bacteria_Firmicutes_Clostridia_Clostridiales_Lachnospiraceae_Coprococcus_66
OTU_55040	7.46E-01	8.97E-01	1.01E-02	0.51	1.40	Macrococcus_goetzii_87	Bacteria_Firmicutes_97_Bacilli_50_Lactobacillales_41_Carnobacteriaceae_39_Catellicoccus_35
OTU_7836	9.60E-01	6.76E-01	3.76E-01	0.87	1.18	Escherichia_coli_92	Bacteria_Proteobacteria_97_Gammaproteobacteria_97_Enterobacterales_97_Enterobacteriaceae_97_Pseudescherichia_96
OTU_86317	7.07E-01	9.13E-01	6.22E-01	0.54	0.72	Clostridium_perfringens_96	Bacteria_Firmicutes_Clostridia_97_Clostridiales_97_Clostridiaceae 1_97_Sarcina_17
OTU_89975	1.00E+00	8.65E-01	5.91E-02	0.39	1.54	Phocaeicola_coprophilus_97	Bacteria_Bacteroidetes_Bacteroidia_Bacteroidales_Bacteroidaceae_Phocaeicola_100
OTU_9677	9.14E-01	6.04E-01	4.43E-03	2.02	0.42	Anaerotignum_faecicola_95	Bacteria_Firmicutes_Clostridia_Clostridiales_Lachnospiraceae_94_Anaerotignum_92
OTU_1203	9.38E-01	7.49E-01	8.00E-02	0.40	0.12	Phocaeicola_vulgatus_100	Bacteria_Bacteroidetes_Bacteroidia_Bacteroidales_Bacteroidaceae_Phocaeicola_80
OTU_12151	7.07E-01	6.83E-01	5.73E-01	0.42	0.29	Bacteroides_stercoris_96	Bacteria_Bacteroidetes_Bacteroidia_Bacteroidales_Bacteroidaceae_Bacteroides_69
OTU_1262	7.46E-01	6.14E-01	7.66E-02	2.09	0.27	Prevotella_copri_96	Bacteria_Bacteroidetes_Bacteroidia_Bacteroidales_Prevotellaceae_96_Prevotella_96
OTU_1538	NA	9.54E-01	6.52E-02	0.00	1.12	Methylophilus_methylotrophus_95	Bacteria_Proteobacteria_Betaproteobacteria_Nitrosomonadales_81_Methylophilaceae_72_Methylophilus_33
OTU_17031	8.74E-01	9.94E-01	1.01E-02	0.73	1.66	Blautia_faecicola_91	Bacteria_Firmicutes_Clostridia_61_Clostridiales_61_Lachnospiraceae_61_Lachnobacterium_8
OTU_1749	9.87E-01	6.04E-01	1.44E-01	0.87	1.84	Paraclostridium_bifermentans_98	Bacteria_Firmicutes_Clostridia_72_Clostridiales_72_Peptostreptococcaceae_72_Paraclostridium_72
OTU_2307	7.91E-01	6.56E-01	5.02E-01	0.93	1.20	Enterocloster_citroniae_98	Bacteria_Firmicutes_Clostridia_Clostridiales_Lachnospiraceae_Enterocloster_67
OTU_2335	1.00E+00	6.63E-01	3.24E-02	2.35	0.35	Ligilactobacillus_apodemi_96	Bacteria_Firmicutes_Bacilli_Lactobacillales_Enterococcaceae_49_Melissococcus_24
OTU_2561	8.60E-01	6.04E-01	8.29E-01	1.07	1.24	Dorea_longicatena_97	Bacteria_Firmicutes_Clostridia_Clostridiales_Lachnospiraceae_Dorea_100
OTU_34528	7.93E-01	9.06E-01	6.51E-01	0.89	1.07	Coprococcus_comes_97	Bacteria_Firmicutes_Clostridia_Clostridiales_Lachnospiraceae_Coprococcus_65
OTU_38349	8.16E-01	7.31E-01	1.19E-02	1.67	0.44	Bacteroides_uniformis_97	Bacteria_Bacteroidetes_Bacteroidia_Bacteroidales_Bacteroidaceae_Bacteroides_100
OTU_4780	9.54E-01	6.04E-01	6.75E-04	1.56	0.20	Parabacteroides_distasonis_97	Bacteria_Firmicutes_51_Clostridia_51_Clostridiales_51_Lachnospiraceae_51_Catonella_26
OTU_6086	7.57E-01	6.06E-01	6.96E-01	1.45	1.05	Roseburia_intestinalis_99	Bacteria_Firmicutes_Clostridia_Clostridiales_Lachnospiraceae_Roseburia_100
OTU_7715	9.97E-01	5.09E-01	7.87E-01	0.98	1.07	Enterocloster_clostridioformis_96	Bacteria_Firmicutes_79_Clostridia_79_Clostridiales_79_Lachnospiraceae_79_Enterocloster_37
OTU_8357	7.07E-01	6.04E-01	1.48E-01	0.77	1.61	Faecalibacterium_prausnitzii_94	Bacteria_Firmicutes_Clostridia_Clostridiales_Ruminococcaceae_Faecalibacterium_69
OTU_911	1.00E+00	6.04E-01	3.49E-02	1.76	0.41	Phascolarctobacterium_succinatutens_98	Bacteria_Firmicutes_Negativicutes_99_Acidaminococcales_99_Acidaminococcaceae_99_Phascolarctobacterium_99

OTU_919	8.16E-01	8.11E-01	4.18E-03	0.27	2.27	Phascolarctobacterium_faecium_95	Bacteria_Firmicutes_Negativicutes_Acidaminococcales_Acidaminococcaceae_Phascolarctobacterium_100
OTU_947	7.07E-01	6.04E-01	4.18E-01	0.63	0.23	Streptococcus_salivarius_97	Bacteria_Firmicutes_Bacilli_Lactobacillales_Streptococcaceae_99_Streptococcus_99
OTU_1007	7.07E-01	8.83E-01	4.80E-02	1.90	0.14	Fusobacterium_mortiferum_95	Bacteria_Fusobacteria_86_Fusobacteriia_86_Fusobacteriales_86_Fusobacteriaceae_84_Cetobacterium_58
OTU_10132	9.06E-01	9.32E-01	9.37E-01	1.00	1.03	Enterococcus_thailandicus_96	Bacteria_Firmicutes_Bacilli_99_Lactobacillales_98_Carnobacteriaceae_45_Isobaculum_33
OTU_1016	7.07E-01	8.17E-01	1.65E-01	0.31	2.11	Coprococcus_comes_94	Bacteria_Firmicutes_Clostridia_Clostridiales_Lachnospiraceae_Bariatricus_67
OTU_112248	9.84E-01	8.73E-01	7.39E-03	0.32	2.11	Bacteroides_kribbi_97	Bacteria_Bacteroidetes_Bacteroidia_Bacteroidales_Bacteroidaceae_Bacteroides_100
OTU_11288	7.14E-01	8.10E-01	3.07E-03	2.17	0.22	Bacteroides_uniformis_97	Bacteria_Bacteroidetes_Bacteroidia_Bacteroidales_Bacteroidaceae_Bacteroides_99
OTU_11368	9.59E-01	9.09E-01	8.75E-02	1.06	0.36	Bacteroides_faecis_95	Bacteria_Bacteroidetes_Bacteroidia_Bacteroidales_Bacteroidaceae_Bacteroides_76
OTU_12654	8.17E-01	8.48E-01	1.15E-03	1.99	0.17	Bacteroides_caecimuris_95	Bacteria_Bacteroidetes_Bacteroidia_Bacteroidales_Bacteroidaceae_Bacteroides_86
OTU_2123	7.07E-01	7.40E-01	1.63E-02	0.56	1.74	Veillonella_tobetsuensis_92	Bacteria_Firmicutes_Clostridia_56_Clostridiales_56_Lachnospiraceae_56_Hungatella_54
OTU_2202	9.16E-01	6.16E-01	1.22E-03	1.36	0.07	Bacteroides_timonensis_93	Bacteria_Bacteroidetes_Bacteroidia_Bacteroidales_Bacteroidaceae_77_Bacteroides_75
OTU_2479	1.00E+00	9.02E-01	2.03E-02	1.83	0.86	Clostridium_symbiosum_98	Bacteria_Firmicutes_Clostridia_Clostridiales_Lachnospiraceae_Clostridium XlVa_66
OTU_24940	8.25E-01	8.73E-01	9.89E-02	1.27	0.74	Clostridium_symbiosum_95	Bacteria_Firmicutes_Clostridia_Clostridiales_Lachnospiraceae_Clostridium XlVa_67
OTU_3928	9.38E-01	4.60E-01	2.26E-01	0.97	1.56	Paraclostridium_benzoelyticum_96	Bacteria_Firmicutes_Clostridia_97_Clostridiales_97_Peptostreptococcaceae_97_Paraclostridium_90
OTU_51274	8.23E-01	9.14E-01	9.20E-03	0.36	1.49	Shigella_dysenteriae_95	Bacteria_Proteobacteria_Gammaproteobacteria_Enterobacterales_Enterobacteriaceae_Pseudescherichia_66
OTU_67151	1.00E+00	8.51E-01	1.61E-02	2.08	0.38	Pseudescherichia_vulneris_90	Bacteria_Proteobacteria_Betaproteobacteria_67_Burkholderiales_65_Sutterellaceae_65_Sutterella_65
OTU_67762	7.07E-01	6.44E-01	4.62E-01	1.22	0.51	Enterobacter_hormaechei_99	Bacteria_Proteobacteria_Gammaproteobacteria_Enterobacterales_Enterobacteriaceae_85_Enterobacter_35
OTU_696	8.54E-01	9.89E-01	4.18E-01	0.37	0.16	Megasphaera_elsdenii_96	Bacteria_Firmicutes_Negativicutes_Veillonellales_Veillonellaceae_Megasphaera_100
OTU_7337	9.87E-01	6.04E-01	3.75E-01	1.03	1.39	Hungatella_hathewayi_95	Bacteria_Firmicutes_Clostridia_Clostridiales_Lachnospiraceae_90_Hungatella_84
OTU_838	7.07E-01	9.69E-01	1.15E-03	1.76	0.28	Erysipelatoclostridium_ramosum_92	Bacteria_Firmicutes_Erysipelotrichia_Erysipelotrichales_Erysipelatoclostridiaceae_Erysipelatoclostridium_100
OTU_9010	9.38E-01	7.02E-01	6.82E-02	0.87	0.28	Ruminococcus_lactaris_99	Bacteria_Firmicutes_Clostridia_Clostridiales_Lachnospiraceae_Ruminococcus2_85
OTU_10149	7.10E-01	6.04E-01	3.09E-01	1.50	0.27	Veillonella_rogosae_97	Bacteria_Firmicutes_Negativicutes_Veillonellales_Veillonellaceae_Veillonella_100
OTU_1029	7.50E-01	6.24E-01	5.00E-02	1.73	0.28	Cronobacter_turicensis_91	Bacteria_Proteobacteria_93_Gammaproteobacteria_93_Enterobacterales_93_Enterobacteriaceae_93_Pseudescherichia_89
OTU_118519	NA	6.76E-01	2.30E-02	0.00	1.57	Pseudescherichia_vulneris_97	Bacteria_Proteobacteria_Gammaproteobacteria_Enterobacterales_Enterobacteriaceae_Escherichia/Shigella_63
OTU_130282	NA	6.04E-01	3.74E-02	0.00	2.57	Bacteroides_kribbi_97	Bacteria_Bacteroidetes_Bacteroidia_Bacteroidales_Bacteroidaceae_Bacteroides_100
OTU_1462	7.57E-01	6.04E-01	3.66E-01	0.45	1.40	Clostridium_polysaccharolyticum_92	Bacteria_Firmicutes_Clostridia_Clostridiales_Ruminococcaceae_58_Flintibacter_52
OTU_15791	9.37E-01	7.01E-01	1.99E-02	2.08	0.39	Bacteroides_xylanisolvens_95	Bacteria_Bacteroidetes_Bacteroidia_Bacteroidales_Bacteroidaceae_Bacteroides_66
OTU_17847	7.07E-01	6.57E-01	7.36E-01	1.17	1.03	Pseudescherichia_vulneris_88	Bacteria_Proteobacteria_99_Gammaproteobacteria_99_Enterobacterales_99_Enterobacteriaceae_99_Pseudescherichia_97
OTU_2987	7.07E-01	8.54E-01	9.41E-01	1.13	1.17	Bacteroides_ovatus_97	Bacteria_Bacteroidetes_Bacteroidia_Bacteroidales_Bacteroidaceae_Bacteroides_100
OTU_3020	7.07E-01	8.68E-01	4.11E-02	1.80	0.50	Coprococcus_comes_96	Bacteria_Firmicutes_Clostridia_Clostridiales_Lachnospiraceae_Coprococcus_64
OTU_3031	8.67E-01	7.36E-01	3.27E-01	0.75	1.29	Enterococcus_hirae_92	Bacteria_Firmicutes_99_Clostridia_59_Clostridiales_59_Peptostreptococcaceae_58_Paraclostridium_55
OTU_35803	7.75E-01	6.04E-01	6.93E-02	0.49	0.95	Enterococcus_faecalis_98	Bacteria_Firmicutes_Bacilli_Lactobacillales_Enterococcaceae_99_Enterococcus_75
OTU_395	NA	9.34E-01	1.31E-01	0.00	1.69	Azospirillum_lipoferum_98	Bacteria_Proteobacteria_Alphaproteobacteria_Rhodospirillales_Azospirillaceae_Azospirillum_100
OTU_44830	8.06E-01	6.13E-01	1.14E-01	1.94	0.37	Veillonella_tobetsuensis_97	Bacteria_Firmicutes_Negativicutes_Veillonellales_Veillonellaceae_Veillonella_100
OTU_4699	9.14E-01	6.43E-01	5.11E-01	1.20	0.96	Bacteroides_cellulosilyticus_87	Bacteria_Firmicutes_55_Clostridia_55_Clostridiales_55_Lachnospiraceae_54_Merdimonas_20
OTU_57997	9.92E-01	6.14E-01	1.22E-01	0.50	1.02	Enterococcus_faecalis_98	Bacteria_Firmicutes_Bacilli_Lactobacillales_Enterococcaceae_95_Enterococcus_60
OTU_63171	8.25E-01	8.14E-01	8.60E-01	0.29	0.25	Phocaeicola_coprophilus_96	Bacteria_Bacteroidetes_Bacteroidia_Bacteroidales_Bacteroidaceae_Phocaeicola_100
OTU_7577	8.99E-01	9.73E-01	6.15E-01	1.04	1.25	Enterocloster_asparagiformis_96	Bacteria_Firmicutes_Clostridia_Clostridiales_Lachnospiraceae_99_Enterocloster_87
OTU_77758	7.07E-01	9.13E-01	1.74E-02	2.50	0.07	Bacteroides_stercoris_97	Bacteria_Bacteroidetes_Bacteroidia_Bacteroidales_Bacteroidaceae_Bacteroides_100
OTU_1699	9.04E-01	6.04E-01	6.60E-01	1.04	1.26	Murimonas_intestini_91	Bacteria_Firmicutes_Clostridia_Clostridiales_Ruminococcaceae_95_Dysosmobacter_95
OTU_1764	9.14E-01	6.73E-01	1.37E-02	2.23	0.54	Bacteroides_ovatus_97	Bacteria_Bacteroidetes_Bacteroidia_Bacteroidales_Bacteroidaceae_Bacteroides_100
OTU_2690	9.92E-01	7.79E-01	1.46E-02	1.90	0.55	Clostridium_tarantellae_92	Bacteria_Firmicutes_Clostridia_Clostridiales_Clostridiaceae 1_67_Anaerobacter_23
OTU_3367	7.07E-01	8.28E-01	4.87E-02	2.12	0.55	Bacteroides_xylanisolvens_98	Bacteria_Bacteroidetes_Bacteroidia_Bacteroidales_Bacteroidaceae_Bacteroides_100
OTU_3911	8.45E-01	6.04E-01	2.43E-01	0.56	1.23	Parabacteroides_chongii_96	Bacteria_Bacteroidetes_Bacteroidia_Bacteroidales_Porphyromonadaceae_99_Parabacteroides_99
OTU_396	NA	6.61E-01	9.74E-02	0.00	1.75	Aquimonas_voraii_97	Bacteria_Proteobacteria_Gammaproteobacteria_Xanthomonadales_99_Rhodanobacteraceae_84_Aquimonas_84
OTU_46588	7.91E-01	6.04E-01	1.82E-03	2.50	0.07	Bacteroides_finegoldii_97	Bacteria_Bacteroidetes_Bacteroidia_Bacteroidales_Bacteroidaceae_Bacteroides_100
OTU_48863	7.07E-01	6.72E-01	2.35E-02	1.13	0.09	Veillonella_atypica_97	Bacteria_Firmicutes_Negativicutes_Veillonellales_Veillonellaceae_Veillonella_100
OTU_6084	7.94E-01	7.83E-01	4.66E-01	1.12	1.44	Veillonella_dispar_95	Bacteria_Firmicutes_Negativicutes_Veillonellales_94_Veillonellaceae_94_Veillonella_94
OTU_71377	7.07E-01	7.91E-01	4.67E-02	1.19	0.35	Bacteroides_xylanisolvens_95	Bacteria_Bacteroidetes_Bacteroidia_Bacteroidales_Bacteroidaceae_Phocaeicola_98
OTU_8514	8.85E-01	6.04E-01	9.65E-01	1.13	1.15	Acidaminococcus_fermentans_93	Bacteria_Firmicutes_Negativicutes_95_Acidaminococcales_92_Acidaminococcaceae_92_Acidaminococcus_92
OTU_892	9.80E-01	6.04E-01	1.66E-01	2.02	0.66	Pseudescherichia_vulneris_90	Bacteria_Proteobacteria_42_Gammaproteobacteria_42_Enterobacterales_42_Enterobacteriaceae_42_Pseudescherichia_30
OTU_9288	7.07E-01	6.04E-01	5.42E-01	1.00	0.68	Cronobacter_dublinensis_98	Bacteria_Proteobacteria_Gammaproteobacteria_Enterobacterales_Enterobacteriaceae_Cronobacter_54
OTU_980	7.07E-01	6.05E-01	2.27E-01	1.34	0.38	Oscillibacter_valericigenes_95	Bacteria_Firmicutes_Clostridia_Clostridiales_Ruminococcaceae_Oscillibacter_98
OTU_1019	7.57E-01	6.63E-01	4.67E-02	0.33	2.16	Paraclostridium_bifermentans_100	Bacteria_Firmicutes_94_Clostridia_94_Clostridiales_94_Peptostreptococcaceae_94_Paraclostridium_92
OTU_107861	7.07E-01	NA	3.17E-01	2.24	0.00	Veillonella_rogosae_98	Bacteria_Firmicutes_Negativicutes_Veillonellales_Veillonellaceae_Veillonella_100
OTU_1442	7.07E-01	7.88E-01	3.45E-02	1.95	0.59	Roseburia_inulinivorans_97	Bacteria_Firmicutes_Clostridia_Clostridiales_Lachnospiraceae_Roseburia_99
OTU_15094	7.07E-01	6.10E-01	1.64E-01	0.52	0.23	Bifidobacterium_callitrichidarum_93	Bacteria_Actinobacteria_86_Actinobacteria_86_Bifidobacteriales_86_Bifidobacteriaceae_86_Bifidobacterium_53
OTU_1931	9.87E-01	6.04E-01	2.54E-01	1.54	0.85	Bacteroides_fragilis_99	Bacteria_Bacteroidetes_99_Bacteroidia_97_Bacteroidales_97_Bacteroidaceae_90_Bacteroides_46
OTU_1951	7.07E-01	6.05E-01	2.43E-01	0.24	0.61	Clostridium_tepidum_98	Bacteria_Firmicutes_Clostridia_81_Clostridiales_81_Clostridiaceae 1_81_Haloimpatiens_27
OTU_27653	7.57E-01	8.89E-01	1.06E-01	1.73	0.76	Prevotella_histicola_92	Bacteria_Bacteroidetes_Bacteroidia_Bacteroidales_Prevotellaceae_Prevotella_99
OTU_31961	8.97E-01	6.04E-01	3.52E-04	1.71	0.45	Bacteroides_thetaiotaomicron_95	Bacteria_Bacteroidetes_Bacteroidia_Bacteroidales_Bacteroidaceae_Bacteroides_100
OTU_34234	7.73E-01	9.51E-01	2.01E-03	1.83	0.17	Streptococcus_gordonii_98	Bacteria_Firmicutes_Bacilli_Lactobacillales_Streptococcaceae_Streptococcus_99
OTU_36311	9.14E-01	6.04E-01	3.51E-01	0.55	0.92	Enterococcus_faecalis_98	Bacteria_Firmicutes_Bacilli_Lactobacillales_Enterococcaceae_94_Enterococcus_74
OTU_428	9.59E-01	6.04E-01	8.58E-01	0.88	0.76	Peptoniphilus_lacrimalis_100	Bacteria_Firmicutes_Clostridia_Clostridiales_Peptoniphilaceae_Peptoniphilus_100
OTU_4827	9.67E-01	6.39E-01	5.78E-02	0.54	1.66	Sellimonas_intestinalis_97	Bacteria_Firmicutes_Clostridia_Clostridiales_Lachnospiraceae_Sellimonas_100
OTU_67600	1.00E+00	8.94E-01	4.65E-03	1.62	0.21	Streptococcus_mitis_98	Bacteria_Firmicutes_Bacilli_Lactobacillales_Streptococcaceae_99_Streptococcus_99
OTU_684	7.07E-01	6.04E-01	4.30E-02	2.48	0.09	Clostridium_quinii_91	Bacteria_Proteobacteria_47_Gammaproteobacteria_47_Enterobacterales_46_Enterobacteriaceae_46_Pseudescherichia_43

OTU_7311	7.57E-01	8.28E-01	8.12E-01	0.88	0.80	Pseudescherichia_vulneris_89	Bacteria_Firmicutes_79_Clostridia_79_Clostridiales_79_Lachnospiraceae_79_Enterocloster_33
OTU_8067	8.60E-01	8.37E-01	6.65E-02	0.96	1.73	Bacteroides_kribbi_96	Bacteria_Bacteroidetes_Bacteroidia_Bacteroidales_Bacteroidaceae_Bacteroides_100
OTU_81088	7.07E-01	8.37E-01	7.37E-02	2.57	0.14	Bacteroides_stercoris_97	Bacteria_Bacteroidetes_Bacteroidia_Bacteroidales_Bacteroidaceae_Bacteroides_99
OTU_10347	9.64E-01	7.29E-01	2.92E-04	1.88	0.38	Bacteroides_uniformis_90	Bacteria_Bacteroidetes_79_Bacteroidia_79_Bacteroidales_79_Bacteroidaceae_78_Phocaeicola_25
OTU_11474	9.05E-01	6.04E-01	9.29E-01	1.11	1.06	Enterocloster_citroniae_97	Bacteria_Firmicutes_Clostridia_Clostridiales_Lachnospiraceae_Enterocloster_69
OTU_12641	7.07E-01	6.04E-01	1.09E-05	2.03	0.38	Bacteroides_xylanisolvens_98	Bacteria_Bacteroidetes_Bacteroidia_Bacteroidales_Bacteroidaceae_Bacteroides_100
OTU_1354	7.07E-01	8.14E-01	2.82E-02	1.12	0.08	Bacteroides_uniformis_97	Bacteria_Bacteroidetes_Bacteroidia_Bacteroidales_Bacteroidaceae_Bacteroides_99
OTU_1510	1.00E+00	9.80E-01	6.33E-01	0.08	0.12	Phocaeicola_plebeius_98	Bacteria_Bacteroidetes_99_Bacteroidia_99_Bacteroidales_99_Bacteroidaceae_99_Phocaeicola_83
OTU_1626	9.31E-01	7.02E-01	3.37E-01	0.95	1.33	Enterococcus_thailandicus_94	Bacteria_Firmicutes_Bacilli_98_Lactobacillales_96_Enterococcaceae_68_Vagococcus_28
OTU_1670	9.08E-01	8.51E-01	2.25E-01	0.17	0.43	Anaerobutyricum_hallii_96	Bacteria_Firmicutes_Clostridia_Clostridiales_Lachnospiraceae_Anaerobutyricum_78
OTU_2287	9.59E-01	6.04E-01	6.29E-01	0.97	0.81	Eggerthella_lenta_99	Bacteria_Actinobacteria_99_Coriobacteriia_99_Eggerthellales_99_Eggerthellaceae_99_Eggerthella_99
OTU_2734	7.07E-01	6.04E-01	2.21E-01	1.20	0.70	Enterococcus_faecalis_90	Bacteria_Firmicutes_99_Clostridia_61_Clostridiales_61_Clostridiaceae 1_61_Desnuesiella_31
OTU_369	7.07E-01	6.04E-01	6.01E-01	2.04	0.95	Clostridium_saccharobutylicum_97	Bacteria_Firmicutes_Clostridia_Clostridiales_Clostridiaceae 1_Clostridium sensu stricto_100
OTU_445	8.49E-01	9.75E-01	1.15E-01	0.41	0.15	Anaerotaenia_torta_98	Bacteria_Firmicutes_Clostridia_Clostridiales_Lachnospiraceae_Anaerotaenia_22
OTU_4719	9.32E-01	8.78E-01	2.03E-02	1.91	0.42	Bacteroides_xylanisolvens_96	Bacteria_Bacteroidetes_Bacteroidia_Bacteroidales_Bacteroidaceae_Bacteroides_97
OTU_4891	7.82E-01	9.66E-01	8.14E-01	0.33	0.38	Dorea_formicigenerans_96	Bacteria_Firmicutes_Clostridia_Clostridiales_Lachnospiraceae_Dorea_56
OTU_9529	8.25E-01	8.82E-01	8.81E-01	0.77	0.82	Atopobacter_phocae_89	Bacteria_Firmicutes_Bacilli_89_Lactobacillales_75_Carnobacteriaceae_73_Isobaculum_57
OTU_11346	9.01E-01	6.14E-01	3.49E-01	0.73	1.04	Shigella_dysenteriae_95	Bacteria_Proteobacteria_Gammaproteobacteria_Enterobacterales_Enterobacteriaceae_Pseudescherichia_74
OTU_12867	8.25E-01	9.49E-01	1.10E-02	1.82	0.37	Clostridium_perfringens_96	Bacteria_Firmicutes_Clostridia_Clostridiales_Clostridiaceae 1_Sarcina_41
OTU_13290	8.25E-01	6.04E-01	9.48E-01	1.08	1.06	Kineothrix_alysoides_95	Bacteria_Firmicutes_Clostridia_Clostridiales_Lachnospiraceae_99_Coprococcus_34
OTU_1445	7.07E-01	9.53E-01	9.16E-02	1.49	0.36	Bacillus_cytotoxicus_93	Bacteria_Firmicutes_Bacilli_83_Lactobacillales_30_Carnobacteriaceae_25_Isobaculum_24
OTU_1829	9.29E-01	6.63E-01	2.79E-01	0.90	1.39	Flintibacter_butyricus_95	Bacteria_Firmicutes_Clostridia_Clostridiales_Ruminococcaceae_Flintibacter_82
OTU_2371	7.57E-01	9.82E-01	8.55E-05	1.74	0.28	Hungatella_effluvii_92	Bacteria_Firmicutes_Clostridia_Clostridiales_Lachnospiraceae_Clostridium XlVa_15
OTU_3018	9.38E-01	7.24E-01	7.65E-01	0.81	0.92	Lachnoclostridium_pacaense_93	Bacteria_Firmicutes_Clostridia_Clostridiales_Lachnospiraceae_Butyrivibrio_17
OTU_3294	7.07E-01	9.60E-01	4.12E-02	1.10	0.44	Bifidobacterium_stercoris_96	Bacteria_Actinobacteria_Actinobacteria_Bifidobacteriales_Bifidobacteriaceae_Bifidobacterium_75
OTU_4198	7.07E-01	7.73E-01	7.79E-02	2.15	0.40	Roseburia_inulinivorans_99	Bacteria_Firmicutes_94_Clostridia_94_Clostridiales_94_Lachnospiraceae_94_Roseburia_62
OTU_52443	7.13E-01	6.04E-01	5.52E-03	1.75	0.42	Bacteroides_uniformis_97	Bacteria_Bacteroidetes_Bacteroidia_Bacteroidales_Bacteroidaceae_Bacteroides_100
OTU_681	8.73E-01	9.59E-01	9.65E-01	0.09	0.08	Alistipes_senegalensis_100	Bacteria_Bacteroidetes_Bacteroidia_Bacteroidales_Rikenellaceae_Alistipes_100
OTU_7355	7.07E-01	6.04E-01	3.64E-01	0.80	1.28	Eubacterium_callanderi_95	Bacteria_Firmicutes_Clostridia_Clostridiales_Eubacteriaceae_Pseudoramibacter_85
OTU_8837	7.07E-01	8.33E-01	8.71E-01	1.40	1.30	Bacteroides_xylanisolvens_98	Bacteria_Bacteroidetes_Bacteroidia_Bacteroidales_Bacteroidaceae_Bacteroides_100
OTU_11136	9.38E-01	8.02E-01	9.60E-01	1.13	1.11	Enterocloster_citroniae_97	Bacteria_Firmicutes_Clostridia_Clostridiales_Lachnospiraceae_Enterocloster_71
OTU_1407	7.07E-01	6.04E-01	3.49E-02	0.38	1.88	Paraclostridium_benzoelyticum_98	Bacteria_Firmicutes_Clostridia_67_Clostridiales_67_Peptostreptococcaceae_67_Paraclostridium_67
OTU_16358	7.20E-01	8.95E-01	2.42E-02	0.52	1.35	Lentibacillus_juripiscarius_88	Bacteria_Firmicutes_Bacilli_96_Lactobacillales_76_Carnobacteriaceae_63_Isobaculum_48
OTU_20314	7.10E-01	NA	3.77E-02	3.28	0.00	Prevotella_copri_96	Bacteria_Bacteroidetes_Bacteroidia_Bacteroidales_Prevotellaceae_Prevotella_99
OTU_2053	8.05E-01	9.35E-01	1.00E-01	0.58	0.19	Phocaeicola_vulgatus_98	Bacteria_Bacteroidetes_82_Bacteroidia_82_Bacteroidales_82_Bacteroidaceae_82_Phocaeicola_62
OTU_3511	9.92E-01	8.71E-01	1.95E-02	2.06	0.21	Veillonella_parvula_98	Bacteria_Firmicutes_70_Negativicutes_70_Veillonellales_56_Veillonellaceae_56_Veillonella_54
OTU_50766	8.89E-01	9.27E-01	1.34E-01	1.18	0.80	Enterocloster_citroniae_97	Bacteria_Firmicutes_Clostridia_Clostridiales_Lachnospiraceae_Enterocloster_81
OTU_54520	9.87E-01	6.05E-01	7.42E-02	0.53	1.06	Enterococcus_faecalis_98	Bacteria_Firmicutes_Bacilli_Lactobacillales_Enterococcaceae_97_Enterococcus_62
OTU_5533	8.97E-01	6.04E-01	2.82E-01	0.82	1.37	Erysipelatoclostridium_ramosum_97	Bacteria_Firmicutes_Erysipelotrichia_Erysipelotrichales_Erysipelatoclostridiaceae_Erysipelatoclostridium_100
OTU_609	8.32E-01	6.04E-01	1.30E-01	2.02	0.18	Ileibacterium_valens_90	Bacteria_Firmicutes_Erysipelotrichia_Erysipelotrichales_Erysipelotrichaceae_Allobaculum_32
OTU_63739	7.75E-01	5.62E-01	2.45E-02	1.37	0.45	Clostridium_perfringens_97	Bacteria_Firmicutes_Clostridia_99_Clostridiales_99_Clostridiaceae 1_94_Clostridium sensu stricto_50
OTU_7232	8.31E-01	9.18E-01	2.34E-01	0.73	1.24	Faecalibacterium_prausnitzii_92	Bacteria_Firmicutes_Clostridia_Clostridiales_Ruminococcaceae_99_Faecalibacterium_62
OTU_7480	7.07E-01	7.29E-01	1.50E-02	1.44	0.64	Dorea_longicatena_97	Bacteria_Firmicutes_Clostridia_Clostridiales_Lachnospiraceae_Dorea_86
OTU_827	7.12E-01	6.04E-01	8.43E-01	1.37	1.14	Coprococcus_comes_97	Bacteria_Firmicutes_94_Clostridia_94_Clostridiales_94_Lachnospiraceae_94_Bariatricus_72
OTU_883	9.60E-01	6.04E-01	3.74E-02	1.78	0.09	Enterococcus_hirae_90	Bacteria_Firmicutes_Clostridia_72_Clostridiales_72_Clostridiaceae 1_72_Proteiniclasticum_38
OTU_926	9.08E-01	6.04E-01	1.30E-01	0.35	1.70	Eubacterium_limosum_92	Bacteria_Firmicutes_Clostridia_Clostridiales_Eubacteriaceae_94_Eubacterium_93
OTU_10182	7.07E-01	4.60E-01	6.59E-03	1.83	0.52	Clostridium_perfringens_89	Bacteria_Proteobacteria_82_Gammaproteobacteria_82_Enterobacterales_81_Enterobacteriaceae_81_Pseudescherichia_76
OTU_1453	7.07E-01	6.04E-01	1.94E-01	0.43	0.02	Megasphaera_hexanoica_94	Bacteria_Firmicutes_Negativicutes_Veillonellales_Veillonellaceae_Megasphaera_72
OTU_1483	7.74E-01	6.07E-01	7.99E-03	2.25	0.26	Clostridium_polysaccharolyticum_88	Bacteria_Firmicutes_70_Clostridia_70_Clostridiales_70_Lachnospiraceae_70_Bariatricus_24
OTU_1750	8.90E-01	9.46E-01	5.21E-01	0.97	1.25	Coprococcus_comes_95	Bacteria_Firmicutes_Clostridia_Clostridiales_Lachnospiraceae_Coprococcus_79
OTU_1831	8.85E-01	6.04E-01	3.58E-01	1.41	1.01	Hungatella_effluvii_97	Bacteria_Firmicutes_83_Clostridia_83_Clostridiales_83_Lachnospiraceae_83_Merdimonas_43
OTU_1885	9.75E-01	6.04E-01	1.46E-02	1.67	0.27	Enterococcus_hirae_97	Bacteria_Firmicutes_Bacilli_Lactobacillales_Enterococcaceae_91_Vagococcus_41
OTU_3359	7.98E-01	6.14E-01	8.39E-01	1.01	1.09	Enterococcus_hirae_92	Bacteria_Firmicutes_Bacilli_Lactobacillales_Enterococcaceae_91_Melissococcus_47
OTU_735	NA	7.08E-01	2.03E-02	0.00	1.43	Hyphomicrobium_aestuarii_94	Bacteria_Proteobacteria_Alphaproteobacteria_Rhizobiales_97_Xanthobacteraceae_24_Methylorhabdus_12
OTU_8976	9.05E-01	8.73E-01	9.78E-01	0.97	0.98	Enterococcus_faecalis_96	Bacteria_Firmicutes_Bacilli_99_Lactobacillales_97_Carnobacteriaceae_41_Isobaculum_28
OTU_107883	7.07E-01	9.35E-01	3.57E-01	1.47	0.09	Veillonella_rogosae_98	Bacteria_Firmicutes_Negativicutes_Veillonellales_Veillonellaceae_Veillonella_100
OTU_11659	9.43E-01	8.36E-01	5.52E-01	0.67	0.85	Lachnoclostridium_pacaense_98	Bacteria_Firmicutes_Clostridia_Clostridiales_Lachnospiraceae_Clostridium XlVa_44
OTU_13472	7.07E-01	6.90E-01	3.18E-01	0.68	1.03	Enterococcus_faecalis_96	Bacteria_Firmicutes_Bacilli_Lactobacillales_Enterococcaceae_83_Melissococcus_41
OTU_2994	9.75E-01	9.53E-01	1.55E-01	1.35	0.91	Coprococcus_comes_100	Bacteria_Firmicutes_Clostridia_Clostridiales_Lachnospiraceae_Coprococcus_69
OTU_36229	9.99E-01	6.04E-01	5.97E-01	0.67	0.79	Enterococcus_faecalis_97	Bacteria_Firmicutes_Bacilli_Lactobacillales_Enterococcaceae_92_Enterococcus_54
OTU_4516	9.43E-01	9.62E-01	7.42E-01	1.15	1.28	Enterocloster_clostridioformis_95	Bacteria_Firmicutes_Clostridia_Clostridiales_Lachnospiraceae_Coprococcus_37
OTU_5204	7.46E-01	6.57E-01	1.14E-03	1.29	0.19	Phocaeicola_vulgatus_88	Bacteria_Bacteroidetes_45_Bacteroidia_45_Bacteroidales_45_Bacteroidaceae_45_Phocaeicola_41
OTU_616	7.07E-01	9.91E-01	2.45E-01	0.12	0.02	Catenibacterium_mitsuokai_97	Bacteria_Firmicutes_96_Erysipelotrichia_96_Erysipelotrichales_96_Erysipelotrichaceae_96_Catenibacterium_83
OTU_7474	9.99E-01	9.94E-01	9.71E-01	0.67	0.68	Phocaeicola_coprophilus_97	Bacteria_Bacteroidetes_Bacteroidia_Bacteroidales_Bacteroidaceae_Phocaeicola_100
OTU_9261	7.07E-01	6.04E-01	1.27E-02	1.22	0.47	Bacteroides_uniformis_95	Bacteria_Bacteroidetes_Bacteroidia_Bacteroidales_Bacteroidaceae_Bacteroides_79
OTU_10568	8.88E-01	8.14E-01	2.17E-01	0.94	0.58	Enterocloster_clostridioformis_97	Bacteria_Firmicutes_Clostridia_Clostridiales_Lachnospiraceae_Enterocloster_54
OTU_1204	7.80E-01	6.14E-01	6.02E-03	1.03	0.21	Escherichia_coli_91	Bacteria_Proteobacteria_97_Gammaproteobacteria_97_Enterobacterales_97_Enterobacteriaceae_97_Pseudescherichia_95

OTU_1357	7.07E-01	7.31E-01	1.26E-01	1.11	0.02	Fusobacterium_mortiferum_94	Bacteria_Fusobacteria_78_Fusobacteriia_78_Fusobacteriales_78_Fusobacteriaceae_76_Cetobacterium_50
OTU_1367	8.49E-01	6.04E-01	4.41E-02	1.81	0.11	Veillonella_dispar_88	Bacteria_Fusobacteria_95_Fusobacteriia_95_Fusobacteriales_95_Fusobacteriaceae_90_Psychrilyobacter_34
OTU_1394	7.74E-01	6.14E-01	2.07E-01	1.52	0.60	Sutterella_wadsworthensis_98	Bacteria_Proteobacteria_84_Betaproteobacteria_83_Burkholderiales_83_Sutterellaceae_83_Sutterella_82
OTU_13955	7.81E-01	9.57E-01	1.95E-01	1.21	0.74	Enterocloster_citroniae_95	Bacteria_Firmicutes_Clostridia_Clostridiales_Lachnospiraceae_Enterocloster_86
OTU_1451	7.07E-01	7.38E-01	7.49E-02	0.93	0.24	Clostridium_perfringens_95	Bacteria_Firmicutes_Clostridia_Clostridiales_Clostridiaceae 1_89_Anaerobacter_32
OTU_1883	7.12E-01	9.18E-01	7.83E-03	0.51	1.82	Hungatella_hathewayi_91	Bacteria_Firmicutes_Negativicutes_51_Acidaminococcales_51_Acidaminococcaceae_51_Phascolarctobacterium_51
OTU_21165	9.37E-01	9.18E-01	9.78E-02	1.64	0.86	Roseburia_inulinivorans_97	Bacteria_Firmicutes_Clostridia_Clostridiales_Lachnospiraceae_Roseburia_99
OTU_30073	8.06E-01	9.62E-01	6.49E-01	1.05	1.22	Roseburia_inulinivorans_96	Bacteria_Firmicutes_Clostridia_Clostridiales_Lachnospiraceae_Roseburia_84
OTU_33867	7.07E-01	6.05E-01	3.09E-02	0.63	1.22	Enterococcus_faecalis_98	Bacteria_Firmicutes_Bacilli_Lactobacillales_Enterococcaceae_96_Enterococcus_75
OTU_3543	8.94E-01	9.11E-01	2.75E-02	1.69	0.50	Enterococcus_hirae_90	Bacteria_Firmicutes_89_Bacilli_89_Lactobacillales_82_Carnobacteriaceae_58_Isobaculum_51
OTU_36013	7.07E-01	9.18E-01	3.54E-02	0.68	0.17	Phocaeicola_vulgatus_97	Bacteria_Bacteroidetes_Bacteroidia_Bacteroidales_Bacteroidaceae_Phocaeicola_91
OTU_3733	7.07E-01	6.04E-01	9.85E-01	0.62	0.63	Bacteroides_mediterraneensis_97	Bacteria_Bacteroidetes_Bacteroidia_Bacteroidales_Bacteroidaceae_Phocaeicola_69
OTU_4212	9.56E-01	9.90E-01	3.90E-01	0.70	0.94	Hungatella_hathewayi_88	Bacteria_Firmicutes_97_Bacilli_79_Lactobacillales_73_Carnobacteriaceae_67_Isobaculum_56
OTU_748	7.85E-01	8.68E-01	8.23E-02	1.47	0.17	Kineothrix_alysoides_96	Bacteria_Firmicutes_Clostridia_Clostridiales_Lachnospiraceae_Kineothrix_77
OTU_1053	8.58E-01	9.70E-01	7.14E-01	0.07	0.05	Bifidobacterium_callitrichidarum_99	Bacteria_Actinobacteria_95_Actinobacteria_95_Bifidobacteriales_94_Bifidobacteriaceae_94_Neoscardovia_33
OTU_15442	7.07E-01	6.14E-01	1.15E-01	0.47	0.04	Streptococcus_sinensis_98	Bacteria_Firmicutes_Bacilli_Lactobacillales_Streptococcaceae_Streptococcus_100
OTU_2928	9.37E-01	9.32E-01	2.07E-01	0.86	0.55	Faecalibacterium_prausnitzii_98	Bacteria_Firmicutes_Clostridia_Clostridiales_Ruminococcaceae_Faecalibacterium_100
OTU_3330	9.43E-01	9.25E-01	2.03E-02	0.58	1.63	Enterocloster_aldenensis_96	Bacteria_Firmicutes_Clostridia_Clostridiales_Lachnospiraceae_98_Enterocloster_84
OTU_4180	9.68E-01	8.41E-01	2.46E-01	1.24	0.84	Escherichia_albertii_87	Bacteria_Firmicutes_77_Clostridia_77_Clostridiales_77_Lachnospiraceae_77_Ruminococcus2_36
OTU_4489	7.07E-01	8.17E-01	8.16E-01	1.02	0.93	Enterocloster_citroniae_97	Bacteria_Firmicutes_Clostridia_Clostridiales_Lachnospiraceae_Enterocloster_94
OTU_4939	NA	6.63E-01	1.35E-01	0.00	1.93	Methyloversatilis_universalis_97	Bacteria_Proteobacteria_Betaproteobacteria_Nitrosomonadales_68_Sterolibacteriaceae_68_Methyloversatilis_67
OTU_62047	1.00E+00	9.33E-01	1.21E-02	1.78	0.48	Bacteroides_xylanisolvens_98	Bacteria_Bacteroidetes_Bacteroidia_Bacteroidales_Bacteroidaceae_Bacteroides_100
OTU_7374	1.00E+00	6.94E-01	1.08E-01	0.36	0.76	Flintibacter_butyricus_92	Bacteria_Firmicutes_Clostridia_Clostridiales_Ruminococcaceae_61_Flintibacter_23
OTU_7590	9.45E-01	8.68E-01	8.19E-01	0.55	0.64	Paeniclostridium_sordellii_98	Bacteria_Firmicutes_Clostridia_Clostridiales_Peptostreptococcaceae_Paeniclostridium_100
OTU_80206	8.64E-01	6.04E-01	7.59E-02	0.55	1.23	Enterococcus_hirae_96	Bacteria_Firmicutes_Bacilli_Lactobacillales_Enterococcaceae_79_Vagococcus_33
OTU_8319	7.07E-01	7.61E-01	1.01E-01	0.45	0.17	Collinsella_aerofaciens_96	Bacteria_Actinobacteria_Coriobacteriia_Coriobacteriales_Coriobacteriaceae_Collinsella_100
OTU_8333	8.06E-01	8.68E-01	4.42E-01	0.74	0.97	Pseudescherichia_vulneris_96	Bacteria_Proteobacteria_Gammaproteobacteria_Enterobacterales_Enterobacteriaceae_Escherichia/Shigella_81
OTU_995	9.92E-01	8.68E-01	1.08E-01	0.91	1.66	Lachnotalea_glycerini_96	Bacteria_Firmicutes_Clostridia_Clostridiales_Lachnospiraceae_Clostridium XlVa_70
OTU_10124	7.07E-01	9.93E-01	5.10E-03	1.42	0.34	Bifidobacterium_catenulatum_96	Bacteria_Actinobacteria_Actinobacteria_Bifidobacteriales_Bifidobacteriaceae_Bifidobacterium_97
OTU_11158	7.07E-01	9.65E-01	1.46E-01	1.28	0.82	Hungatella_hathewayi_97	Bacteria_Firmicutes_Clostridia_Clostridiales_Lachnospiraceae_Hungatella_45
OTU_1141	7.07E-01	8.08E-01	1.42E-01	0.88	0.14	Phocaeicola_vulgatus_98	Bacteria_Bacteroidetes_95_Bacteroidia_95_Bacteroidales_95_Bacteroidaceae_95_Phocaeicola_80
OTU_1185	9.67E-01	7.73E-01	2.84E-02	1.27	0.35	Sutterella_massiliensis_99	Bacteria_Proteobacteria_Betaproteobacteria_Burkholderiales_97_Sutterellaceae_97_Sutterella_97
OTU_14631	9.43E-01	8.83E-01	5.71E-02	1.59	0.62	Hungatella_effluvii_95	Bacteria_Firmicutes_Clostridia_Clostridiales_Lachnospiraceae_Clostridium XlVa_73
OTU_1496	8.06E-01	7.15E-01	3.74E-02	1.47	0.51	Peptoniphilus_koenoeneniae_96	Bacteria_Firmicutes_Clostridia_Clostridiales_Peptoniphilaceae_76_Peptoniphilus_69
OTU_17354	7.12E-01	6.93E-01	8.22E-03	0.55	1.61	Bacteroides_thetaiotaomicron_100	Bacteria_Bacteroidetes_94_Bacteroidia_94_Bacteroidales_94_Bacteroidaceae_94_Bacteroides_88
OTU_1964	7.07E-01	7.94E-01	9.24E-02	0.50	1.80	Roseburia_inulinivorans_93	Bacteria_Firmicutes_Clostridia_Clostridiales_Lachnospiraceae_97_Roseburia_89
OTU_2212	7.80E-01	7.89E-01	5.38E-01	0.48	0.26	Pantoea_agglomerans_97	Bacteria_Proteobacteria_Gammaproteobacteria_Enterobacterales_Enterobacteriaceae_Leclercia_78
OTU_2225	7.07E-01	6.04E-01	7.00E-02	1.13	0.37	Roseburia_inulinivorans_91	Bacteria_Firmicutes_97_Clostridia_97_Clostridiales_97_Lachnospiraceae_97_Roseburia_65
OTU_3479	7.91E-01	7.36E-01	5.69E-01	0.93	1.10	Escherichia_albertii_88	Bacteria_Proteobacteria_76_Gammaproteobacteria_76_Enterobacterales_76_Enterobacteriaceae_76_Pseudescherichia_76
OTU_3772	9.66E-01	9.85E-01	8.21E-01	0.77	0.83	Pseudescherichia_vulneris_97	Bacteria_Proteobacteria_Gammaproteobacteria_Enterobacterales_Enterobacteriaceae_Escherichia/Shigella_90
OTU_3840	8.52E-01	6.66E-01	2.07E-01	0.93	0.30	Bacteroides_faecichinchillae_96	Bacteria_Bacteroidetes_Bacteroidia_Bacteroidales_Bacteroidaceae_98_Bacteroides_98
OTU_3960	9.84E-01	9.17E-01	1.39E-01	0.86	1.37	Coprococcus_comes_97	Bacteria_Firmicutes_Clostridia_61_Clostridiales_61_Lachnospiraceae_61_Bariatricus_34
OTU_4406	7.12E-01	6.83E-01	2.14E-01	0.68	0.23	Collinsella_aerofaciens_93	Bacteria_Actinobacteria_Coriobacteriia_Eggerthellales_67_Eggerthellaceae_67_Slackia_67
OTU_4948	9.64E-01	6.63E-01	1.12E-01	0.67	1.04	Hungatella_hathewayi_90	Bacteria_Firmicutes_96_Bacilli_57_Lactobacillales_41_Carnobacteriaceae_37_Isobaculum_25
OTU_494	7.46E-01	9.49E-01	6.72E-01	0.45	0.67	Serratia_surfactantfaciens_100	Bacteria_Proteobacteria_Gammaproteobacteria_Enterobacterales_Yersiniaceae_98_Serratia_98
OTU_496	7.98E-01	NA	1.77E-01	2.95	0.00	Clostridium_cocleatum_91	Bacteria_Firmicutes_Erysipelotrichia_Erysipelotrichales_Erysipelotrichaceae_Faecalibacillus_100
OTU_52219	7.46E-01	6.23E-01	1.61E-02	0.36	1.06	Enterococcus_faecalis_98	Bacteria_Firmicutes_Bacilli_Lactobacillales_Enterococcaceae_95_Enterococcus_67
OTU_61137	7.07E-01	9.57E-01	4.96E-02	2.15	0.16	Bacteroides_thetaiotaomicron_98	Bacteria_Bacteroidetes_Bacteroidia_Bacteroidales_Bacteroidaceae_Bacteroides_100
OTU_6302	7.82E-01	6.04E-01	1.03E-01	1.14	0.21	Vallitalea_pronyensis_86	Bacteria_Firmicutes_91_Clostridia_84_Clostridiales_83_Lachnospiraceae_22_Abyssivirga_10
OTU_10079	8.25E-01	6.72E-01	2.85E-02	1.33	0.36	Faecalicatena_orotica_97	Bacteria_Firmicutes_Clostridia_Clostridiales_Lachnospiraceae_Faecalicatena_48
OTU_10111	7.07E-01	9.90E-01	4.81E-01	1.07	0.84	Hespellia_porcina_90	Bacteria_Proteobacteria_63_Gammaproteobacteria_63_Enterobacterales_63_Enterobacteriaceae_63_Pseudescherichia_57
OTU_14999	7.12E-01	7.79E-01	2.48E-01	1.13	0.73	Bacteroides_fragilis_96	Bacteria_Bacteroidetes_Bacteroidia_Bacteroidales_Bacteroidaceae_Bacteroides_100
OTU_15516	9.02E-01	9.03E-01	6.73E-04	1.74	0.25	Clostridium_carnis_93	Bacteria_Firmicutes_Bacilli_58_Lactobacillales_49_Carnobacteriaceae_45_Isobaculum_37
OTU_1588	7.07E-01	6.04E-01	2.91E-03	2.19	0.14	Vagococcus_martis_87	Bacteria_Bacteroidetes_70_Bacteroidia_70_Bacteroidales_70_Bacteroidaceae_70_Mediterranea_67
OTU_16021	7.33E-01	9.03E-01	1.80E-02	1.84	0.26	Bacteroides_finegoldii_96	Bacteria_Bacteroidetes_Bacteroidia_Bacteroidales_Bacteroidaceae_Bacteroides_84
OTU_1791	7.46E-01	6.04E-01	4.63E-02	1.97	0.58	Mediterraneibacter_glycyrrhizinilyticus_94	Bacteria_Firmicutes_Clostridia_Clostridiales_Lachnospiraceae_99_Mediterraneibacter_90
OTU_2171	8.57E-01	6.19E-01	3.82E-03	1.90	0.09	Escherichia_coli_89	Bacteria_Firmicutes_65_Negativicutes_65_Acidaminococcales_65_Acidaminococcaceae_65_Phascolarctobacterium_65
OTU_3878	9.87E-01	4.60E-01	2.69E-01	0.70	1.17	Erysipelatoclostridium_ramosum_94	Bacteria_Firmicutes_69_Erysipelotrichia_68_Erysipelotrichales_68_Erysipelatoclostridiaceae_67_Erysipelatoclostridium_67
OTU_4860	7.07E-01	7.70E-01	4.11E-01	0.41	0.23	Bifidobacterium_catenulatum_95	Bacteria_Actinobacteria_95_Actinobacteria_95_Bifidobacteriales_94_Bifidobacteriaceae_94_Bifidobacterium_54
OTU_53928	7.07E-01	6.04E-01	8.92E-01	0.60	0.54	Citrobacter_braakii_98	Bacteria_Proteobacteria_Gammaproteobacteria_Enterobacterales_Enterobacteriaceae_97_Citrobacter_61
OTU_57958	8.49E-01	6.63E-01	1.94E-01	1.25	0.63	Clostridium_tarantellae_93	Bacteria_Firmicutes_Clostridia_Clostridiales_Lachnospiraceae_46_Mobilitalea_10
OTU_692	8.90E-01	8.28E-01	9.89E-01	0.19	0.18	Bifidobacterium_stercoris_99	Bacteria_Actinobacteria_87_Actinobacteria_87_Bifidobacteriales_87_Bifidobacteriaceae_87_Pseudoscardovia_44
OTU_71396	9.38E-01	6.04E-01	6.44E-03	1.42	0.26	Bacteroides_caccae_96	Bacteria_Bacteroidetes_Bacteroidia_Bacteroidales_Bacteroidaceae_Bacteroides_99
OTU_7361	8.29E-01	6.14E-01	3.80E-01	0.83	1.44	Dialister_succinatiphilus_94	Bacteria_Firmicutes_Negativicutes_Veillonellales_95_Veillonellaceae_95_Dialister_94
OTU_91205	8.94E-01	8.89E-01	6.97E-04	0.12	1.68	Bacteroides_faecichinchillae_96	Bacteria_Bacteroidetes_Bacteroidia_Bacteroidales_Bacteroidaceae_Bacteroides_96
OTU_101026	9.46E-01	9.98E-01	9.20E-01	0.81	0.84	Enterocloster_asparagiformis_96	Bacteria_Firmicutes_Clostridia_Clostridiales_Lachnospiraceae_Enterocloster_34
OTU_11582	7.07E-01	9.46E-01	4.62E-01	0.65	0.37	Peptoniphilus_duerdenii_96	Bacteria_Firmicutes_Clostridia_Clostridiales_Peptoniphilaceae_Peptoniphilus_100

OTU_12781	7.46E-01	8.77E-01	1.30E-03	1.39	0.23	Veillonella_atypica_89	Bacteria_Firmicutes_Clostridia_96_Clostridiales_96_Lachnospiraceae_96_Bariatricus_74
OTU_1887	8.74E-01	6.35E-01	9.63E-03	1.55	0.32	Senegalimassilia_anaerobia_93	Bacteria_Actinobacteria_Coriobacteriia_Coriobacteriales_Coriobacteriaceae_Senegalimassilia_100
OTU_19515	7.93E-01	6.72E-01	3.49E-01	1.07	0.68	Enterococcus_hirae_97	Bacteria_Firmicutes_Bacilli_Lactobacillales_97_Enterococcaceae_70_Enterococcus_58
OTU_2246	1.00E+00	8.47E-01	8.21E-01	1.08	0.97	Anaerotaenia_torta_89	Bacteria_Firmicutes_93_Clostridia_74_Clostridiales_74_Lachnospiraceae_74_Anaerotaenia_9
OTU_28061	8.06E-01	6.30E-01	4.00E-01	1.07	0.82	Hespellia_porcina_89	Bacteria_Firmicutes_57_Clostridia_57_Clostridiales_57_Lachnospiraceae_57_Catonella_30
OTU_2921	7.07E-01	6.06E-01	7.47E-01	0.83	0.93	Flavonifractor_plautii_100	Bacteria_Firmicutes_Clostridia_Clostridiales_Ruminococcaceae_Flavonifractor_100
OTU_3081	7.07E-01	6.04E-01	7.37E-01	0.97	0.79	Coprococcus_comes_97	Bacteria_Firmicutes_Clostridia_Clostridiales_Lachnospiraceae_Bariatricus_71
OTU_500	9.08E-01	7.31E-01	2.19E-01	0.34	0.11	Prevotella_stercorea_97	Bacteria_Bacteroidetes_Bacteroidia_Bacteroidales_Prevotellaceae_Prevotella_100
OTU_776	7.46E-01	5.09E-01	3.57E-01	0.58	0.12	Streptococcus_peroris_89	Bacteria_Firmicutes_Clostridia_91_Clostridiales_91_Lachnospiraceae_91_Merdimonas_32
OTU_8047	7.57E-01	6.14E-01	7.67E-02	1.70	0.57	Clostridium_perfringens_96	Bacteria_Firmicutes_Clostridia_98_Clostridiales_98_Clostridiaceae 1_86_Sarcina_50
OTU_8094	8.99E-01	6.04E-01	6.80E-02	0.56	1.42	Paraclostridium_benzoelyticum_97	Bacteria_Firmicutes_Clostridia_Clostridiales_Peptostreptococcaceae_Paraclostridium_88
OTU_825	9.47E-01	8.02E-01	6.95E-01	0.24	0.16	Megasphaera_elsdenii_96	Bacteria_Firmicutes_Negativicutes_99_Veillonellales_99_Veillonellaceae_99_Megasphaera_99
OTU_941	7.07E-01	6.04E-01	1.48E-01	0.63	0.19	Peptoniphilus_grossensis_96	Bacteria_Firmicutes_Clostridia_Clostridiales_Peptoniphilaceae_Peptoniphilus_100
OTU_9850	9.73E-01	8.45E-01	3.70E-03	1.89	0.35	Clostridium_tarantellae_96	Bacteria_Firmicutes_Clostridia_Clostridiales_Clostridiaceae 1_81_Sarcina_46
OTU_10199	7.91E-01	4.60E-01	8.23E-01	0.84	0.93	Pseudescherichia_vulneris_97	Bacteria_Proteobacteria_Gammaproteobacteria_Enterobacterales_Enterobacteriaceae_Escherichia/Shigella_65
OTU_1486	9.72E-01	6.04E-01	4.03E-03	1.57	0.07	Phascolarctobacterium_succinatutens_91	Bacteria_Firmicutes_Negativicutes_98_Acidaminococcales_98_Acidaminococcaceae_98_Phascolarctobacterium_98
OTU_16436	9.46E-01	6.63E-01	2.17E-01	0.59	0.98	Enterocloster_citroniae_95	Bacteria_Firmicutes_Clostridia_Clostridiales_Lachnospiraceae_Enterocloster_59
OTU_1735	7.07E-01	6.04E-01	4.34E-01	0.98	0.72	Bacteroides_caccae_96	Bacteria_Bacteroidetes_Bacteroidia_Bacteroidales_Bacteroidaceae_Bacteroides_99
OTU_1780	9.89E-01	6.04E-01	3.71E-01	0.95	1.33	Acidaminococcus_fermentans_98	Bacteria_Firmicutes_Negativicutes_94_Acidaminococcales_94_Acidaminococcaceae_94_Acidaminococcus_92
OTU_1793	9.87E-01	9.94E-01	6.65E-02	1.24	0.53	Blautia_faecicola_93	Bacteria_Firmicutes_Clostridia_94_Clostridiales_94_Lachnospiraceae_94_Eisenbergiella_15
OTU_1801	7.07E-01	9.94E-01	7.79E-01	1.04	0.90	Escherichia_albertii_88	Bacteria_Proteobacteria_69_Gammaproteobacteria_69_Enterobacterales_69_Enterobacteriaceae_69_Pseudescherichia_69
OTU_2671	7.34E-01	6.72E-01	3.06E-02	1.84	0.19	Pseudescherichia_vulneris_100	Bacteria_Proteobacteria_Gammaproteobacteria_Enterobacterales_98_Enterobacteriaceae_97_Pseudescherichia_42
OTU_3026	7.91E-01	6.39E-01	1.27E-01	1.47	0.66	Veillonella_parvula_98	Bacteria_Firmicutes_Negativicutes_99_Veillonellales_86_Veillonellaceae_86_Veillonella_86
OTU_33365	1.00E+00	9.32E-01	9.14E-01	0.91	0.94	Pseudescherichia_vulneris_100	Bacteria_Proteobacteria_Gammaproteobacteria_Enterobacterales_Enterobacteriaceae_Pseudescherichia_43
OTU_3614	9.04E-01	7.99E-01	1.45E-04	2.10	0.14	Bacteroides_xylanisolvens_96	Bacteria_Bacteroidetes_Bacteroidia_Bacteroidales_Bacteroidaceae_97_Bacteroides_97
OTU_3656	9.87E-01	6.10E-01	9.05E-03	2.02	0.07	Fusobacterium_mortiferum_95	Bacteria_Fusobacteria_99_Fusobacteriia_99_Fusobacteriales_99_Fusobacteriaceae_95_Cetobacterium_62
OTU_645	9.92E-01	6.04E-01	2.44E-01	1.14	0.06	Liquorilactobacillus_satsumensis_97	Bacteria_Firmicutes_Bacilli_Lactobacillales_Lactobacillaceae_Ligilactobacillus_56
OTU_741	8.95E-01	9.91E-01	3.39E-02	1.85	0.05	Falcatimonas_natans_96	Bacteria_Firmicutes_Clostridia_Clostridiales_Lachnospiraceae_Mediterraneibacter_36
OTU_803	8.37E-01	6.04E-01	4.45E-03	1.93	0.13	Dielma_fastidiosa_100	Bacteria_Firmicutes_Erysipelotrichia_Erysipelotrichales_Erysipelotrichaceae_Dielma_100
OTU_1142	7.87E-01	NA	1.78E-01	0.19	0.00	Mitsuokella_multacida_93	Bacteria_Firmicutes_Negativicutes_Selenomonadales_62_Selenomonadaceae_62_Mitsuokella_62
OTU_11667	7.46E-01	9.53E-01	3.75E-01	0.12	0.27	Kosakonia_cowanii_98	Bacteria_Proteobacteria_Gammaproteobacteria_Enterobacterales_Enterobacteriaceae_87_Citrobacter_21
OTU_13909	9.41E-01	6.05E-01	5.64E-02	0.86	0.43	Clostridium_perfringens_97	Bacteria_Firmicutes_Clostridia_Clostridiales_Clostridiaceae 1_83_Clostridium sensu stricto_52
OTU_15547	7.07E-01	8.79E-01	9.96E-01	0.82	0.82	Eisenbergiella_massiliensis_89	Bacteria_Proteobacteria_57_Gammaproteobacteria_57_Enterobacterales_57_Enterobacteriaceae_56_Pseudescherichia_51
OTU_16253	8.85E-01	8.85E-01	8.73E-01	0.14	0.16	Kosakonia_cowanii_98	Bacteria_Proteobacteria_Gammaproteobacteria_Enterobacterales_Enterobacteriaceae_90_Kosakonia_35
OTU_2073	7.70E-01	6.04E-01	3.48E-02	0.37	0.83	Escherichia_albertii_92	Bacteria_Proteobacteria_85_Gammaproteobacteria_85_Enterobacterales_85_Enterobacteriaceae_82_Pseudescherichia_69
OTU_2084	9.06E-01	8.36E-01	6.43E-01	0.33	0.25	Anaerobutyricum_hallii_95	Bacteria_Firmicutes_Clostridia_Clostridiales_Lachnospiraceae_Clostridium XlVa_67
OTU_2108	9.48E-01	7.80E-01	2.03E-03	0.38	1.40	Bacteroides_thetaiotaomicron_100	Bacteria_Bacteroidetes_Bacteroidia_Bacteroidales_Bacteroidaceae_Bacteroides_100
OTU_4139	9.37E-01	6.06E-01	4.19E-01	0.77	1.14	Lachnoclostridium_pacaense_96	Bacteria_Firmicutes_Clostridia_Clostridiales_Lachnospiraceae_Clostridium XlVa_46
OTU_4521	7.07E-01	8.10E-01	2.68E-03	0.17	1.16	Enterococcus_faecalis_89	Bacteria_Firmicutes_Bacilli_40_Lactobacillales_35_Carnobacteriaceae_25_Catellicoccus_17
OTU_4746	7.57E-01	8.65E-01	8.10E-01	1.05	0.97	Ruminococcus_gnavus_92	Bacteria_Firmicutes_87_Clostridia_87_Clostridiales_87_Lachnospiraceae_87_Murimonas_10
OTU_5128	7.07E-01	7.16E-01	2.49E-01	1.19	0.75	Pseudescherichia_vulneris_99	Bacteria_Proteobacteria_97_Gammaproteobacteria_97_Enterobacterales_97_Enterobacteriaceae_97_Pseudescherichia_84
OTU_5378	7.07E-01	6.04E-01	1.35E-02	1.28	0.44	Enterococcus_hirae_97	Bacteria_Firmicutes_Bacilli_99_Lactobacillales_98_Enterococcaceae_87_Vagococcus_22
OTU_53900	8.48E-01	6.04E-01	4.97E-02	0.43	1.10	Enterococcus_faecalis_98	Bacteria_Firmicutes_Bacilli_Lactobacillales_Enterococcaceae_96_Enterococcus_61
OTU_5430	9.87E-01	6.57E-01	2.45E-04	1.91	0.21	Parabacteroides_distasonis_95	Bacteria_Bacteroidetes_Bacteroidia_Bacteroidales_Porphyromonadaceae_87_Parabacteroides_87
OTU_64440	8.31E-01	6.04E-01	8.75E-01	0.90	0.85	Enterococcus_faecalis_97	Bacteria_Firmicutes_Bacilli_Lactobacillales_Enterococcaceae_84_Enterococcus_44
OTU_64916	7.07E-01	8.68E-01	8.70E-02	1.85	0.13	Bacteroides_eggerthii_95	Bacteria_Bacteroidetes_Bacteroidia_Bacteroidales_Bacteroidaceae_Bacteroides_99
OTU_67857	8.16E-01	8.68E-01	6.82E-02	0.55	1.00	Enterococcus_faecalis_98	Bacteria_Firmicutes_Bacilli_Lactobacillales_Enterococcaceae_99_Enterococcus_72
OTU_862	7.07E-01	6.04E-01	2.45E-01	0.30	1.65	Veillonella_dispar_89	Bacteria_Firmicutes_98_Negativicutes_94_Veillonellales_63_Veillonellaceae_63_Veillonella_60
OTU_1255	9.66E-01	6.04E-01	1.16E-01	1.78	0.39	Enterococcus_dispar_94	Bacteria_Firmicutes_Bacilli_65_Lactobacillales_65_Enterococcaceae_43_Melissococcus_42
OTU_1790	7.07E-01	8.14E-01	6.44E-03	1.37	0.19	Bacteroides_thetaiotaomicron_97	Bacteria_Bacteroidetes_Bacteroidia_Bacteroidales_Bacteroidaceae_Bacteroides_100
OTU_1929	8.25E-01	6.04E-01	3.10E-01	1.26	0.61	Enterocloster_citroniae_97	Bacteria_Firmicutes_94_Clostridia_94_Clostridiales_94_Lachnospiraceae_94_Enterocloster_55
OTU_35767	9.08E-01	6.04E-01	3.93E-04	1.84	0.35	Bacteroides_xylanisolvens_98	Bacteria_Bacteroidetes_Bacteroidia_Bacteroidales_Bacteroidaceae_Bacteroides_100
OTU_37876	7.93E-01	6.04E-01	1.57E-01	1.35	0.46	Fusobacterium_mortiferum_90	Bacteria_Fusobacteria_78_Fusobacteriia_78_Fusobacteriales_78_Fusobacteriaceae_78_Cetobacterium_52
OTU_3855	7.57E-01	7.08E-01	1.35E-02	0.29	1.58	Veillonella_dispar_98	Bacteria_Firmicutes_58_Negativicutes_58_Selenomonadales_28_Sporomusaceae_26_Anaerosinus_19
OTU_3953	8.25E-01	6.04E-01	3.42E-02	0.41	1.54	Erysipelatoclostridium_ramosum_88	Bacteria_Firmicutes_Erysipelotrichia_96_Erysipelotrichales_96_Erysipelatoclostridiaceae_95_Erysipelatoclostridium_95
OTU_4280	7.11E-01	6.04E-01	2.01E-01	1.05	0.64	Enterococcus_faecalis_90	Bacteria_Proteobacteria_63_Gammaproteobacteria_63_Enterobacterales_59_Enterobacteriaceae_58_Pseudescherichia_55
OTU_4770	7.07E-01	8.14E-01	4.37E-02	1.59	0.75	Roseburia_inulinivorans_96	Bacteria_Firmicutes_Clostridia_Clostridiales_Lachnospiraceae_Roseburia_76
OTU_5079	NA	6.65E-01	1.10E-01	0.00	2.03	Piscinibacterium_candidicorallinum_96	Bacteria_Proteobacteria_Betaproteobacteria_Nitrosomonadales_43_Sterolibacteriaceae_43_Methyloversatilis_43
OTU_578	8.29E-01	7.31E-01	5.25E-02	2.05	0.03	Prevotella_stercorea_92	Bacteria_Bacteroidetes_Bacteroidia_Bacteroidales_Prevotellaceae_Prevotella_91
OTU_610	9.08E-01	6.14E-01	1.19E-01	2.02	0.32	Acetivibrio_clariflavus_88	Bacteria_Firmicutes_99_Clostridia_99_Clostridiales_99_Ruminococcaceae_68_Flintibacter_9
OTU_65820	7.07E-01	8.02E-01	9.69E-01	0.77	0.76	Pseudescherichia_vulneris_90	Bacteria_Proteobacteria_83_Gammaproteobacteria_83_Enterobacterales_82_Enterobacteriaceae_79_Pseudescherichia_78
OTU_70560	7.07E-01	8.79E-01	1.45E-01	0.95	0.29	Veillonella_denticariosi_98	Bacteria_Firmicutes_Negativicutes_Veillonellales_Veillonellaceae_Veillonella_100
OTU_74210	7.93E-01	7.07E-01	7.65E-02	0.48	0.10	Bifidobacterium_stercoris_98	Bacteria_Actinobacteria_Actinobacteria_Bifidobacteriales_Bifidobacteriaceae_Bifidobacterium_90
OTU_75428	9.87E-01	9.90E-01	2.04E-02	0.68	1.26	Shigella_dysenteriae_96	Bacteria_Proteobacteria_Gammaproteobacteria_Enterobacterales_Enterobacteriaceae_Escherichia/Shigella_62
OTU_7999	7.57E-01	6.04E-01	8.23E-02	0.57	1.24	Senegalimassilia_anaerobia_96	Bacteria_Actinobacteria_95_Coriobacteriia_95_Coriobacteriales_95_Coriobacteriaceae_95_Senegalimassilia_95
OTU_8064	7.07E-01	6.04E-01	6.60E-01	0.76	0.89	Enterococcus_faecalis_93	Bacteria_Firmicutes_Bacilli_58_Lactobacillales_57_Carnobacteriaceae_37_Catellicoccus_21
OTU_83297	7.07E-01	6.63E-01	4.34E-01	1.36	0.70	Pseudescherichia_vulneris_91	Bacteria_Proteobacteria_Gammaproteobacteria_53_Enterobacterales_53_Enterobacteriaceae_53_Pseudescherichia_53

OTU_8764	9.87E-01	6.72E-01	6.29E-01	1.10	0.92	Kineothrix_alysoides_94	Bacteria_Firmicutes_Clostridia_Clostridiales_Lachnospiraceae_Clostridium XlVa_45
OTU_95620	9.92E-01	7.65E-01	1.25E-01	0.28	2.07	Kineothrix_alysoides_97	Bacteria_Firmicutes_Clostridia_Clostridiales_Lachnospiraceae_Kineothrix_52
OTU_11406	7.07E-01	7.31E-01	2.34E-01	1.08	0.44	Bacteroides_xylanisolvens_97	Bacteria_Bacteroidetes_Bacteroidia_Bacteroidales_Bacteroidaceae_Bacteroides_99
OTU_11575	8.06E-01	6.10E-01	1.70E-02	1.21	0.28	Pseudescherichia_vulneris_97	Bacteria_Proteobacteria_94_Gammaproteobacteria_94_Enterobacterales_94_Enterobacteriaceae_93_Pseudescherichia_82
OTU_1170	7.85E-01	7.55E-01	3.22E-02	1.88	0.23	Clostridium_perfringens_98	Bacteria_Firmicutes_Clostridia_74_Clostridiales_74_Clostridiaceae 1_74_Sarcina_46
OTU_13023	7.07E-01	6.39E-01	1.01E-02	1.98	0.07	Bacteroides_stercoris_98	Bacteria_Bacteroidetes_92_Bacteroidia_92_Bacteroidales_92_Bacteroidaceae_90_Bacteroides_71
OTU_1608	8.77E-01	6.04E-01	2.08E-02	1.71	0.34	Kineothrix_alysoides_97	Bacteria_Firmicutes_Clostridia_Clostridiales_Lachnospiraceae_Kineothrix_45
OTU_1752	8.85E-01	9.95E-01	1.07E-01	1.31	0.70	Intestinibacter_bartlettii_89	Bacteria_Bacteroidetes_79_Bacteroidia_79_Bacteroidales_79_Bacteroidaceae_78_Mediterranea_55
OTU_2142	9.59E-01	6.04E-01	9.61E-01	0.71	0.72	Senegalimassilia_anaerobia_97	Bacteria_Actinobacteria_Coriobacteriia_Coriobacteriales_Coriobacteriaceae_Senegalimassilia_100
OTU_22056	9.96E-01	8.50E-01	6.37E-01	0.69	0.80	Enterococcus_faecalis_98	Bacteria_Firmicutes_Bacilli_Lactobacillales_Enterococcaceae_99_Enterococcus_93
OTU_2252	NA	7.58E-01	1.27E-02	0.00	1.37	Pseudescherichia_vulneris_99	Bacteria_Proteobacteria_Gammaproteobacteria_98_Enterobacterales_97_Enterobacteriaceae_97_Pseudescherichia_66
OTU_31210	8.26E-01	9.12E-01	8.37E-02	0.56	1.74	Bacteroides_thetaiotaomicron_97	Bacteria_Bacteroidetes_Bacteroidia_Bacteroidales_Bacteroidaceae_Bacteroides_100
OTU_3937	7.57E-01	9.80E-01	1.46E-02	0.31	1.52	Phascolarctobacterium_faecium_97	Bacteria_Firmicutes_Negativicutes_Acidaminococcales_Acidaminococcaceae_Phascolarctobacterium_100
OTU_463	9.23E-01	6.04E-01	3.74E-02	1.57	0.30	Treponema_succinifaciens_87	Bacteria_Spirochaetes_64_Spirochaetia_64_Spirochaetales_64_Spirochaetaceae_64_Rectinema_23
OTU_4664	8.33E-01	6.04E-01	1.94E-02	2.09	0.16	Roseburia_inulinivorans_94	Bacteria_Firmicutes_Clostridia_Clostridiales_Lachnospiraceae_99_Roseburia_55
OTU_4874	7.59E-01	6.04E-01	9.39E-01	0.07	0.07	Kosakonia_oryzendophytica_88	Bacteria_Firmicutes_77_Clostridia_77_Clostridiales_77_Lachnospiraceae_76_Fusicatenibacter_14
OTU_6367	9.31E-01	7.51E-01	1.66E-01	0.50	1.06	Enterocloster_clostridioformis_94	Bacteria_Firmicutes_Clostridia_Clostridiales_Lachnospiraceae_86_Enterocloster_81
OTU_66961	9.65E-01	8.18E-01	2.50E-01	1.43	0.84	Enterococcus_faecalis_91	Bacteria_Firmicutes_93_Bacilli_66_Lactobacillales_63_Carnobacteriaceae_43_Catellicoccus_38
OTU_8132	9.38E-01	6.68E-01	4.05E-02	1.72	0.28	Bacteroides_xylanisolvens_96	Bacteria_Bacteroidetes_Bacteroidia_Bacteroidales_Bacteroidaceae_72_Bacteroides_71
OTU_8428	7.07E-01	9.35E-01	2.86E-02	1.58	0.82	Bacteroides_xylanisolvens_95	Bacteria_Bacteroidetes_Bacteroidia_Bacteroidales_Bacteroidaceae_Bacteroides_98
OTU_8433	9.92E-01	8.65E-01	5.96E-01	0.98	0.76	Enterococcus_hirae_91	Bacteria_Proteobacteria_61_Gammaproteobacteria_61_Enterobacterales_60_Enterobacteriaceae_59_Pseudescherichia_55
OTU_984	8.13E-01	6.04E-01	3.35E-02	1.33	0.09	Lactobacillus_johnsonii_96	Bacteria_Firmicutes_Bacilli_Lactobacillales_Lactobacillaceae_Lactobacillus_60
OTU_11215	8.67E-01	6.66E-01	1.44E-02	0.35	1.59	Bacteroides_fragilis_95	Bacteria_Bacteroidetes_52_Bacteroidia_52_Bacteroidales_52_Bacteroidaceae_52_Mediterranea_37
OTU_11774	9.08E-01	6.04E-01	1.77E-01	0.67	1.22	Roseburia_inulinivorans_89	Bacteria_Firmicutes_Erysipelotrichia_61_Erysipelotrichales_61_Erysipelatoclostridiaceae_61_Erysipelatoclostridium_61
OTU_13677	9.99E-01	6.05E-01	1.38E-01	1.00	0.45	Peptoniphilus_koenoeneniae_97	Bacteria_Firmicutes_Clostridia_99_Clostridiales_99_Peptoniphilaceae_90_Peptoniphilus_76
OTU_1404	8.95E-01	6.64E-01	6.32E-02	0.29	1.53	Bacteroides_fragilis_100	Bacteria_Bacteroidetes_86_Bacteroidia_86_Bacteroidales_86_Bacteroidaceae_85_Bacteroides_56
OTU_14586	7.46E-01	6.50E-01	3.49E-01	0.65	1.35	Kineothrix_alysoides_96	Bacteria_Firmicutes_Clostridia_Clostridiales_Lachnospiraceae_Ruminococcus2_38
OTU_15548	7.46E-01	6.50E-01	8.98E-01	0.74	0.79	Veillonella_tobetsuensis_86	Bacteria_Proteobacteria_80_Gammaproteobacteria_80_Enterobacterales_79_Enterobacteriaceae_78_Pseudescherichia_78
OTU_1632	9.78E-01	6.04E-01	3.31E-02	1.91	0.09	Ruminococcus_bromii_94	Bacteria_Firmicutes_Clostridia_Clostridiales_Ruminococcaceae_91_Ruminococcus_72
OTU_2159	7.07E-01	7.31E-01	9.21E-02	2.14	0.02	Streptococcus_thermophilus_99	Bacteria_Firmicutes_38_Bacilli_36_Lactobacillales_36_Enterococcaceae_21_Pilibacter_19
OTU_2957	7.10E-01	6.39E-01	3.89E-01	0.68	1.15	Fusobacterium_nucleatum_100	Bacteria_Fusobacteria_Fusobacteriia_Fusobacteriales_Fusobacteriaceae_Fusobacterium_100
OTU_32087	8.25E-01	9.61E-01	6.21E-01	0.88	1.04	Pseudescherichia_vulneris_96	Bacteria_Proteobacteria_89_Gammaproteobacteria_89_Enterobacterales_89_Enterobacteriaceae_87_Pseudescherichia_70
OTU_3474	7.07E-01	9.54E-01	3.33E-04	2.14	0.17	Bacteroides_finegoldii_96	Bacteria_Bacteroidetes_Bacteroidia_Bacteroidales_Bacteroidaceae_Bacteroides_100
OTU_3950	7.07E-01	6.04E-01	1.01E-01	0.48	1.33	Pseudescherichia_vulneris_89	Bacteria_Proteobacteria_80_Gammaproteobacteria_80_Enterobacterales_80_Enterobacteriaceae_76_Pseudescherichia_76
OTU_4113	7.07E-01	7.40E-01	7.42E-02	1.21	0.38	Bifidobacterium_catenulatum_95	Bacteria_Actinobacteria_99_Actinobacteria_99_Bifidobacteriales_99_Bifidobacteriaceae_99_Bifidobacterium_67
OTU_4522	8.06E-01	8.68E-01	4.00E-01	0.59	0.90	Escherichia_albertii_90	Bacteria_Proteobacteria_85_Gammaproteobacteria_85_Enterobacterales_85_Enterobacteriaceae_85_Pseudescherichia_84
OTU_4803	7.07E-01	8.63E-01	3.25E-01	0.81	1.10	Escherichia_coli_97	Bacteria_Proteobacteria_Gammaproteobacteria_Enterobacterales_Enterobacteriaceae_Escherichia/Shigella_98
OTU_4997	1.00E+00	9.59E-01	4.04E-03	0.56	1.42	Gibbsiella_dentisursi_90	Bacteria_Proteobacteria_Gammaproteobacteria_Enterobacterales_Enterobacteriaceae_Pseudescherichia_94
OTU_5361	9.92E-01	6.14E-01	1.21E-02	1.91	0.08	Prevotella_copri_97	Bacteria_Bacteroidetes_Bacteroidia_Bacteroidales_Prevotellaceae_Prevotella_100
OTU_549	8.16E-01	7.31E-01	4.62E-02	2.43	0.03	Ruminococcus_albus_97	Bacteria_Firmicutes_Clostridia_Clostridiales_Ruminococcaceae_Ruminococcus_98
OTU_5699	7.11E-01	6.04E-01	1.32E-03	1.63	0.35	Bacteroides_uniformis_96	Bacteria_Bacteroidetes_Bacteroidia_Bacteroidales_Bacteroidaceae_Bacteroides_98
OTU_13170	8.49E-01	7.97E-01	4.58E-01	0.82	1.08	Ruminococcus_torques_96	Bacteria_Firmicutes_Clostridia_Clostridiales_Lachnospiraceae_Coprococcus_24
OTU_1769	9.92E-01	7.46E-01	7.99E-03	1.18	0.14	Butyricimonas_paravirosa_98	Bacteria_Bacteroidetes_Bacteroidia_Bacteroidales_Odoribacteraceae_Butyricimonas_100
OTU_1788	8.97E-01	8.71E-01	2.86E-01	0.61	0.96	Dorea_longicatena_97	Bacteria_Firmicutes_Clostridia_Clostridiales_Lachnospiraceae_Dorea_70
OTU_1961	8.06E-01	6.14E-01	1.44E-01	0.51	1.27	Clostridium_perfringens_99	Bacteria_Firmicutes_Negativicutes_54_Acidaminococcales_54_Acidaminococcaceae_54_Phascolarctobacterium_54
OTU_22882	8.25E-01	9.52E-01	2.38E-02	1.23	0.55	Flavonifractor_plautii_91	Bacteria_Firmicutes_Clostridia_Clostridiales_Ruminococcaceae_80_Flavonifractor_80
OTU_3150	7.75E-01	9.46E-01	6.77E-01	0.64	0.92	Limosilactobacillus_mucosae_96	Bacteria_Firmicutes_Bacilli_99_Lactobacillales_99_Lactobacillaceae_99_Limosilactobacillus_98
OTU_31908	9.38E-01	6.04E-01	2.15E-03	1.32	0.40	Clostridium_perfringens_97	Bacteria_Firmicutes_Clostridia_Clostridiales_Clostridiaceae 1_99_Clostridium sensu stricto_58
OTU_3357	7.07E-01	9.71E-01	7.26E-01	0.88	1.07	Desulfovibrio_piger_96	Bacteria_Proteobacteria_Deltaproteobacteria_98_Desulfovibrionales_98_Desulfovibrionaceae_98_Desulfovibrio_75
OTU_34435	9.77E-01	8.17E-01	2.15E-01	0.28	1.79	Paeniclostridium_sordellii_100	Bacteria_Firmicutes_Clostridia_Clostridiales_Peptostreptococcaceae_Paeniclostridium_100
OTU_37797	9.38E-01	9.33E-01	1.97E-03	1.95	0.09	Bacteroides_caccae_97	Bacteria_Bacteroidetes_Bacteroidia_Bacteroidales_Bacteroidaceae_Bacteroides_100
OTU_4342	9.92E-01	6.04E-01	4.89E-02	1.37	0.68	Coprococcus_comes_95	Bacteria_Firmicutes_Clostridia_Clostridiales_Lachnospiraceae_Bariatricus_74
OTU_4854	1.00E+00	8.28E-01	1.95E-02	0.10	0.57	Gemmiger_formicilis_96	Bacteria_Firmicutes_Clostridia_Clostridiales_Ruminococcaceae_Gemmiger_75
OTU_75340	8.82E-01	6.04E-01	5.37E-04	1.13	0.07	Bacteroides_caccae_96	Bacteria_Bacteroidetes_Bacteroidia_Bacteroidales_Bacteroidaceae_Bacteroides_98
OTU_12307	9.06E-01	6.04E-01	2.55E-02	1.43	0.16	Phascolarctobacterium_succinatutens_96	Bacteria_Firmicutes_Negativicutes_Acidaminococcales_Acidaminococcaceae_Phascolarctobacterium_100
OTU_13498	9.87E-01	9.46E-01	1.63E-02	1.36	0.21	Bacteroides_uniformis_94	Bacteria_Bacteroidetes_Bacteroidia_Bacteroidales_Bacteroidaceae_Phocaeicola_59
OTU_1450	8.25E-01	6.04E-01	9.72E-01	0.22	0.21	Flavonifractor_plautii_89	Bacteria_Firmicutes_96_Clostridia_96_Clostridiales_96_Ruminococcaceae_96_Flavonifractor_95
OTU_2535	7.70E-01	6.10E-01	4.46E-01	1.18	0.67	Romboutsia_timonensis_98	Bacteria_Firmicutes_Clostridia_Clostridiales_Peptostreptococcaceae_Romboutsia_100
OTU_2936	9.38E-01	6.04E-01	3.93E-01	1.08	0.76	Peptoniphilus_tyrrelliae_96	Bacteria_Firmicutes_Clostridia_Clostridiales_Peptoniphilaceae_Peptoniphilus_100
OTU_33534	7.07E-01	6.59E-01	4.06E-01	0.73	0.99	Enterococcus_faecalis_98	Bacteria_Firmicutes_Bacilli_Lactobacillales_Enterococcaceae_Enterococcus_78
OTU_3623	9.64E-01	6.04E-01	3.22E-02	1.25	0.54	Escherichia_albertii_88	Bacteria_Proteobacteria_53_Gammaproteobacteria_53_Enterobacterales_52_Enterobacteriaceae_52_Pseudescherichia_52
OTU_3838	8.48E-01	9.75E-01	9.41E-01	1.02	1.05	Agathobacter_ruminis_90	Bacteria_Firmicutes_70_Clostridia_70_Clostridiales_70_Lachnospiraceae_70_Bariatricus_31
OTU_4706	9.64E-01	7.44E-01	1.32E-01	0.60	1.03	Enterococcus_faecalis_93	Bacteria_Firmicutes_97_Bacilli_97_Lactobacillales_96_Carnobacteriaceae_63_Isobaculum_26
OTU_51171	1.00E+00	6.04E-01	1.84E-01	0.65	1.43	Kineothrix_alysoides_97	Bacteria_Firmicutes_Clostridia_Clostridiales_Lachnospiraceae_Kineothrix_22
OTU_79888	7.57E-01	6.72E-01	3.65E-01	0.55	0.79	Enterococcus_faecalis_98	Bacteria_Firmicutes_Bacilli_Lactobacillales_Enterococcaceae_92_Enterococcus_84
OTU_8078	7.57E-01	6.05E-01	2.56E-01	0.78	1.13	Hungatella_hathewayi_97	Bacteria_Firmicutes_Clostridia_Clostridiales_Lachnospiraceae_Hungatella_99

	Significance for passage number (Model 1)	
	**pFDR<0.05 in MCG-3**	**pFDR<0.05 in MCG-3B**
**Number of OTUs**	0	2
**Percentage of OTUs**	0.00%	0.09%
**Total number of OTUs**	2336	

Number and percentage of total OTUs significantly affected by passage number

	Significance for NDG presence (Model 2)		
	**pFDR<0.05**	**pFDR<0.01**	**pFDR<0.001**
**Number of OTUs**	1070	535	226
**Percentage of OTUs**	45.80%	22.90%	9.67%
**Total number of OTUs**	2336		

Number and percentage of total OTUs significantly affected by NDG presence
